# Temperature‐Controlled Mechanochemistry for the Nickel‐Catalyzed Suzuki–Miyaura‐Type Coupling of Aryl Sulfamates via Ball Milling and Twin‐Screw Extrusion.**

**DOI:** 10.1002/anie.202210508

**Published:** 2022-10-05

**Authors:** Robert R. A. Bolt, Sarah E. Raby‐Buck, Katharine Ingram, Jamie A. Leitch, Duncan L. Browne

**Affiliations:** ^1^ Department of Pharmaceutical and Biological Chemistry University College London (UCL) School of Pharmacy 29-39 Brunswick Square, Bloomsbury London WC1N 1AX UK; ^2^ Syngenta, Jealott's Hill International Research Centre Bracknell, Berkshire RG42 6EY UK

## Abstract

The use of temperature‐controlled mechanochemistry to enable the mechanochemical nickel‐catalyzed Suzuki–Miyaura coupling is herein described. Transitioning from a capricious room‐temperature protocol, through to a heated, PID‐controlled programmable jar heater manifold was required to deliver an efficient method for the coupling of aryl sulfamates (derived from ubiquitous phenols) and aryl boronic acid species. Furthermore, this process is conducted using a base‐metal nickel catalyst, in the absence of bulk solvent, and in the absence of air/moisture sensitive reaction set‐ups. This methodology is showcased through translation to large‐scale twin‐screw extrusion methodology enabling 200‐fold scale increase, producing decagram quantities of C−C coupled material.

The nickel catalyzed Suzuki–Miyaura‐type coupling of aryl sulfamates and boronic acid derivatives enabled by temperature‐controlled mechanochemistry via the development of a programmable PID‐controlled jar heater is reported. This base‐metal‐catalyzed, solvent‐free, all‐under‐air protocol was also scaled 200‐fold using twin‐screw extrusion technology affording decagram quantities of material.

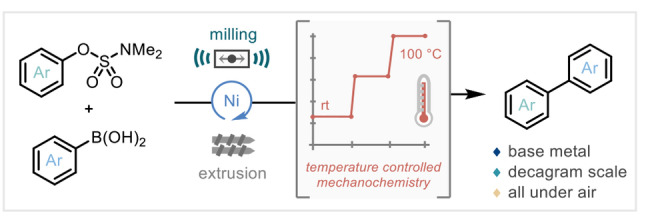

## Introduction

The fusion of an aryl (pseudo)halide and a boronic acid/ester species via Suzuki–Miyaura‐type coupling mechanisms has become one of the most commonplace retrosynthetic disconnections in organic chemistry.^[1]^ Due to its reliable and robust nature, the (usually) palladium‐catalyzed cross coupling protocol is one of the most dependable transformations used in industrial settings, especially in late‐stage functionalization and the rapid assembly of diverse molecules.^[2]^

**Scheme 1:**
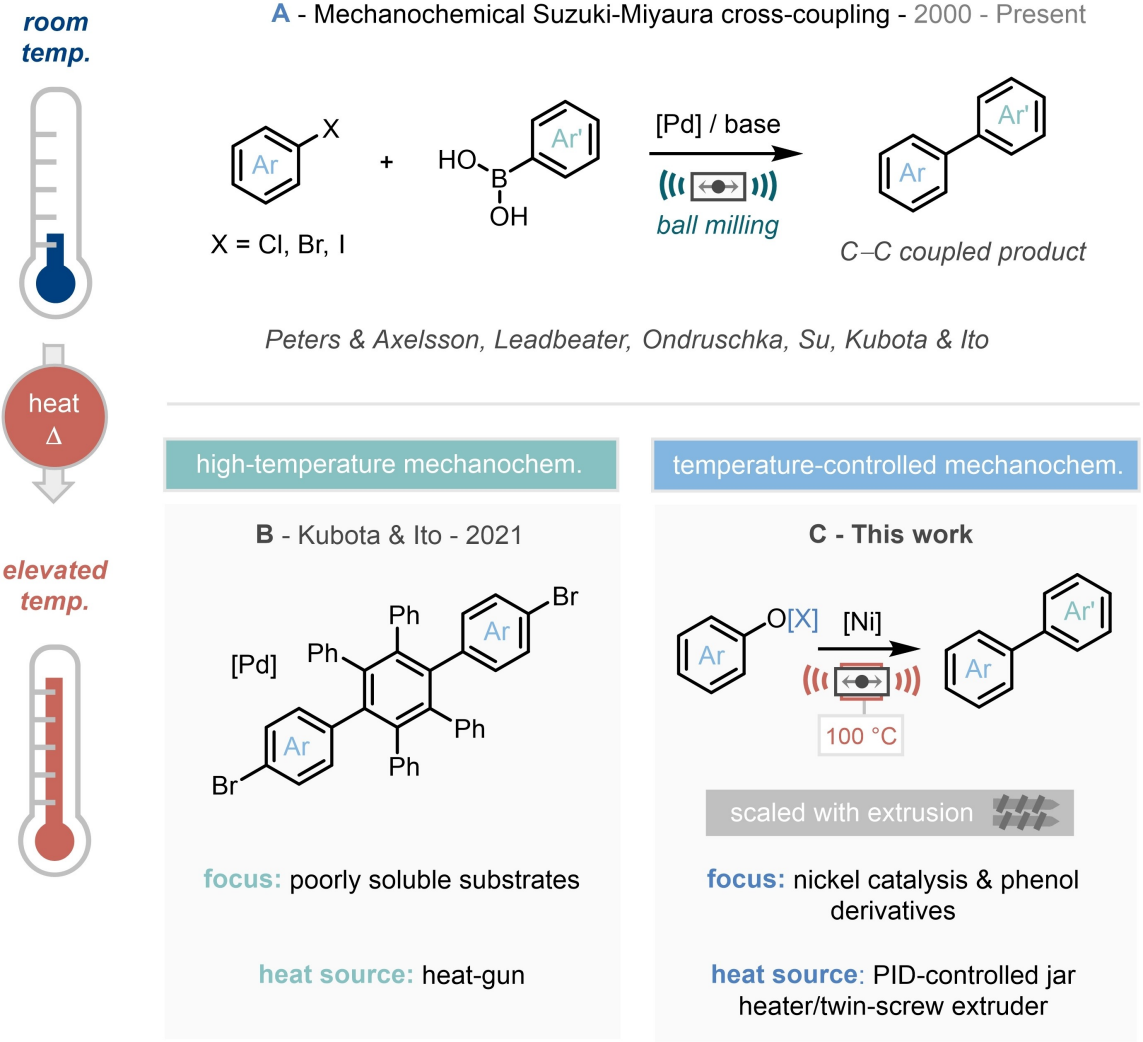
Suzuki–Miyaura‐type cross coupling in mechanochemistry. A) Room temperature coupling using palladium catalysis. B) High‐temperature Suzuki–Miyaura coupling. C) This work on temperature‐controlled nickel catalysis with phenol derivatives.

Due to the importance of Suzuki–Miyaura chemistry, this transformation has also been one of the main targets in the development of mechanochemical cross‐coupling protocols.^[3]^ Research interest and endeavors in mechanochemistry have been growing in recent years, not only as a sustainable method for reducing solvent waste^[4]^—which in turn drives more favorable process‐mass intensity (PMI)^[5]^ and E‐factor^[6]^ metrics—but also that this enabling technology can provide drastically reduced reaction times, improved/complementary selectivity to established solution‐phase methodology,^[7]^ as well as negating the (often required) use of the air/moisture sensitive set‐ups.^[8]^ To this end, pioneering work from Peters & Axelsson,^[9]^ Leadbeater,^[10]^ Ondrushcka,^[11]^ Su,^[12]^ and Kubota and Ito^[13]^ has explored the mechanochemistry‐driven palladium‐catalyzed Suzuki–Miyaura coupling (Scheme 1A).^[14]^ Within this context, previous reports have demonstrated the opportunities that high‐temperature mechanochemistry can provide challenging Suzuki–Miyaura‐type coupling protocols.^[15]^

The ability to carry out mechanochemical protocols at variable temperatures (either below or above room temperature) has witnessed a sustained challenge and has only been explored in select examples to date. As early as 2003, Kaupp and co‐workers disclosed the use of the custom made double walled stainless‐steel jars equipped with fittings capable of circulating heated or cooled liquid through the jar during milling, applying this to the mechanochemical synthesis of arylboronic ester species.^[15]^ This study has inspired a small number of reports in the past two decades, and in 2021, Kubota and Ito revealed a landmark step in the use of high‐temperature mechanochemistry, by employing a heat‐gun clamped above the milling jars to engage an array of poorly soluble substrate materials in Suzuki–Miyaura cross‐coupling protocols (Scheme 1B).[^15^, ^17^]

At the outset of this project, we were intrigued to transition mechanochemical Suzuki–Miyaura methodology away from the stark over‐reliance on palladium complexes to more earth‐abundant, inexpensive, base‐metal nickel‐based catalyst systems. Further to this, to integrate a new family of precursors into this chemistry—and due to past success with nickel systems^[18]^—the use of activated phenol inputs was prioritized.

To realize this proposed reaction blueprint, we deduced that recourse to temperature control may be critical to engage the challenging base‐metal catalytic protocol. Drawing from these targets, landmark prior reports, and our own previous experience in ball‐milling‐enabled cross‐coupling chemistry, herein we report our findings (Scheme 1C).

## Results and Discussion

### Preliminary Experiments

**Scheme 2:**
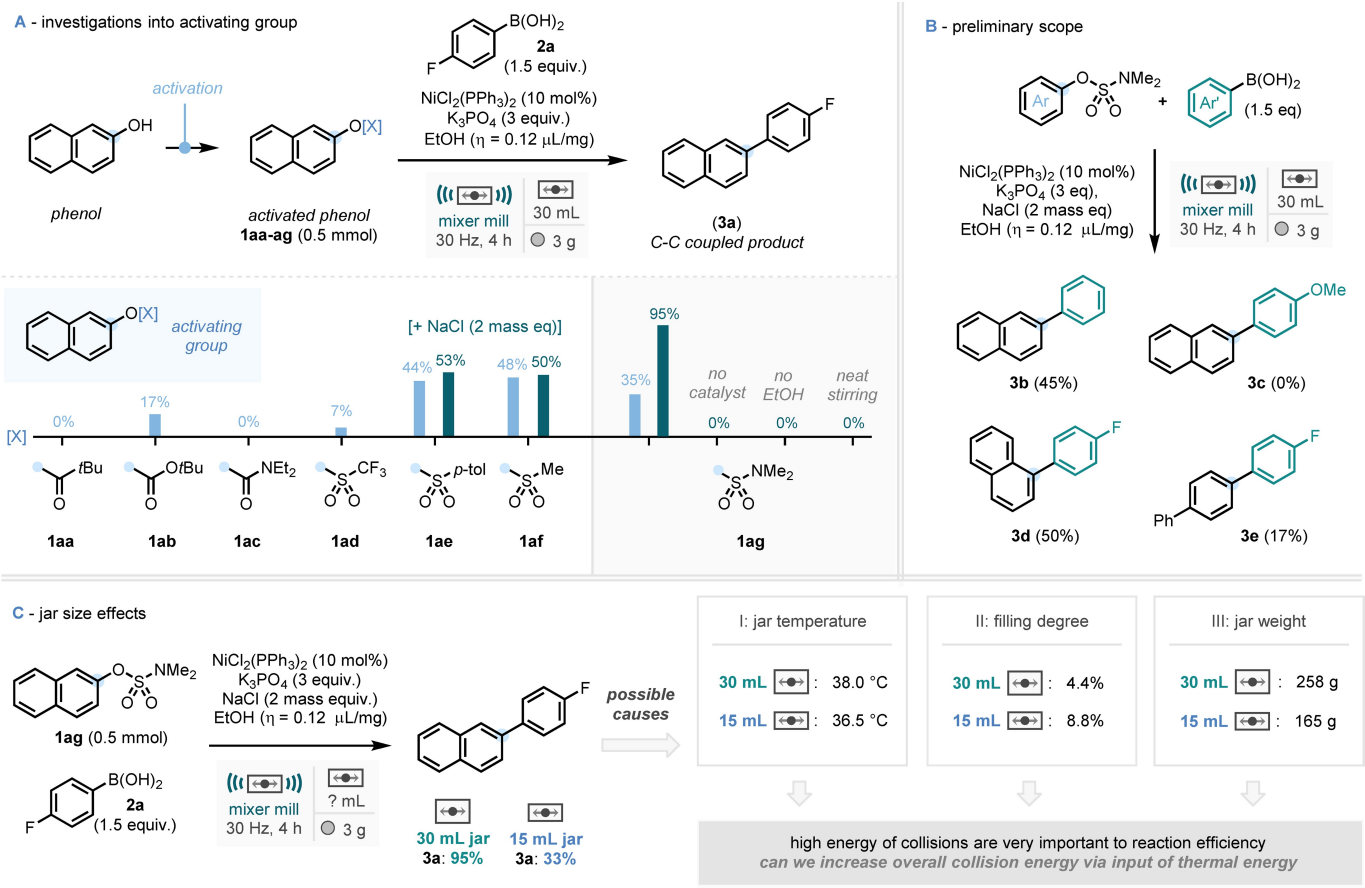
Initial studies into the nickel‐catalyzed Suzuki‐type coupling of activated phenols. A) Exploration of different activating groups for phenols. B) Preliminary scope on small selection of sulfamates and aryl boronic acids. C) Investigations into the profound difference in efficiency of different jar sizes.

Initially applying conditions from archetypal solution‐phase nickel‐catalyzed Suzuki–Miyaura‐type coupling protocols,^[19]^ preliminary investigations (see Supporting Information for full details) unveiled a model reaction system employing NiCl_2_(PPh_3_)_2_ (10 mol %) as the base‐metal catalyst, K_3_PO_4_ as the base, and EtOH as a liquid assisted grinding (LAG) agent^[20]^ (0.12 μL mg^−1^, 10 weight %). Optimization studies commenced with the coupling of 4‐fluorophenylboronic acid (**2 a**) and a survey of activated phenols (Scheme 2A). After 4 hours of ball milling at 30 Hz, pivalate (**1 aa**) and carbamate (**1 ac**) resulted in no observed formation of the C−C coupled product (**3 a**), where employment of a Boc‐substituted derivative (**1 ab**) led to a promising but low yield of 17 %. The use of a triflate activating group (**1 ad**) resulted in a surprisingly low yield of 7 % considering their widespread utility. Following this, three further sulfonyl derived activating groups: tosylate (**1 ae**, 44 %), mesylate (**1 af**, 48 %), and sulfamate (**1 ag**, 35 %) resulted in the most promising amounts of product formation. Interrogation of the rheology of the reaction mixture highlighted that the addition of a grinding auxiliary, such as NaCl, may be beneficial to the reaction medium. Whilst the addition of NaCl only led to a slight increase in yield for tosylate (**1 ae**) and mesylate (**1 af**), the yield for sulfamate **1 ag** significantly increased from 35 % in the absence of NaCl to 95 % with inclusion of the grinding auxiliary. From this point on, it is worth noting that the LAG quantities are calculated without consideration of the grinding auxiliary. Finally, control experiments using sulfamate (**1 ag**) demonstrated no reactivity in the absence of catalyst or the ethanol LAG and that stirring the reaction mixture in a standard flask also showed no formation of the C−C coupled product.

Using these conditions, an initial preliminary scope with a small cross‐section of sulfamates and boronic acids was conducted (Scheme 2B). The reaction of phenyl boronic acid (**2 b**) with **1 ag** and the coupling of **2 a** with the isomeric 1‐naphthyl dimethylsulfamate (**1 b**) gave products **3 b** (45 %) and **3 d** (50 %). These results constitute significantly reduced yields vs the model substrate (95 %) considering the seemingly trivial differences. Further to this, employing 4‐methoxy phenyl boronic acid with **1 ag** gave no observed formation of corresponding product **3 c**, whilst using a biphenyldimethylsulfamate derivative gave a poor yield of 17 % (**3 e**) after four hours of milling time.

Over the course of the optimization studies, it was observed that the size of the jar had a profound effect on reaction efficiency (Scheme 2C). Using a smaller 15 mL jar, the model reaction system resulted in a significantly lower yield of 33 % of **3 a**, compared to the model system, using a 30 mL jar (95 %). We reasoned that this could potentially be due to three factors—(i) jar temperature, (ii) filling degree, or (iii) jar weight.

In order to assess the temperature of the reaction in the 15 mL and 30 mL jars, thermocouples were used to gain real‐time temperature data over the 4‐hour reaction period.^[21]^ The data demonstrates that both processes proceed to similar plateau/steady‐state temperature (38.0 °C for 30 mL, 36.5 °C for 15 mL, see Supporting Information for heating profiles). The filling degree within the milling jar (comparison of total volume of reagents and ball against total volume of jar cavity)^[22]^ affects the mechanical energy transferred in each collision, the frequency of successful collision and contributes to the latent heat of milling.^[23]^ In this instance the lower filling degree of the 30 mL reaction will lead to higher energy collisions, which could be responsible for the difference in observed reactivity. Finally, heavier jars lead to higher energy transfer on impact between the ball and the moving jar. To this end, the almost 100 g difference in weight between the 30 mL and 15 mL jars could result in this discrepancy in yield.

Whilst all three of these causes are likely contributors, each one results in the same conclusion that high‐energy collisions are particularly crucial for the efficiency of this reaction. To this end, it was apparent that increasing overall collision energy—which could be achieved via the input of supplementary thermal energy (in addition to the mechanical energy already present)—could prove valuable to the generation of a wider substrate‐scope system.^[24]^

### Heat Source Studies

**Scheme 3:**
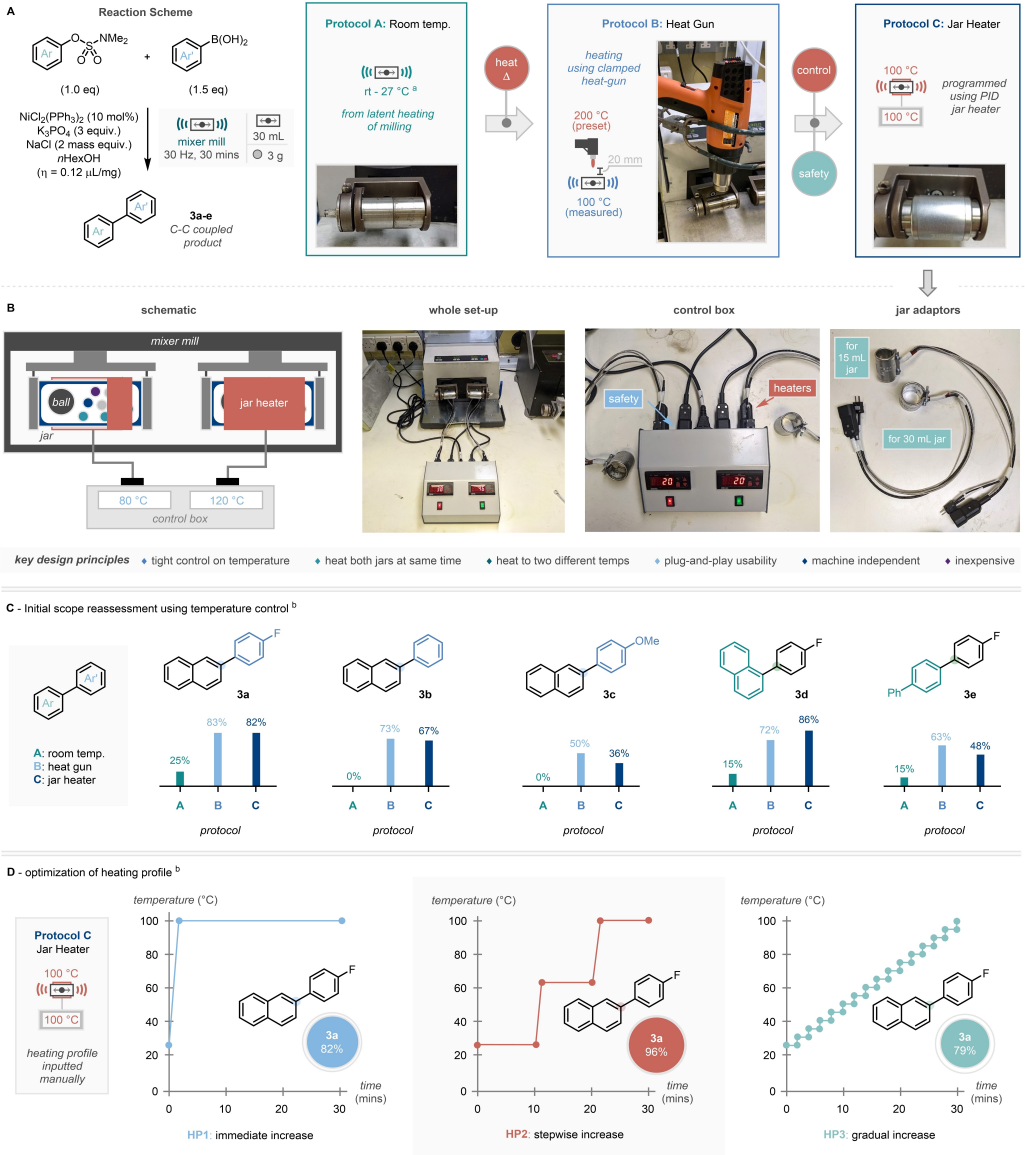
Temperature‐controlled Suzuki–Miyaura coupling. A) Evolution of heating devices in temperature‐controlled mechanochemistry from heat gun to PID‐controlled jar heater. B) Schematic and pictorial overview of the prototyped jar heater. C) Results from different heating protocols on subset of compounds. D) Fine‐tuning of heating profile for the Suzuki–Miyaura cross‐coupling. ^a^ Jar temperature produced by latent heat of milling after 30 minutes reaction time. ^b^ Conditions as per top left of scheme – numbers given are calculated via ^1^H NMR analysis of the crude reaction mixture against mesitylene as an internal standard.

With this in mind and as a prelude for direct comparisons of a range of heating processes, the optimized room temperature reaction (Scheme 2A) was repeated replacing ethanol with hexanol as LAG (boiling point 157 °C) and the milling time was shortened to 30 minutes, to better highlight any potential improvements from applying heat (Protocol A, Scheme 3A).

We initially employed a heat‐gun‐based set up similar to reports by Kubota and Ito.^[17]^ In this case a controllable heat gun is placed approximately 20 mm directly above the milling vessel and set to 200 °C, the measured temperature of the reaction vessel was 100 °C.^[25]^ Conducting the same “5‐compound” scope (from Scheme 2B) at this effective milling temperature of 100 °C, pleasingly led to increased yields for all 5 substrate combinations. Most markedly, reactions that didn't react at all after 30 minutes milling at room temperature (**3 b** and **3 c**) were “turned on” with yields of 73 % and 50 % respectively when heating was applied (Protocol B, Scheme 3C). It is noteworthy that these increases in reactivity are not solely due to a phase change (melting of the sulfamate, **1 ag**: mp=71 °C and **1 b**: mp=76 °C) but also in substrates which remain solid despite the temperature increase (**1 c**: mp=113 °C), showing temperature‐based increase in reactivity.

Despite these initially promising results, using the heat gun raised concerns over safety and precision of heating. Using the heat gun meant that the mill's cover had to be manually removed, requiring its safety features to be bypassed. As previously mentioned, the temperature reached by the reaction vessel is significantly lower (by around 50 %) than the temperature set on the heat gun representing a wasteful energy loss. Furthermore, the heating is also undirected, heating not just the reaction vessel but the whole arm of the mill^[26]^ and the neighboring reaction vessel.

To address these concerns a more accurate, controllable jar heating device was designed, developed, prototyped, and tested in house (Scheme 3B).^[17g]^ The main device consisted of a PID controller and interchangeable band heaters with internal thermocouples. The mill‐independent device allows the precise heating of two jars to individual temperatures and can be used for a range of different jar sizes without compromising the in‐built safety features of the milling device.^[27]^ Examining the 5‐compound scope with the heating device set to accurately heat to and maintain 100 °C demonstrated comparable results to that of the heat gun and superior to that of the room temperature alternative (Protocol C, Scheme 3C).

### Heating Regime Studies

The introduction of heating—specifically, controlled heating—to this mechanochemical Suzuki–Miyaura manifold also introduced two new parameters, often not readily available in ball milling: heating profile and temperature set‐point. The first of these was optimized using the three proposed heating profiles including: immediate (HP1, Scheme 3D), stepwise (HP2), and gradual increase (HP3).^[28]^ It was found that application of immediate (HP1) and gradual (HP3) heat resulted in similar yields of 82 % and 79 % respectively, however the use of a stepwise heating profile with a middle temperature halfway between room temperature and the end temperature (63 °C) gave an excellent yield of 96 % (HP2).^[29]^

The second parameter; temperature set‐point, was explored in a progressive manner between 80 °C and 130 °C using electronically varied boronic acids **2 a**–**e** (Scheme S1, see Supporting Information for full details). Analysis of this spread of yields revealed that the highest and most consistent yields (determined by analysis of the lowest standard deviation) were achieved at 100 °C, therefore this was brought forward as the “go‐to” temperature for further scope elaboration. Despite this, for electron‐rich boronic acid systems higher temperatures (120–130 °C) were shown to improve reaction efficiency so will be employed for comparison with similar derivatives in the reaction scope.

### Reaction Scope

**Scheme 4:**
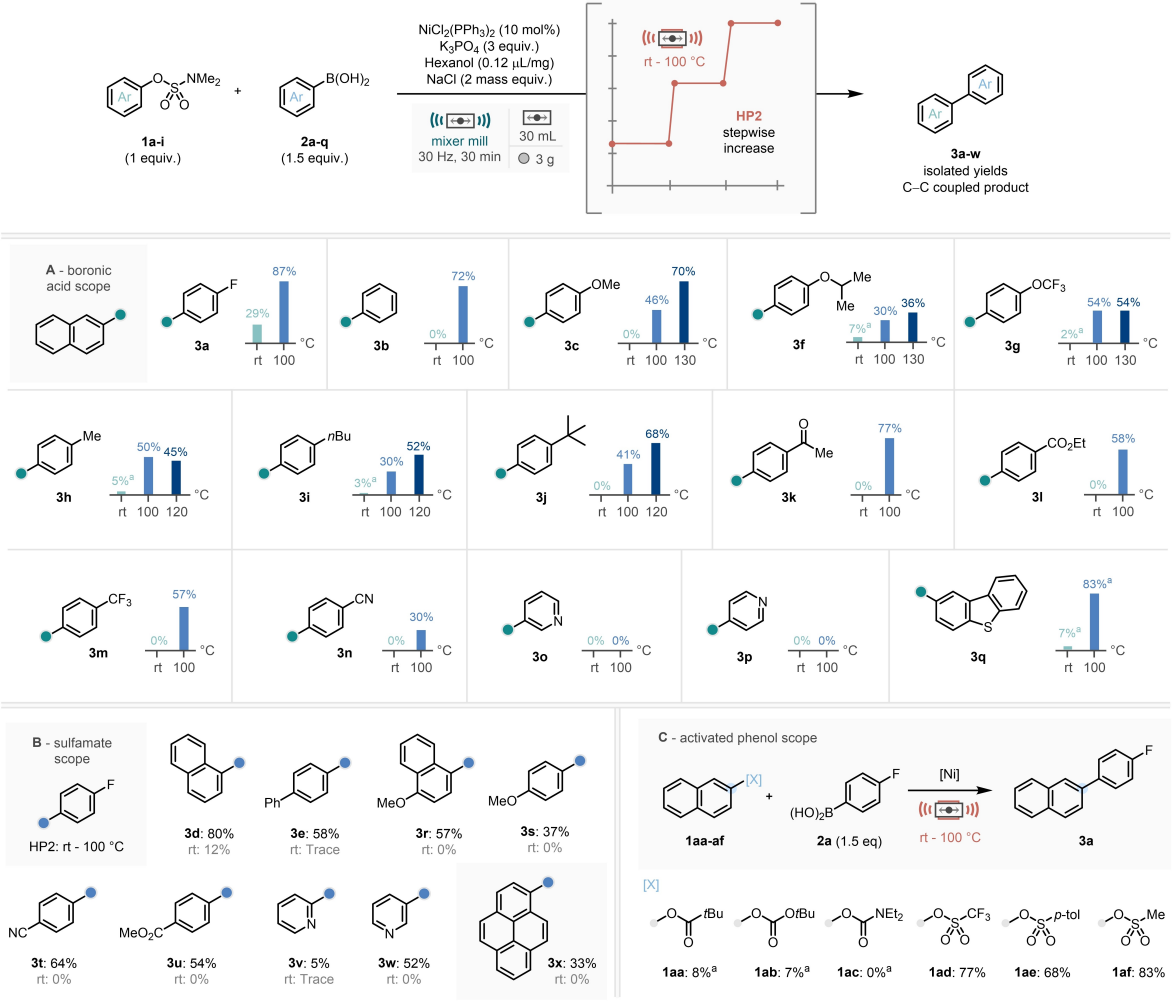
Scope of the temperature‐controlled mechanochemical nickel‐catalyzed Suzuki–Miyaura‐type coupling. A) Boronic acid scope. B) Sulfamate scope. C) Revisiting activated phenol scope. ^a 1^H NMR yield calculated vs. mesitylene as an internal standard.

Using all the information gained from our optimization studies on reaction conditions, heating apparatus, heating profile, and initial temperature of choice, the scope and limitations of this reaction methodology were explored (Scheme 4). This was carried out using a library of aryl sulfamates and boronic acid species, where comparisons to room temperature mechanochemical reactions are also shown.

Studying boronic acid derivatives of a variety of electronic properties revealed that under our new system a selection of substrates performed well (**3 a**–**n**), with a slight drop in yield for electron‐rich alkoxy‐substituted substrates (**3 c** & **3 f**, Scheme 4A).^[30]^ Despite this, aligned with our above investigations, across the board lower yields could be generally improved through operating at a higher reaction temperature of 120 or 130 °C. Whilst unfortunately pyridine derivatives were unsuccessful in this methodology (**3 o**–**p**) a dibenzothiophene heteroaromatic boronic acid delivered the C−C coupled product in excellent conversions at 100 °C (**3 q**).^[31]^ Following this a survey of aryl sulfamates were studied (Scheme 4B), all showing modest to good reaction efficiency (**3 d**–**e**, **3 s**–**u**). Importantly heteroaromatic 3‐pyridyl‐sulfamate derivative **3 w** was demonstrated to engage well with this chemistry. Furthermore, one key opportunity which mechanochemistry offers is the processing of traditionally insoluble products.^[15]^ In this vein, the large aromatic pyren‐1‐ol sulfamate derivative performed well in this methodology, achieving a moderate but promising yield of the biaryl **3 x**.

It is noteworthy that—excluding naphthalene substrates **3 a** and **3 d—**all substrates in the scope studies delivered C−C coupled product in lower than 10 % yield when the reaction was run without application of any external heating. This serves to consolidate the opportunities that temperature‐controlled mechanochemistry can offer in developing new solvent‐free methodology, especially in base‐metal catalysis.

At this point we were curious to revisit the other activated alcohol species to explore whether our optimised temperature‐controlled system would improve the efficiency of their participation in the reaction (Scheme 4C). Whilst carbonate/carbamate derivatives (**1 aa**–**ac**) remained very low yielding, the sulfonate derivatives were impressive under these new conditions, especially the triflate (**1 ad**, 77 %) structure which previously only afforded C−C coupled product in 7 % yield when conducting the reaction methodology without supplementary thermal energy for 4 hours (Scheme 2A).

### Scale‐Up using Twin‐Screw Extrusion

The scalability of mechanochemical transformations enabled by ball milling (especially mixer mills) has remained a question in need of an answer to truly assert mechanochemistry as a penetrative sustainable technology, especially in industrial settings. To this end, the last 5 years has witnessed endeavors into the application of continuous twin‐screw extrusion as a “flow chemistry” solution to this problem, where multiple research teams have provided key insights into the processing of large quantities of solids and slurries.^[32]^ In addition to the variables provided in ball‐milling, twin‐screw extrusion offers further new unique reaction parameters to consider. These include feed rate, screw speed, and screw configuration. Notably extruders allow the fine control of reaction temperature, in our case along 7 individually heated temperature segments.^[33]^ With this in mind, we reasoned that extrusion could be applicable to our temperature‐controlled Suzuki–Miyaura‐type coupling.

**Scheme 5:**
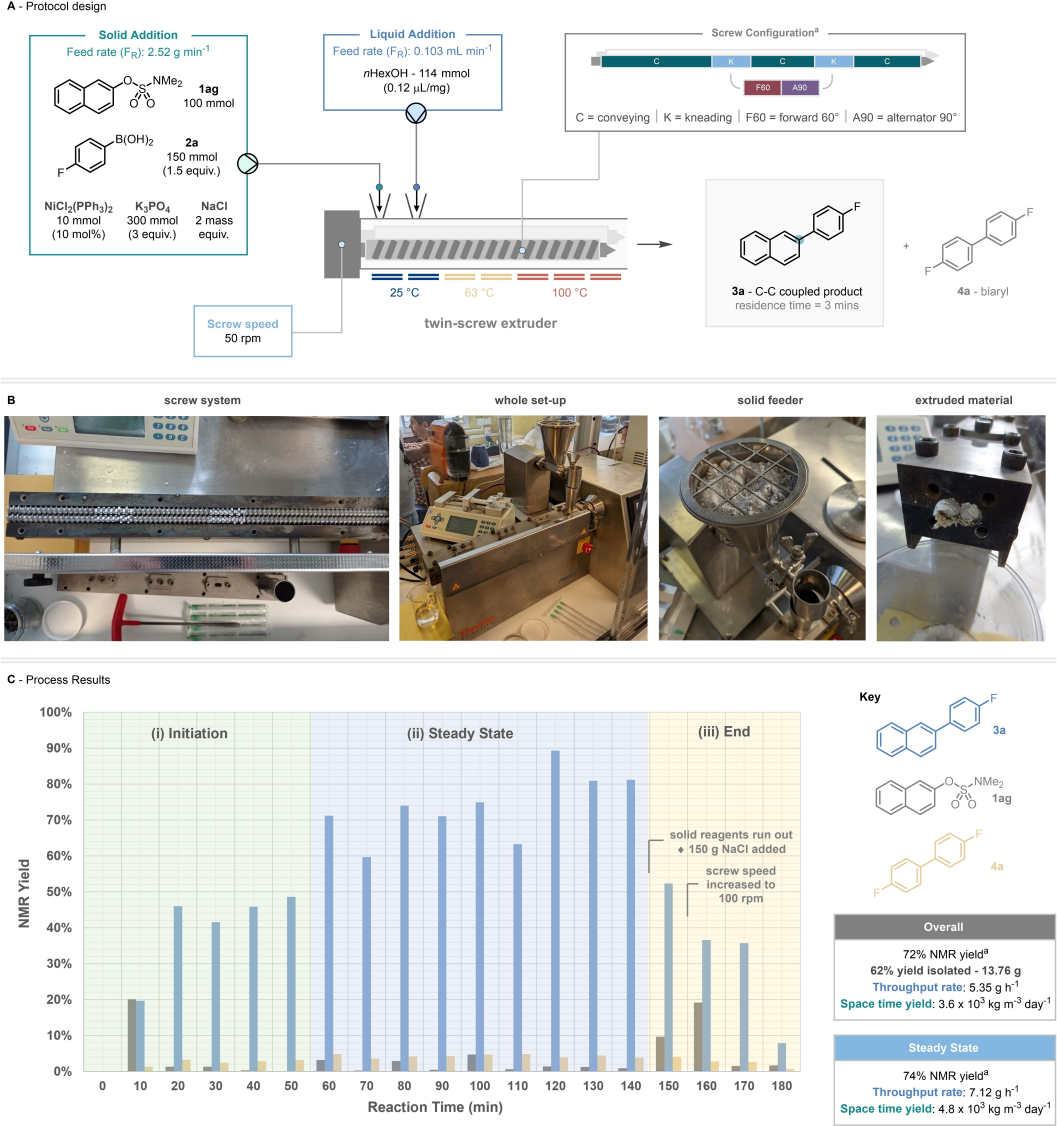
Upscaling the mechanochemical nickel‐catalyzed Suzuki–Miyaura coupling via twin‐screw extrusion. A) In depth protocol design. B) Pictorial representation of extrusion run. C) Results from the 100 mmol‐scale extrusion process including data on throughput rate and space time yield (STY).

The extrusion protocol was set up using a screw speed of 50 rpm, a screw configuration containing forward 60° and alternator 90° kneading sections, and temperature elements set to mimic our three‐stage heating protocol (HP2, Scheme 5A). Scaling our methodology up 200‐fold to a 100 mmol scale of sulfamate starting material, all the solid materials—sulfamate (**1 ag**), boronic acid (**2 a**, 150 mmol), NiCl_2_(PPh_3_)_2_ (10 mmol), K_3_PO_4_ (300 mmol), and NaCl (2 mass equiv.)—were mixed manually in a beaker with a spatula and then added to a volumetric hopper over the solid feed port. In addition to this, a syringe was loaded with *n*HexOH (114 mmol) and the needle positioned over a second liquid addition port. To achieve an input rate of 0.722 mmol min^−1^, the solid feeder was set to add 2.52 g min^−1^.^[34]^ and the liquid feeder to 0.103 mL min^−1^.

The extrusion process was run for 3 hours—with a residence time (T_R_) of 3 minutes and collecting the extruded material in 10‐minute intervals. Subsequent interrogation of each collected portion determined the yield (by NMR against an internal standard) of coupled product (**3 a**), leftover starting material (**1 ag**), and the biaryl by‐product (**4 a**) and revealed three distinct sections to the reaction process, (i) Initiation, (ii) Steady state, and (iii) End (Scheme 5C).^[35]^

Firstly, an initiation section was observed with low conversion (0–10 mins), and then higher conversions with low yield (20–50 mins). This can be attributed to the extruder achieving a reactor steady state which involves the filling of all kneading sections with crude reaction material. After this point, the screw reactor stays at a steady torque throughout the whole process (in this case ≈1.3 Nm).

In addition to this, analysis of the production of biaryl **4 a** suggests that—as this biaryl product is formed through the reductive activation of the Ni^II^ precursor to the active Ni^0^ catalytic species—first observing 5 % of **4 a** at 60 minutes suggests only at this point do we have full activation of the 10 mol % nickel pre‐catalyst.

Following this we observed sustained periods of high product formation (60–89 %) for the next 90 minutes which we are terming the “steady state”. This productive period only ended after 150 minutes as the solid feed ran out of input material. At this point, NaCl (150 g) was added to the solid feeder and passed through the system to ensure passing of all the reaction mixture out of the screw system.^[36]^ This alteration of the reaction morphology (increased amounts of NaCl compared to other reaction components) feeding into the extruder is witnessed in the final “end” section with a reduction in product formation and increase in **1 ag** remaining. Further to this, screw speed was increased to 100 rpm at 160 minutes to prevent “over‐torquing” of the salt mixture.

Over the course of the full run an NMR yield of 74 % was achieved (based on 100 mmol of sulfamate **1 ag**), with 13.76 g of C−C coupled product isolated after chromatography (62 %). This corresponds to a throughput rate of 5.35 g h^−1^ and a space‐time yield of 3.6×10^3^ kg m^−3^ day^−1^.^[37]^ These figures also include both the initiation and end sections of the reaction process, and theoretically if the process was elongated further by running with more material, it is the steady state section that would predominantly increase. To this end, analyzing the metrics of the steady state section—a throughput rate of 7.12 g h^−1^ and a space time yield of 4.8 ×10^3^ kg m^−3^ day^−1^—reveal the opportunities that twin‐screw extrusion can offer the decagram‐plus construction of C−C bonds. However, we note here that there is still substantial work to be done in characterizing and understanding the dynamics of the reactive extrusion systems, this would lead to better process understanding and potential gains in reaction yield. There is also a requirement to develop more scale‐appropriate purification methods.

Finally, when analyzing the process mass intensity (PMI_(reaction)_)^[38]^ of our process against the most similar literature process of Kappe and co‐workers^[19a]^—using microwave chemistry to facilitate the coupling of aryl sulfamates and aryl boronic acids—show our PMI_(reaction)_—large scale: 21.76 and PMI_(reaction)_—small scale: 18.59 are significantly improved vs. the microwave alternative: 42.77.^[39]^

## Conclusion

In conclusion, a temperature‐controlled mechanochemical Suzuki–Miyaura‐type coupling of aryl sulfamates and boronic acid species enabled by nickel catalysis has been realized. This was facilitated by the designing, prototyping, and building of a PID‐controlled programmable jar heater which enabled fine‐tuned control of temperature of our reaction system. Through optimization of heating regimes and temperature screens an optimal set of conditions were uncovered which were applied to a selection of aryl boronic acids and aryl sulfamates—each time demonstrating improved reactivity when benchmarked against the, often unsuccessful, room temperature reaction conditions.

Further to this, successful preliminary translation to continuous twin‐screw extrusion technology has been demonstrated, enabling a 200‐fold scale up and synthesis of over 13 g of C−C coupled material—all in the absence of bulk reaction solvent, using a base‐metal catalyst system, using a protocol designed on the milligram scale. Work is ongoing to further expand translation from our PID‐controlled programmable jar heater methodologies to large‐scale extrusion protocols.

## Conflict of interest

The authors declare no conflict of interest.

1

## Supporting information

As a service to our authors and readers, this journal provides supporting information supplied by the authors. Such materials are peer reviewed and may be re‐organized for online delivery, but are not copy‐edited or typeset. Technical support issues arising from supporting information (other than missing files) should be addressed to the authors.

Supporting Information

Supporting Information

Supporting Information

## Data Availability

The data that support the findings of this study are available from the corresponding author upon reasonable request.
