# Supplementary material for: Temperature‐Controlled Mechanochemistry for the Nickel‐Catalyzed Suzuki–Miyaura‐Type Coupling of Aryl Sulfamates via Ball Milling and Twin‐Screw Extrusion
Source: Angew Chem Int Ed Engl. 2022 Oct 5;61(44):e202210508. doi: 10.1002/anie.202210508 (PMC9828252; doi:10.1002/anie.202210508)
Supplement: Supplementary file 3 — Supporting Information [file ANIE-61-0-s003.pdf]

Angewandte Chemie
Eine Zeitschrift der Gesellschaft Deutscher Chemiker

Supporting Information

# Temperature-Controlled Mechanochemistry for the Nickel-Catalyzed Suzuki–Miyaura-Type Coupling of Aryl Sulfamates via Ball Milling and Twin-Screw Extrusion

*R. R. A. Bolt, S. E. Raby-Buck, K. Ingram, J. A. Leitch*, D. L. Browne**

**Table of Contents**


1. General Experimental ........ S3
2. Equipment used ........ S4
3. Preliminary optimisation studies ........ S8
4. Temperature-controlled mechanochemistry set-ups ........ S9
   4.1. Heat gun set-up ........ S9
   4.2. Heat device set-up ........ S11
5. Jar heater details ........ S13
   5.1: Parts lists ........ S13
   5.2: Images ........ S14
   5.3: Internal wiring schematic ........ S15
6. Monitoring reaction temperatures ........ S16
7. Further experiments ........ S18
8. Variable temperature experiments using HP2 ........ S20
9. Sustainability Metrics ........ S21
   9.1: Process Mass Intensity (PMI) calculations ........ S21
   9.2: E-factor calculations ........ S22
   9.3: Space time yield (STY) calculations ........ S23
10: Extrusion protocols ........ S24
11. Experimental procedures ........ S28
   11.1 Synthesis of starting materials ........ S28
      11.1.1. Procedures for other activated phenol starting materials ........ S28
      11.1.2. General Procedure A: Synthesis of aryl sulfamates ........ S31
      11.1.3. General procedure B: Hydroxyl pyridine dimethylsulfamate synthesis ........ S33
   11.2. Synthesis of C-C coupled products ........ S35
      11.2.1. General Procedure C: Mechanochemical nickel-catalysed Suzuki-Miyaura cross coupling... S35
12. NMR spectra ........ S44
13. References ........ S87

## 1. General Experimental

All chemicals were obtained from commercially available sources and used without additional purification unless stated otherwise.

Ball milling was carried out on a Retsch MM 400 Mixer mill and an IST500. Stainless steel milling jars and stainless-steel grinding balls were used unless stated otherwise.

**Thin layer chromatography (TLC)** was carried out on Merck TLC 60Å silica gel sheets and visualised using ultraviolet light of wavelength 254 nm.

**Flash column chromatography (FFC)** was performed using Merck/Sigma Aldrich silica gel (40-60 Å) as the stationary phase.

**$^{1}$H NMR, $^{13}$C NMR and $^{19}$F NMR spectra** were obtained on a Bruker Avance 400 at 400.13 MHz ($^{1}$H NMR at 400 MHz, $^{13}$C NMR at 101 MHz), Bruker Avance 500 fitted with a cryoprobe, at 500 MHz ($^{1}$H NMR at 500 MHz, $^{13}$C NMR at 126 MHz) and Bruker Avance Neo 700 at 700 MHz ($^{19}$F NMR at 659 MHz, $CDCl_3$) at ambient temperature with chloroform-d as the deuterated solvent unless stated otherwise. All chemical shifts δ are reported in parts per million (ppm) and are referenced to the residual solvent peak ($CHCl_3$ – 7.26 and 77.16 ppm for $^{1}$H and $^{13}$C respectively). Data for $^{1}$H NMR are reported as: chemical shift (multiplicity, coupling constant (J, reported in Hertz - Hz) and integration). $^{13}$C NMR are reported as only chemical shift. Multiplicities are given as: s (singlet), d (doublet), t (triplet), q (quartet), h (hextet), m (multiplet), dd (doublet of doublet), ddd (doublet of doublet of doublets), dddd (doublet of doublet of doublet of doublets). Complex splitting patterns are reported as m.

High resolution mass spectral (**HRMS**) data were obtained on a Micromass Q-TOF Premier Tandem Mass Spectrometer coupled to nano acquilty LC. Spectra were obtained using electrospray (ES)

Infrared spectra were recorded on an Agilent Cary 630 FTIR spectrometer.

Melting points were measured on a Stuart SMP10 melting point apparatus.

## 2. Equipment used

**Mixer Mills**: Mechanochemical reactions were conducted using a Retsch MM400 mixer mill and an IST500 high-energy mixer mill at the frequency denoted where relevant (below see www.retsch.com and www.insolidotech.org for more details)

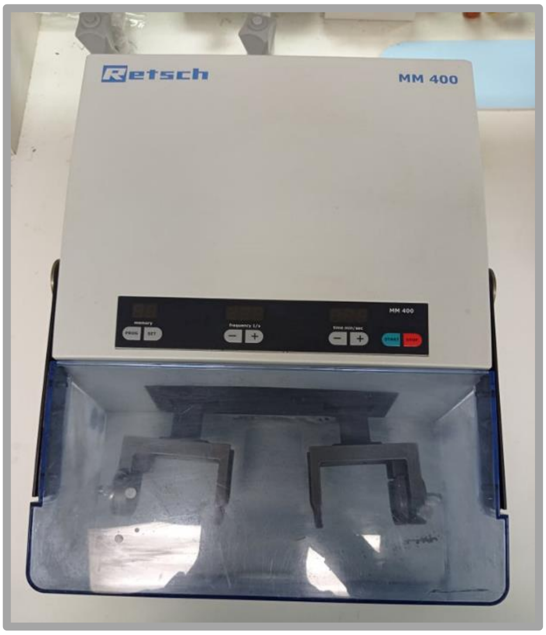


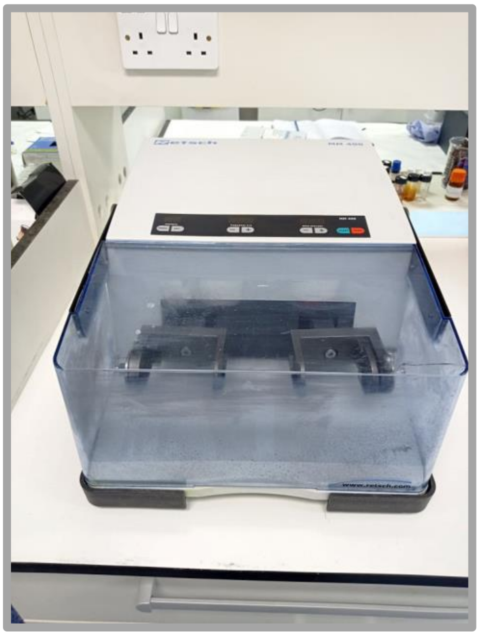

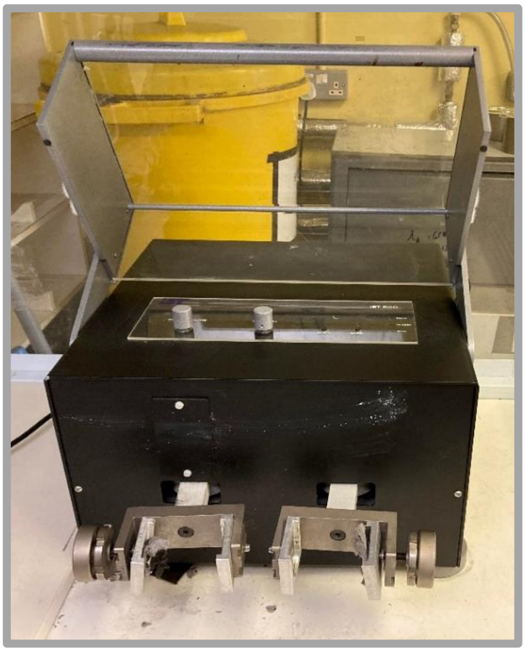

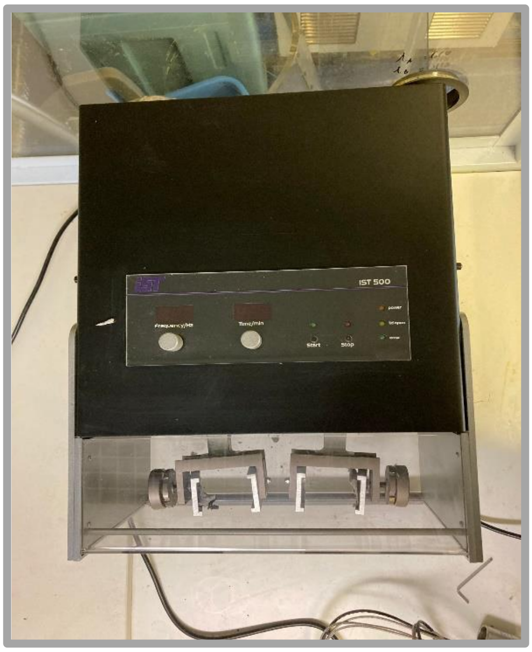

**Jars**: Mechanochemical reactions were conducted using stainless steel jars (316 SS grade) with Teflon seals which were fabricated in-house (15 mL left, 30 mL right).

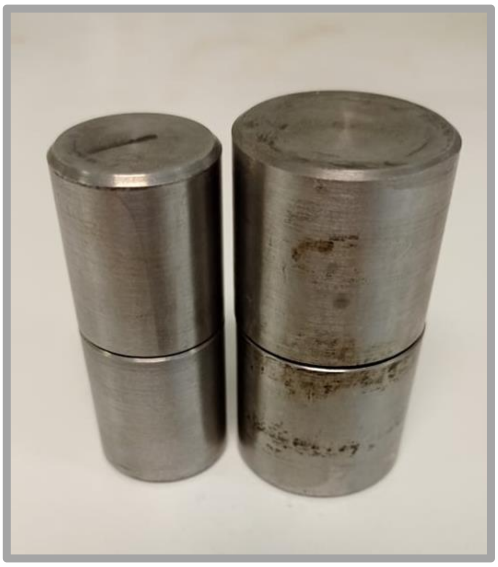

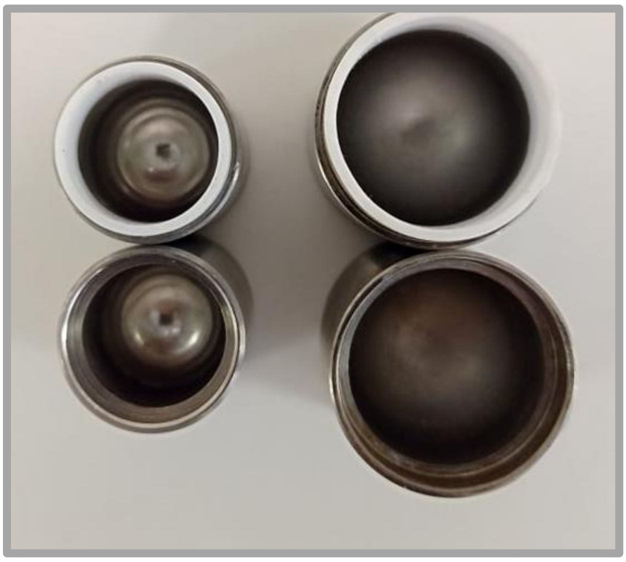

**Balls**: Stainless steel balls (316 SS grade) were purchased from Bearing Boys limited (ww.bearingboys.co.uk) at a variety of sizes (see below - left to right 2 g, 3 g, 4 g, 7 g, 8 g, 12 g)

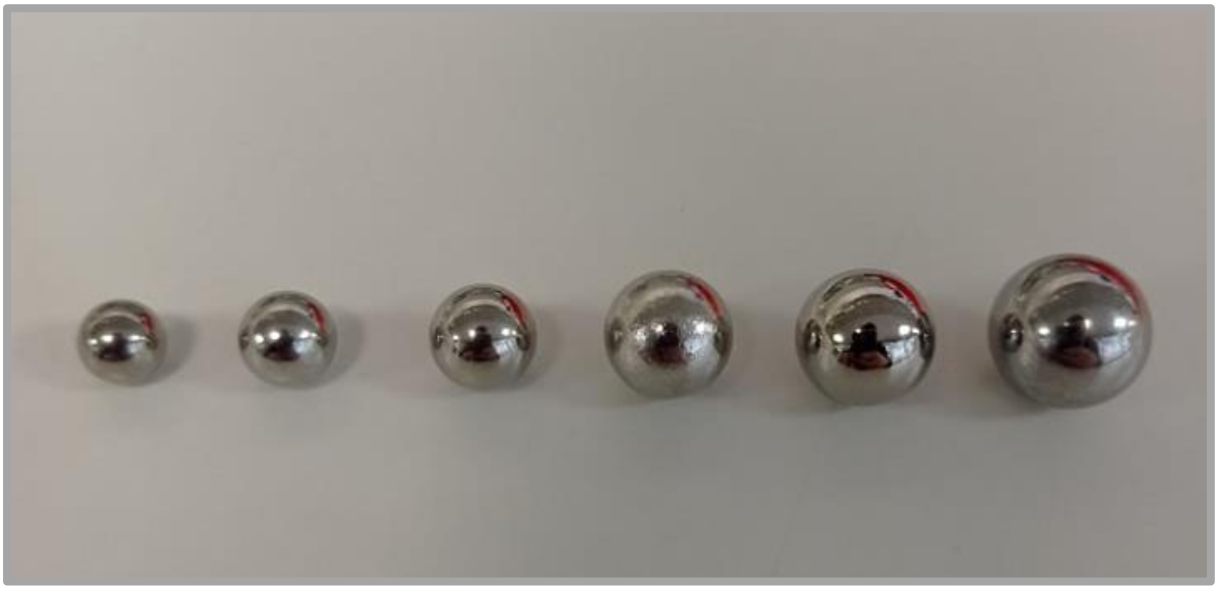

**High-temperature mechanochemistry**: Mechanochemical reactions conducted at high temperature using a heat-gun apparatus were carried out using an evolution HDG200 heat-gun set to the appropriate temperature (see below).

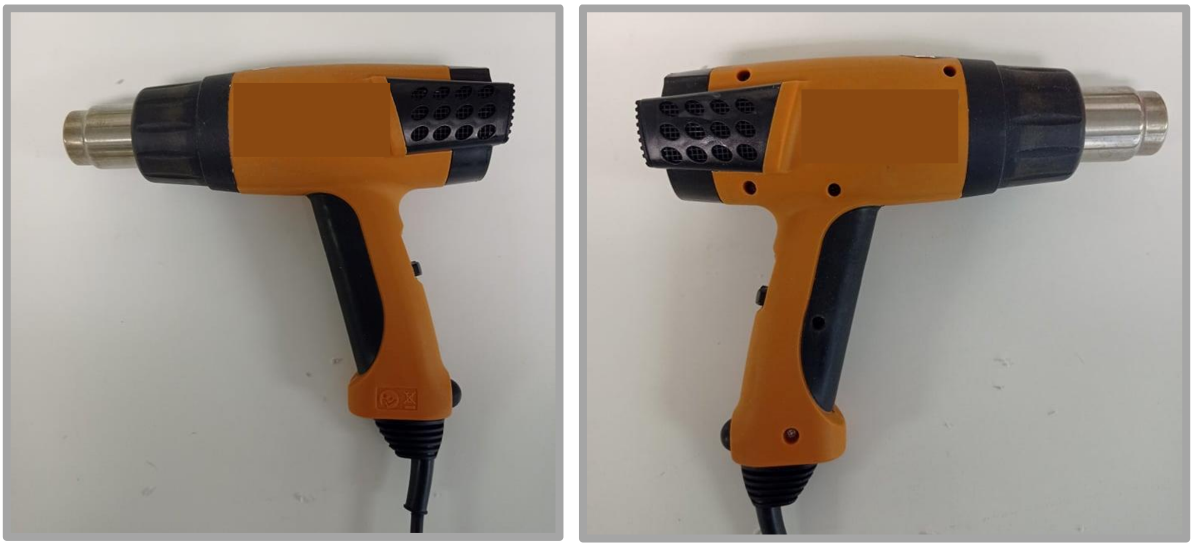

**Retsch MM400 Lid removal**: For heat gun experiments both the lid and the plastic cover on the milling arms need to be removed (see below). The plastic cover on the milling arms are removed using a Phillips screwdriver.

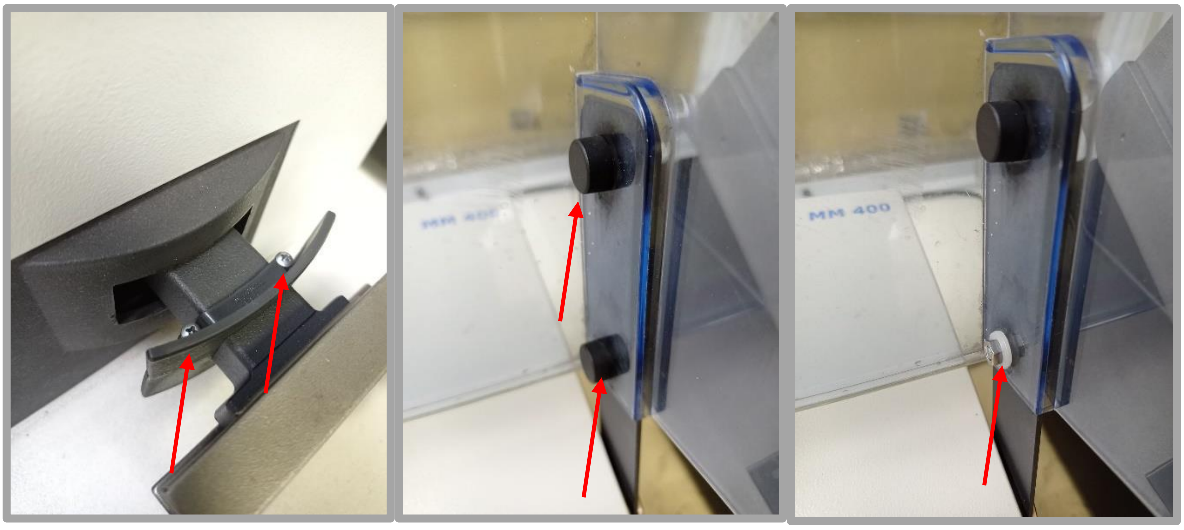


The plastic safety lid is removed by first removing the plastic caps over the bolt and then removing the nut and plastic washer (see above).

**Retsch MM400 base plate removal**: For heat band experiments both the milling arms covers, and the base plate needs to be removed. See above to remove the plastic covers on the milling arms. To remove the base plate, place the instrument on its side and remove the three screws with a M4 Allen key (see below).

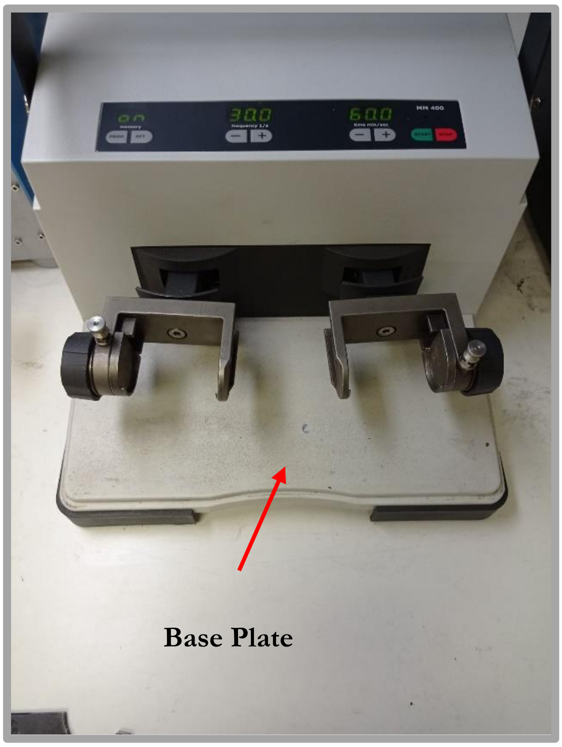
Base Plate

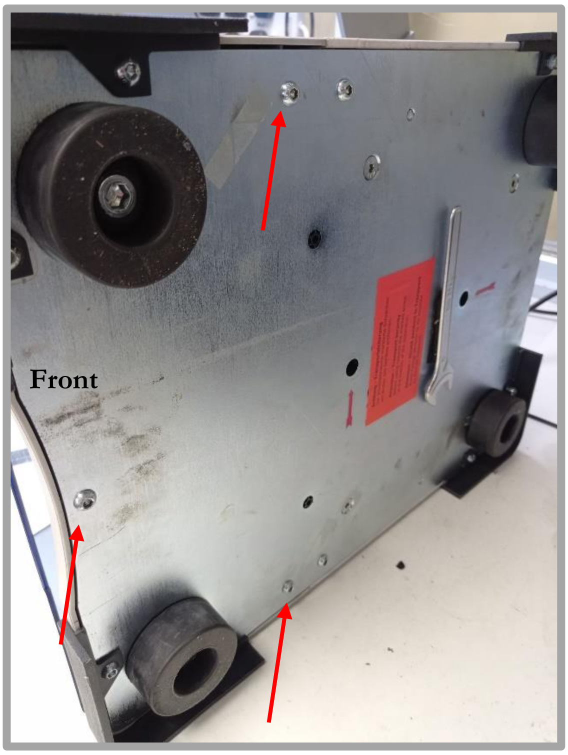
Front

## 3. Preliminary optimisation studies

**Table S1**. Preliminary optimisation studies on naphthyl sulfamate derivative

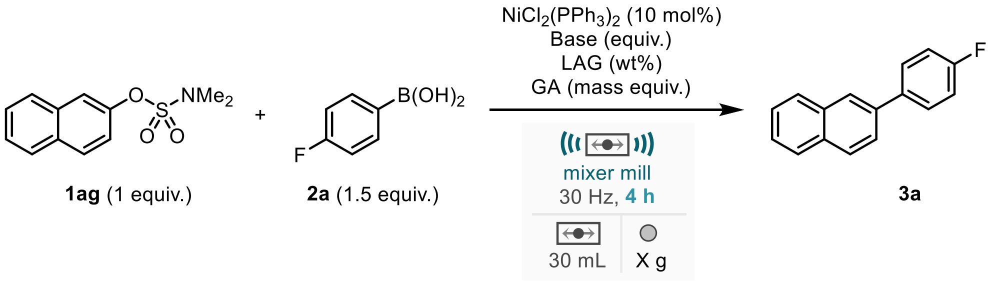


| Entry | LAG (wt%)[a] | GA (mass equiv.)[b] | Ball Mass (g) | Base (equiv.) | Yield (%)[c] |
|---|---|---|---|---|---|
| 1[d] | None | None | 3 | $K_3PO_4$ (3) | - |
| 2 | None | None | 3 | $K_3PO_4$ (3) | 0 |
| 3 | EtOH (5) | None | 3 | $K_3PO_4$ (3) | 57 |
| 4 | EtOH (10) | None | 3 | $K_3PO_4$ (3) | 34 |
| 5 | EtOH (20) | None | 3 | $K_3PO_4$ (3) | 0 |
| 6 | EtOH (5) | NaCl (0.5) | 3 | $K_3PO_4$ (3) | 62 |
| 7 | EtOH (5) | NaCl (1.0) | 3 | $K_3PO_4$ (3) | 65 |
| 8 | EtOH (10) | NaCl (0.5) | 3 | $K_3PO_4$ (3) | 57 |
| 9 | EtOH (10) | NaCl (1.0) | 3 | $K_3PO_4$ (3) | 74 |
| **10** | **EtOH (10)** | **NaCl (2.0)** | **3[e]** | **$K_3PO_4$ (3)** | **95 (75)[f]** |
| 11 | EtOH (10) | NaCl (1.0) | 2 | $K_3PO_4$ (3) | 45 |
| 12 | EtOH (10) | NaCl (1.0) | 4 | $K_3PO_4$ (3) | 77 |
| 13 | EtOH (10) | NaCl (1.0) | 7 | $K_3PO_4$ (3) | 80 |
| 14 | EtOH (10) | NaCl (1.0) | 8 | $K_3PO_4$ (3) | 58 |
| 15 | EtOH (10) | NaCl (1.0) | 12 | $K_3PO_4$ (3) | 40 |
| 16 | EtOH (10) | NaCl (1.0) | 7 | $Na_3PO_4$ (3) | 0 |
| 17 | EtOH (10) | NaCl (1.0) | 7 | CsF (3) | 0 |
| 18 | EtOH (10) | NaCl (1.0) | 7 | $K_2CO_3$ (3) | 0 |
| 19 | EtOH (10) | NaCl (1.0) | 7 | *t*-BuOK (3) | 20 |
| 20 | EtOH (10) | NaCl (1.0) | 7 | $K_3PO_4$ (4) | 82 |
| 21 | EtOH (10) | NaCl (1.0) | 7 | $K_3PO_4$ (5) | 77 |
| 22 | EtOH (10) | NaCl (1.0) | 7 | $K_3PO_4$ (6) | 11 |
| 23 | EtOH (10) | NaCl (1.0) | 7 | $K_3PO_4$ (7) | 35 |

[a] LAG = liquid assisted grinding agent. Weight% values were calculated against the sum of all reaction components, excluding the grinding axuliary. 10 weight% roughly translates to 0.122 μL/mg.

[b] GA = Grinding Auxiliary. Mass equivalents calculated from sum of all solid reaction components (i.e. excluding LAG agent).

[c] Yields determined by $^1$H NMR of the crude reaction mixture using mesitylene (23 μL, 0.167 mmol) as an internal standard.

[d] No nickel catalyst

[e] 3 g ball refers to a 9 mm stainless steel ball

[f] Isolated yield after silica gel chromatography

## 4. Temperature-controlled mechanochemistry set-ups

### 4.1. Heat gun set-up

1. Ball added to milling jar
2. Chemicals added

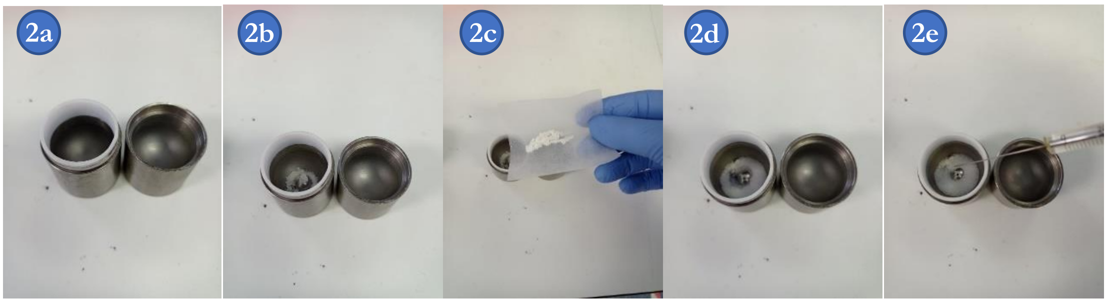


3. Jar closed
4. Placed on mixer mill

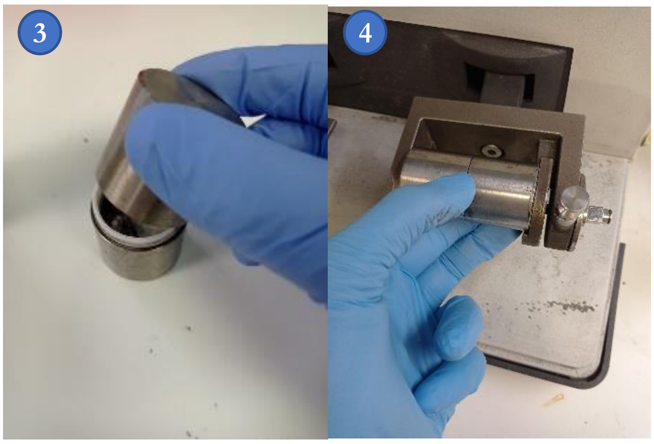


5. Heat gun set positioned and safety screen placed (Due to the removal of the devices protective cover)
6. Heat gun started
7. Grinding immediately initiated

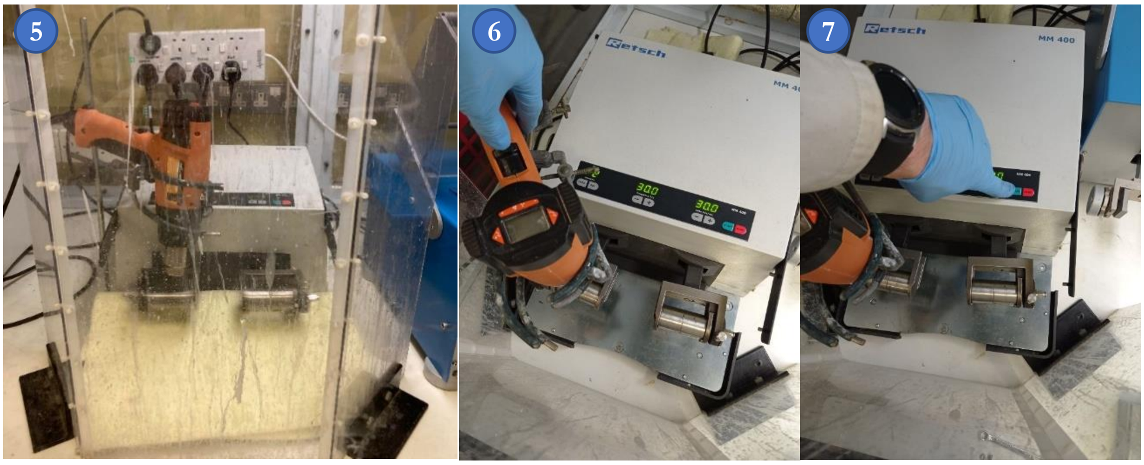

8. Reaction material removed using spatula and washed out using ≈ 30 mL EtOAc and 30 mL water and sonicated to break up larger particles (left; before and right; after)

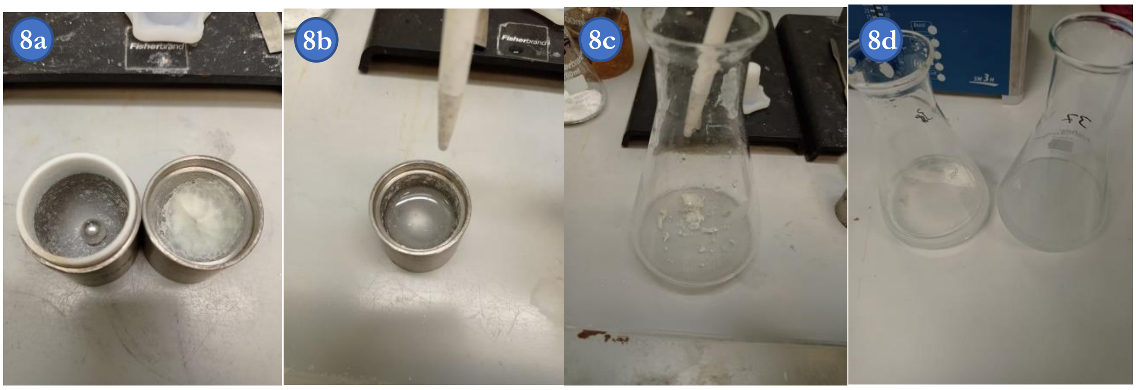


9. The organic phase (EtOAc top layer) was washer with 1.1M NaOH, Water and Brine and the solution was then dried over $MgSO_4$ and filtered

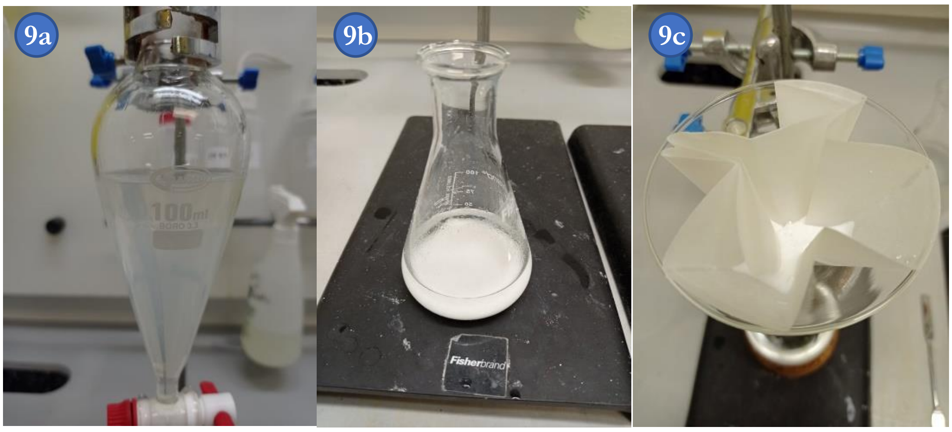


10. The solvent was then removed using a rotary evaporator to produce the crude product. Mesitylene was added as internal standard.

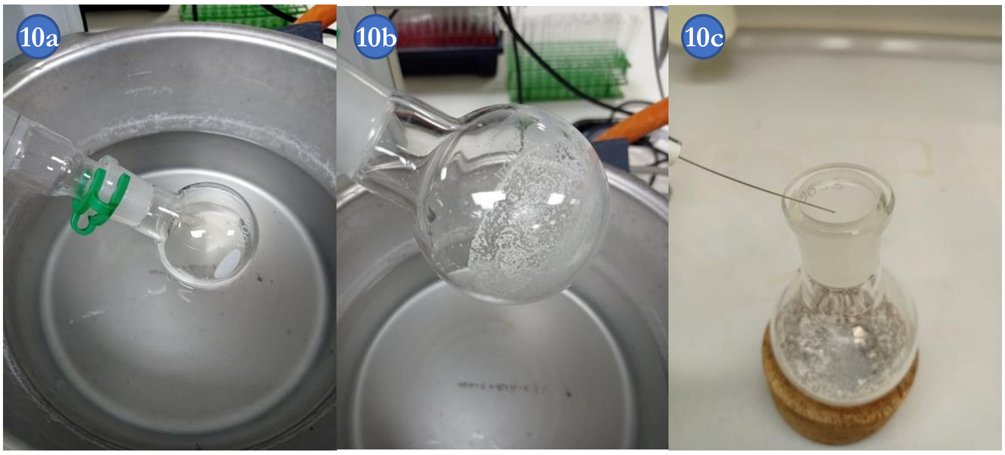

### 4.2. Heat device set-up

1. Steps 1-3 the same as 4.1
2. Band heater added over jar and tightened into position
3. Placed on mixer mill

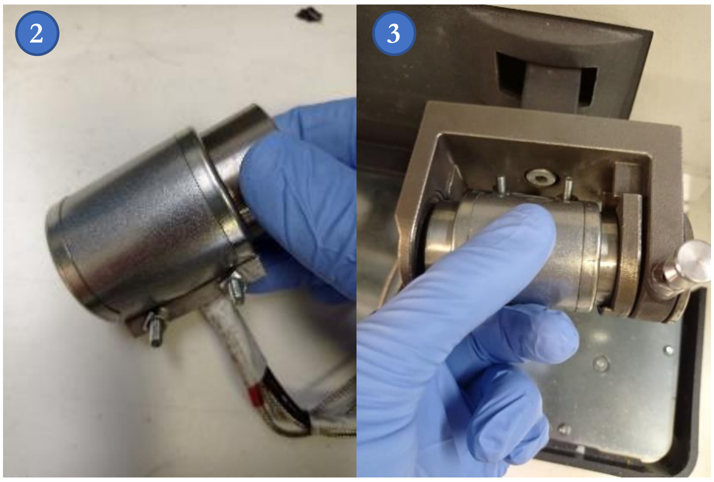


4. Cover closed (to turn off magnetic safety switch feature)

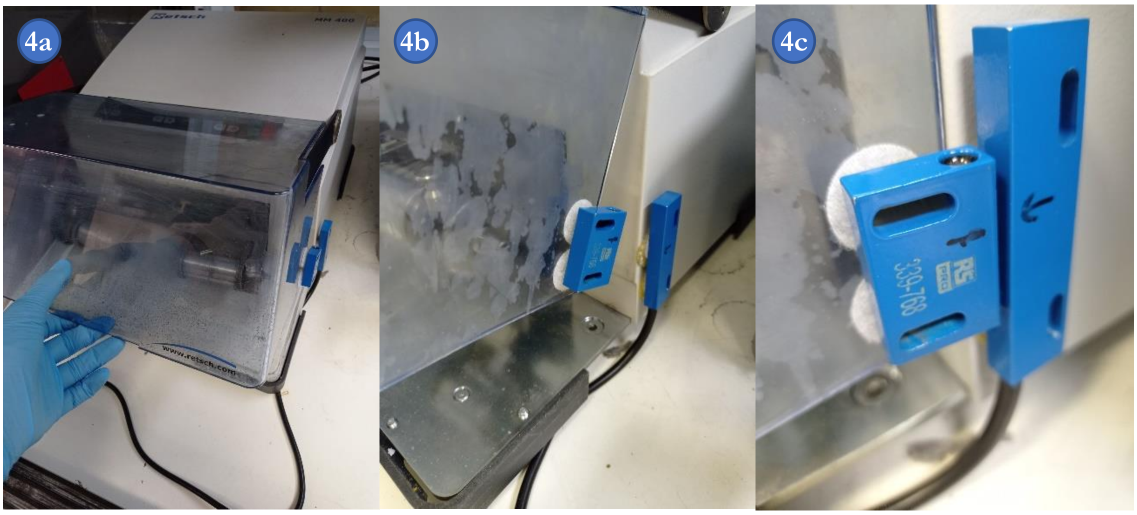


5. Control unit of band heater set to required temperature

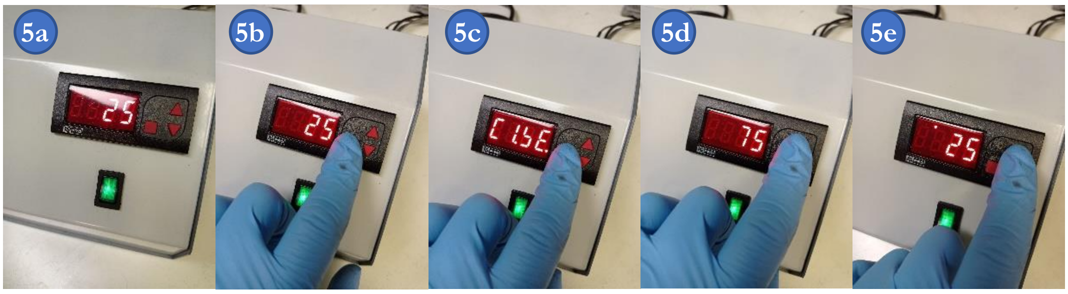


6. Milling commenced

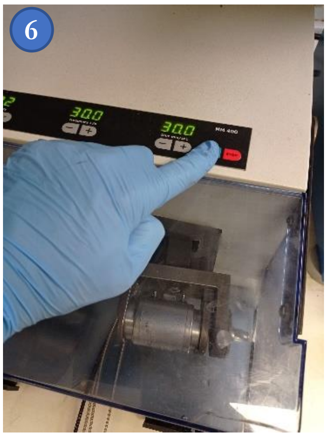


7. Steps 8-10 the same as 4.1

## 5. Jar heater details

### 5.1: Parts lists

**Table S2:** Parts list for PID-Controlled Unit

| Item | Supplier | Code | Quantity* |
|---|---|---|---|
| Mains lead | RS Components | 262-1126 | 1 |
| IEC Filter | RS Components | 123-0274 | 1 |
| Rocker Switch Red | RS Components | 419-782 | 1 |
| Rocker Switch Green | RS Components | 419-788 | 1 |
| PID controller | RS Components | 124-1059 | 2 |
| Magnetic Switch | CPC Farnell | PRM-SA-002 | 2 |
| Magnet | CPC Farnell | PRA-SA-003 | 2 |
| Thermocouple Plug J | RS Components | 455-9714 | 2 |
| Thermocouple Plug socket J | RS Components | 455-9691 | 2 |
| Housing unit | RS Components | 232-910 | 1 |
| C14 Cable mount IEC Male | RS Components | 776-9113 | 4 |
| C13 IEC outlet F | CPC Farnell | 4787.1 | 4 |

* Quantity required to make one heating unit excluding band heaters

Sourced from:

- RS Components – https://uk.rs-online.com/web/
- CPC Farnell - https://cpc.farnell.com/

**Table S3:** Parts list for band heaters

| Item | Supplier | Code | Quantity* |
|---|---|---|---|
| Mica Heater Band - 28mm Ø x 50mm 240w 240v, Type Low Cost with 500mm (3 core) Braided Leads + Type 'J' Thermocouple – **for 15 mL jar** | Gary Duncan | MHB39010 | 2 |
| Mica Heater Band - 35mm Ø x 50mm 300w 240v, Type Low Cost with 500mm (3 core) Braided Leads + Type 'J' Thermocouple – **for 30 mL jar** | Gary Duncan | MHB39011 | 2 |

* Quantity required to have two sets of interchangeable jar sizes

Sourced from:

- Gary Duncan Ltd - https://garyduncan.co.uk/

## 5.2: Images

**PID-Controlled Unit front view:** A – PID Controller, B – Rocker Switch

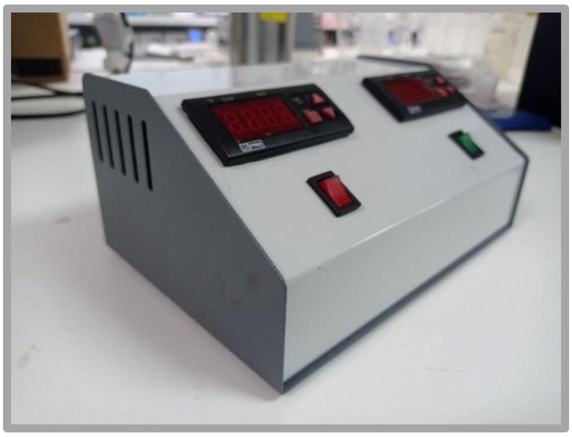

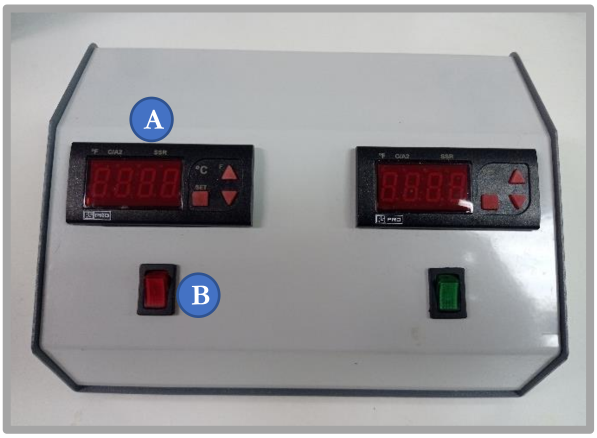


**PID-Controlled Unit back view:** C – IEC Filter, D – Thermocouple plug socket type J, E – Grounding Screw, F – C13 IEC outlet F

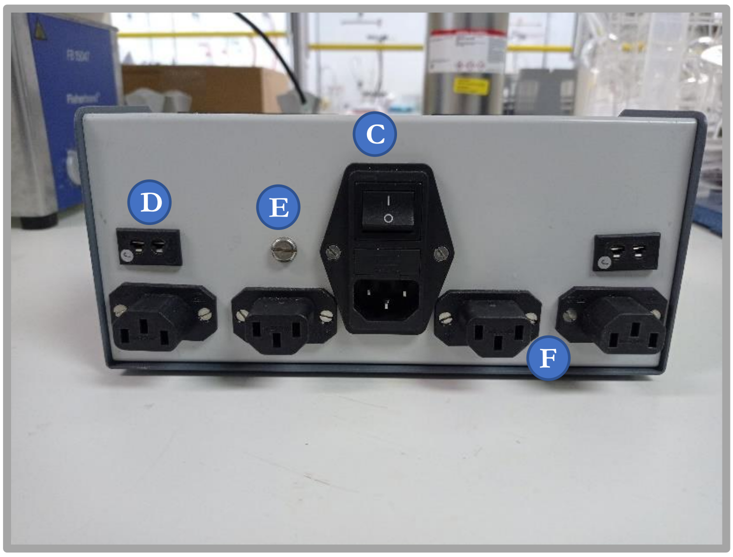


**Magnetic reed switch diagram:** A magnetic reed switch is a switch induced by the magnetic field upon it. This one is normally an open switch (NO) where the contacts are not conducted when no magnet is nearby, however upon the introduction of a magnet the contacts close completing the circuit, see schematic below.

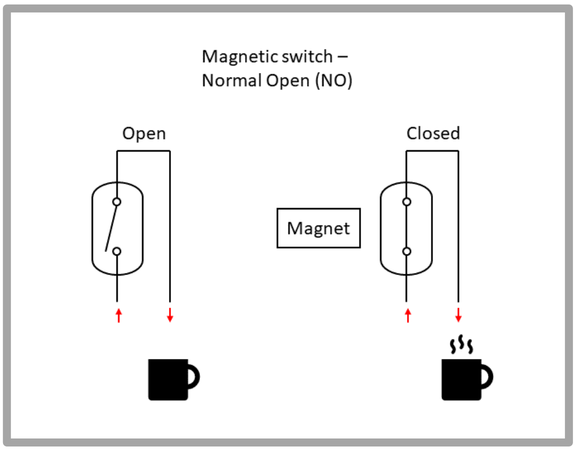

## 5.3: Internal wiring schematic

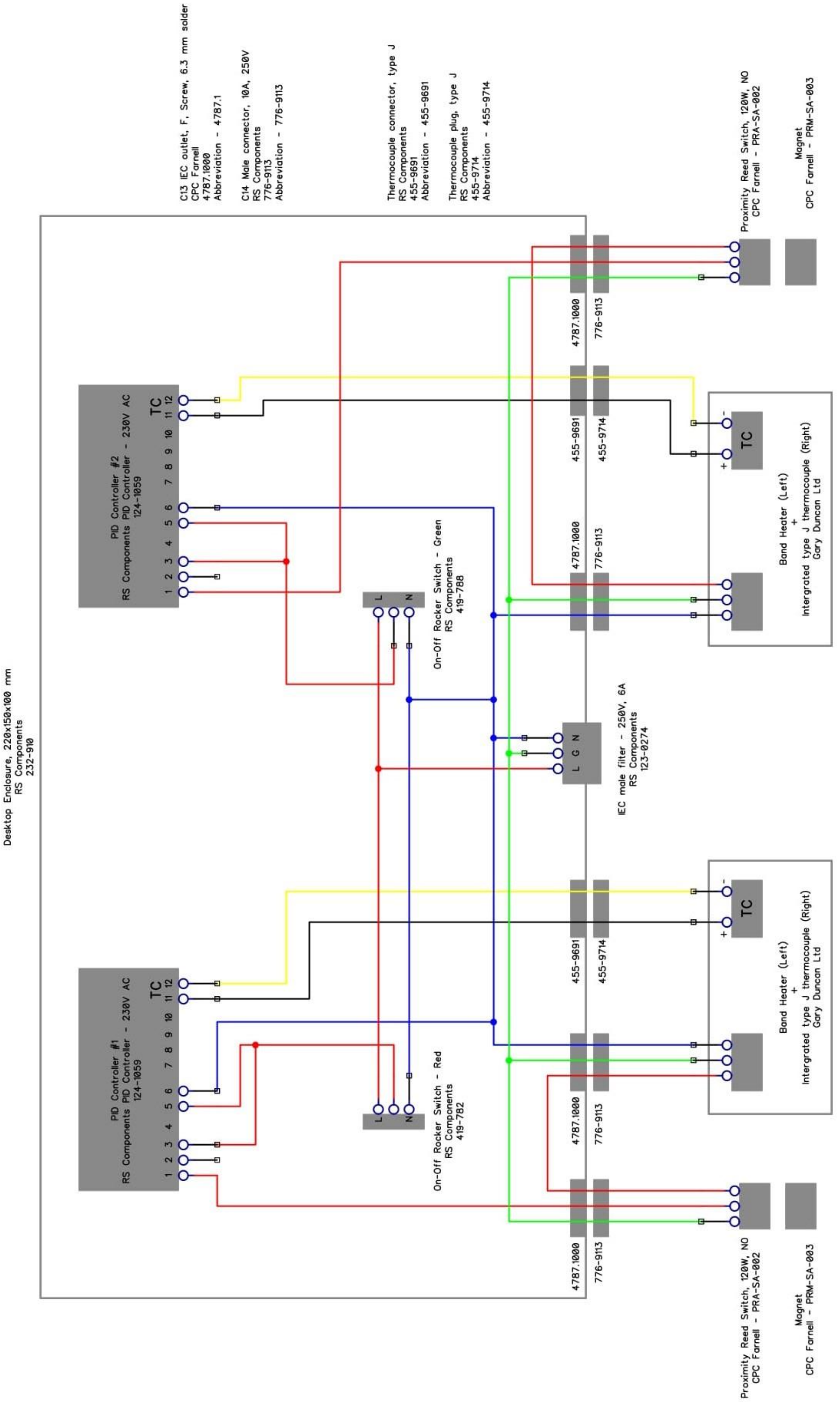

## 6. Monitoring reaction temperatures

The temperature of the milled reactions (both when ran at room temperature and at elevated temperatures were monitored over time using a **Lascar Electronics Data Logger** (lascarelectronics.com) attached to a **RS Pro Type K Thermocouple**. The thermocouple was attached to the jars using electrical tape and remained fastened throughout milling. Temperature values were obtained every 1 second over the course of a milling period. These temperature values were verified through use of an IR thermometer (**Testo 830-T1**) on the outside of the milling jar and also of an opened milling jar just after reaction finished.

**Heating profiles - 15 mL jar vs. 30 mL jar**. To the relevant stainless steel milling jar was added a 3 g stainless steel ball, 1-naphthyl dimethylsulfamate (0.5 mmol), 4-fluorophenyl boronic acid (0.75 mmol), $NiCl_2(PPh_3)_2$ (10 mol%), sodium chloride (2 mass equiv.), hexanol (0.122 µL/mg) and tripotassium phosphate (1.5 mmol). The jar was then milled at 30 Hz for 4 hours and the temperature monitored over time. Darker lines in graph below shows a 25 second moving average for clarity. Original values are shown in pastel colours.

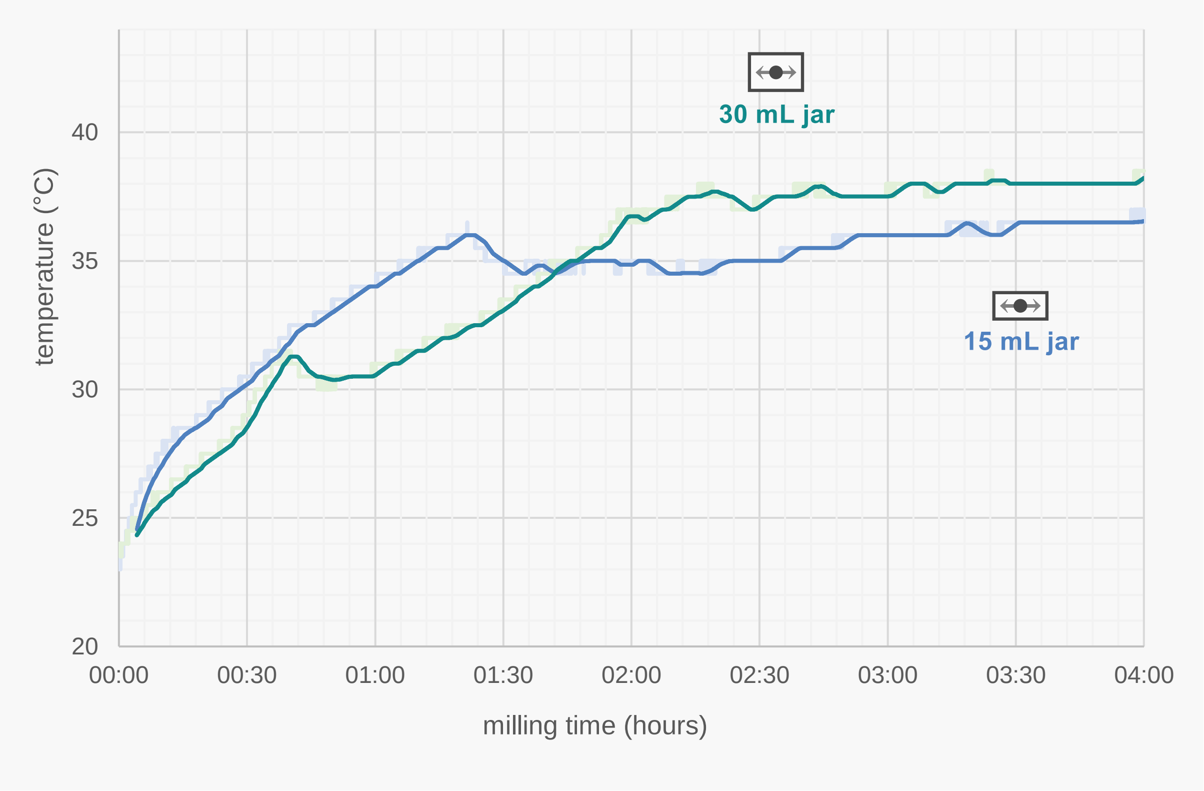


**Figure S1**: Temperature monitoring studies using different sized jars

**Heating profiles – Heat-Gun vs. Jar Heaters**. To the relevant stainless steel milling jar was added a 3 g stainless steel ball, 1-naphthyl dimethylsulfamate (0.5 mmol), 4-fluorophenyl boronic acid (0.75 mmol), $NiCl_2(PPh_3)_2$ (10 mol%), sodium chloride (2 mass equiv.), hexanol (0.122 µL/mg) and tripotassium phosphate (1.5 mmol). Using either the heat-gun set-up or the jar heater set-up, the jar was then milled at 30 Hz for 30 minutes and the temperature monitored over time. As the jar heater can overshoot – manual oversight of the temperature by stopping the temperature increase about 5 degrees below the target and then pressing heat again – can lead to a more accurate reaction profile (shown in blue). This latter process was used throughout the subsequent reaction development. Darker lines in the graph below shows a 25 second moving average for clarity. Original values are shown in pastel colours.

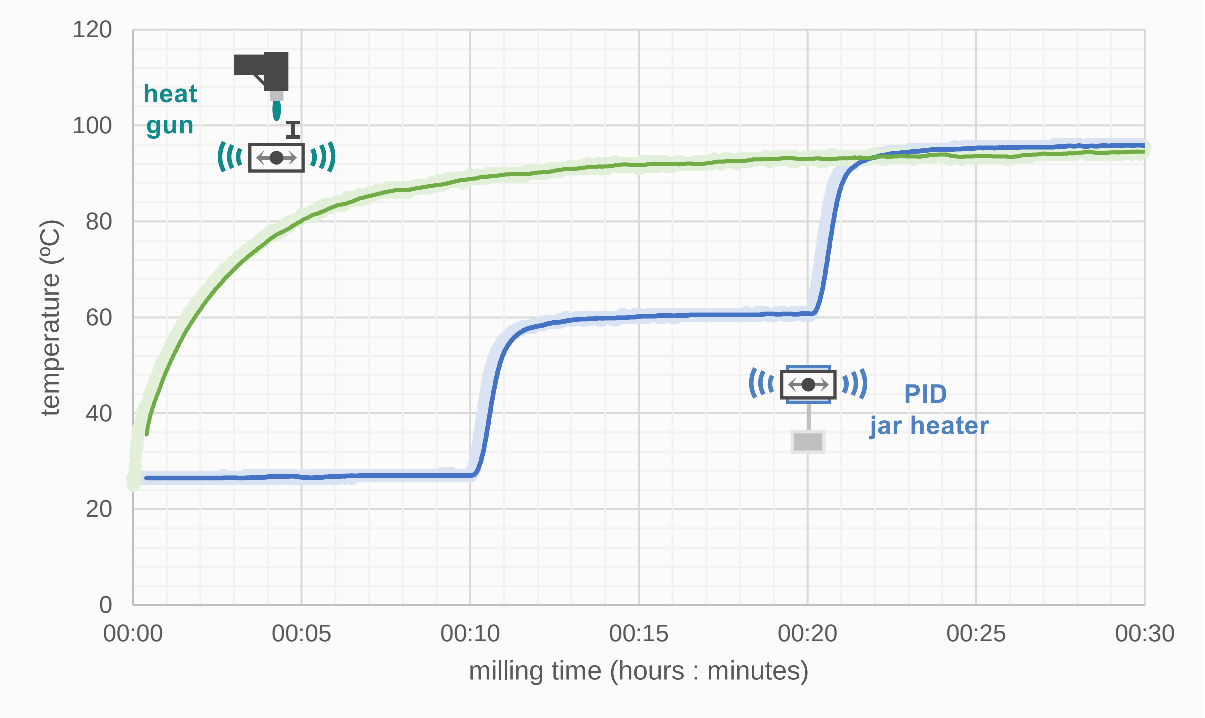


**Figure S2**: Temperature monitoring studies using different heating methods

## 7. Further experiments

**Table S4**: Reactions run in triplicate and quadruplicate to explore reproducibility

| Entry | Substrate | End Temp | Run 1 | Run 2 | Run 3 | Run 4 | Average Yield | SD |
|---|---|---|---|---|---|---|---|---|
| 1 | **3l** | 110 | 53 | 65 | 51 | N/A | 56 | 6 |
| 2 | **3c** | 120 | 63 | 62 | 69 | 68 | 66 | 3 |

**Table S5**: Varying jar size with a variety of ball sizes using the jar heater protocol and hexanol as LAG

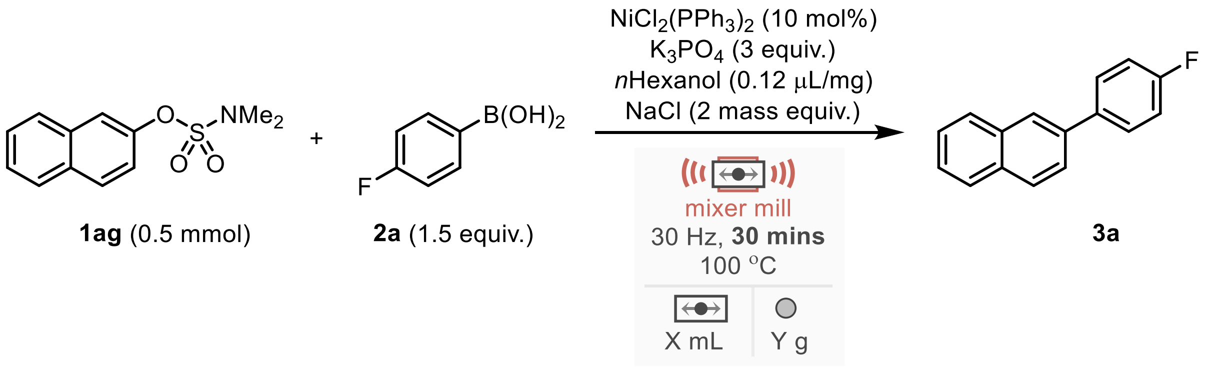


| Entry | Jar size (**X**) | Ball Size (**Y**) | NMR Yield |
|---|---|---|---|
| 1 | 30 mL | 3 g | 96% |
| 2 | 15 mL | 2 g | 87% |
| 3 | 15 mL | 1 g | 93% |

**Table S6**: Varying jar size with a variety of ball sizes using the room temperature protocol and hexanol as LAG

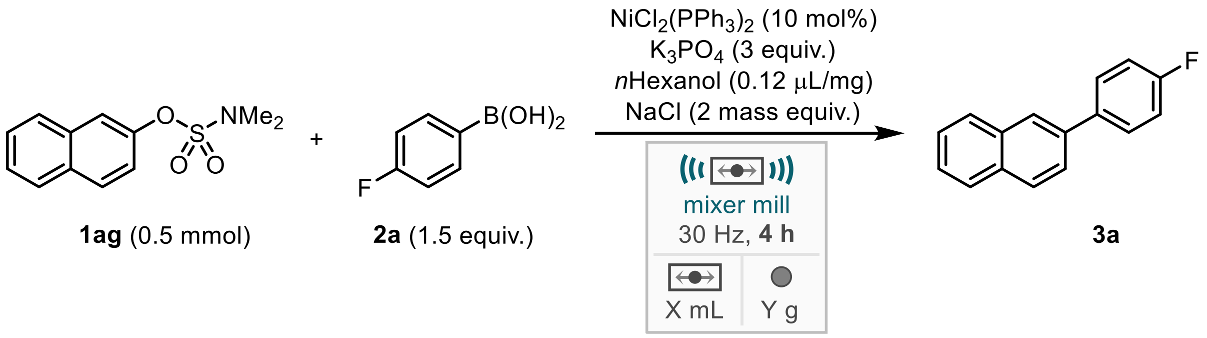


| Entry | Jar size (**X**) | Ball Size (**Y**) | NMR Yield |
|---|---|---|---|
| 1 | 30 mL | 3 g | 85% |
| 2 | 15 mL | 2 g | 54% |
| 3 | 15 mL | 1 g | 55% |

30 ml → 15 ml jar is 0.5, 3 g ball to 1.5 g ball however we only have 1 g and 2 g milling balls so both were used for comparison

**Table S5**: Varying jar size with a variety of ball sizes using the jar heater protocol and hexanol as LAG

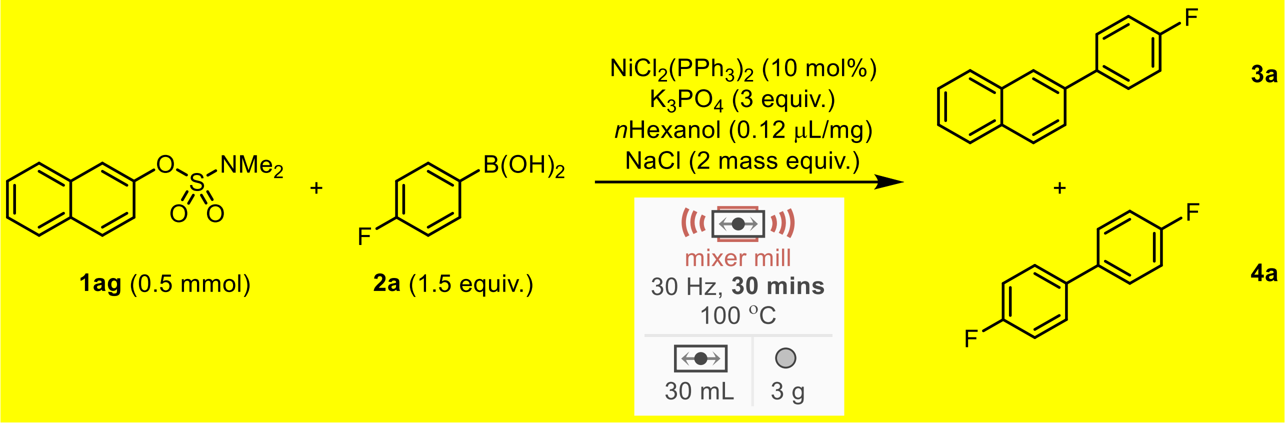


| Entry | 10 min (RT) | 10 min (63 °C) | 10 min (100 °C) | Average NMR Yield **3a** | Biphenyl yield **4a** |
|---|---|---|---|---|---|
| 1 | X | | | 8% | 2% |
| 2 | X | X | | 69% | 4% |
| 3 | X | X | X | 96% | 6% |

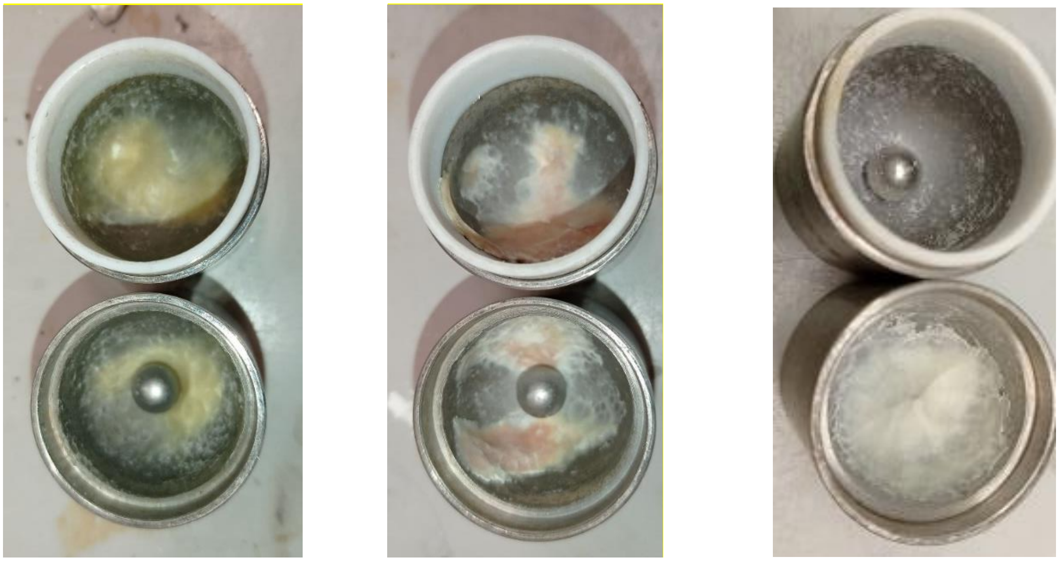

Images: 10 minutes of milling (Left), 20 minutes of milling (Centre), 30 minutes of milling (Right)

## 8. Variable temperature experiments using HP2

Starting with electron rich 4-methoxyphenyl boronic acid (**2c**), a steady increase was observed culminating in highest yields at 130 °C. It should be noted that (along with **2b** and **2d**) no reaction occurred without the addition of heat, at either 30 mins or 4-hour reaction time. The well-studied electron neutral 4-fluorophenyl boronic acid (**2a**) showed excellent reactivity at all studied temperatures with those between 90 °C and 120 °C resulting in the greatest yields. Interestingly in this case, further heating to 130 °C resulted in a decreased yield of 85%. Proceeding to electron poor boronic acids bearing $CO_2Et$ (**2d**) and $CF_3$ (**2e**) similar good reactivity was observed throughout the temperature range, with optimal points at 120 °C and 100 °C respectively.

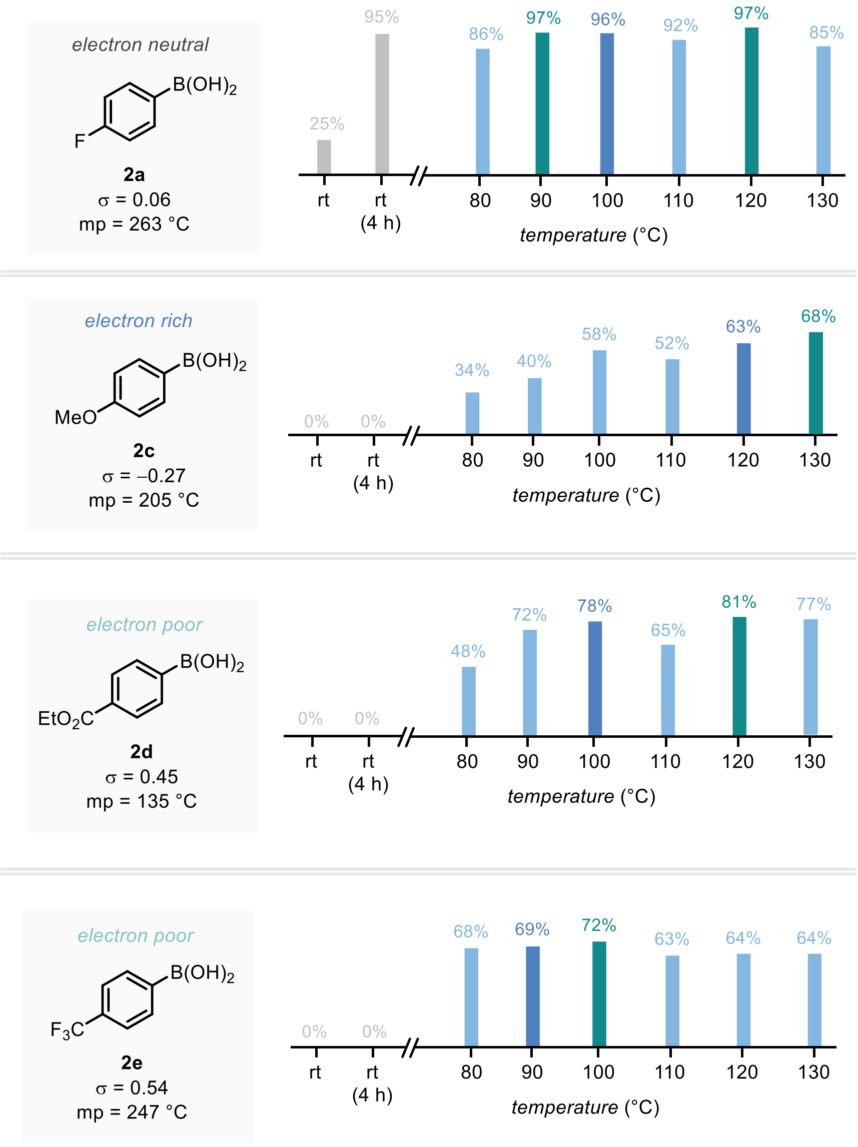


**Scheme S1:** Temperature variations using a variety of electronically-diverse boronic acid species including control experiments at room temperature

The consistent drop in yield at 110 °C across this study is suggested to be linked to a phase change in the sulfamate starting material which has a melting point of. 71-74 °C. The use of 110 °C goes through a mid point temperature of 68 °C (for 10 minutes during HP2) whereas the alternative temperatures (higher than this) result in a mid point above or below this. To assess this finding the 110 °C reaction was conducted again for **1ag** and **2d** with a mid point of 73 °C. In so doing, the yield moderately increased from 65% to 75% demonstrating the importance of the phase change.

## 9. Sustainability Metrics

### 9.1: Process Mass Intensity (PMI) calculations

$$PMI_{reaction} = \frac{Total\ mass\ of\ step}{Mass\ of\ product}$$

### Milled reactions

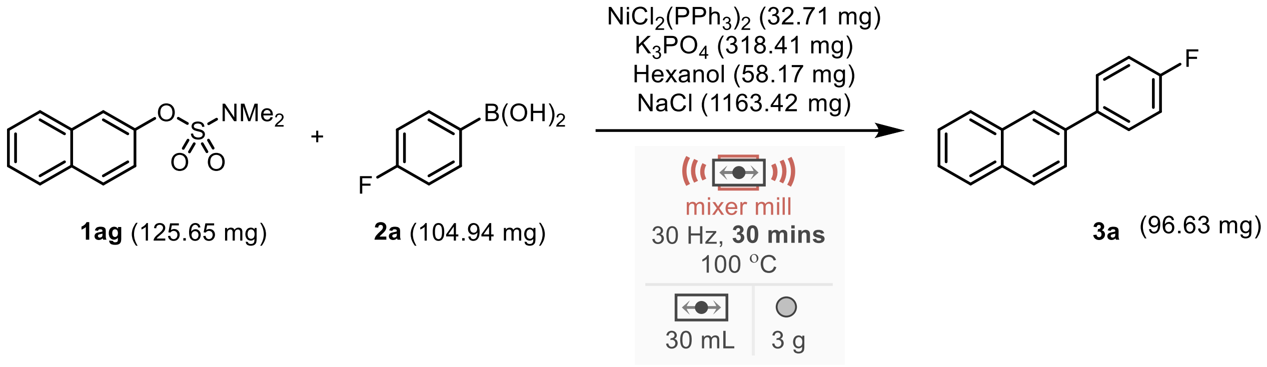


$$PMI_{reaction} = \frac{125.65 + 104.94 + 32.71 + 318.41 + 58.17 + 1163.42}{96.63} = 18.66$$

### Extruded reactions

50 mmol

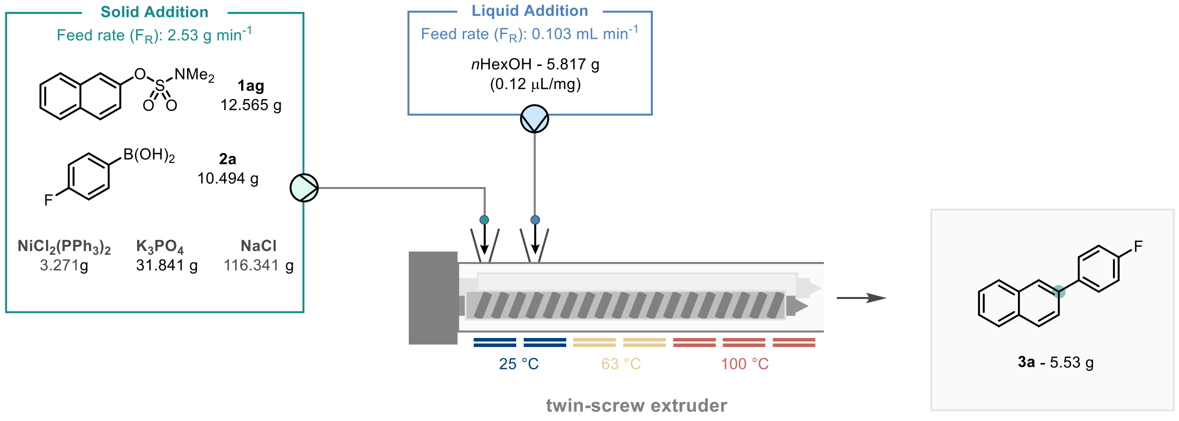


$$PMI_{reaction} = \frac{12.565 + 10.494 + 3.271 + 31.841 + 116.341 + 5.815}{5.53} = 32.60969851$$

100 mmol

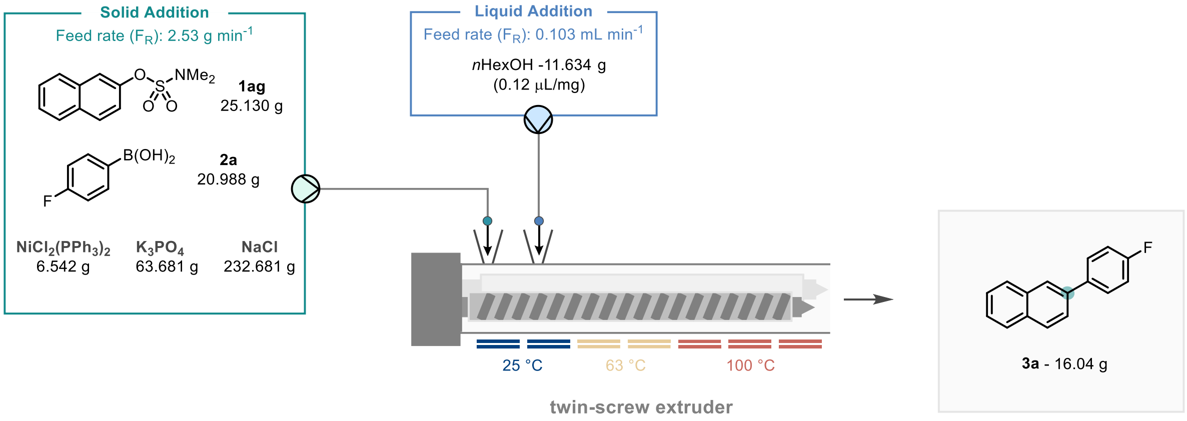


$$PMI_{reaction} = \frac{25.130 + 20.988 + 6.542 + 63.681 + 11.634 + 232.681}{16.04} = 21.760$$

## 9.2: E-factor calculations

$$E\ factor = \frac{mass\ of\ input - mass\ of\ product}{mass\ of\ product}$$

### Milled reaction

$$E\ factor = \frac{(125.65 + 104.94 + 32.71 + 318.41 + 58.17 + 1163.42) - 96.63}{96.63} = 17.66191659$$

### Extruded reactions

50 mmol

$$E\ factor = \frac{(12.565 + 10.494 + 3.271 + 31.841 + 116.341 + 5.815) - 5.53}{5.53} = 31.6096985$$

100 mmol

$$E\ factor = \frac{(25.13 + 20.99 + 6.54 + 63.68 + 11.634 + 232.68) - 16.04}{16.04} = 21.49$$

## 9.3: Space time yield (STY) calculations

$$STY\ (kg\ days^{-1}m^{-3}) = \frac{mass\ of\ product\ (kg)}{duration\ of\ reaction\ (days) \times volume\ of\ reactor(m^3)}$$

50 mmol extrusion

$$STY_{overall} = \frac{0.00553}{0.048611111 \times 0.00003542} = 3211.74\ kg\ days^{-1}m^{-3}$$

100 mmol extrusion

$$STY_{steady\ state} = \frac{0.01067}{0.0625 \times 0.00003542} = 4819.87\ kg\ days^{-1}m^{-3}$$

$$STY_{overall} = \frac{0.01604}{0.125 \times 0.00003542} = 3622.81\ kg\ days^{-1}m^{-3}$$

## 10: Extrusion protocols

The large-scale extrusion protocols were carried out using a Thermo Scientific™ Process 11 Twin-screw Extruder (TSE) with 7 controllable heating sections.

### General Extrusion Procedure

To a large beaker was added 2-naphthalene sulfamate (1 equiv.), 4-fluorophenyl boronic acid (1.5 equiv.), $NiCl_2(PPh_3)_2$ (10 mol%), sodium chloride (2 mass equiv.) and $K_3PO_4$ (3 equivs.). The mixture was mixed by hand using a spatula. The pre-mixed mixture was added to the volumetric hopper situated at the first port (**Main Feed**, Figure S2) with a feed rate of 2.52 $gmin^{-1}$ – calibrated *ex situ*. Hexanol (0.1 volume equiv.) was added via syringe pump through the second port (**Liquid feeding**) at a rate of 0.103 mL $min^{-1}$

The TSE was set at 50 rpm and each of the twin-screw extruder seven heating zones were heated to the correct temperature (2 x 25 °C, 2 x 63 °C, 3 x 100 °C).

The screw configuration was arranged as shown below in Figure S2.

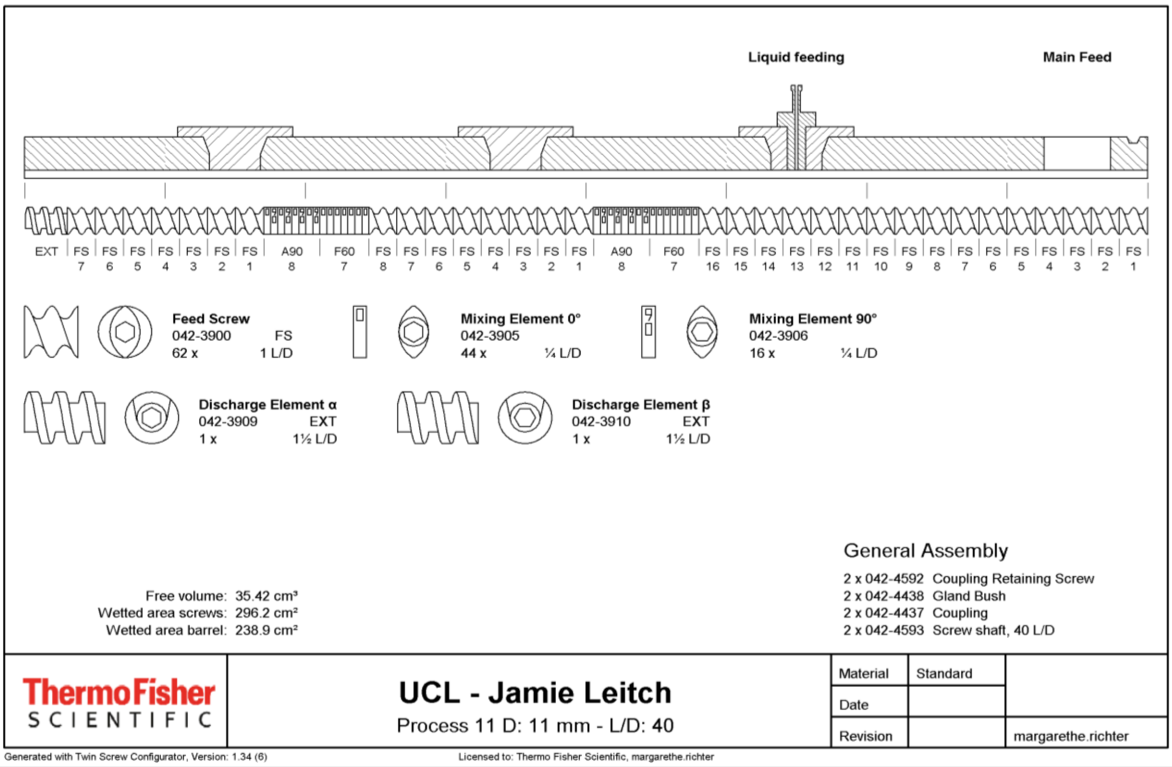


**Figure S2**: Screw configuration utilised in this process, provided by Thermo Fisher Scientific

The reaction mixture came out of the twin-screw extruder and was collected in a beaker filled with water (≈40 mL) at 10-minute intervals. Each beaker was added to a separating funnel and ethyl acetate added. The reaction mixture was then washed with 1.1 M NaOH, water and brine, dried over magnesium sulfate and concentrated *in vacuo* to give the crude reaction mixture.

The resulting crude residue was purified by silica gel flash column chromatography using hexane to give the pure product unless stated otherwise.

## 50 mmol reaction

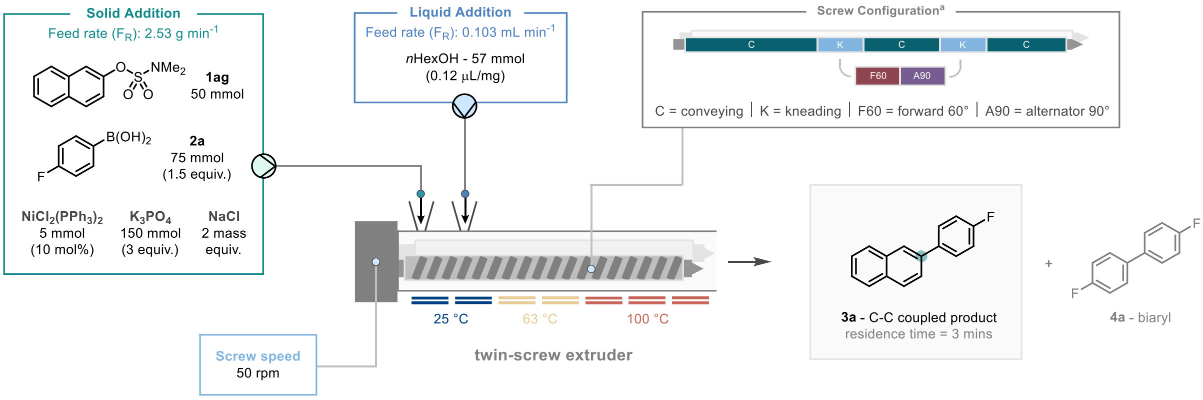


2-napthalene sulfamate (12.565 g, 50 mmol, 1 equiv.), 4-fluorophenyl boronic acid (10.494 g, 75 mmol, 1.5 equiv.), $NiCl_2(PPh_3)_2$ (3.271 g, 2.5 mmol, 10 mol%), $K_3PO_4$ (31.841 g, 150 mmol, 3 equiv.) and sodium chloride (116.341 g, 2 mass equiv.) were added to the large beaker and the mixture was mixed by hand using a spatula and added to a volumetric single screw feeder.

Hexanol (7.146 mL, 57 mmol, 0.1 volume equiv.) was added via syringe pump through the second port at a rate of 0.103 mL $min^{-1}$

**Table S7:** Results from the 50 mmol extrusion protocol. Yields given are NMR yields calculated vs. mmol $min^{-1}$ starting material input against mesitylene as an internal standard

| Time (mins) | Product Yield | Starting material yield | Biphenyl yield | Conversion (P+SM+B) |
|---|---|---|---|---|
| 0 | 0% | 0% | 0% | 0% |
| 10 | 23% | 20% | 2% | 45% |
| 20 | 52% | 1% | 6% | 59% |
| 30 | 61% | 0% | 5% | 66% |
| 40 | 62% | 0% | 5% | 67% |
| 50 | 59% | 0% | 6% | 65% |
| 60 | 73% | 10% | 6% | 89% |
| 70 | 15% | 9% | 7% | 31% |

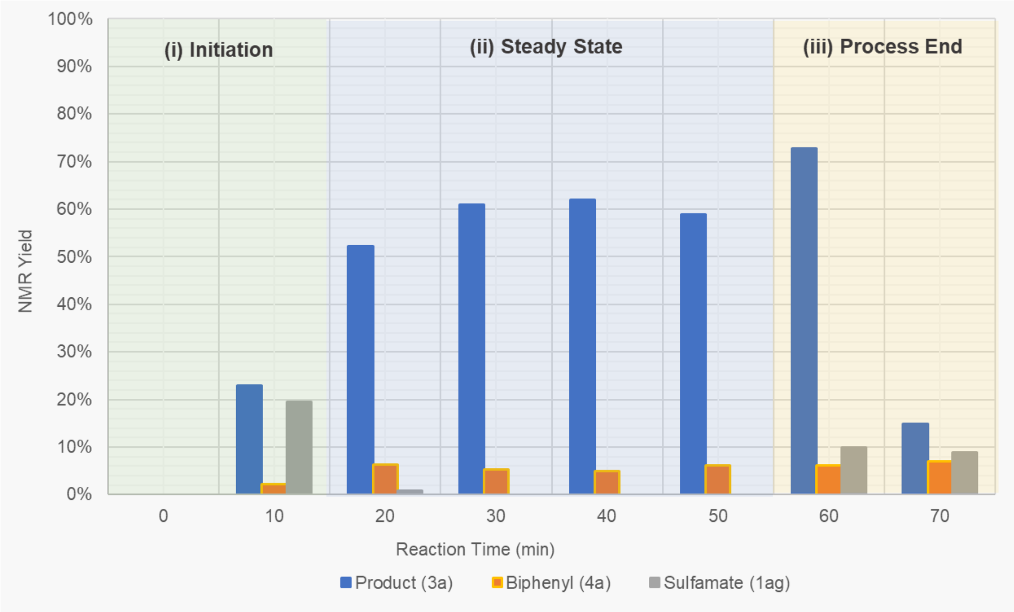


**Figure S3.** Results from 50 mmol reaction

## 100 mmol reaction

2-napthalene sulfamate (25.130 g, 100 mmol, 1 equiv.), 4-fluorophenyl boronic acid (20.988 g, 150 mmol, 1.5 equiv.), $NiCl_2(PPh_3)_2$ (6.542 g, 5 mmol, 10 mol%), $K_3PO_4$ (63.681 g, 300 mmol, 3 equiv.) and sodium chloride (232.681 g, 2 mass equiv.) were added to the large beaker and the mixture was mixed by hand using a spatula and added to a volumetric single screw feeder.*

Hexanol (14.3 mL, 114 mmol, 0.1 volume equiv.) was added via syringe pump through the second port at a rate of 0.103 mL $min^{-1}$.

Material started appearing at the end of the extruder after ~3 minutes and torque values between 1.8 and 3.2 Nm were maintained throughout the process.

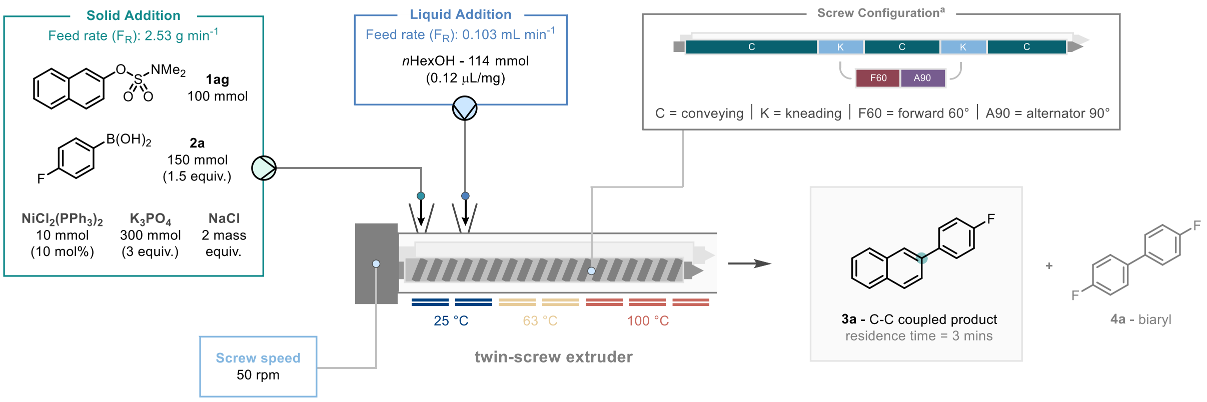


*Sodium chloride and $K_3PO_4$ were both dried in 175 °C oven for approximately 3 hours prior to use

**Table S8:** Results from the 100 mmol extrusion protocol. Yields given are NMR yields calculated vs. mmol $min^{-1}$ starting material input against mesitylene as an internal standard

| Time (mins) | Product Yield | Starting material yield | Biphenyl yield | Conversion (P+SM+B) |
|---|---|---|---|---|
| 0 | 0% | 0% | 0% | 0% |
| 10 | 20% | 20% | 1% | 41% |
| 20 | 46% | 1% | 3% | 51% |
| 30 | 42% | 1% | 2% | 45% |
| 40 | 46% | 0% | 3% | 49% |
| 50 | 49% | 0% | 3% | 52% |
| 60 | 71% | 3% | 5% | 79% |
| 70 | 60% | 0% | 4% | 63% |
| 80 | 74% | 3% | 4% | 81% |
| 90 | 71% | 0% | 4% | 76% |
| 100 | 75% | 5% | 5% | 84% |
| 110 | 63% | 1% | 5% | 69% |
| 120 | 89% | 1% | 4% | 95% |
| 130 | 81% | 1% | 4% | 87% |
| 140 | 81% | 1% | 4% | 86% |
| 150 | 52% | 10% | 4% | 66% |
| 160 | 37% | 19% | 3% | 58% |
| 170 | 36% | 2% | 3% | 40% |
| 180 | 8% | 2% | 1% | 10% |

*150g of NaCl was added at 140 minutes and the screw speed was increased from 50 rpm to 100 rpm at 150 minutes as torque values of over 4.0 Nm were recorded.

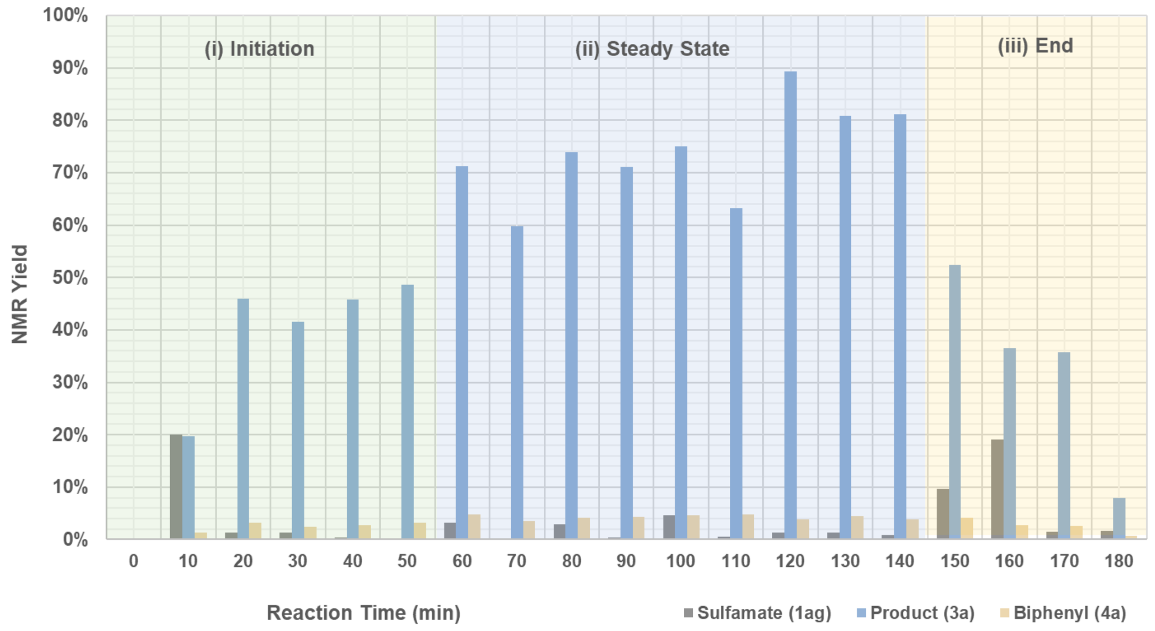


**Figure S4.** Results from 100 mmol reaction

Fractions from (ii) Steady state were combined and purified *via* silica gel column chromatography to give pure product **3a**. (i) Initation and (ii) End were also combined together and purified *via* silica gel column chromatography. The two pots of material were combined together to give the overall isolated yield, 13.76 g (62%).

## 11. Experimental procedures

## 11.1 Synthesis of starting materials

### 11.1.1. Procedures for other activated phenol starting materials

**2-Naphthyl pivalate (1aa)**

Prepared according to modified literature.[1] To an oven dried round bottom flask equipped with a stirring bar, alcohol (1 equiv.), $NEt_3$ (1.1 equiv.), DMAP (10 mol%) and DCM was added. Pivaloyl chloride (1.1 equiv.) was added dropwise, and the reaction stirred until effervescence subsided.

The reaction was washed with $NaHSO_4$ and the aqueous layer extracted with DCM (3 x 25 mL). The combined organic layers were washed with brine (3 x 25 mL) and dried over magnesium sulfate. The solvent was evaporated to give the crude product.

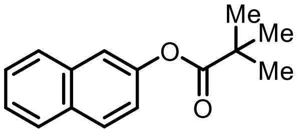


The crude product was purified by flash column chromatography to afford **2-Naphthyl pivalate** (1.10 g, 98%) as a white solid. **mp**: 66–68 °C. **$^1$H NMR** (500 MHz, $CDCl_3$) δ 7.89 – 7.82 (m, 2H), 7.82 – 7.77 (m, 1H), 7.56 – 7.52 (d, *J* = 2.3 Hz, 1H), 7.52 – 7.43 (m, 2H), 7.23 – 7.17 (dd, *J* = 8.8, 2.3 Hz, 1H), 1.43 – 1.39 (s, 9H) ppm. **$^{13}$C NMR** (126 MHz, $CDCl_3$) δ 177.4, 148.9, 133.9, 131.5, 129.5, 127.9, 127.7, 126.6, 125.7, 121.3, 118.5, 39.3, 27.3 ppm.

This data is consistent with the literature.[1]

***tert*-Butyl 2-naphthyl carbonate (1ab)**

Prepared according to modified literature.[2] To an oven dried round bottom flask equipped with a stirring bar, alcohol (1 equiv.), $NEt_3$ (1.1 equiv.), DMAP (10 mol%) and DCM were added. $Boc_2O$ (1.1 equiv.) was added dropwise, and the reaction stirred until effervescence subsided.

The reaction was washed with $NaHSO_4$ and the aqueous layer extracted with DCM (3 x 25 mL). The combined organic layers were washed with brine (3 x 25 mL) and dried over magnesium sulfate. The solvent was evaporated to give the crude product.

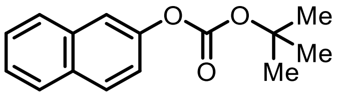


The crude product was purified by flash column chromatography to afford ***tert*-Butyl 2-naphthyl carbonate** (1.06 g, 87%) as a white solid. **mp**: 73–75 °C. **$^1$H NMR** (500 MHz, $CDCl_3$) δ 7.90 – 7.78 (ddd, *J* = 13.5, 8.6, 6.4 Hz 3H), 7.68 – 7.62 (d, *J* = 2.5 Hz, 1H), 7.53 – 7.42 (m, 2H), 7.35 – 7.29 (dt, *J* = 8.8, 1.8 Hz, 1H), 1.62 – 1.57 (s, 9H) ppm. **$^{13}$C NMR** (126 MHz, $CDCl_3$) δ 152.1, 148.8, 133.8, 131.5, 129.5, 127.9, 127.8, 126.7, 125.8, 121.0, 118.3, 83.8, 27.9 ppm.

This data is consistent with the literature.[2]

**2-Naphthyl diethylcarbamate (1ac)**

Prepared according to modified literature.[2] To an oven dried round bottom flask equipped with a stirring bar, sodium hydride (60% parrafin oil) was added under argon. The flask was cooled to 0 °C and the alcohol (1 equiv.) in 1 M dry THF was added over 10 minutes. The reaction mixture was then stirred at rt. For 10 minutes. The reaction mixture was then cooled to 0 °C and Diethyl carbonyl chloride (1.2 equiv.) was added over 10 minutes. The reaction was left to stir overnight.

The reaction was quenched with water. Ether was added and the solution washed with 1.1 M NaOH (25 mL) and water (3 x 25 mL). The combined aqueous layers were then extracted with ether. The combined organic layers were washed with brine (3 x 25 mL) and dried over magnesium sulfate. The solvent was evaporated to give the crude product.

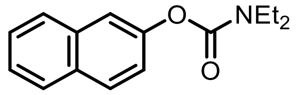


The crude product was purified by flash column chromatography to afford **2-Naphthyl diethylcarbamate** (2.16 g, 90%) as a pale-yellow oil. **$^1$H NMR** (500 MHz, $CDCl_3$) δ 7.87 – 7.81 (m, 2H), 7.81 – 7.77 (dd, *J* = 8.1, 1.4 Hz, 1H), 7.61 – 7.57 (d, *J* = 2.3 Hz, 1H), 7.50 – 7.41 (dddd, *J* = 16.6, 8.2, 6.8, 1.4 Hz, 2H), 7.31 – 7.27 (dd, J = 8.8, 2.4 Hz, 1H), 3.57 - 3.37 (m, 4H), 1.35 – 1.20 (m, 6H) ppm. **$^{13}$C NMR** (126 MHz, $CDCl_3$) δ 154.5, 149.3, 134.0, 131.3, 129.2, 127.8, 127.7, 126.4, 125.4, 121.8, 118.5, 42.4, 42.1, 14.4, 13.6 ppm.

This data is consistent with the literature. [2]

**2-Naphthyl triflate (1ad)**

Prepared according to modified literature.[3] To round bottom flask equipped with a stirring bar, alcohol (1 equiv.), pyridine (1.2 equiv.) and dry DCM were added. The flask was cooled to 0 °C and $Tf_2O$ (1.5 equiv.) was added dropwise. The reaction was warmed to RT and left to stir overnight.

The reaction was saturated with $NaHCO_3$ (25 mL). The aqueous phase was washed with DCM (3 x 25 mL) and dried over magnesium sulfate. The solvent was evaporated to give the crude product.

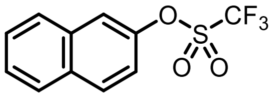


The crude product was purified by flash column chromatography to afford **2-Naphthyl triflate** (1.24 g, 90%) as a white solid. **mp**: 31–33 °C. **$^1$H NMR** (500 MHz, $CDCl_3$) δ 7.97 – 7.85 (m, 3H), 7.79 – 7.74 (d, *J* = 2.5 Hz, 1H), 7.63 – 7.55 (td, *J* = 6.7, 3.7 Hz, 2H), 7.42 – 7.36 (dd, *J* = 9.0, 2.5 Hz, 1H) ppm. **$^{13}$C NMR** (126 MHz, $CDCl_3$) δ 147.2, 133.5, 132.5, 130.8, 128.2, 128.1, 127.7, 127.3, 119.7, 119.4, 119.0 (q, *J* = 321 Hz) ppm.

This data is consistent with the literature. [4]

**2-Naphthyl tosylate (1ae)**

Prepared according to modified literature.[5] To round bottom flask equipped with a stirring bar, alcohol (1 equiv.), $NEt_3$ (1.5 equiv.), DMAP (20 mol%) and DCM were added. The reaction was cooled to 0 °C and TsCl (1.2 equiv.) in DCM was added dropwise and left to stir for 2 hours.

The reaction was quenched with DCM (3 x 25 mL) and the combined organic layers washed with brine (3 x 25 mL) and dried over magnesium sulfate. The solvent was evaporated to give the crude product.

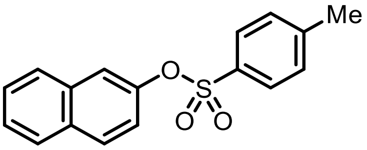

The crude product was purified by flash column chromatography to afford **2-Naphthyl tosylate** (1.28 g, 86%) as a white solid. **mp**: 125–127 °C. **$^1$H NMR** (500 MHz, $CDCl_3$) δ 7.85 – 7.78 (m, 1H), 7.78 – 7.71 (m, 4H), 7.54 – 7.45 (m, 3H), 7.33 – 7.27 (d, *J* = 8.1, 1H), 7.12 – 7.07 (dd, *J* = 8.9, 2.4 Hz, 1H), 2.47 – 2.42 (s, 3H) ppm. **$^{13}$C NMR** (126 MHz, $CDCl_3$) δ 147.3, 145.5, 133.5, 132.5, 132.0, 129.9, 129.9, 128.7, 128.0, 127.9, 127.0, 126.5, 121.3, 120.1, 21.9 ppm.

This data is consistent with the literature.[6]

**2-Naphthyl mesylate (1af)**

Prepared according to modified literature.[7] To an oven dried round bottom flask equipped with a stirring bar, alcohol (1 equiv.), dry pyridine (5 equiv.) and dry DCM were added, and the flask was cooled to 0 °C. Methane sulfonyl chloride (1.2 equiv.) was added dropwise. The reaction was warmed to RT and left to stir overnight.

The reaction was quenched with water. The aqueous layer was washed with DCM (3 x 25 mL). The combined organic layers were washed with 15% HCl (25 mL), brine (3 x 25 mL) and then dried over magnesium sulfate. The solvent was evaporated to give the crude product.

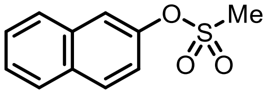

The crude product was purified by recrystallisation (Hexane: DCM) to afford **2-Naphthyl mesylate** (506 mg, 45%) as a pink solid. **mp**: 107–109 °C. **$^1$H NMR** (500 MHz, $CDCl_3$) δ 7.94 – 7.83 (m, 3H), 7.79 – 7.75 (d, *J* = 2.4 Hz, 1H), 7.58 – 7.50 (m, 2H), 7.44 – 7.39 (dd, *J* = 8.9, 2.4 Hz, 1H), 3.21 – 3.18 (s, 3H) ppm. **$^{13}$C NMR** (126 MHz, $CDCl_3$) δ 146.9, 133.7, 132.2, 130.5, 128.0, 128.0, 127.3, 126.8, 120.9, 119.6, 37.6 ppm.

This data is consistent with the literature.[6]

### 11.1.2. General Procedure A: Synthesis of aryl sulfamates

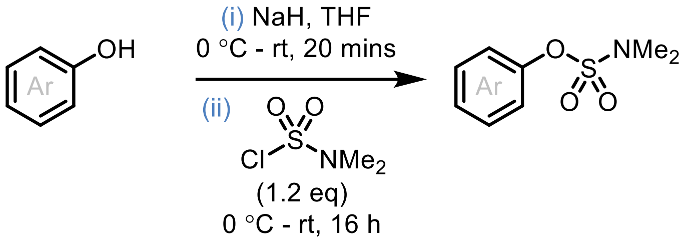


Prepared according to modified literature (Reference).[8] To an oven dried round bottom flask equipped with a stirring bar, sodium hydride (60% parrafin oil) was added under argon. The flask was cooled to 0 °C and the alcohol (1 equiv.) in 1 M dry THF was added over 10 minutes. The reaction mixture was then stirred at rt. For 10 minutes. The reaction mixture was then cooled to 0 °C and *N,N*-Dimethylsulfamoyl chloride (1.2 equiv.) was added over 10 minutes. The reaction was left to stir overnight.

The reaction was quenched with water. Ether was added and the solution washed with 1.1 M NaOH (25 mL) and water (3 x 25 mL). The combined aqueous layers were then extracted with ether. The combined organic layers were washed with brine (3 x 25 mL) and dried over magnesium sulfate. The solvent was evaporated to give the crude product.

This was then purified by flash column chromatography to give the pure product.

**2-Naphthyl dimethylsulfamate (1ag)**

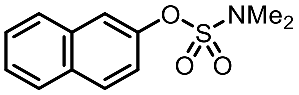


Prepared according to general procedure **A**. Purified by flash column chromatography (Hexane: DCM, 1:1) to give **2-Naphthyl dimethylsulfamate** (2.23 g, 89%) as a pale-yellow solid. **mp**: 71–74 °C **$^1$H NMR** (500 MHz, $CDCl_3$) δ 7.90 – 7.82 (m, 3H), 7.76 (d, *J* = 2.3 Hz, 1H), 7.56 – 7.47 (m, 2H), 7.42 (dd, *J* = 8.9, 2.4 Hz, 1H), 3.03 – 2.99 (s, 6 H) ppm.**$^{13}$C NMR** (126 MHz, $CDCl_3$) δ 147.9, 133.8, 131.9, 130.1, 128.0, 127.9, 127.1, 126.4, 120.9, 119.2, 39.0 ppm.

This data is consistent with the literature.[8]

**1-Naphthyl dimethylsulfamate (1b)**

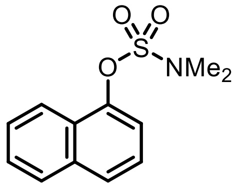


Prepared according to general procedure **A**. Purified by flash column chromatography (Hexane: DCM, 1:1) to give **1-Naphthyl dimethylsulfamate** (530 mg, 84%) as a white solid. **mp**: 76–77 °C **$^1$H NMR** (500 MHz, $CDCl_3$) δ 8.18 (dd, *J* = 8.3, 1.3 Hz, 1H), 7.88 (dd, *J* = 8.5, 1.2 Hz, 1H), 7.81 – 7.76 (m, 1H), 7.46 (t, *J* = 7.9 Hz, 1H), 3.08 – 3.06 (s, 6 H) ppm. **$^{13}$C NMR** (126 MHz, $CDCl_3$) δ 146.2, 135.0, 128.1, 127.3, 126.9, 126.8, 125.5, 121.6, 117.9, 39.1 ppm.

This data is consistent with the literature.[9]

**4-Phenylphenyl dimethylsulfamate (1c)**

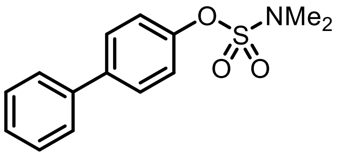


Prepared according to general procedure **A**. Purified by flash column chromatography (Hexane: Ethyl Acetate, 100:0 - 80:20 v:v) to give **4-Phenylphenyl dimethylsulfamate** (577 mg, 83%) as a white solid. **mp**: 114–117 °C **$^1$H NMR** (500 MHz, $CDCl_3$) δ 7.60 (d, *J* = 8.7 Hz, 2H), 7.58 – 7.55 (m, 2H), 7.45 (dd, *J* = 8.4, 6.9 Hz, 2H), 7.39 – 7.34 (m, 3H), 3.03 – 3.00 (s, 6H) ppm. **$^{13}$C NMR** (126 MHz, $CDCl_3$) δ 149.7, 140.1, 140.1, 129.0, 128.6, 127.7, 127.3, 122.2, 39.0 ppm.

This data is consistent with the literature.[10]

**4-Methoxy-1-naphthyl dimethylsulfamate (1d)**

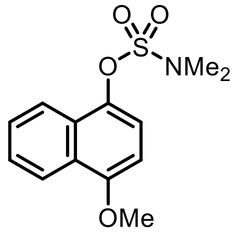


Prepared according to general procedure **A**. Purified by flash column chromatography (Hexane: Ethyl Acetate, 100:0 - 85:15 v:v) to give **4-Methoxy-1-naphthyl dimethylsulfamate** (457 mg, 65%) as a light brown solid. **mp**: 78–80 °C. **$^1$H NMR** (500 MHz, $CDCl_3$) δ 8.27 (d, *J* = 8.4 Hz, 1H), 8.10 (d, *J* = 8.3 Hz, 1H), 7.56 (dddd, *J* = 31.5, 8.3, 6.8, 1.3 Hz, 2H), 7.44 (d, *J* = 8.4 Hz, 1H), 6.75 (d, *J* = 8.4 Hz, 1H), 4.05 – 3.97 (s, 3H), 3.06 – 3.02 (s, 3H) ppm. **$^{13}$C NMR** (126 MHz, $CDCl_3$) δ 154.1, 139.6, 128.1, 127.4, 126.4, 126.2, 122.5, 121.4, 118.2, 102.8, 55.9, 39.0 ppm.

This data is consistent with the literature.[2]

**4-Methoxyphenyl dimethylsulfamate (1e)**

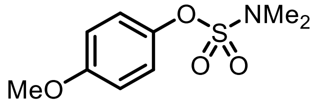


Prepared according to general procedure **A**. Purified by flash column chromatography (Hexane: Ethyl Acetate, 100:0 - 89:11 v:v) to give **4-Methoxyphenyl dimethylsulfamate** (317 mg, 55%) as a colourless oil. **$^1$H NMR** (500 MHz, $CDCl_3$) δ 7.20 (d, *J* = 9.0 Hz, 2H), 6.88 (d, *J* = 9.1 Hz, 2H), 3.82 – 3.78 (s, 3H), 2.97 – 2.94 (s, 6H) ppm. **$^{13}$C NMR** (126 MHz, $CDCl_3$) δ 158.2, 143.7, 123.0, 114.8, 55.8, 38.9 ppm

This data is consistent with the literature.[2]

**4-Cyanophenyl dimethylsulfamate (1f)**

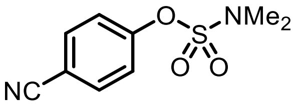


Prepared according to general procedure **A**. Purified by flash column chromatography (Hexane: DCM, 100:0 – 50:50 v:v) to give **4-Cyanophenyl dimethylsulfamate** (377 mg, 67%) as a white solid. **mp**: 71–73 °C **$^1$H NMR** (400 MHz, $CDCl_3$) δ 7.71 (d, *J* = 8.7 Hz, 2H), 7.40 (d, *J* = 8.8 Hz, 2H), 3.05 – 2.99 (s, 6 H) ppm. **$^{13}$C NMR** (101 MHz, $CDCl_3$) δ 153.6, 134.2, 122.6, 118.1, 110.7, 38.9 ppm.

This data is consistent with the literature. [8]

**Methyl-4-hydroxylbenzyl dimethylsulfamate (1g)**

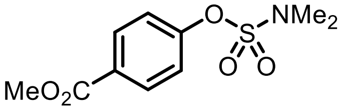


Prepared according to general procedure **A**. Purified by flash column chromatography (Hexane: Ethyl Acetate, 100:0 – 75:25 v:v) to give **Methyl-4-hydroxylbenzyl dimethylsulfamate** (359 mg, 55%) as a white solid. **mp**: 71–73 °C. **$^1$H NMR** (500 MHz, $CDCl_3$) δ 8.08 (d, *J* = 8.7 Hz, 2H), 7.35 (d, *J* = 8.7 Hz, 2H), 3.94 – 3.91 (s, 3H), 3.02 – 2.99 (s, 6H) ppm. **$^{13}$C NMR** (126 MHz, $CDCl_3$) δ 166.2, 153.9, 131.7, 128.6, 121.6, 52.5, 38.9 ppm.

This data is consistent with the literature.[8]

**pyren-1-yl dimethylsulfamate (1j)**

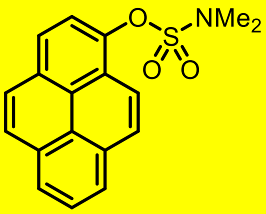


Prepared according to general procedure **A**. Purified by flash column chromatography (Hexane: DCM, 50:50 v:v) to give **pyren-1-yl dimethylsulfamate** (828 mg, 64%) as a white solid. **mp**: 180 – 185 °C. **$^1$H NMR** (500 MHz, $CDCl_3$) δ 8.43 – 8.35 (dd, *J* = 9.2, 1.3 Hz, 1H), 8.28 – 8.20 (d, *J* = 7.7 Hz, 2H), 8.20 – 8.14 (d, *J* = 9.5 Hz, 2H), 8.12 – 8.01 (m, 4H), 3.14 – 3.08 (s, 6 H)ppm. **$^{13}$C NMR** (101 MHz, $CDCl_3$) δ 143.7, 131.3, 131.1, 129.9, 128.7, 127.7, 127.1, 126.7, 125.9, 125.8, 125.6, 125.2, 124.5, 124.0, 120.6, 119.7, 39.1.ppm. **IR:** $\nu_{max}$: 2975, 2941, 1595, 1457, 1353, 1163, 1088, 1051, 965, 891, 842, 801 $cm^{-1}$.

### 11.1.3. General procedure B: Hydroxyl pyridine dimethylsulfamate synthesis

Prepared according to modified literature.[11] To an oven dried microwave vial equipped with stirring bar was added hydroxylpyridine (1 equiv.) in 1 M pyridine. N,N-Dimethylsulfamoyl chloride (1.2 equiv.) was added over 10 minutes. The reaction was heated to 45 °C and left to stir overnight.

The reaction was cooled to rt. Ether was added and washed with 1.1 M NaOH (25 mL) and the layers separated. The aqueous layers were then extracted with ether (2 x 25 mL) followed by ethyl acetate (25 mL). The combined organic layers were with brine (2 x 25 mL) and dried over magnesium sulfate. The solvent was evaporated to give the crude product.

This was then purified by flash column chromatography to give the pure product.

**3-hydroxylpyridyl dimethylsulfamate (1h)**

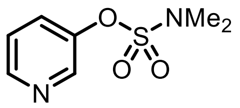

Prepared according to general procedure **B**. Purified by flash column chromatography (Hexane: DCM, 100:0 – 50:50 v:v) to give **3-hydroxylpyridyl dimethylsulfamate** (1.62 g, 80%) as a colourless oil. **$^1$H NMR** (500 MHz, $CDCl_3$) δ 8.58 – 8.52 (m, 2H), 7.67 (ddd, *J* = 8.5, 2.8, 1.4 Hz, 1H), 7.36 (dd, *J* = 8.4, 4.7 Hz, 1H), 3.03 – 3.00 (s, 6H) ppm. **$^{13}$C NMR** (126 MHz, $CDCl_3$) δ 148.0, 147.3, 143.8, 129.5, 124.4, 38.9 ppm.

This data is consistent with the literature.[8]

**2-hydroxylpyridyl dimethylsulfamate (1i)**

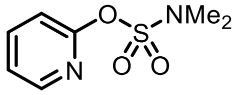

Prepared according to general procedure **B**. Purified by flash column chromatography (Hexane: Ethyl Acetate, 100:0 – 50:50 v:v) to give **2-hydroxylpyridyl dimethylsulfamate** (441 mg, 87%) as a colourless oil. **$^1$H NMR** (500 MHz, $CDCl_3$) δ 8.41 – 8.35 (m, 1H), 7.80 (ddd, *J* = 8.2, 7.3, 2.0 Hz, 1H), 7.25 – 7.23 (m, 1H), 7.19 (dt, *J* = 8.2, 0.9 Hz, 1H), 3.06 - 3.02 (s, 6 H) ppm. **$^{13}$C NMR** (126 MHz, $CDCl_3$) δ 157.6, 148.4, 140.3, 122.5, 115.5, 38.9 ppm.

This data is consistent with the literature.[11]

## 11.2. Synthesis of C-C coupled products

### 11.2.1. General Procedure C: Mechanochemical nickel-catalysed Suzuki-Miyaura cross coupling

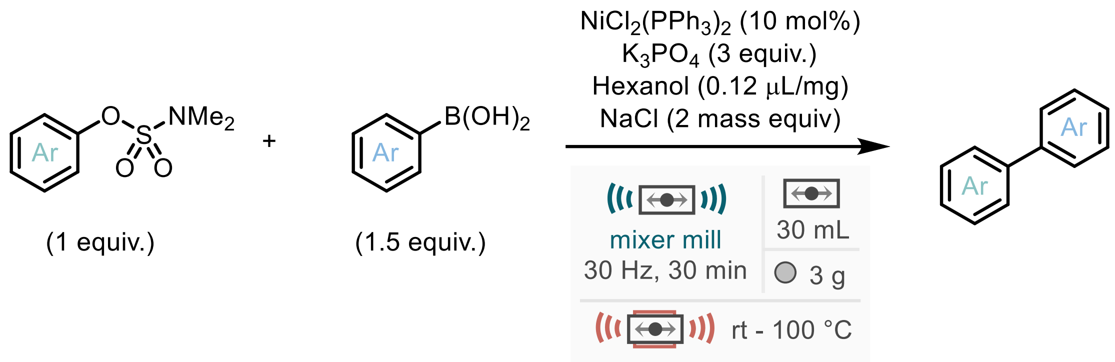


To a 30 mL stainless steel milling jar was added a 3 g stainless steel ball, aryl dimethylsulfamate (0.5 mmol), boronic acid (0.75 mmol), $NiCl_2(PPh_3)_2$ (10 mol%), sodium chloride (2 mass equiv.), hexanol (0.122 µL/mg) and tripotassium phosphate (1.5 mmol). A band heater encased the jar and milled at 30 Hz for 30 minutes. The first 10 minutes were conducted at rt. With the next 10 minutes heated to 63 °C and the remaining 10 minutes to 100 °C. After this time the milling was stopped, and jar allowed to cool to ~40 °C before further manipulation. The reaction mixture was removed from the jar into a conical flask with ethyl acetate (~30 mL) and water (~30 mL) *. The organic layer was washed with 1.1 M NaOH (25 mL), water (50 mL), brine (50 mL), dried over magnesium sulfate, and concentrated *in vacuo* to give the crude reaction mixture.** The resulting crude residue was purified by silica gel flash column chromatography using hexane/ ethyl acetate solvent system to give the pure product unless stated otherwise.

*a sonicator was used to break up any large pieces

** a dilute $H_2O_2$ solution (25 mL) was also used for boronic acids containing electron-donating groups to facilitate removal of $PPh_3$ ligand, which could co-elute with C-C coupled products.

**2-(4-fluorophenyl) naphthalene (3a)**

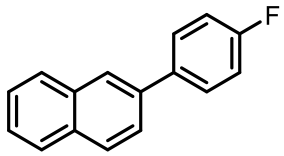


Prepared according to general procedure **C**. Purified by flash column chromatography (Hexane) to give **2-(4-fluorophenyl) naphthalene** (97 mg, 87%) as a white solid. **mp**: 102–105 °C. **$^1$H NMR** (400 MHz, $CDCl_3$) δ 8.00 – 7.89 (s, 1H), 7.89 – 7.76 (m, 3H), 7.70 – 7.53 (m, 3H), 7.53 – 7.38 (m, 2H), 7.20 – 7.02 (m, 2 H) ppm. **$^{13}$C NMR** (101 MHz, $CDCl_3$) δ 162.7 (d, *J* = 246.5 Hz) 137.8, 137.4 (d, *J* = 2.8 Hz) 133.8, 132.7, 129.2 (d, *J* = 8.3 Hz), 128.7, 128.3, 127.8, 126.6, 126.2, 125.8, 125.6, 115.9 (d, *J* = 21.2 Hz) ppm.

This data is consistent with the literature.[12]

**2-phenyl naphthalene (3b)**

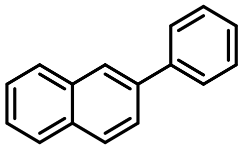

Prepared according to general procedure **C**. Purified by flash column chromatography (Hexane) to give **2-phenyl naphthalene** (74 mg, 72%) as a white solid. **mp**: 101–103 °C. **$^{1}$H NMR** (500 MHz, $CDCl_3$) δ 8.09 – 8.02 (s, 1H), 7.93 – 7.86 (m, 3H), 7.76 – 7.72 (m, 3H), 7.52 – 7.48 (m, 4H) 7.40 – 7.37 (t, *J* = 7.4 Hz, 1H) ppm. **$^{13}$C NMR** (500 MHz, $CDCl_3$) δ 141.1, 138.5, 133.6, 132.6, 128.9, 128.4, 128.2, 127.6, 127.4, 127.4, 126.3, 125.9, 125.8, 125.6 ppm.

This data is consistent with the literature. [13]

**2-(4-methoxyphenyl) naphthalene (3c)**

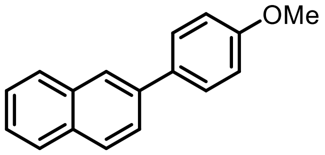


Prepared according to general procedure **C** however using 78 °C as the mid temperature and 130 °C as the final temperature. Purified by flash column chromatography (Hexane: Ethyl Acetate, 100:0 - 97:3 v:v) to give **2-(4-methoxyphenyl) naphthalene** (76 mg, 70%) as a white solid. **mp**: 132–136 °C. **$^{1}$H NMR** (400 MHz, $CDCl_3$) δ 8.02 – 7.97 (s, 1H), 7.95 – 7.81 (m, 3H), 7.76 – 7.70 (dd, *J* = 8.6, 1.9 Hz, 1H), 7.69 – 7.63 (m, 2H), 7.53 – 7.43 (m, 2H), 3.91 – 3.86 (s, 3H) ppm**.** **$^{13}$C NMR** (101 MHz, $CDCl_3$) δ 159.4, 138.3, 133.9, 133.8, 128.6, 128.5, 128.2, 127.8, 126.4, 125.8, 125.6, 125.2, 114.5, 55.5 ppm.

This data is consistent with the literature.[2]

**2-(4-isopropoxyphenyl)naphthalene (3f)**

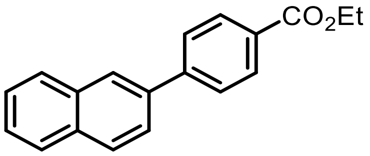


Prepared according to general procedure **C** however using 78 °C as the mid temperature and 130 °C as the final temperature. Purified by flash column chromatography (Hexane: Ethyl Acetate, 100:0 - 90:10 v:v) to give **2-(4-isopropoxyphenyl) naphthalene** (47 mg, 36%) as a white solid. **mp**: 105–109 °C. **$^{1}$H NMR** (400 MHz, $CDCl_3$) δ 8.01 – 7.98 (s, 1H), 7.92 – 7.82 (m, 3H), 7.76 – 7.70 (dd, *J* = 8.5, 1.9 Hz, 1H), 7.68 – 7.62 (m, 2H), 7.53 – 7.43 (m, 2H), 7.04 – 6.98 (m, 2H), 4.71 – 4.56 (hept, *J* = 6.1 Hz, 1H), 1.44 – 1.35 (d, *J* = 6.1 Hz, 6H) ppm. **$^{13}$C NMR** (101 MHz, $CDCl_3$) 157.6, 138.2, 133.8, 133.4, 132.3, 128.4, 128.3, 128.1, 127.6, 126.2, 125.6, 125.5, 125.0, 116.2, 70.0, 22.1 ppm. **IR:** $v_{max}$: 3314, 2978, 2926, 1595, 1498 $cm^{-1}$. **HRMS** (ESI): m/z calculated for $C_{19}H_{18}O$ requires 363.1436 for $[M+H]^+$, found 326.1432.

**2-(4-Trifluoromethylphenyl) naphthalene (3g)**

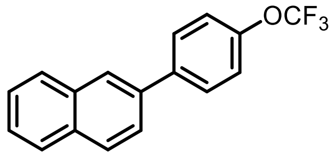


Prepared according to general procedure **C**. Purified by flash column chromatography (Hexane: Ethyl Acetate, 100:0 – 90:10 v:v) to give **2-(4-Trifluoromethylphenyl) naphthalene** (156 mg, 54%) as a white solid. **mp**: 107–110 °C. **$^1$H NMR** (500 MHz, $CDCl_3$) δ 8.04 – 8.00 (s, 1H), 7.96 – 7.85 (m, 3H), 7.77 – 7.68 (m, 3H), 7.56 – 7.48 (tt, *J* = 6.9, 5.2 Hz 2H), 7.36 – 7.31 (d, *J* = 7.7 Hz, 2H) ppm. **$^{13}$C NMR** (126 MHz, $CDCl_3$) 148.8, 140.0, 137.3, 133.7, 132.8, 128.9, 128.8, 128.3, 127.8, 126.7, 126.4, 126.1, 125.4, 121.5, 120.7 (q, *J* = 257.0 Hz) ppm **IR:** $\nu_{max}$: 3060, 1607, 1502, 1267, 1208, 1156, 1111, 1014 $cm^{-1}$. **$^{19}$F{$^1$H} NMR** (659 MHz, $CDCl_3$) δ -57.58.

Analysis by HRMS was not possible due to lack of ionisation despite multiple attempts.

**2-(4-methylphenyl) naphthalene (3h)**

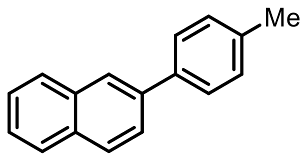


Prepared according to general procedure **C**. Purified by flash column chromatography (Hexane) to give **2-(4-methylphenyl) naphthalene** (55 mg, 50%) as a white solid. **mp**: 94–96 °C. **$^1$H NMR** (400 MHz, $CDCl_3$) δ 8.05 – 8.01 (s, 1H), 7.94 – 7.84 (m, 3H), 7.77 – 7.72 (dd, *J* = 8.5, 1.8 Hz, 1H), 7.66 – 7.61 (d, *J* = 8.1 Hz, 2H), 7.54 – 7.44 (m, 2H), 7.33 – 7.28 (d, *J* = 7.8 Hz, 2H), 2.46 – 2.41 (s, 3H) ppm. **$^{13}$C NMR** (101 MHz, $CDCl_3$) δ 138.6, 138.4, 137.3, 133.9, 132.7, 129.7, 128.5, 128.3, 127.8, 127.4, 126.4, 125.9, 125.7, 125.6, 21.3 ppm.

This data is consistent with the literature.[12]

**2-(4-n-Butyl phenyl) naphthalene (3i)**

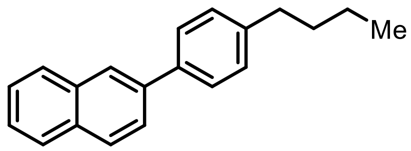


Prepared according to general procedure **C** however using 73 °C as the mid temperature and 120 °C as the final temperature. Purified by flash column chromatography (Hexane) to give **2-(4-n-Butyl phenyl) naphthalene** (67 mg, 52%) as a white solid. **mp**: 45–48 °C. **$^1$H NMR** (400 MHz, $CDCl_3$) δ 8.06 – 8.01 (s, 1H), 7.96 – 7.83 (m, 3H), 7.79 – 7.72 (dd, *J* = 8.5, 1.8 Hz, 1H), 7.68 – 7.62 (m, 2H), 7.54 – 7.43 (m, 2H), 7.34 – 7.27 (m, 2H), 2.75 – 2.63 (m, 2H), 1.73 – 1.59 (m, 2H), 1.49 – 1.35 (h, *J* = 7.4 Hz, 2 H), 1.04 – 0.92 (t, *J* = 7.3 Hz, 3H) ppm. **$^{13}$C NMR** (101 MHz, $CDCl_3$) δ142.4, 138.7, 138.6, 133.9, 132.7, 129.1, 128.5, 128.3, 127.8, 127.4, 126.4, 125.9, 125.7, 125.6, 35.5, 33.8, 22.6, 14.1 ppm.

This data is consistent with the literature.[12]

**2-(4-tert-butylphenyl) naphthalene (3j)**

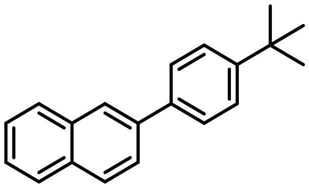

Prepared according to general procedure **C** however using 73 °C as the mid temperature and 120 °C as the final temperature. Purified by flash column chromatography (Hexane) to give **2-(4-tert-butylphenyl) naphthalene** (88 mg, 68%) as a white solid. **mp**: 110–114 °C. **$^1$H NMR** (400 MHz, $CDCl_3$) δ 8.07 – 8.01 (s, 1H), 7.94 – 7.82 (m, 3H), 7.79 – 7.72 (dd, *J* = 8.5, 1.9 Hz, 1H), 7.72 – 7.65 (m, 2H), 7.57 – 7.43 (m, 4H), 1.42 – 1.38 (s, 9H) ppm. **$^{13}$C NMR** (101 MHz, $CDCl_3$) δ 150.6, 138.6, 138.4, 133.9, 132.7, 128.5, 128.3, 127.8, 127.2, 126.4, 126.0, 125.9, 125.7, 125.7, 34.7, 31.5 ppm.

This data is consistent with the literature.[14]

**2-(4-Acetylphenyl) naphthalene (3k)**

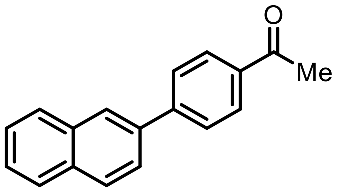


Prepared according to general procedure **C**. Purified by flash column chromatography (Hexane: Ethyl Acetate, 100:0 - 93:7 v:v) to give **2-(4-Acetylphenyl) naphthalene** (95 mg, 77%) as a white solid. **mp**: 115–121 °C. **$^1$H NMR** (400 MHz, $CDCl_3$) δ 8.10 – 8.08 (m, 3H), 7.96 – 7.88 (m, 3H), 7.84 – 7.82 (d, *J* = 8.5 Hz, 2H), 7.78 – 7.76 (dd, *J* = 1.9, 8.5 Hz, 1H), 7.74 – 7.52 (m, 2H), 2.71 – 2.61 (s, 3H). **$^{13}$C NMR** (101 MHz, $CDCl_3$) δ 197.8, 145.7, 137.1, 135.8, 133.5, 133.0, 129.0, 128.7, 128.4, 127.7, 127.5, 126.6, 126.5, 126.4, 125.2, 26.7 ppm.

This data is consistent with the literature. [15]

**2-(4-ethoxycarbonylphenyl) naphthalene (3l)**

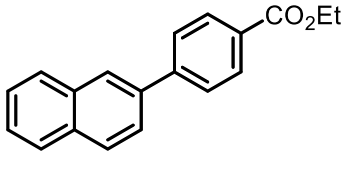


Prepared according to general procedure **C**. Purified by flash column chromatography (Hexane: Ethyl Acetate, 100:0 - 95:5 v:v) to give **2-(4-ethoxycarbonylphenyl) naphthalene** (80 mg, 58%) as a white solid. **mp**: 87–91 °C. **$^1$H NMR** (400 MHz, $CDCl_3$) δ 8.16 – 8.14 (d, *J* = 8.5 Hz, 2H), 8.10 – 8.04 (s, br, 1H), 7.94 – 7.86 (m, 3H) 7.79 – 7.75 (m, 3H), 7.54 – 7.49 (m, 2H), 4.44 – 4.39 (q, *J* =7.2 Hz, 2H) 1.44 – 1.41 (t, *J* =7.2 Hz, 3H) ppm. **$^{13}$C NMR** (101 MHz, $CDCl_3$) δ166.5, 145.4, 137.3, 133.5, 132.9, 130.1, 129.2, 128.6, 128. 3, 127.6, 127.2, 126.5, 126.4, 126.3, 125.2, 61.0, 14.4 ppm.

This data is consistent with the literature. [16]

**2-(4-trifluorophenyl) naphthalene (3m)**

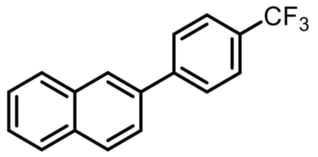


Prepared according to general procedure **C**. Purified by flash column chromatography (Hexane) to give **2-(4-trifluorophenyl) naphthalene** (77 mg, 57%) as a white solid. **mp**: 129–132 °C. **$^1$H NMR** (400 MHz, $CDCl_3$) δ 8.09 – 8.03 (s, br, 1H), 7.95 – 7.87 (m, 3H), 7.83 – 7.81 (d, *J* = 8.0 Hz, 2H), 7.74 – 7.72 (m, 3H), 7.55 – 7.50 (m, 2H) ppm. **$^{13}$C NMR** (101 MHz, $CDCl_3$) δ 144.6, 137.0, 133.5, 132.9, 129.3 (q, *J* = 33.0 Hz), 128.8, 128.3, 127.7, 127.7, 126.6, 126.5, 126.3, 125.8 (q, *J* =3.7 Hz), 125.2, 123.2 ppm.

This data is consistent with the literature. [17]

**2-(4-cyanophenyl) naphthalene (3n)**

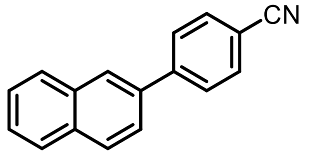


Prepared according to general procedure **C**. Purified by flash column chromatography (Hexane: Ethyl Acetate, 100:0 - 90:10 v:v ) to give **2-(4-cyanophenyl) naphthalene** (34 mg, 30%) as a white solid. **mp**: 156–161 °C. **$^1$H NMR** (400 MHz, $CDCl_3$) δ 8.02 – 7.97 (s, br, 1H), 7.96 – 7.87 (m, 3H), 7.83 – 7.80 (d, *J* =8.7 Hz, 2H), 7.77 – 7.75 (d, *J* = 8.6 Hz, 2H), 7.72 – 7.69 (dd, *J* = 1.9, 8.6 Hz, 1H), 7.56 – 7.51 (m, 2H) ppm. **$^{13}$C NMR** (101 MHz, $CDCl_3$) δ 145.6, 136.4, 133.5, 133.1, 132.6, 128.9, 128.4, 127.9, 127.7, 126.8, 126.7, 126.5, 124.8, 118.9, 110.9 ppm.

This data is consistent with the literature. [18]

**3-(dibenzo[b,d]thiophen-2-yl)pyridine (3q)**

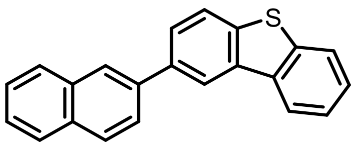


Prepared according to general procedure **C**. Purified by flash column chromatography (Hexane) to give **3-(dibenzo[b,d]thiophen-2-yl)pyridine** (129 mg, 83%) as a white solid. **mp**: 102–106 °C. **$^1$H NMR** (500 MHz, $CDCl_3$) δ 8.51 – 8.46 (s, 1H), 8.29 – 8.26 (m, 1H), 8.18 – 8.15 (s, 1H), 7.99 – 7.82 (m, 7H), 7.55 – 7.49 (m, 4H) ppm. **$^{13}$C NMR** (101 MHz, $CDCl_3$) δ 140.0, 138.7, 138.5, 137.8, 136.2, 135.6, 133.8, 132.7, 128.6, 128.2, 127.7, 126.9, 126.4, 126.0, 125.8, 124.5, 123.1, 123.0, 121.7, 120.3 ppm. **IR:** $v_{max}$: 3306, 1725, 1625, 1524, 1453, 1375, 1341 cm$^{-1}$.

Analysis by HRMS was not possible due to lack of ionisation despite multiple attempts.

**1-(4-Fluorophenyl)naphthalene (3d)**

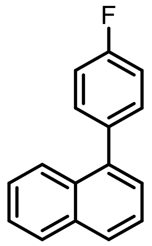

Prepared according to general procedure **C**. Purified by flash column chromatography (Hexane) to give **1-(4-Fluorophenyl)naphthalene** (89 mg, 80%) as a white solid. **mp**: 68–71 °C. **$^1$H NMR** (400 MHz, $CDCl_3$) δ 7.95 – 7.89 (d, *J* = 8.1 Hz, 1H), 7.89 – 7.82 (t, *J* = 8.9 Hz, 2H), 7.59 – 7.38 (m, 6H), 7.24 – 7.15 (t, *J* = 8.7 Hz 2H) ppm. **$^{13}$C NMR** (101 MHz, $CDCl_3$) δ 162.3 (d, *J* = 246.41 Hz) 139.1, 136.6 (d, *J* = 3.0 Hz), 133.8, 131.6, 131.5, 128.3, 127.8, 127.0, 126.2, 125.9, 125.8, 125.4, 115.2 (d, *J* =21.1 Hz) ppm

This data is consistent with the literature.[9]

**4-Fluoro-1,1':4',1''-terphenyl (3e)**

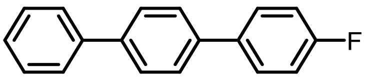

Prepared according to general procedure **C**. Purified by flash column chromatography (Hexane) to give **4-Fluoro-1,1':4',1''-terphenyl** (72 mg, 58%) as a white solid. **mp**: 215–218 °C. **$^1$H NMR** (400 MHz, $CDCl_3$) δ 7.69 – 7.59(m, 8H), 7.49 – 7.46 (t, *J* =7.7 Hz, 2H), 7.39 – 7.36 (t, *J* =7.4 Hz , 3H), 7.17 – 7.14 (t, *J* =8.6 Hz, 2H) ppm. **$^{13}$C NMR** (101 MHz, $CDCl_3$) δ 162.5 (d, *J* = 246.5 Hz), 140.6, 140.1, 139.1, 136.8(d, *J* = 3.5 Hz), 128.8, 128.5 (d, *J* =8.2 Hz), 127.6, 127.4, 127.4, 127.0, 115.7 (d, *J* = 21.2 Hz) ppm

This data is consistent with the literature. [19]

**1-(4-fluorophenyl)-4-methoxynaphthalene (3r)**

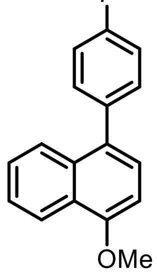

Prepared according to general procedure **C**. Purified by flash column chromatography (Hexane: DCM, 100:0 – 95:5 v:v) to give **1-(4-fluorophenyl)-4-methoxynaphthalene** (72 mg, 57%) as a white solid. **mp**: 94–97 °C. **$^1$H NMR** (400 MHz, $CDCl_3$) δ 8.37 – 8.35 (d, *J* = 8.0Hz, 1H), 7.82 – 7.80 (d, *J* =8.7Hz, 1H), 7.53 –7.42 (m, 4H), 7.32 – 7.31 (d, *J* =7.8 Hz, 1H), 7.19 – 7.16 (t, *J* =8.6 Hz, 2H), 6.88 – 6.87 (d, *J* =7.9 Hz, 1H), 4.08 – 4.02 (s, 3H) ppm. **$^{13}$C NMR** (101 MHz, $CDCl_3$) δ 162.0 (d, J= 245.6 Hz), 155.0, 136.8 (d, *J* = 2.9 Hz), 132.5, 131.7 (2C), 131.5, 126.8 (d, *J* = 38.7 Hz), 125.6, 125.5, 125.2, 122.2, 115.1 (d, *J* =21.1Hz), 103.3, 55.6 ppm. **$^{19}$F{$^1$H} NMR** (659 MHz, $CDCl_3$) δ – 116.09 (s). **IR:** $v_{max}$: 3064, 3042, 3008, 2967, 2840, 2076, 1588, 1510, 1461, 1387 $cm^{-1}$.

Analysis by HRMS was not possible due to lack of ionisation despite multiple attempts.

**4-fluoro-4'-methoxy-1,1'-biphenyl (3s)**

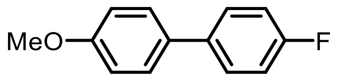


Prepared according to general procedure **C**. Purified by flash column chromatography (Hexane: Ethyl Acetate, 100:0 - 95:5 v:v) to give **4-fluoro-4'-methoxy-1,1'-biphenyl** (38 mg, 37%) as a white solid. **mp**: 87–88 °C. **$^1$H NMR** (400 MHz, $CDCl_3$) δ 7.52 – 7.46 (m, 4H), 7.13 – 7.08 (t, *J* =8.7 Hz, 2H), 6.97 – 6.94 (d, *J* =9.0 Hz, 2H), 3.89 – 3.82 (s, 3H) ppm. **$^{13}$C NMR** (101 MHz, $CDCl_3$) δ 162.1 (d, *J* = 245.6 Hz), 159.1, 137.0 (d, *J* = 2.8 Hz), 132.8, 128.2 (d, *J* = 7.5 Hz), 128.0, 115.5 (d, *J* = 21.1z Hz), 114.2, 55.4 ppm

This data is consistent with the literature.[20]

**4'-fluoro-[1,1'-biphenyl]-4-carbonitrile (3t)**

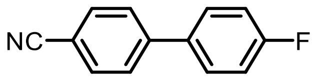


Prepared according to general procedure **C**. Purified by flash column chromatography (Hexane: Ethyl Acetate, 100:0 - 90:10 v:v) to give **4'-fluoro-[1,1'-biphenyl]-4-carbonitrile** (64 mg, 64%) as a white solid. **mp**: 115–118 °C. **$^1$H NMR** (400 MHz, $CDCl_3$) δ 7.72 – 7.70 (d, *J* = 8.4 Hz, 2H), 7.64 – 7.62 (d, *J* = 8.4 Hz, 2H), 7.57 – 7.53 (m, 2H), 7.18 – 7.14 (t, *J* = 8.6, Hz, 2H) pm. **$^{13}$C NMR** (MHz, $CDCl_3$) δ 163.2 (d, *J* = 249.2 Hz), 144.6, 135.3 (d, *J* = 2.9 Hz), 132.7, 129.0 (d, *J* = 8.3 Hz), 127.6, 118.9, 116.1 (d, *J* = 21.9 Hz), 111.0 ppm

This data is consistent with the literature.[21]

**Methyl 4'-fluoro-[1,1'-biphenyl]-4-carboxylate (3u)**

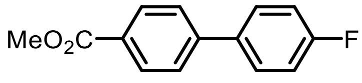


Prepared according to general procedure **C**. Purified by flash column chromatography (Hexane: Ethyl Acetate, 100:0 - 95:5 v:v) to give **methyl 4'-fluoro-[1,1'-biphenyl]-4-carboxylate** (62 mg, 54%) as a white solid. **mp**: 98–100 °C. **$^1$H NMR** (400 MHz, $CDCl_3$) δ 8.12 – 8.09 (d, *J* = 8.1 Hz, 2H), 7.62 – 7.57(m, 4H), 7.17 – 7.14 (t, *J* = 8.7 Hz, 2H), 3.96 – 3.92 (s, 3H) ppm. **$^{13}$C NMR** (101 MHz, $CDCl_3$) δ 167.1, 163.1 (d, *J* = 248.2 Hz), 144.7, 136.3 (d, *J* =3.5 Hz), 130.3, 129.1 (d, *J* =8.2 Hz), 127.0, 115.9 (d, *J* = 21.2 Hz), 52.3 ppm

This data is consistent with the literature.[22]

**2-(4-Fluorophenyl)-pyridine (3v)**

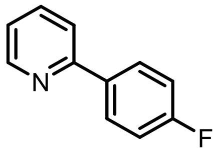

Prepared according to general procedure **C**. Purified by flash column chromatography (Hexane: Ethyl acetate 3:2) to give **2-(4-Fluorophenyl)-pyridine** (5 mg, 5%) as a yellow oil. **$^{1}$H NMR** (400 MHz, $CDCl_3$) δ 8.68 – 8.67 (d, *J* = 4.7 Hz, 1H), 8.00 – 7.95 (m, 2H), 7.77 – 7.73 (t, *J* = 7.7Hz, 1H), 7.69 – 7.67 (d, *J* = 8.0 Hz, 1H), 7.24 – 7.22 (t, *J* = 6.2 Hz, 1H), 7.18 – 7.13 (t, J= 8.7Hz, 2H) ppm. **$^{13}$C NMR** (101 MHz, $CDCl_3$) δ 164.5, 162.5, 156.6, 149.7, 136.8, 135.5 (d, *J* = 3.6 Hz), 128.7 (d, *J* = 6.6 Hz), 122.0, 120.2, 115.7 (d, *J* = 21.5 Hz) ppm

This data is consistent with the literature.[23]

**3-(4-Fluorophenyl)-pyridine (3w)**

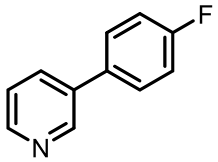

Prepared according to general procedure **C**. Purified by flash column chromatography (Hexane: Ethyl Acetate, 100:0 – 99:1 v:v) to give **3-(4-Fluorophenyl)-pyridine** (45 mg, 52%) as a yellow oil. **$^{1}$H NMR** (400 MHz, $CDCl_3$) δ 8.82 – 8.81 (d, *J* = 2.4 Hz, 1H), 8.60 – 8.59 (dd, *J* =1.6, 4.9 Hz, 1H), 7.85 – 7.82 (ddd, *J* = 1.6, 2.4, 7.9 Hz, 1H), 7.57 – 7.53 (m, 2H), 7.38 – 7.36 (ddd, *J* =0.9, 4.8, 7.9 Hz, 1H), 7.20 – 7.16 (t, *J* = 8.6 Hz, 2H) ppm. **$^{13}$C NMR** (101 MHz, $CDCl_3$) δ 162.9 (d, *J* = 248.1 Hz), 148.5, 148.1, 135.7, 134.3, 133.9 (d, *J* = 2.8 Hz), 128.8 (d, *J* = 8.1 Hz), 123.6, 116.1 (d, *J* = 21.4 Hz) ppm

This data is consistent with the literature.[24]

**1-(4-fluorophenyl)pyrene (3x)**

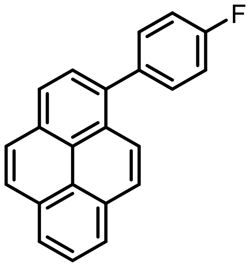

Prepared according to general procedure **C**. Purified by flash column chromatography (Hexane: Ethyl Acetate, 100:0 v:v) to give **1-(4-fluorophenyl)pyrene** (48 mg, 33%) as a white solid. **mp**: 91 – 95 °C **$^{1}$H NMR** (400 MHz, $CDCl_3$) δ 8.27 – 8.20 (m, 2H), 8.20 – 8.16 (d, *J* = 7.6 Hz, 2H), 8.15 – 8.09 (d, *J* = 9.7 Hz, 3H), 8.07 – 7.99 (m, 2H), 7.98 – 7.92 (d, *J* = 7.8 Hz, 1H), 7.63 – 7.55 (m, 2H), 7.30 – 7.23 (m, 2H) ppm. **$^{13}$C NMR** (101 MHz, $CDCl_3$) δ 162.50 (d, *J* = 246.5 Hz), 137.3 (d, *J* = 3.3 Hz), 136.8, 132.2 (d, *J* = 7.9 Hz), 131.6, 131.1, 130.9, 128.7, 127.8, 127.7, 127.7, 127.5, 126.2, 125.4, 125.1, 125.1, 125.1, 125.0, 124.8, 115.5 (d, *J* = 21.4 Hz). ppm. **$^{19}$F{$^{1}$H} NMR** (376 MHz, $CDCl_3$) δ – 115.44 (s). **IR:** $v_{max}$: 3045, 2915, 1602, 1495, 1219, 831 $cm^{-1}$.

**4,4’-Difluoro-1,1’-biphenyl (4a)**

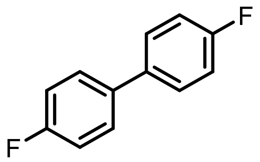


$^{1}$**H NMR** (400 MHz, $CDCl_3$) δ 7.57 – 7.44 (m, 4H), 7.18 – 7.07 (m, 4H) ppm.

This data is consistent with the literature.[25]

## 12. NMR spectra

### 2-Naphthyl pivalate (1aa)

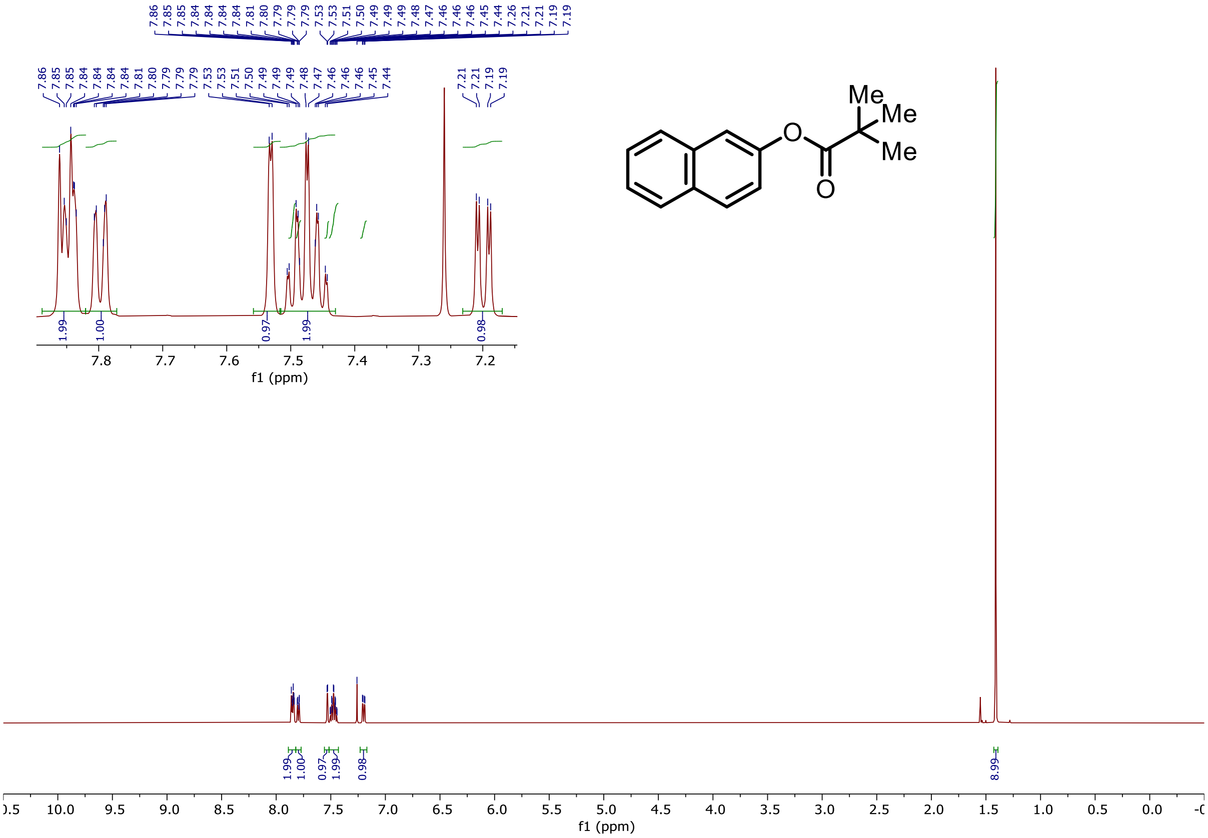


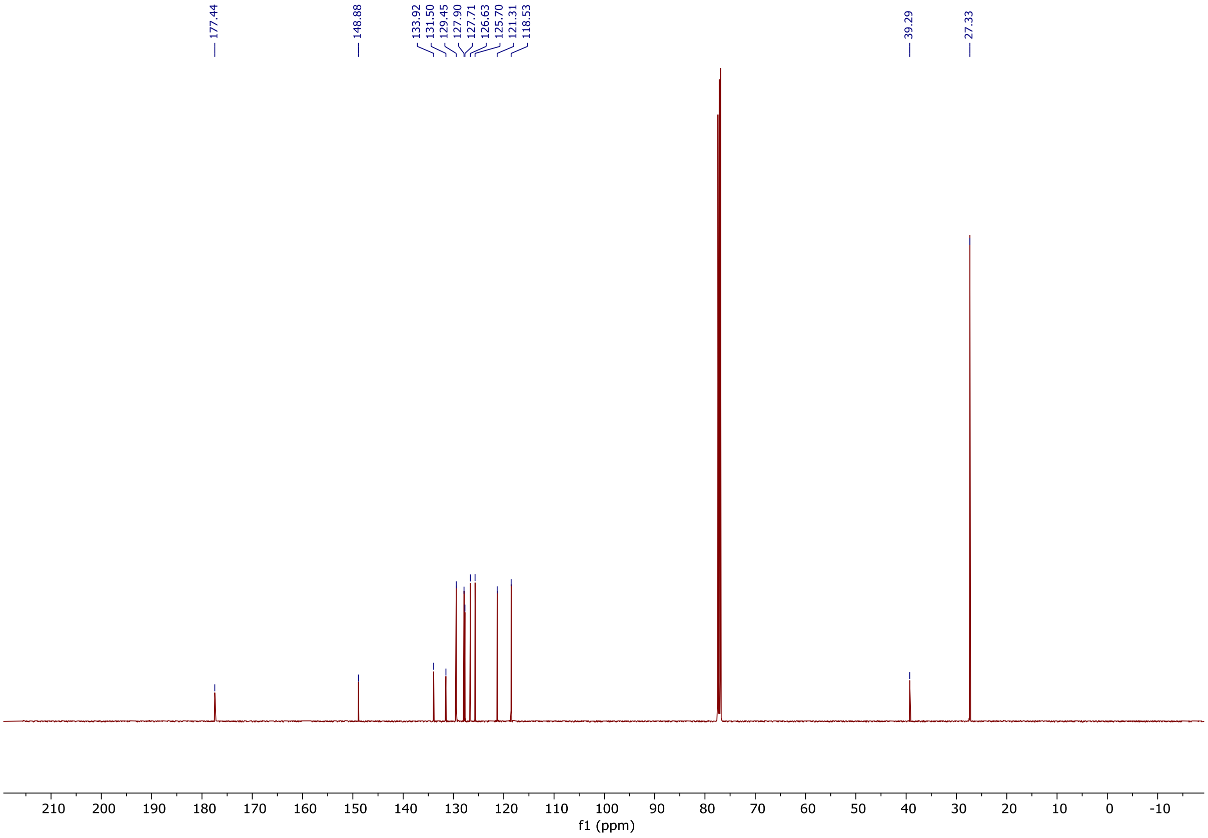

***tert*-Butyl 2-naphthyl carbonate (1ab)**

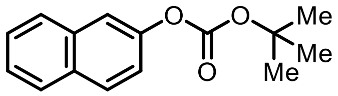

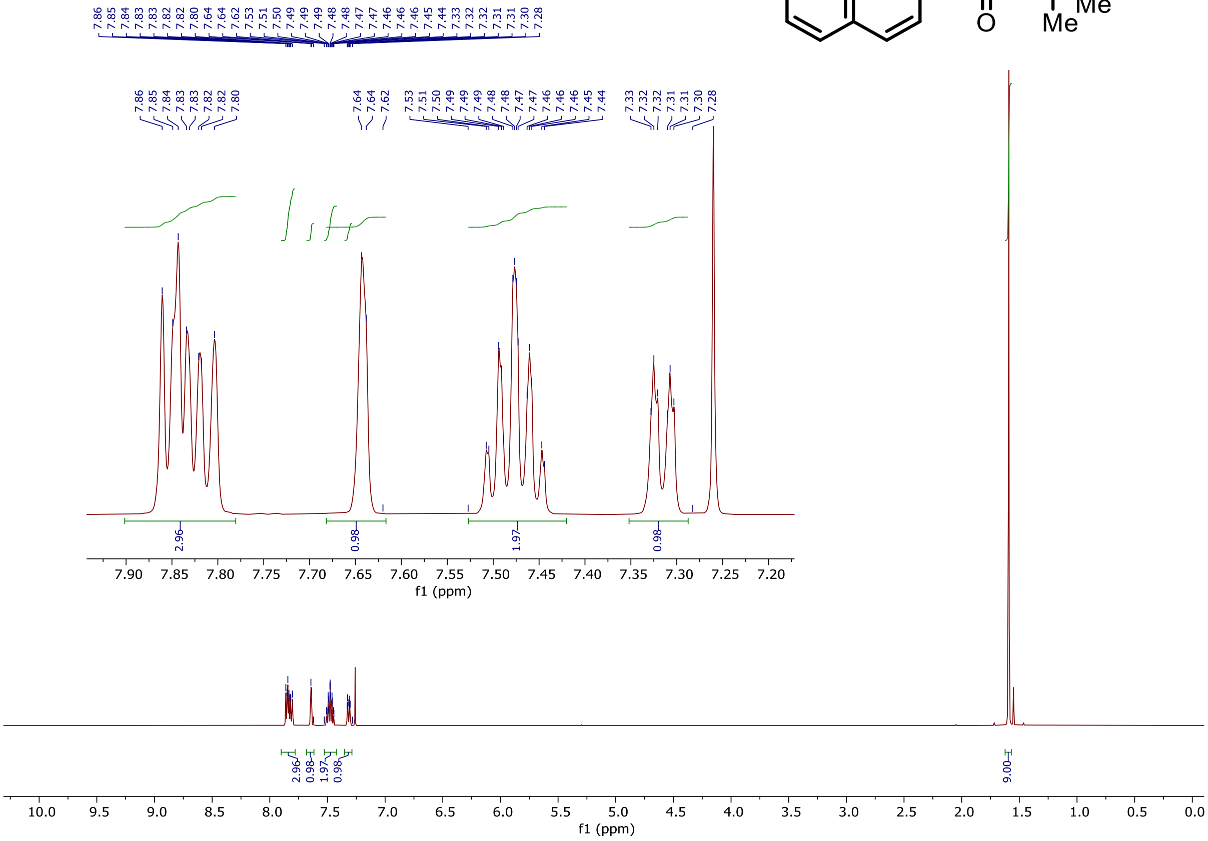


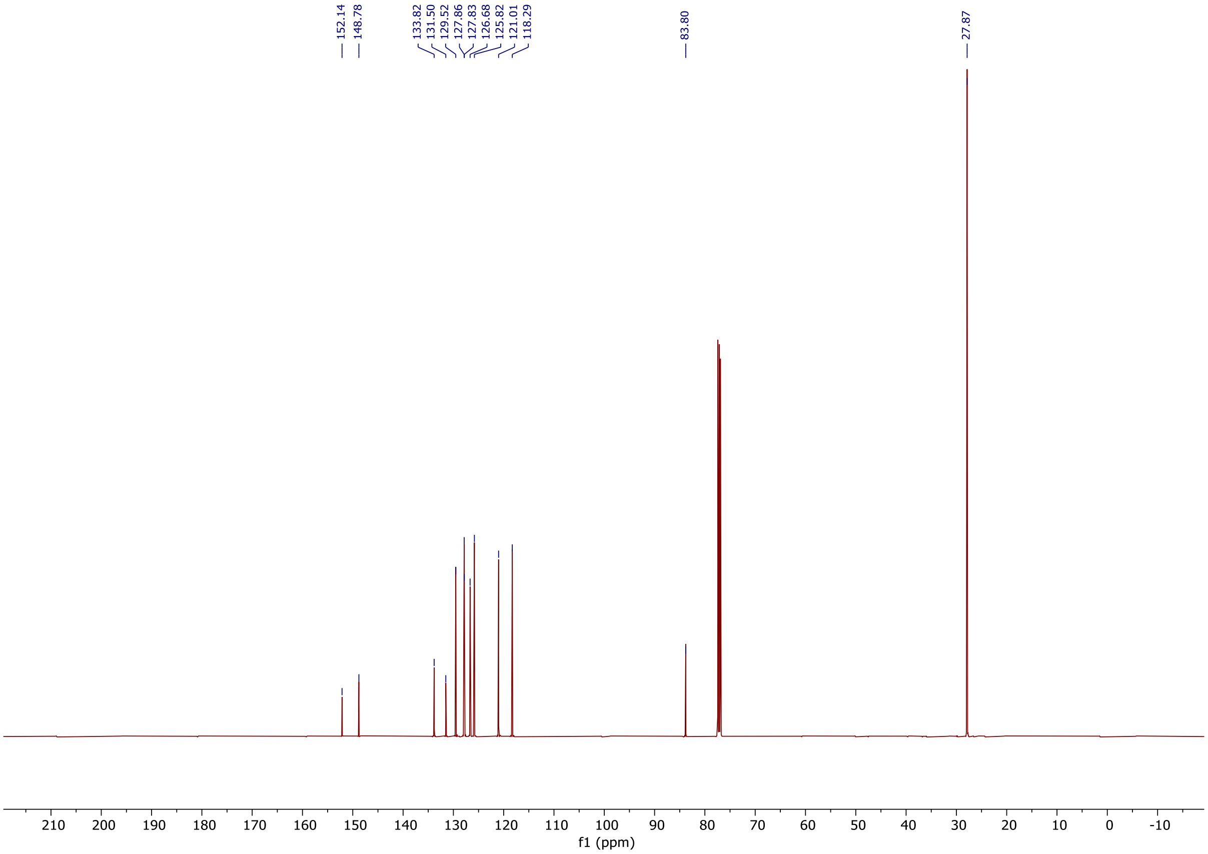

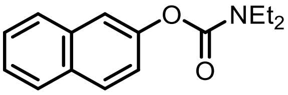


**2-Naphthyl diethylcarbamate (1ac)**

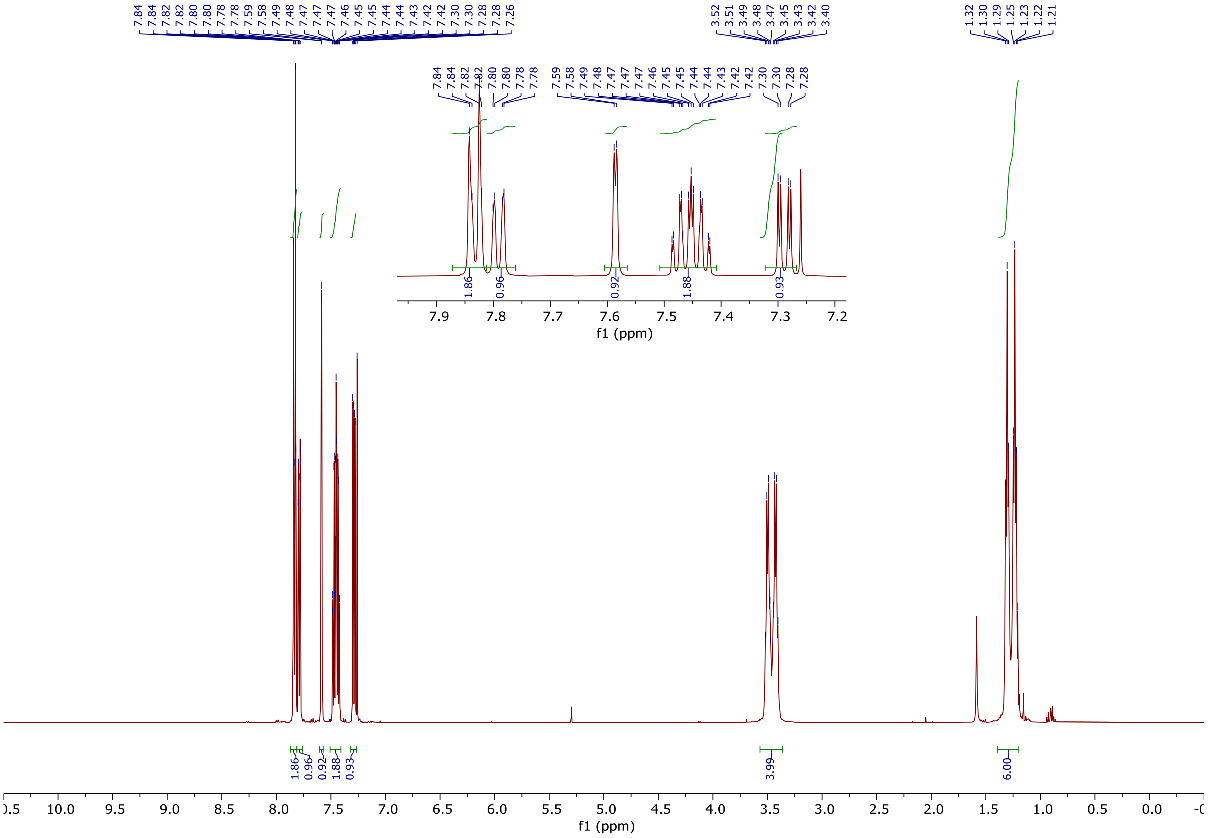


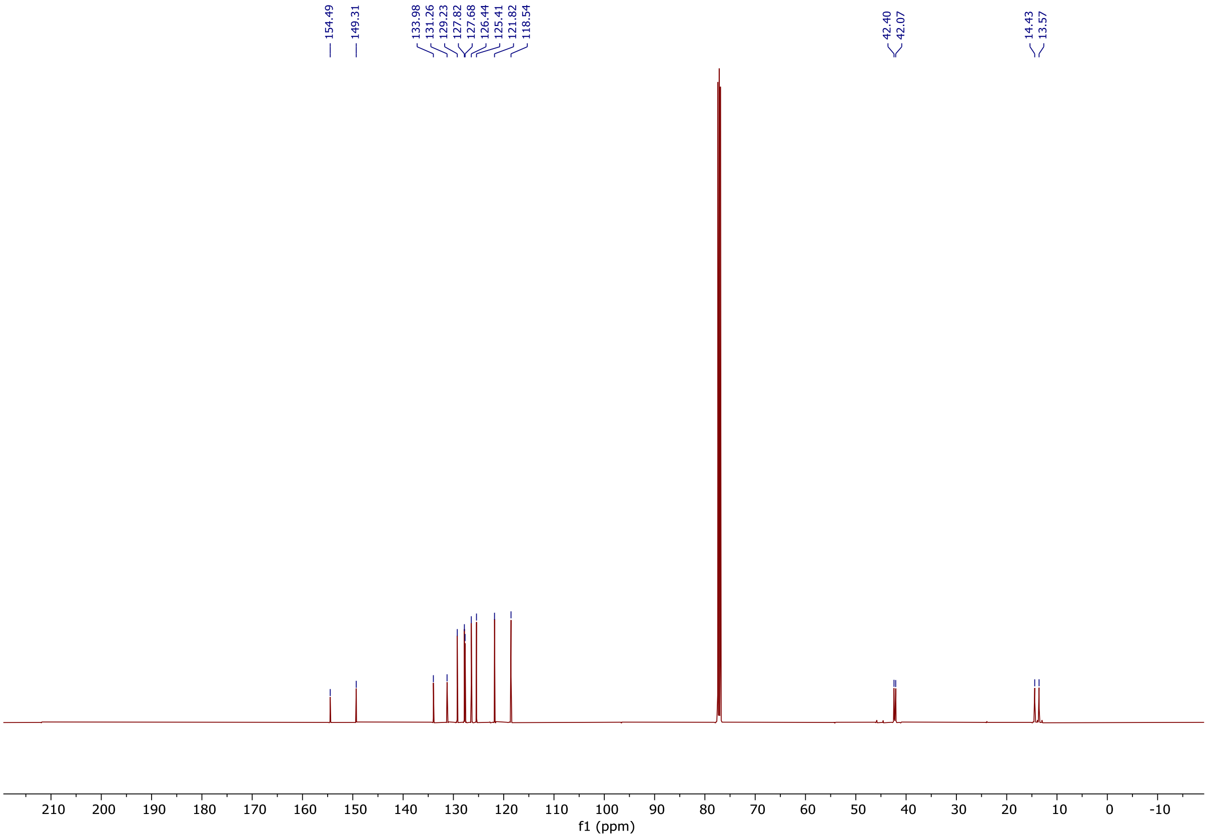

**2-Naphthyl triflate (1ad)**

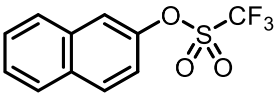

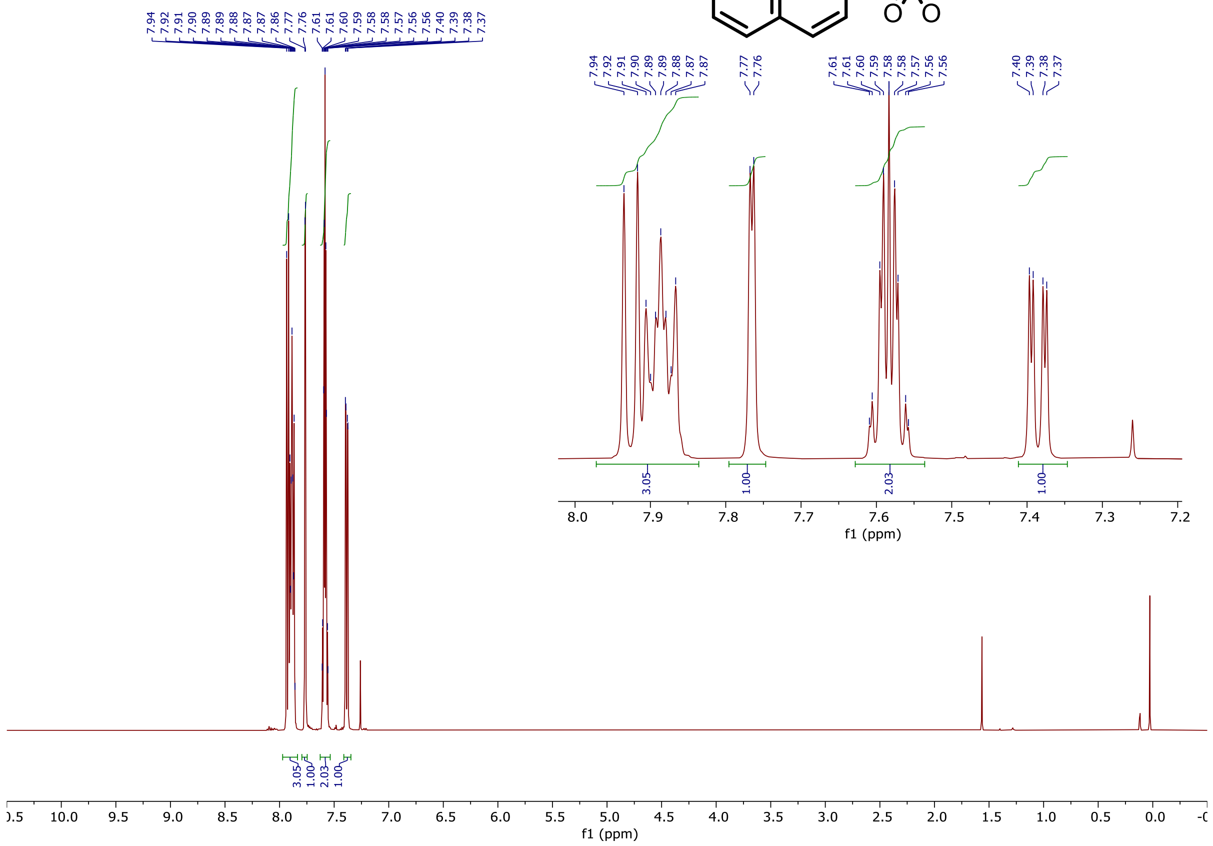

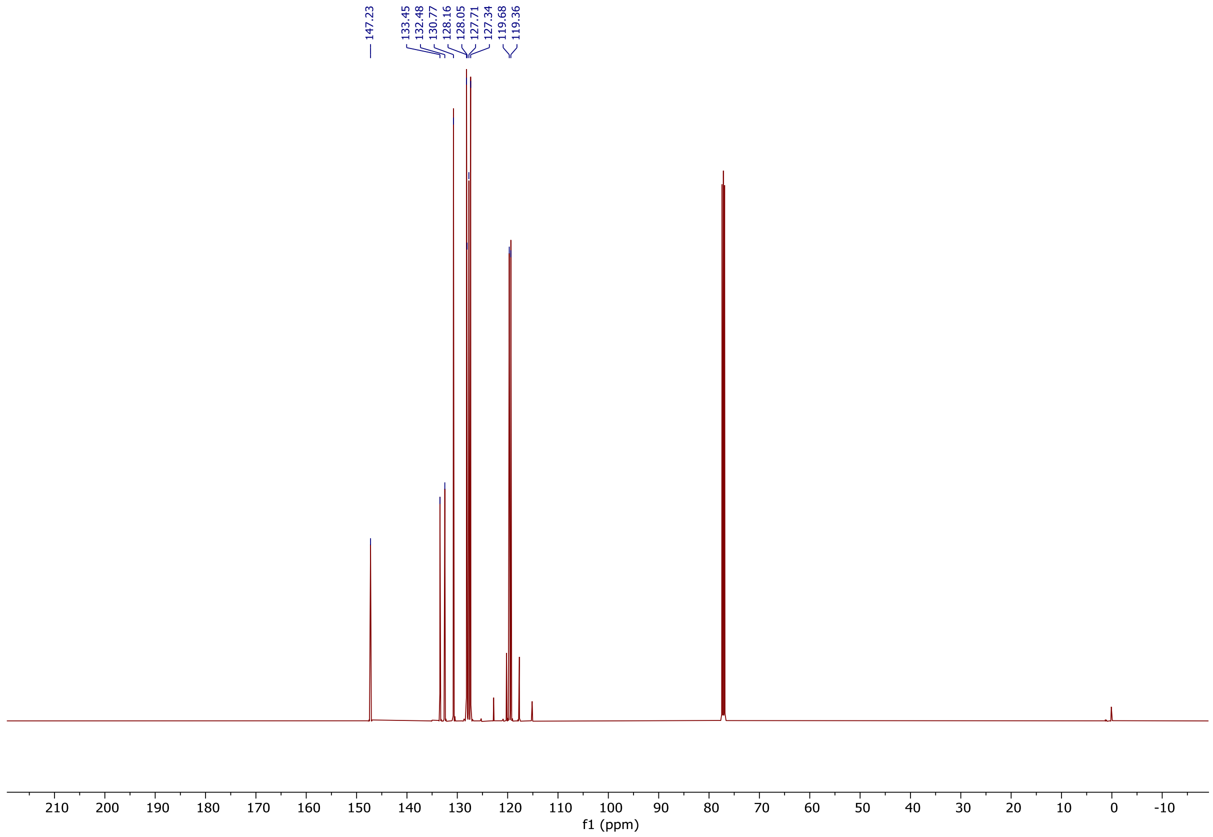

# 2-Naphthyl tosylate (1ae)

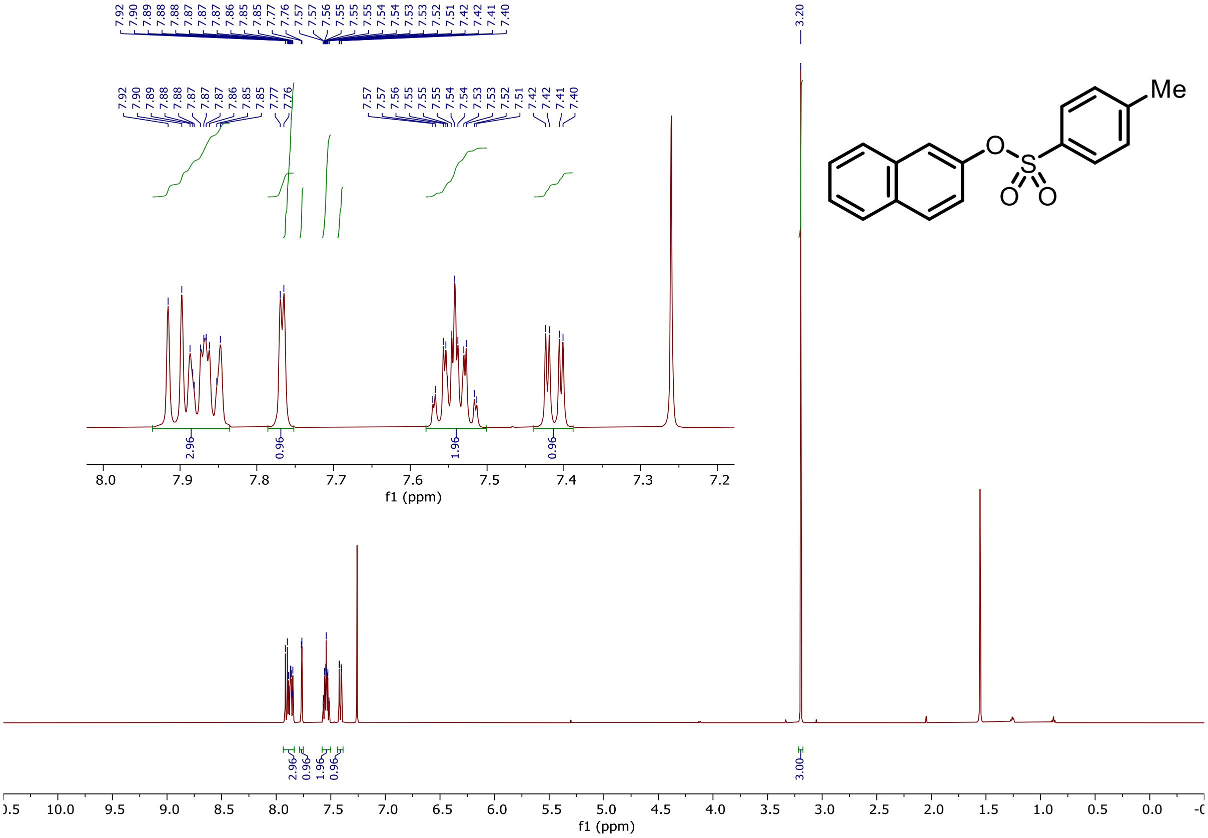


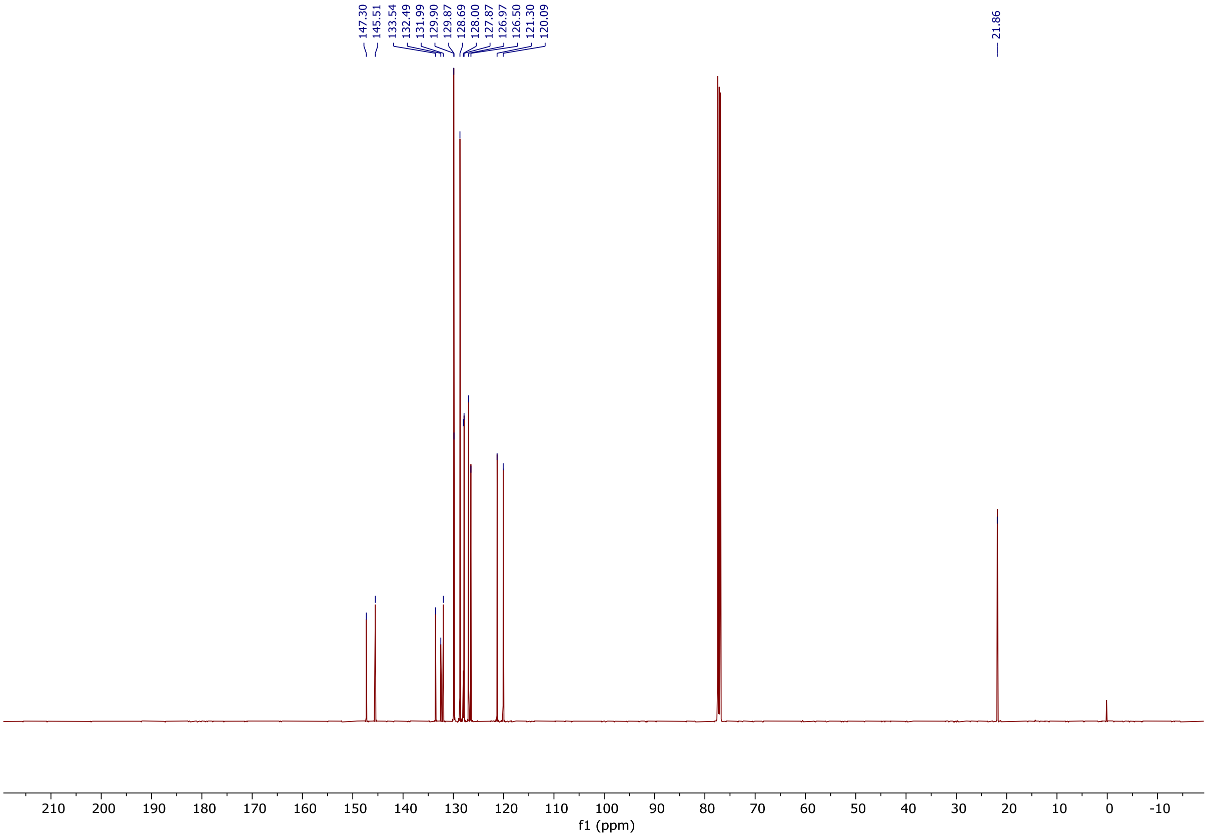

## 2-Naphthyl mesylate (1af)

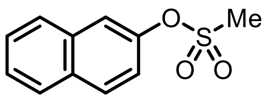


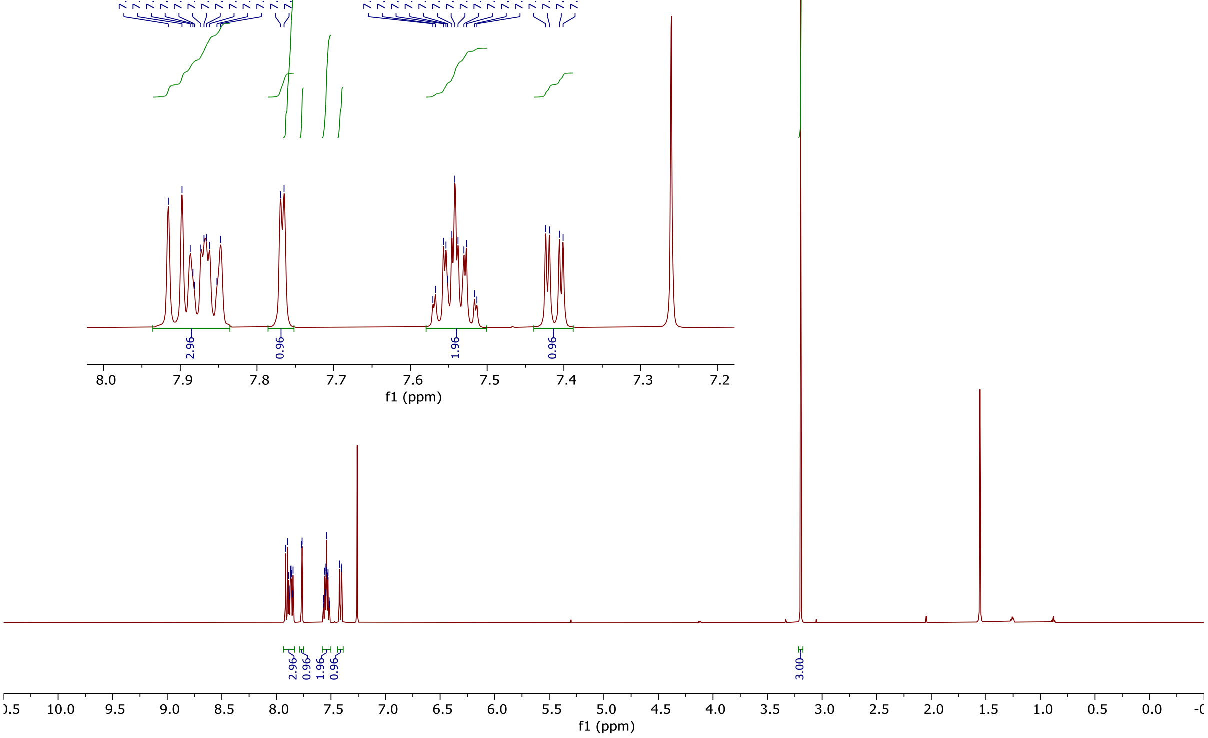


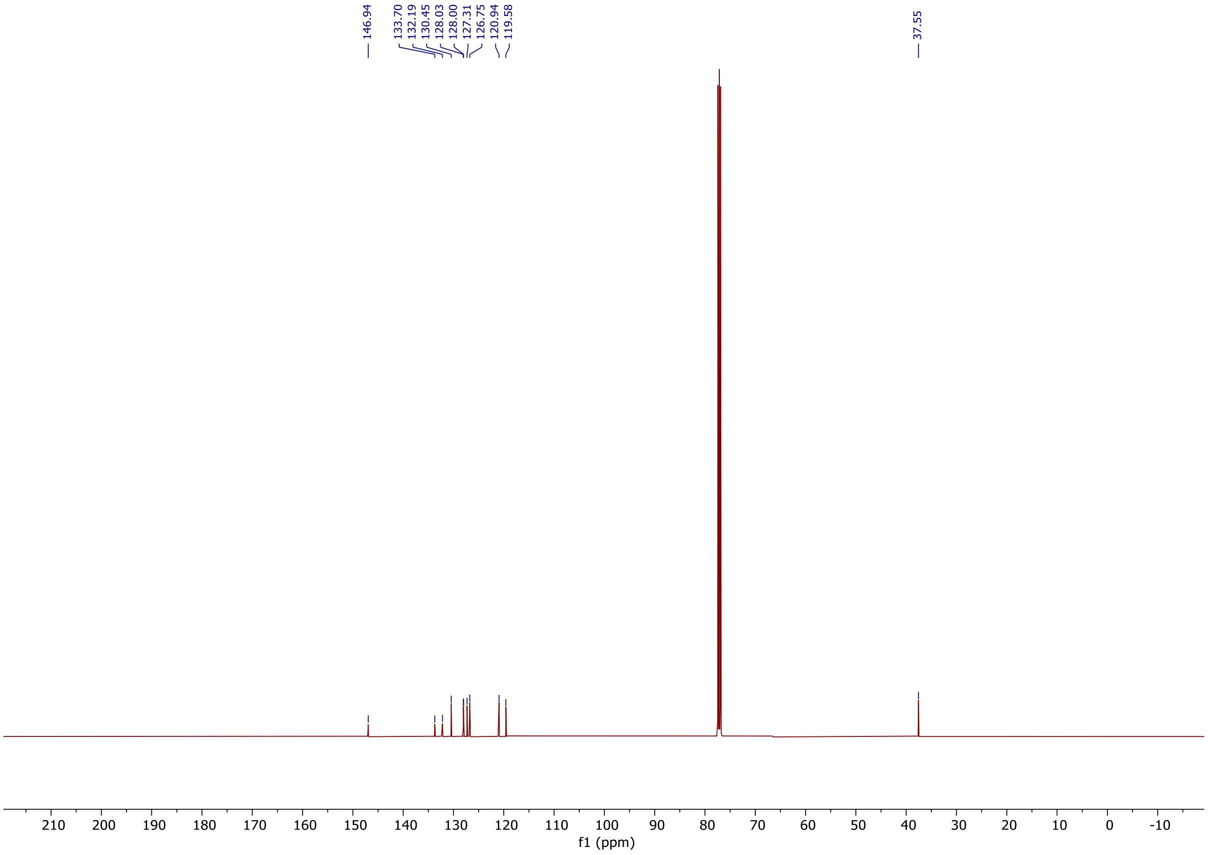

## 2-Naphthyl dimethylsulfamate (1ag)

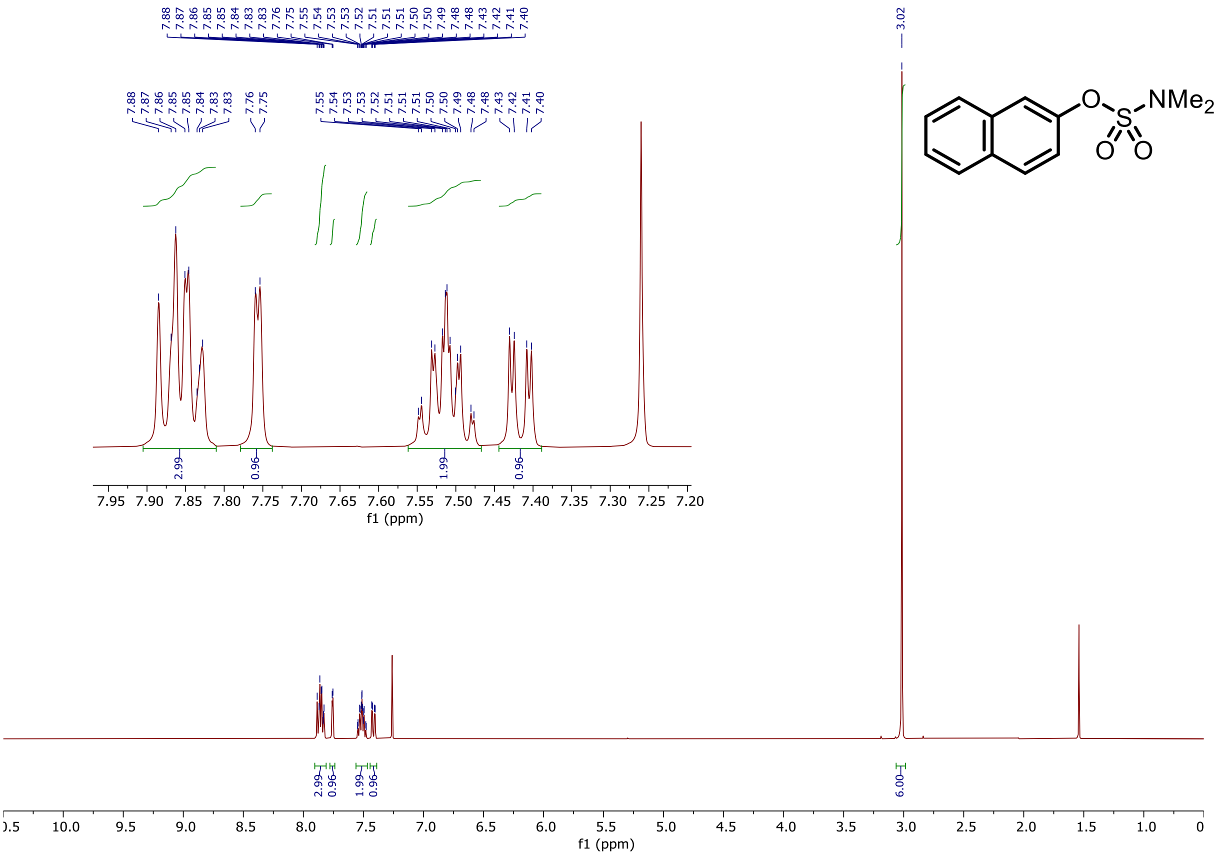

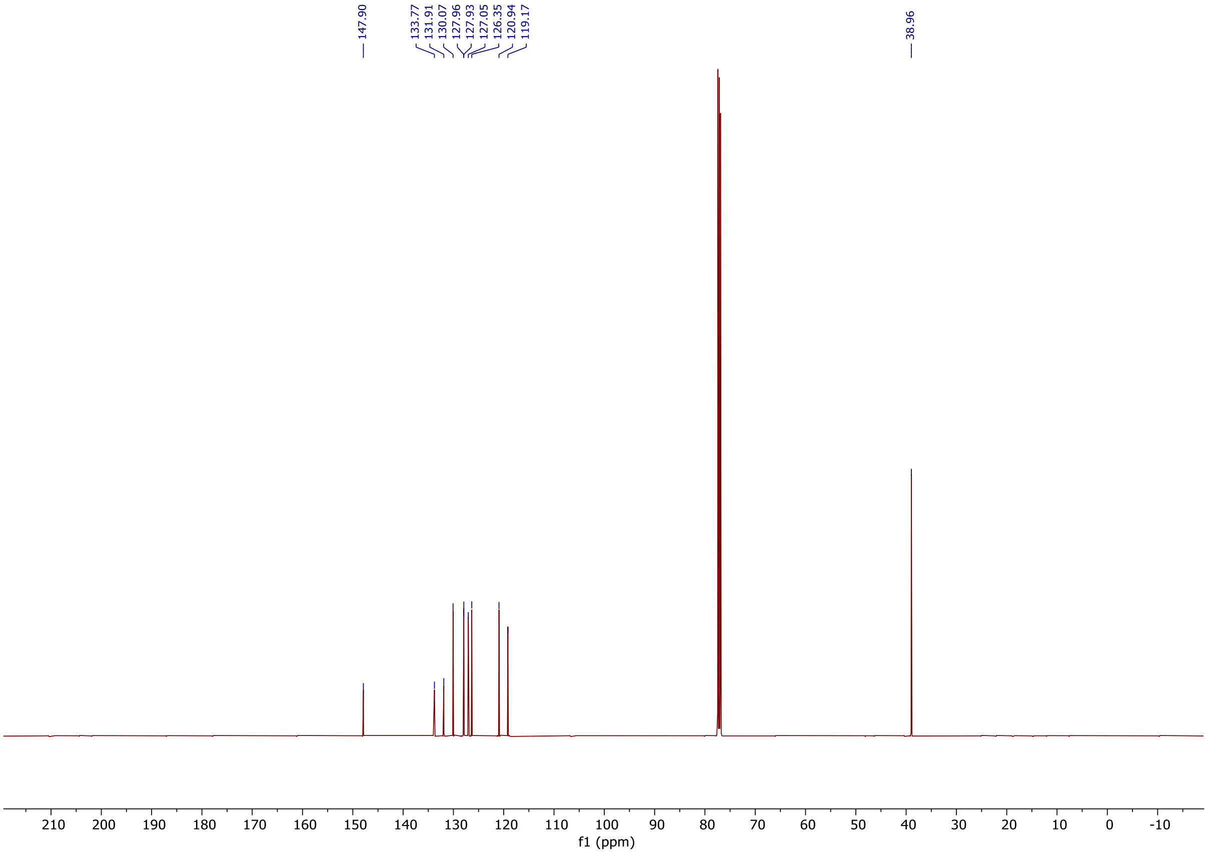

## 1-Naphthyl dimethylsulfamate (1b)

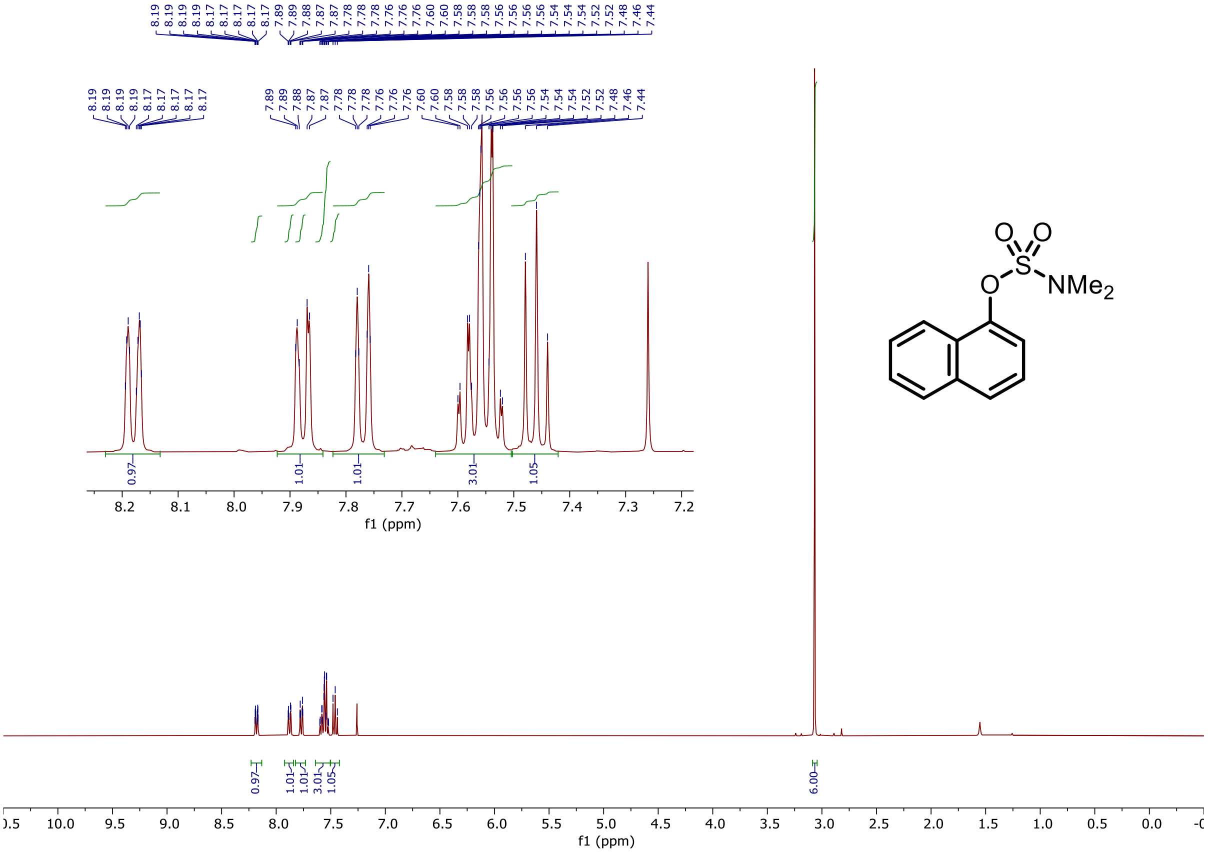


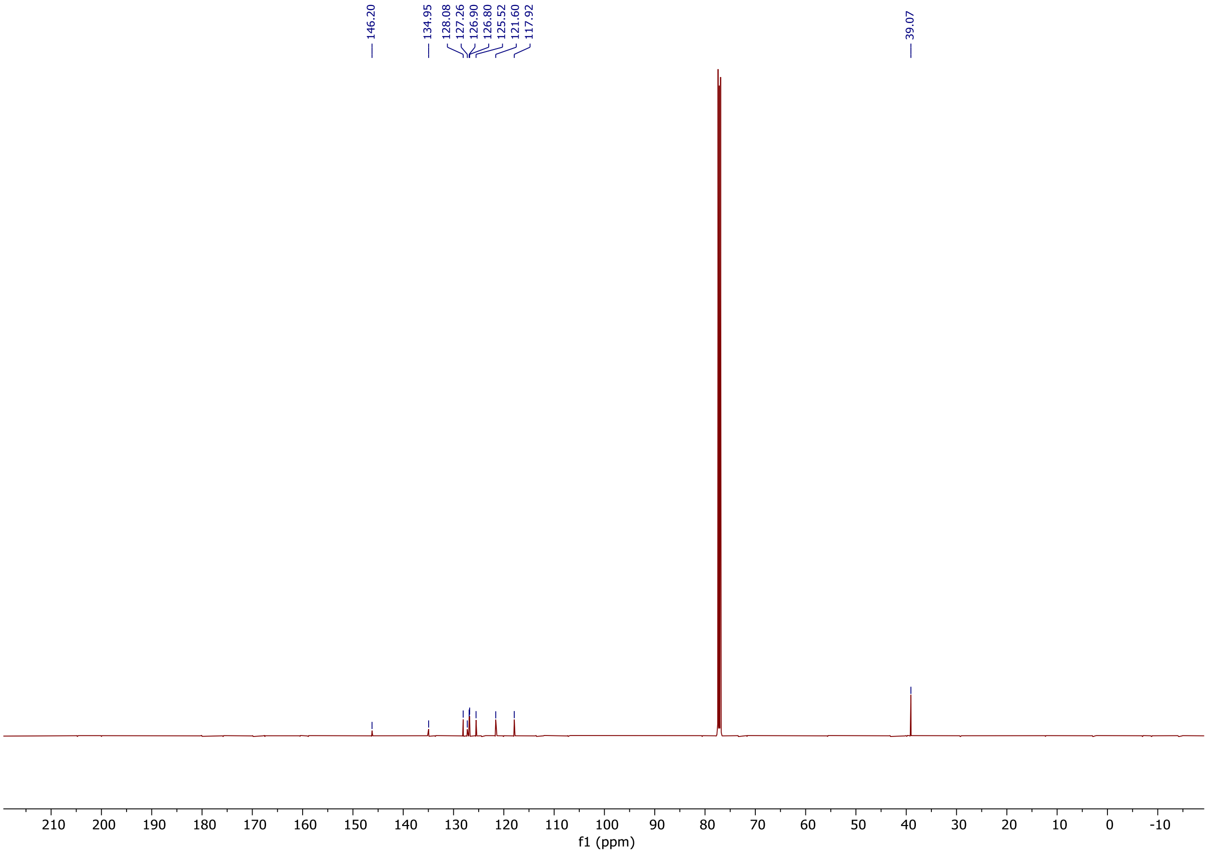

## 4-Phenylphenyl dimethylsulfamate (1c)

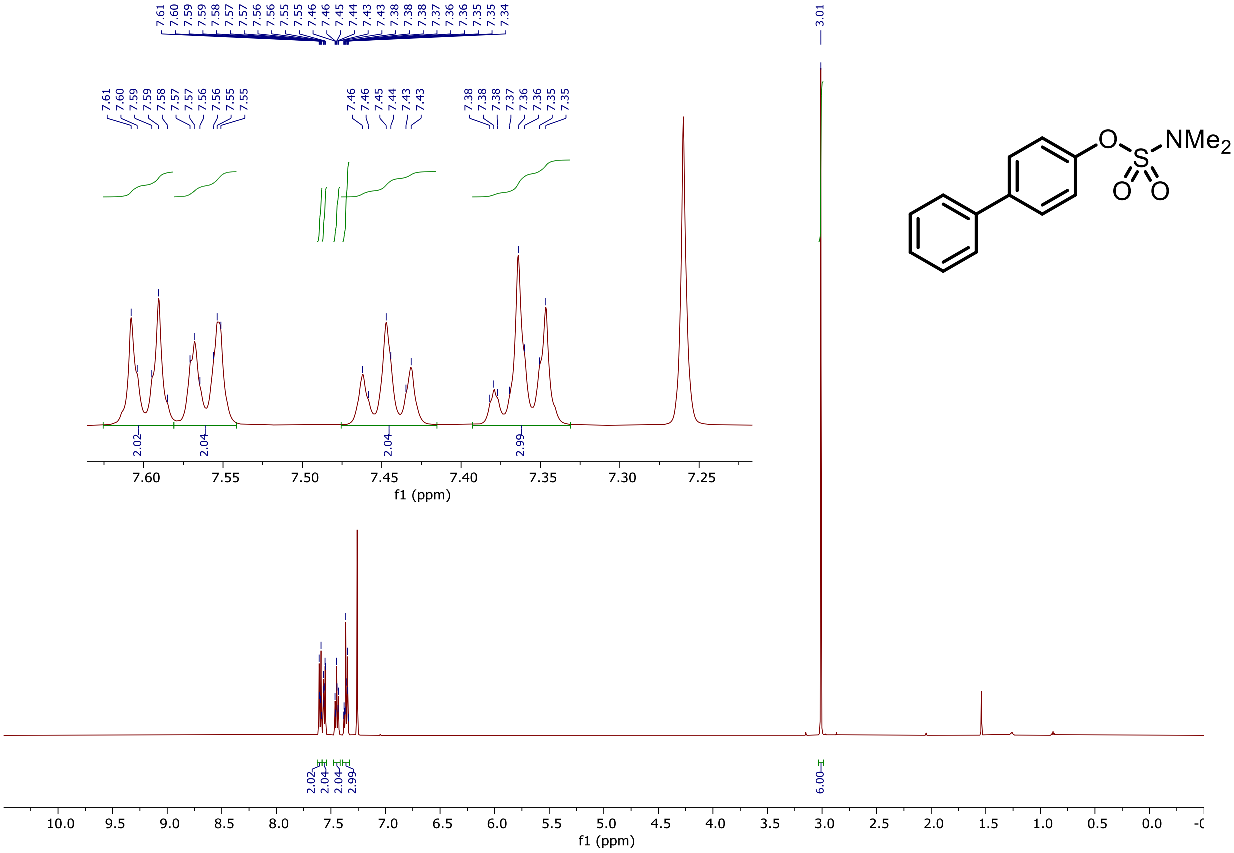


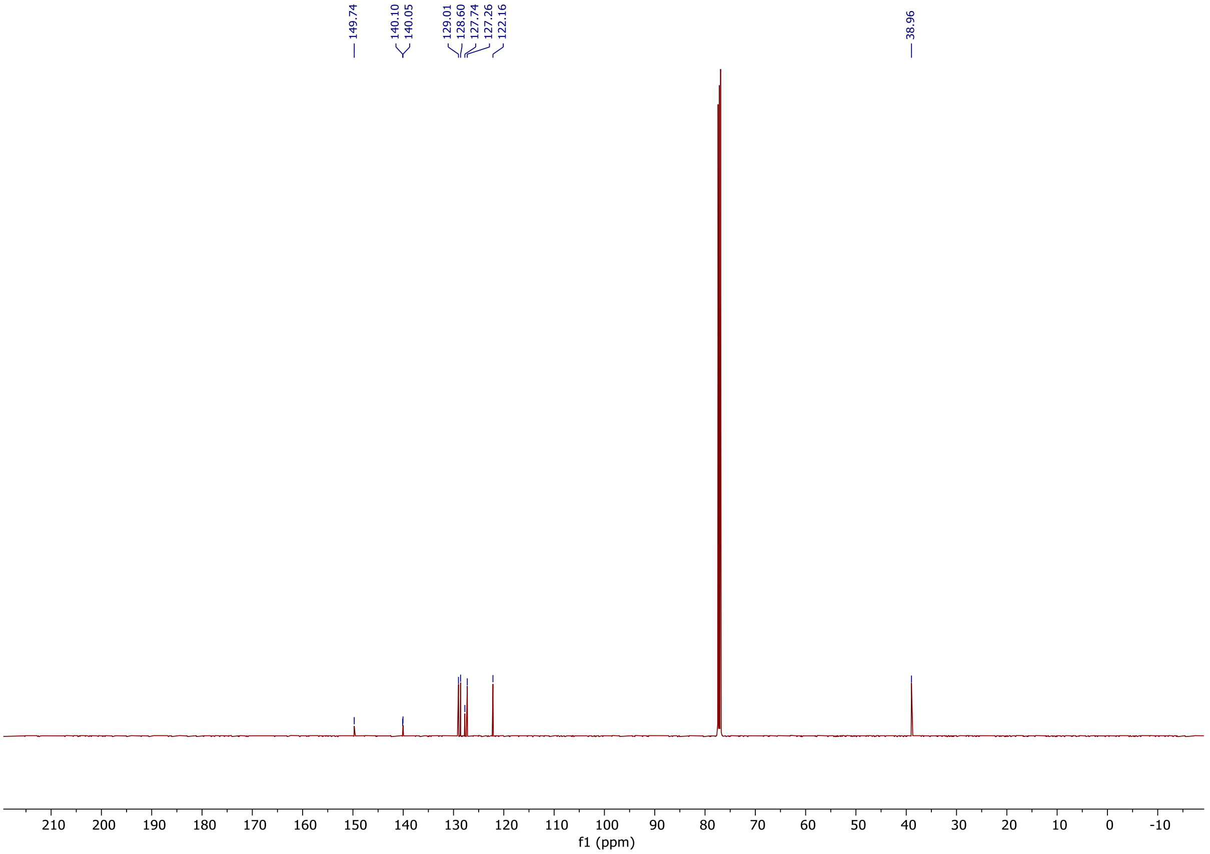

## 4-Methoxy-1-naphthyl dimethylsulfamate (1d)

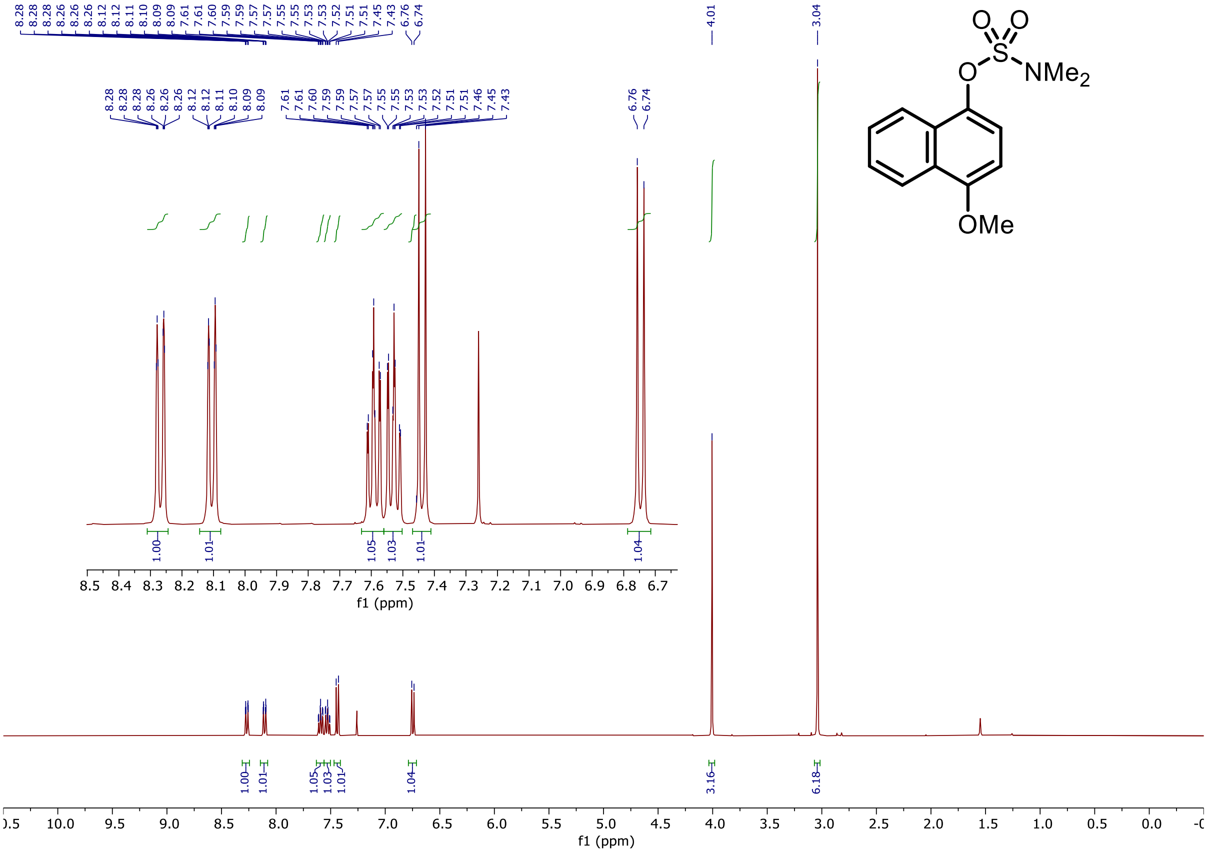


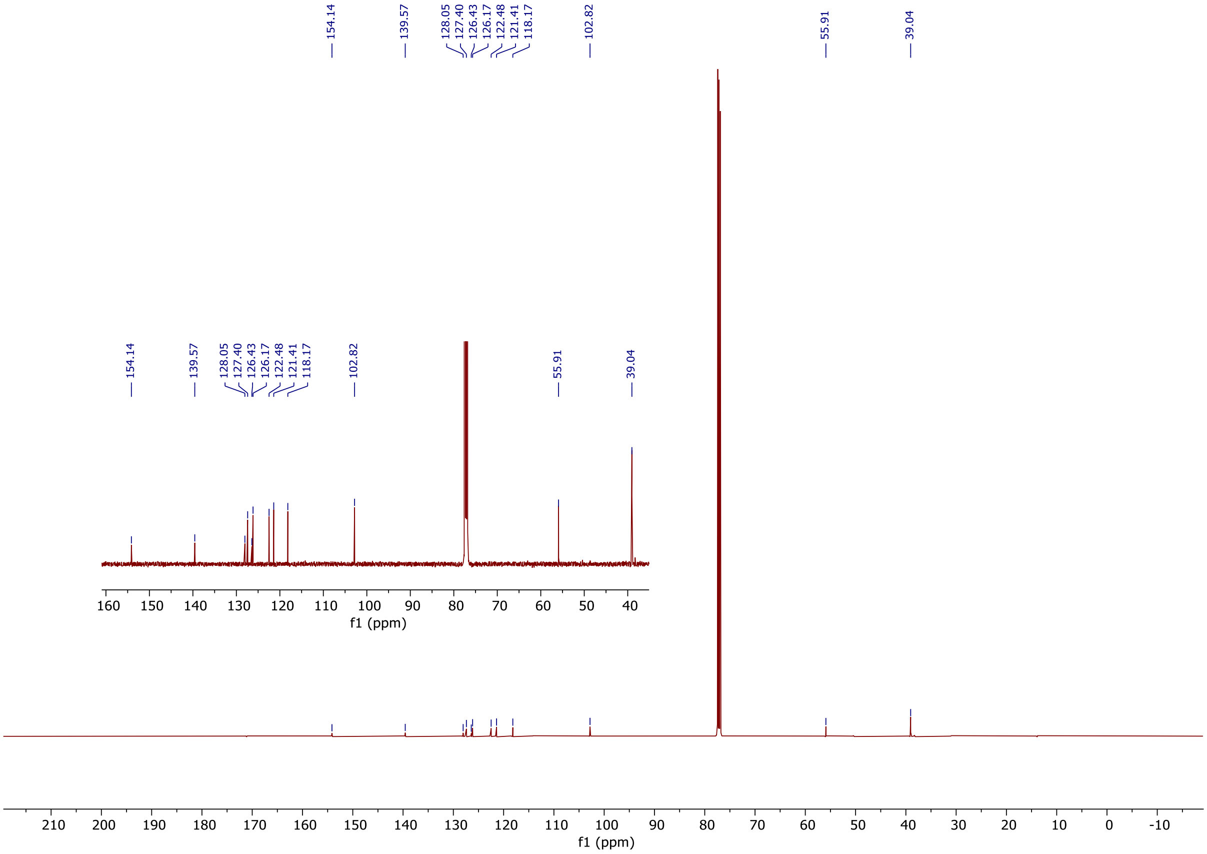

**4-Methoxyphenyl dimethylsulfamate (1e)**

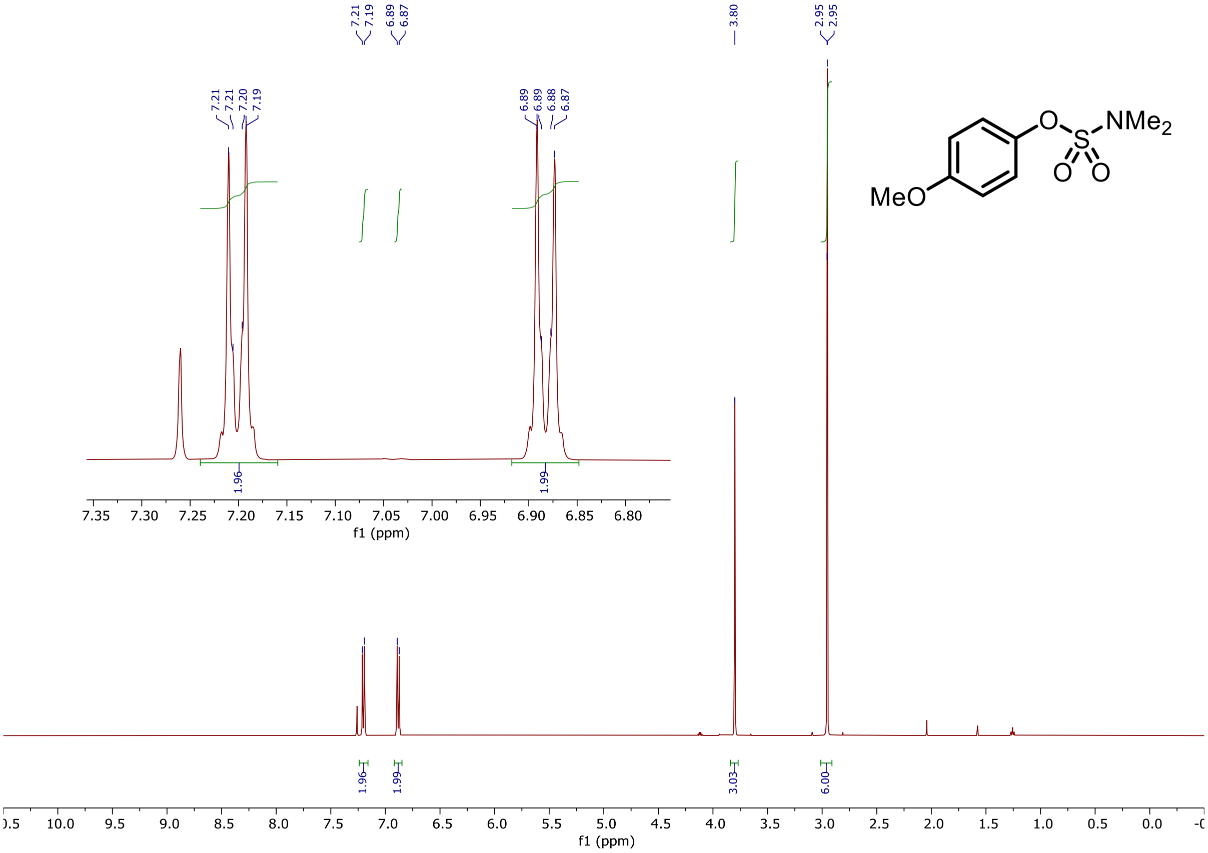


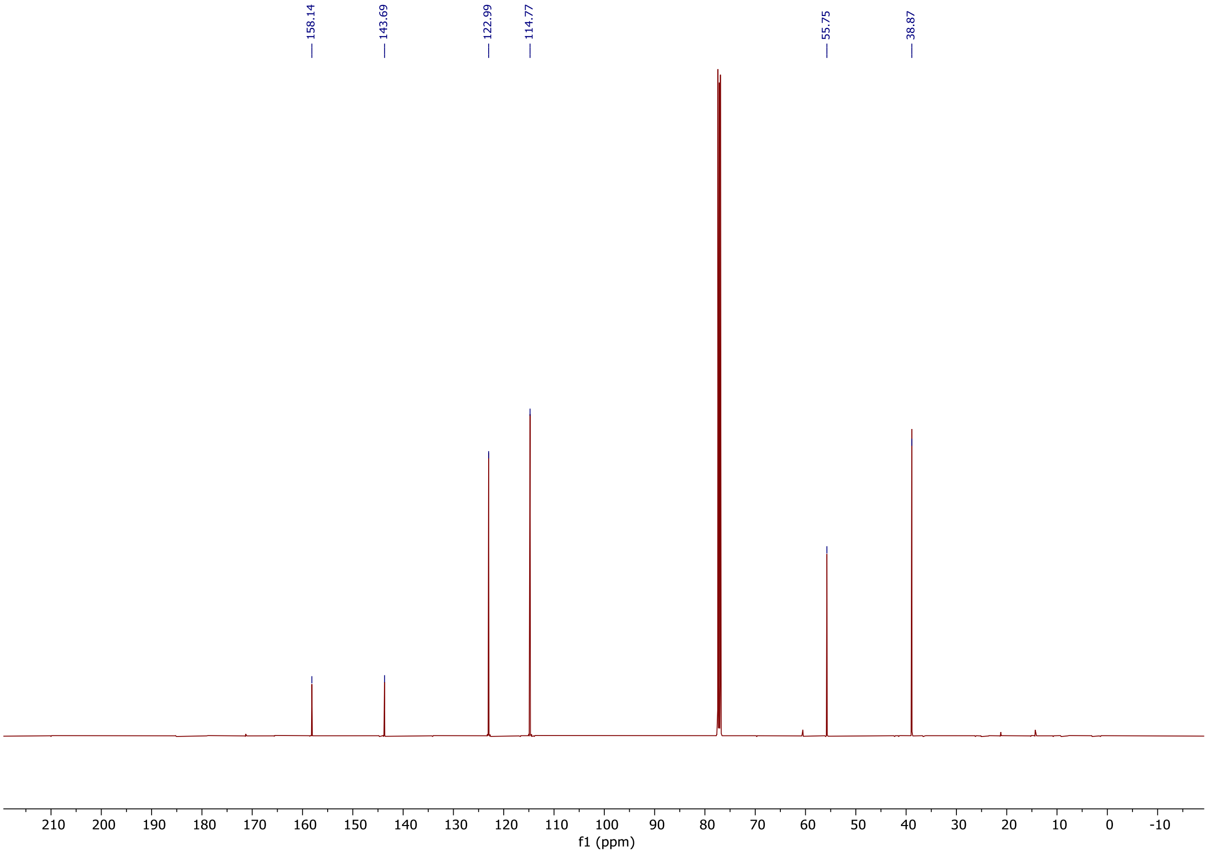

## 4-Cyanophenyl dimethylsulfamate (1f)

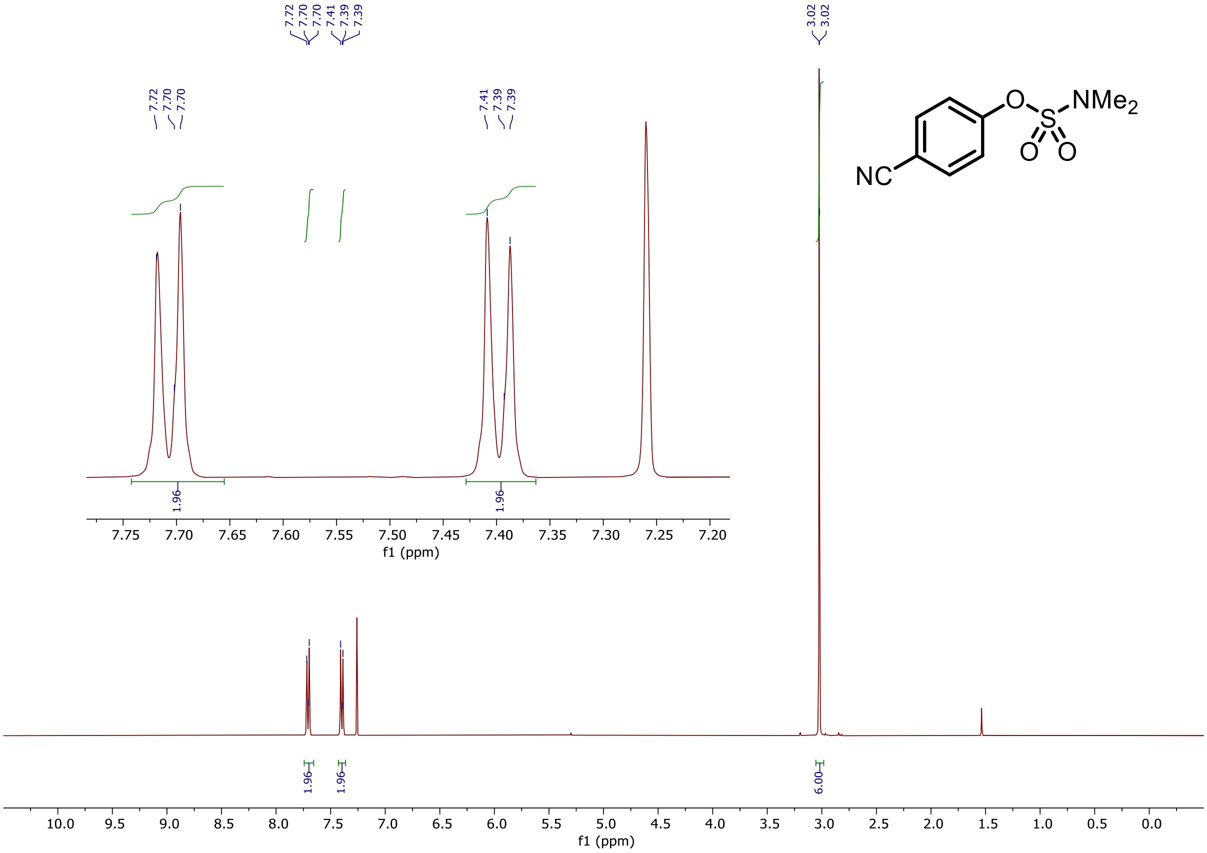


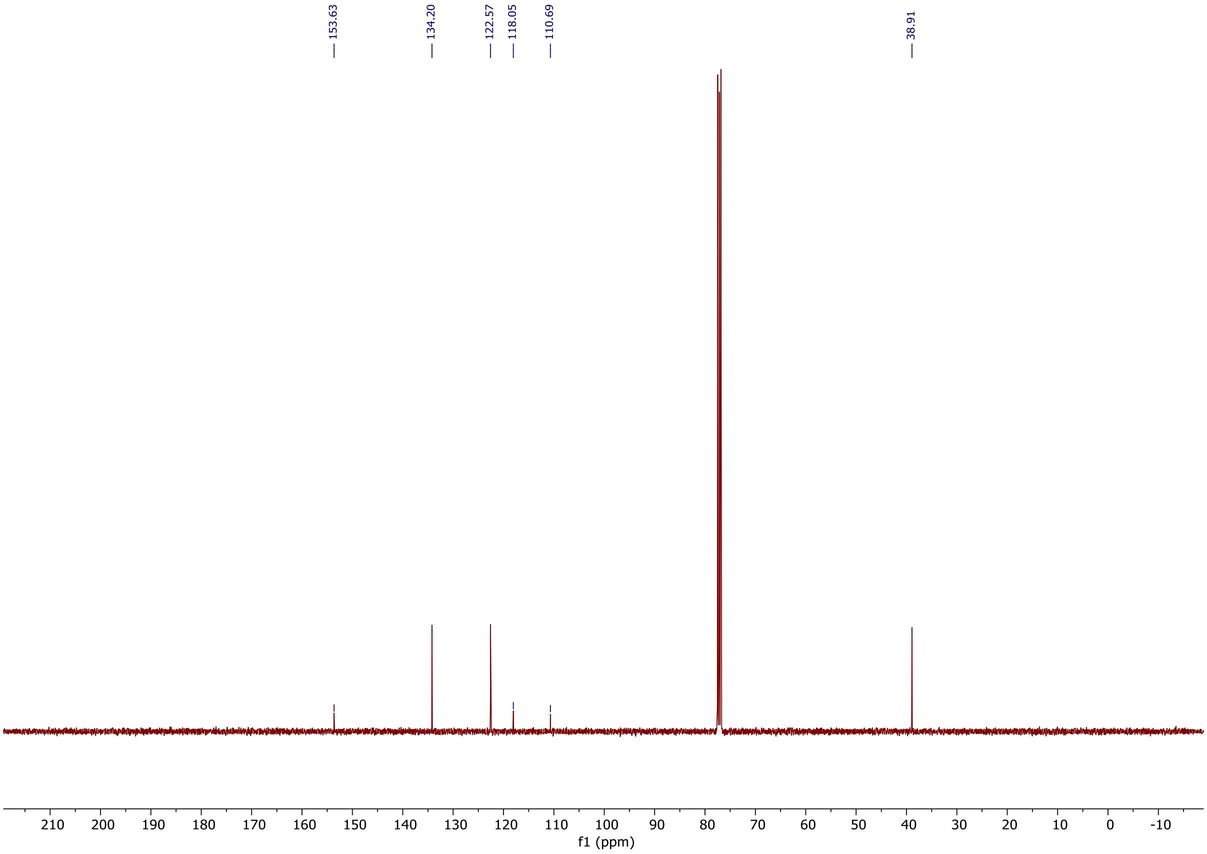

**Methyl-4-hydroxylbenzyl dimethylsulfamate (1g)**

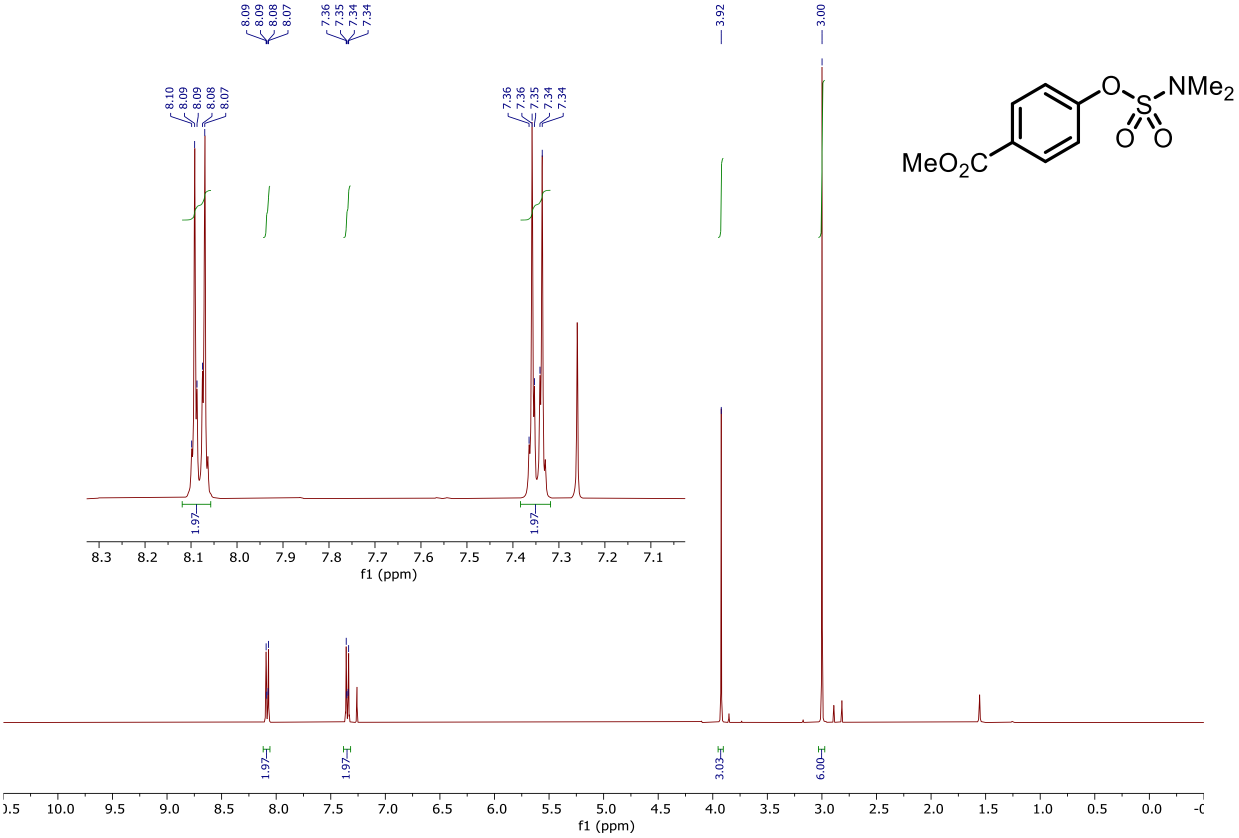


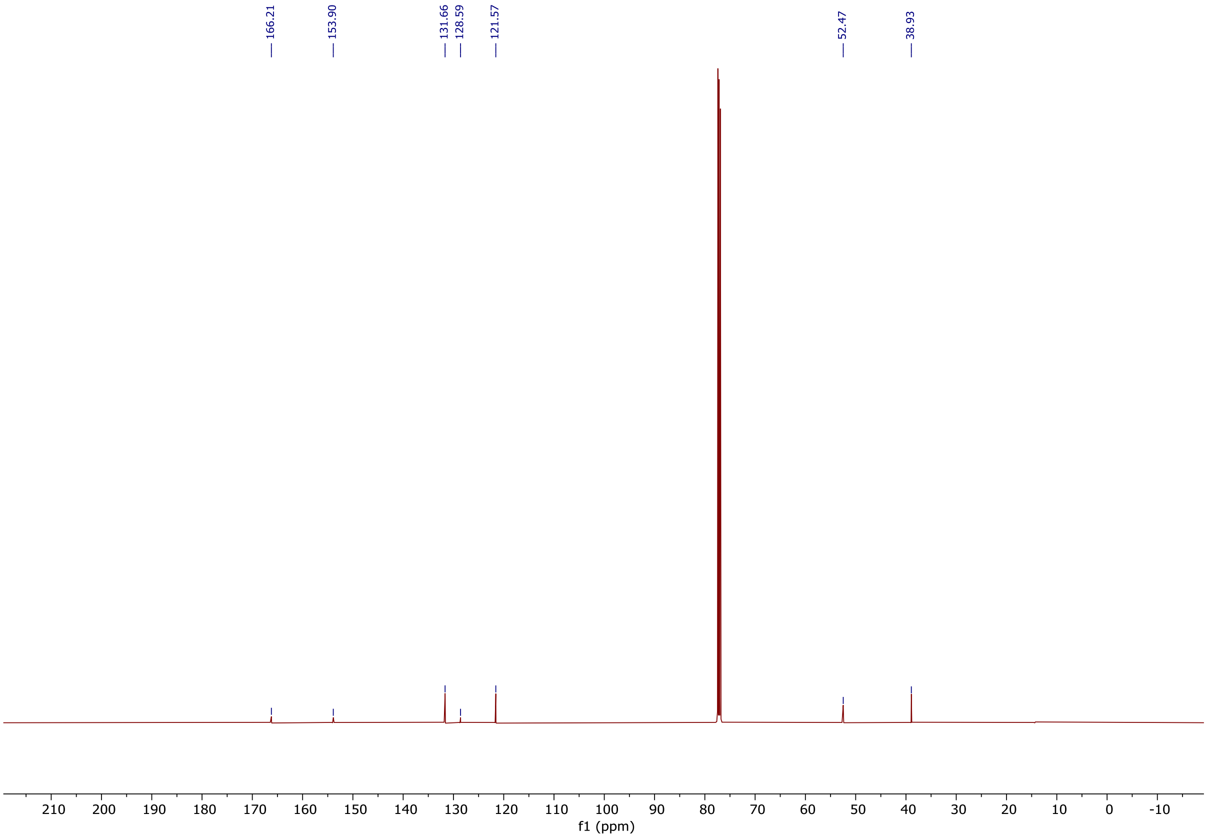

## pyren-1-yl dimethylsulfamate (1j)

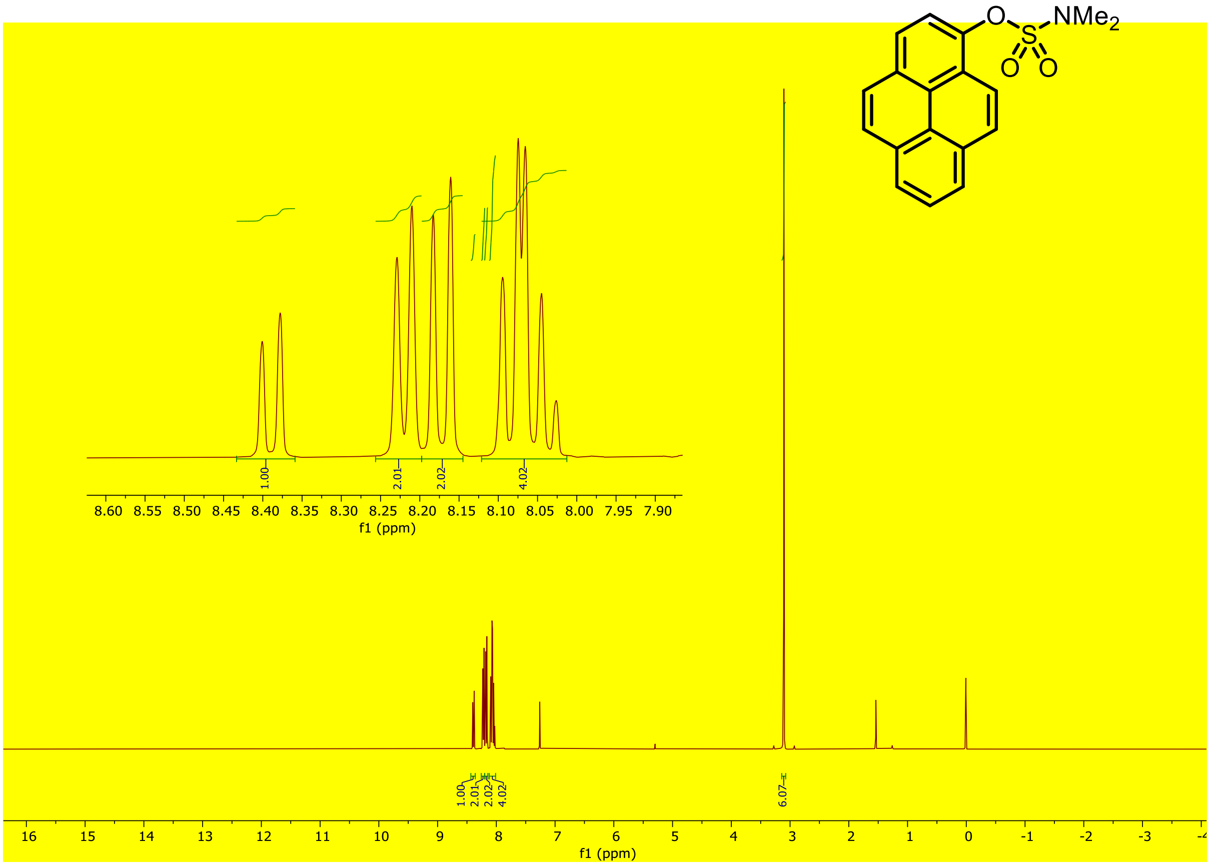


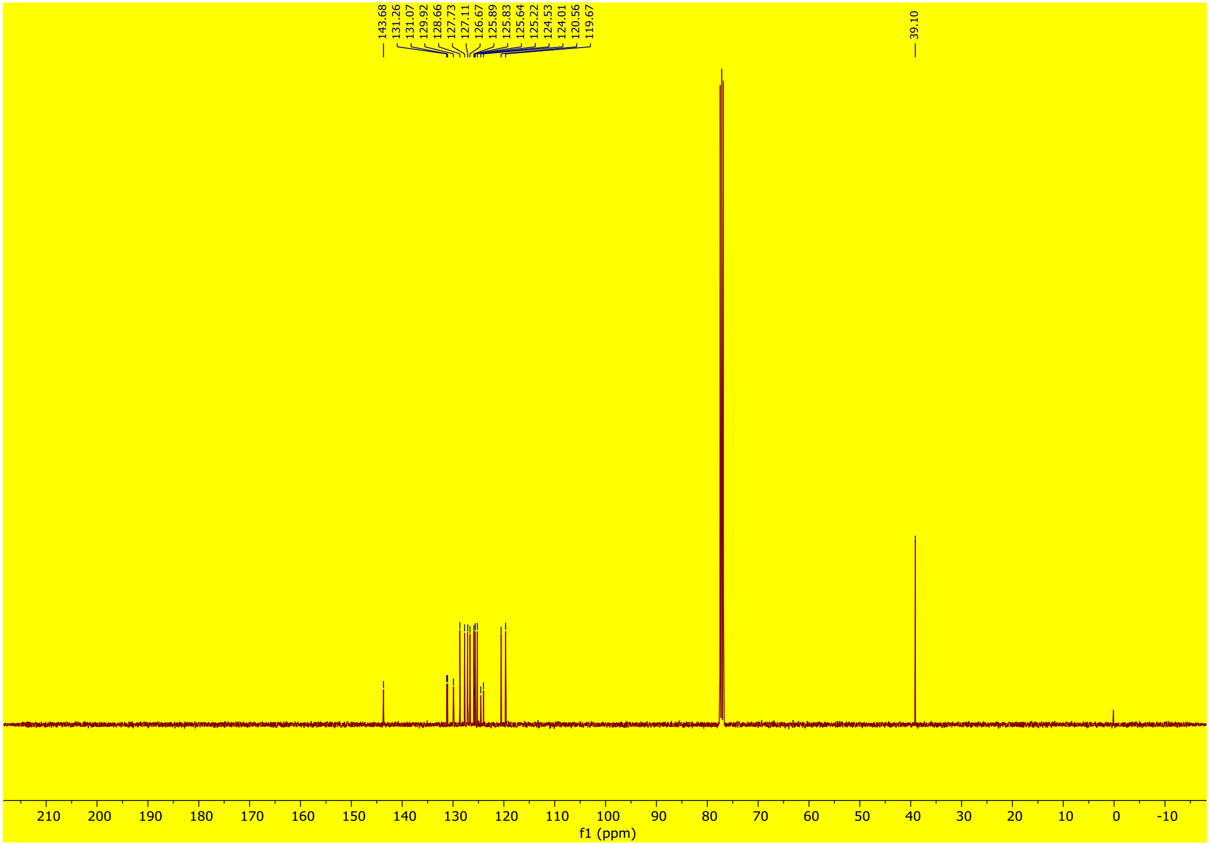

## 3-hydroxylpyridyl dimethylsulfamate (1h)

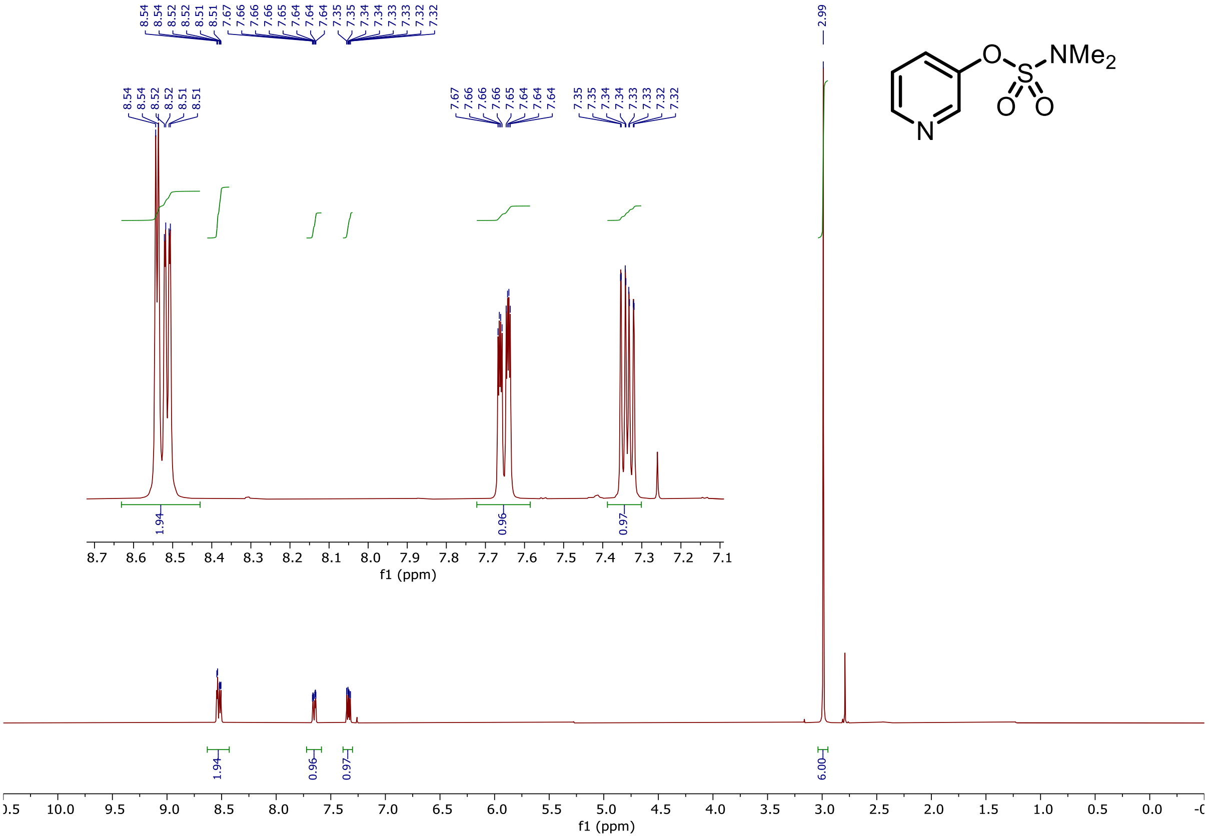

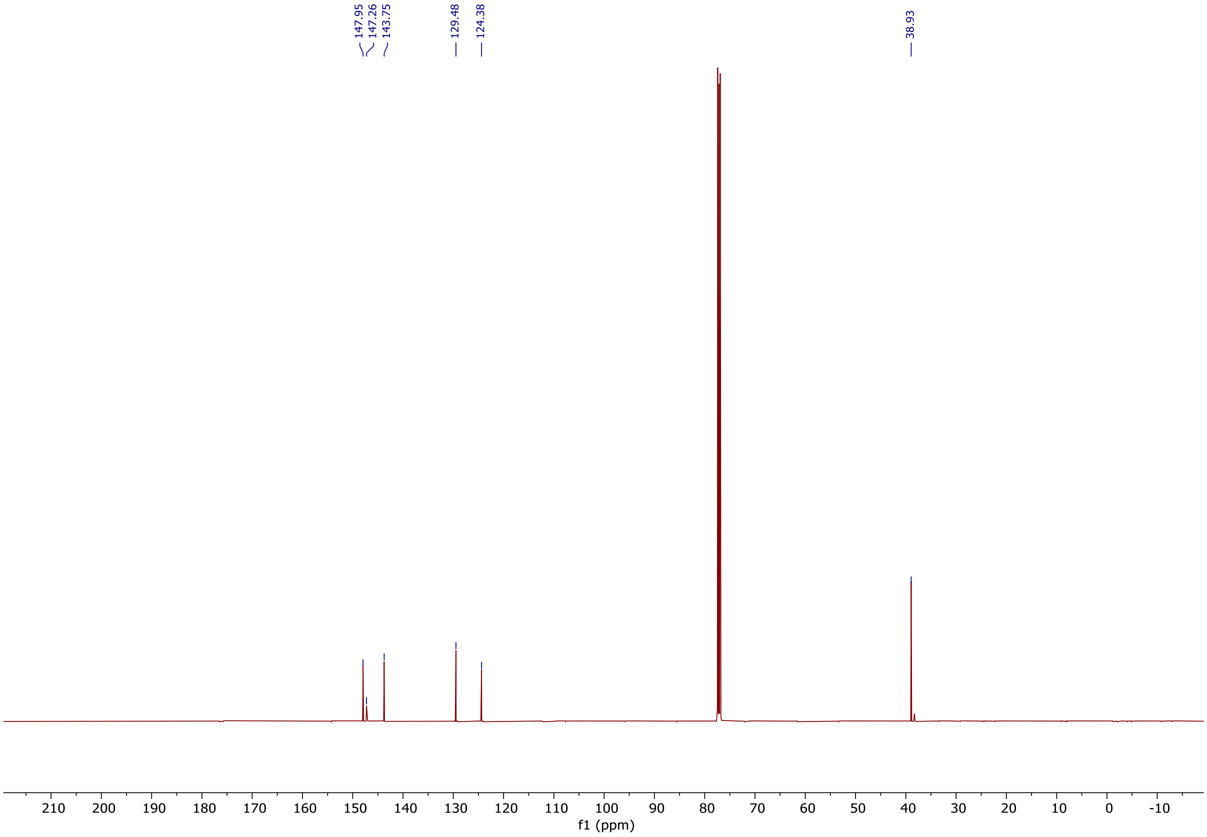


**2-hydroxylpyridyl dimethylsulfamate (1i)**

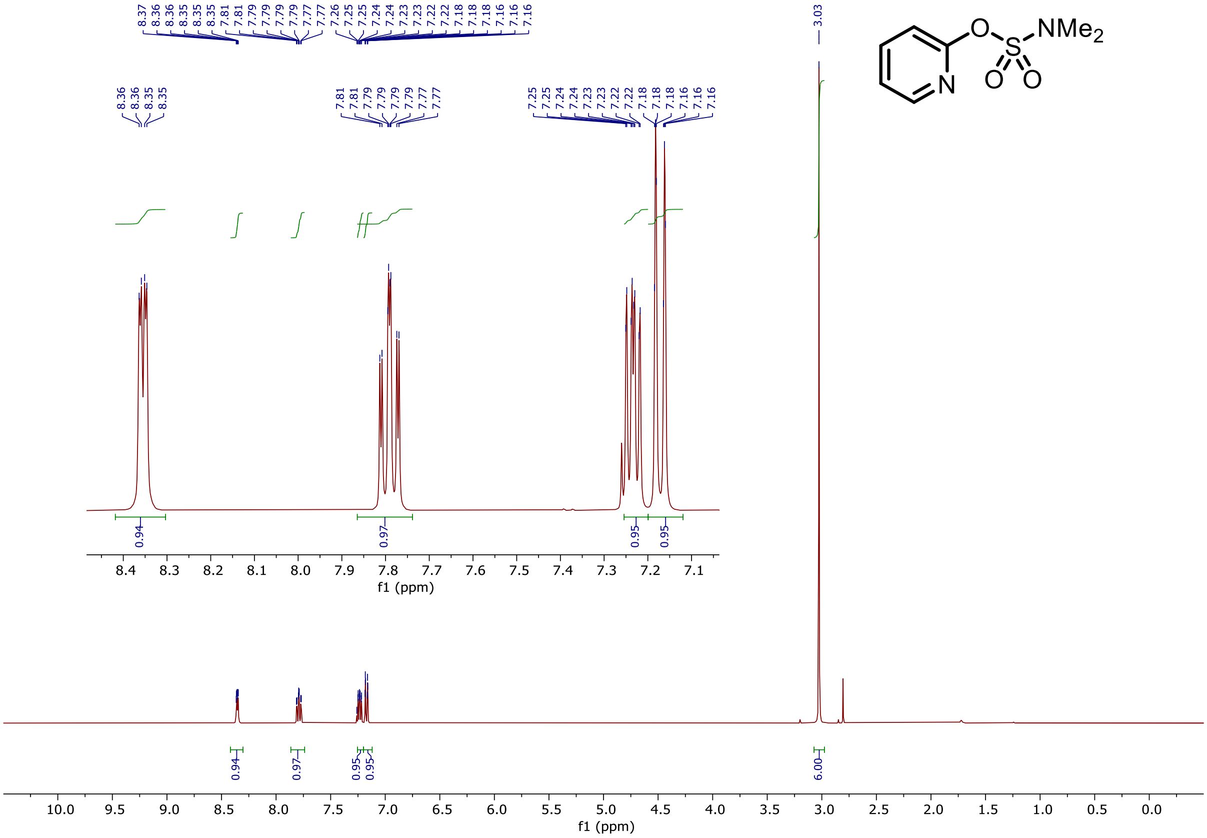

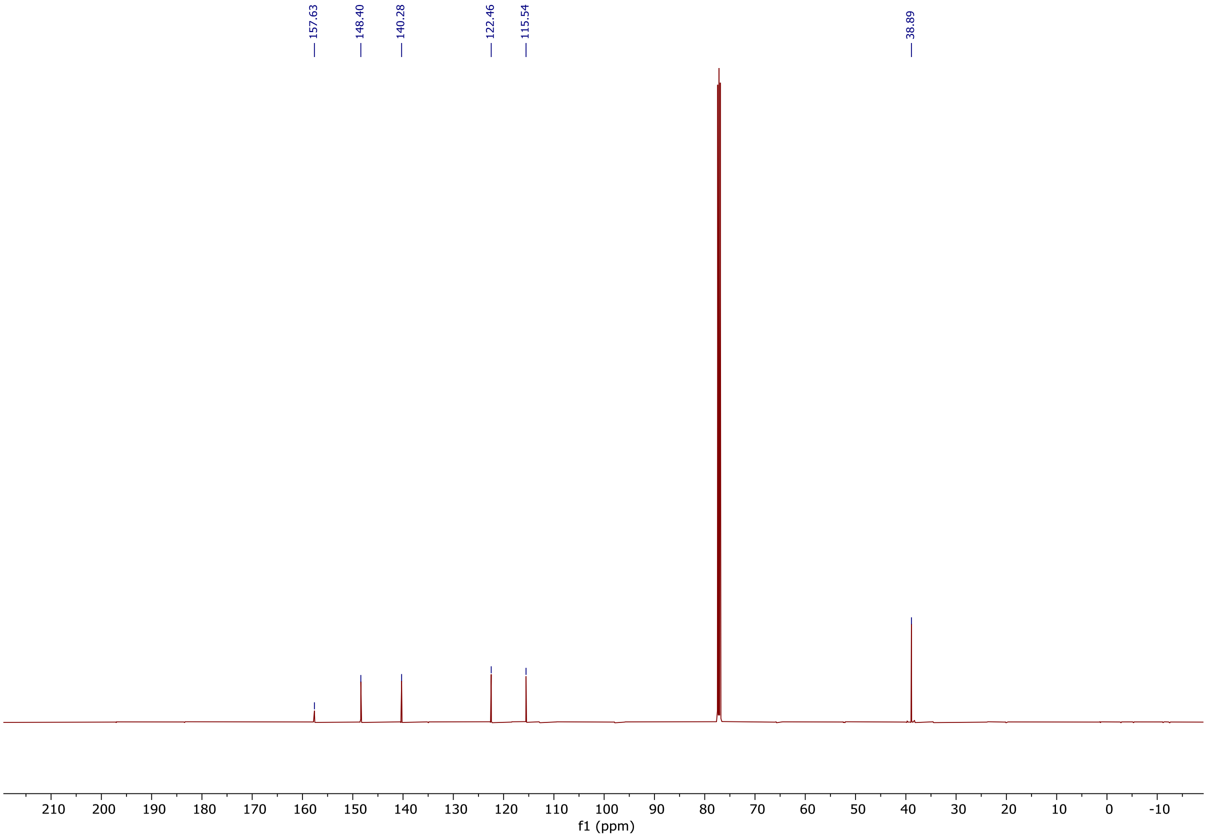


**2-(4-fluorophenyl) naphthalene (3a)**

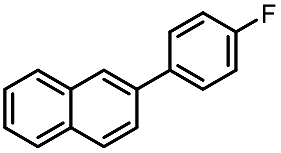

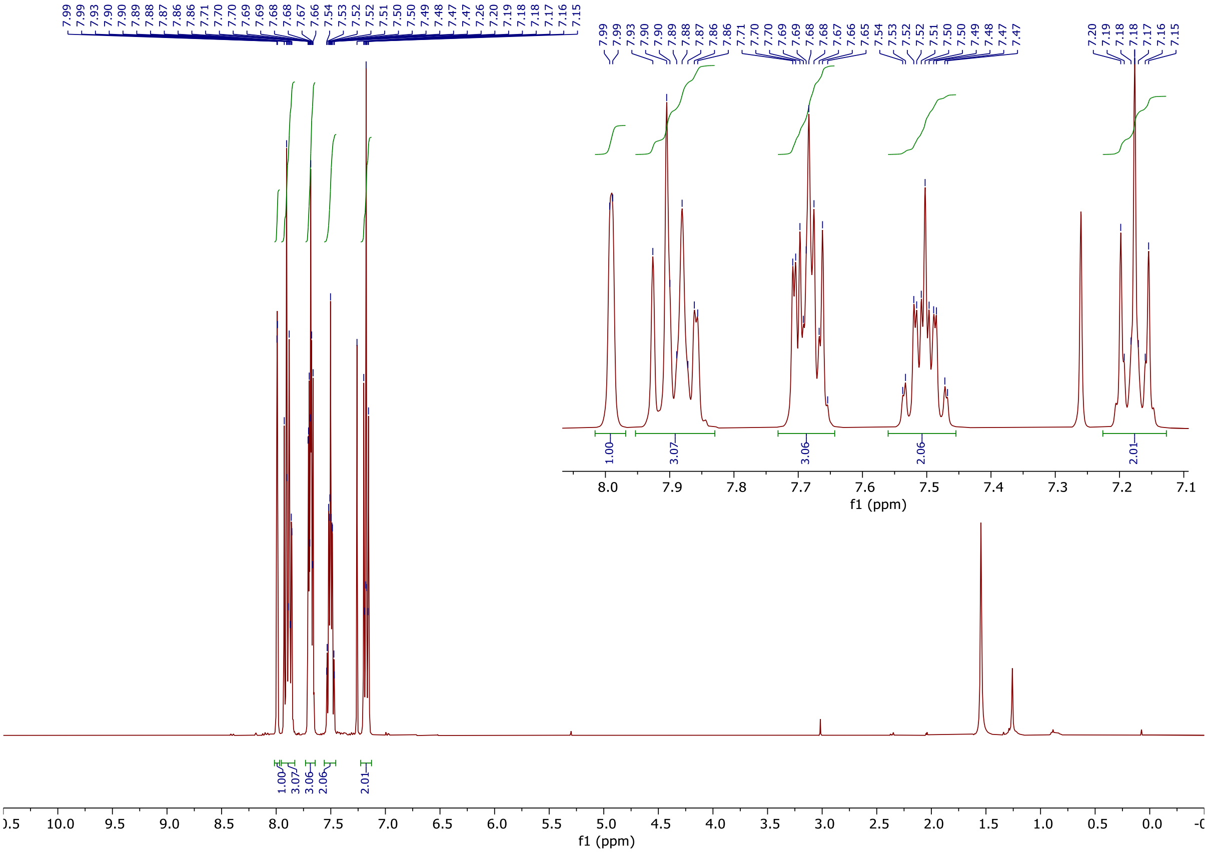


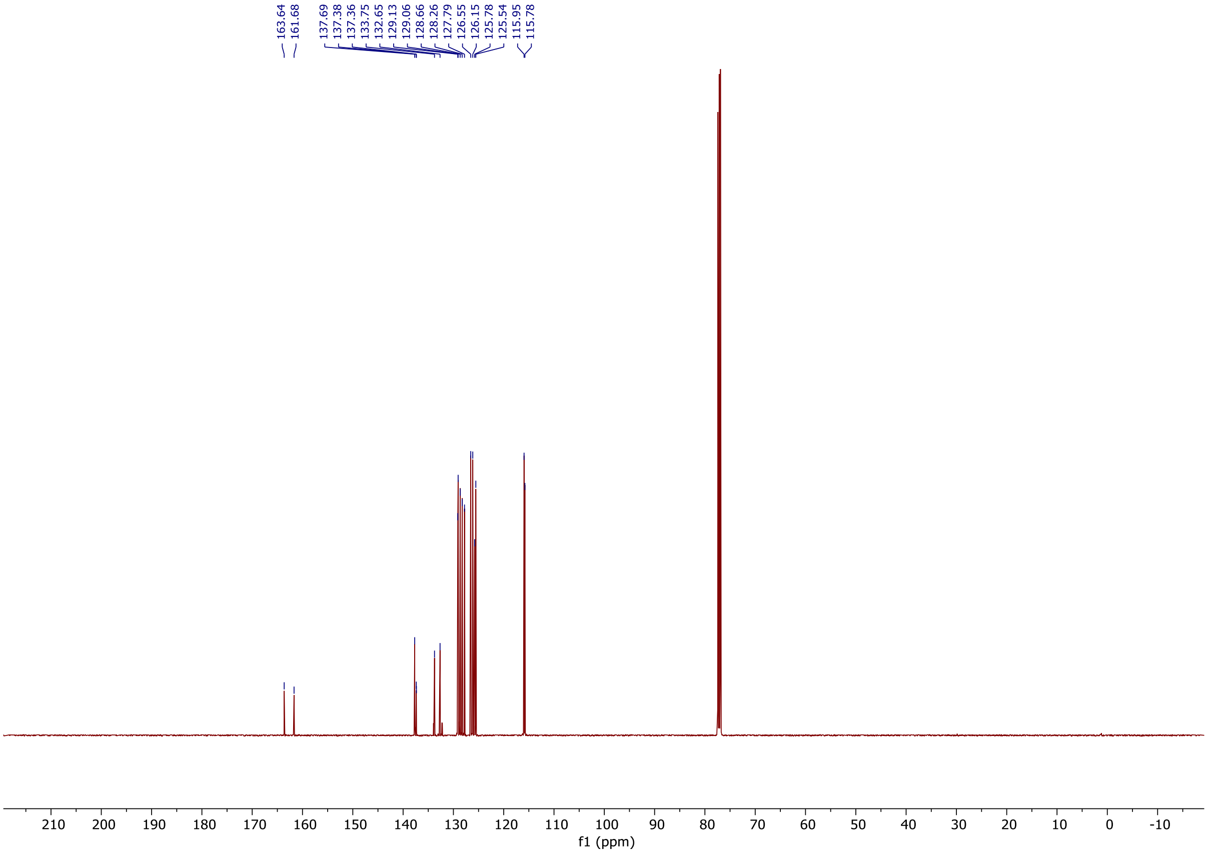


**2-phenyl naphthalene (3b)**

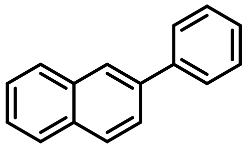

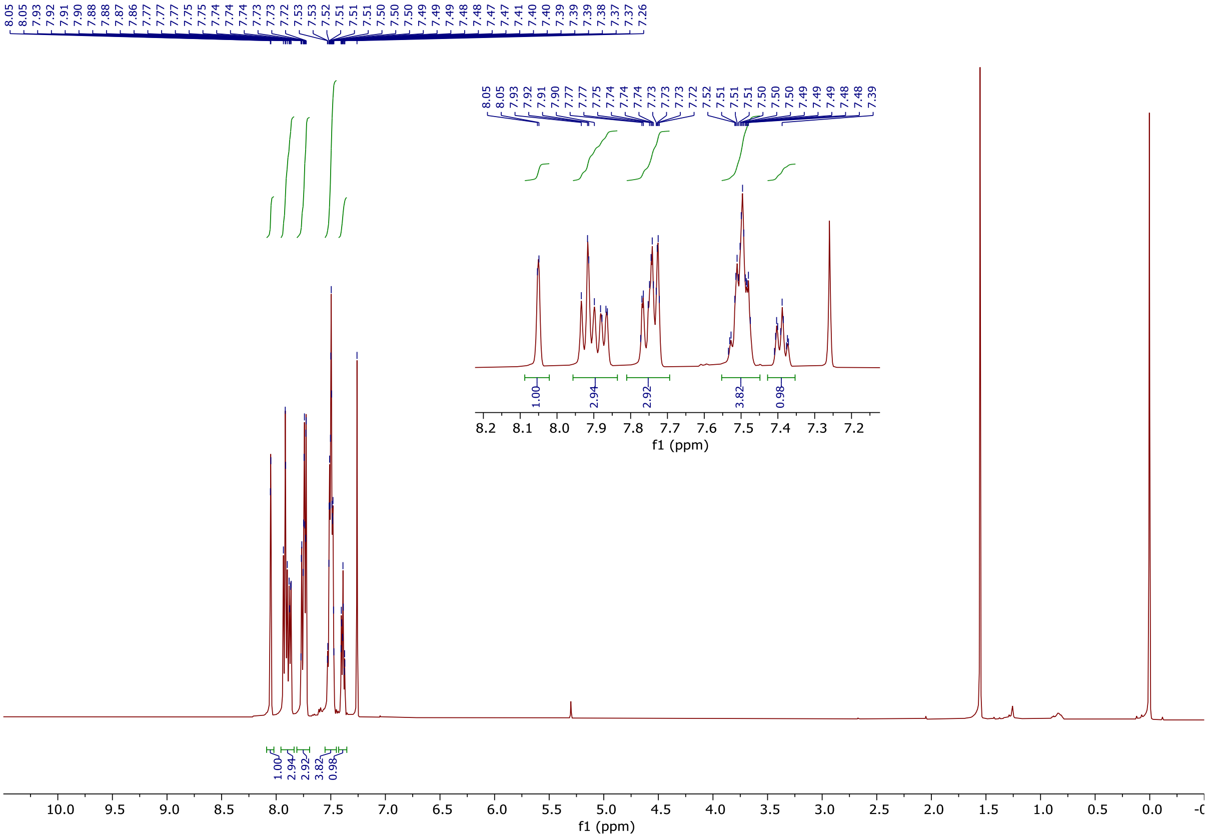


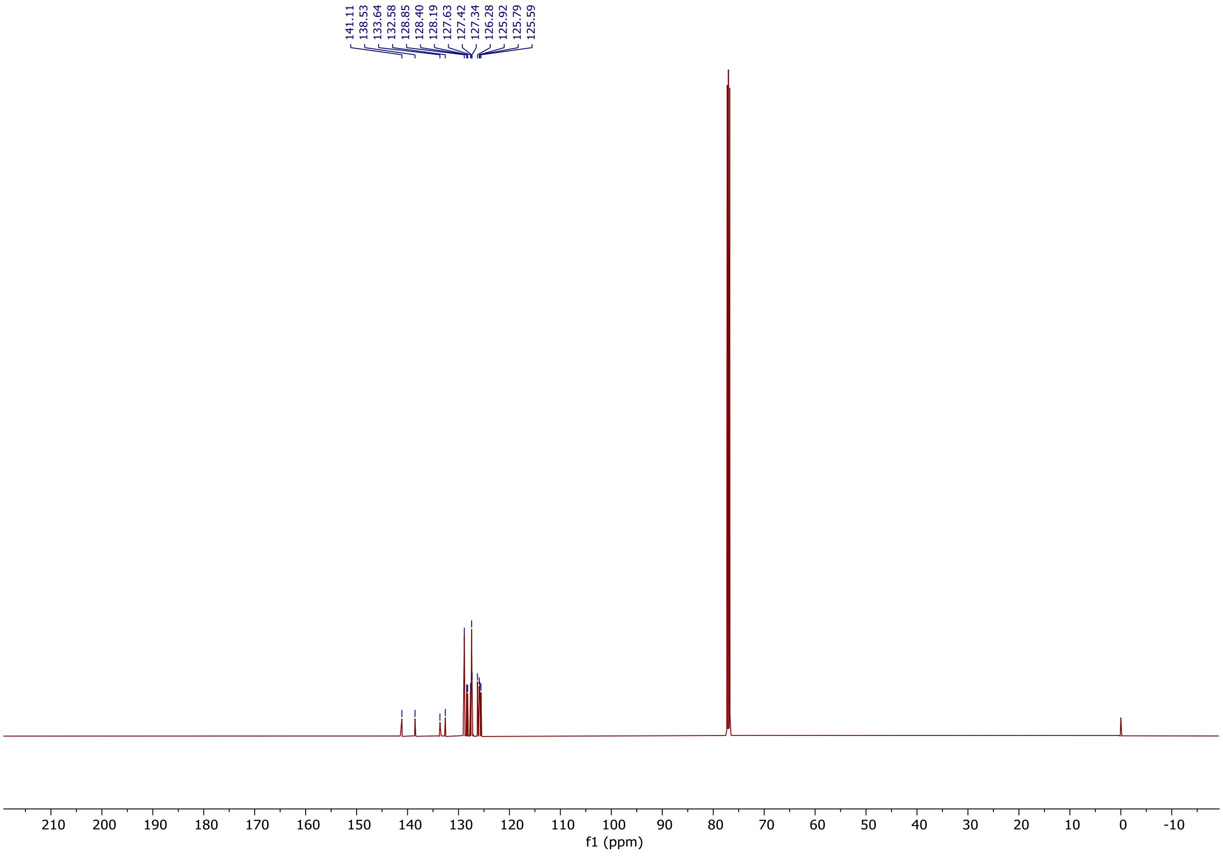


**2-(4-methoxyphenyl) naphthalene (3c)**

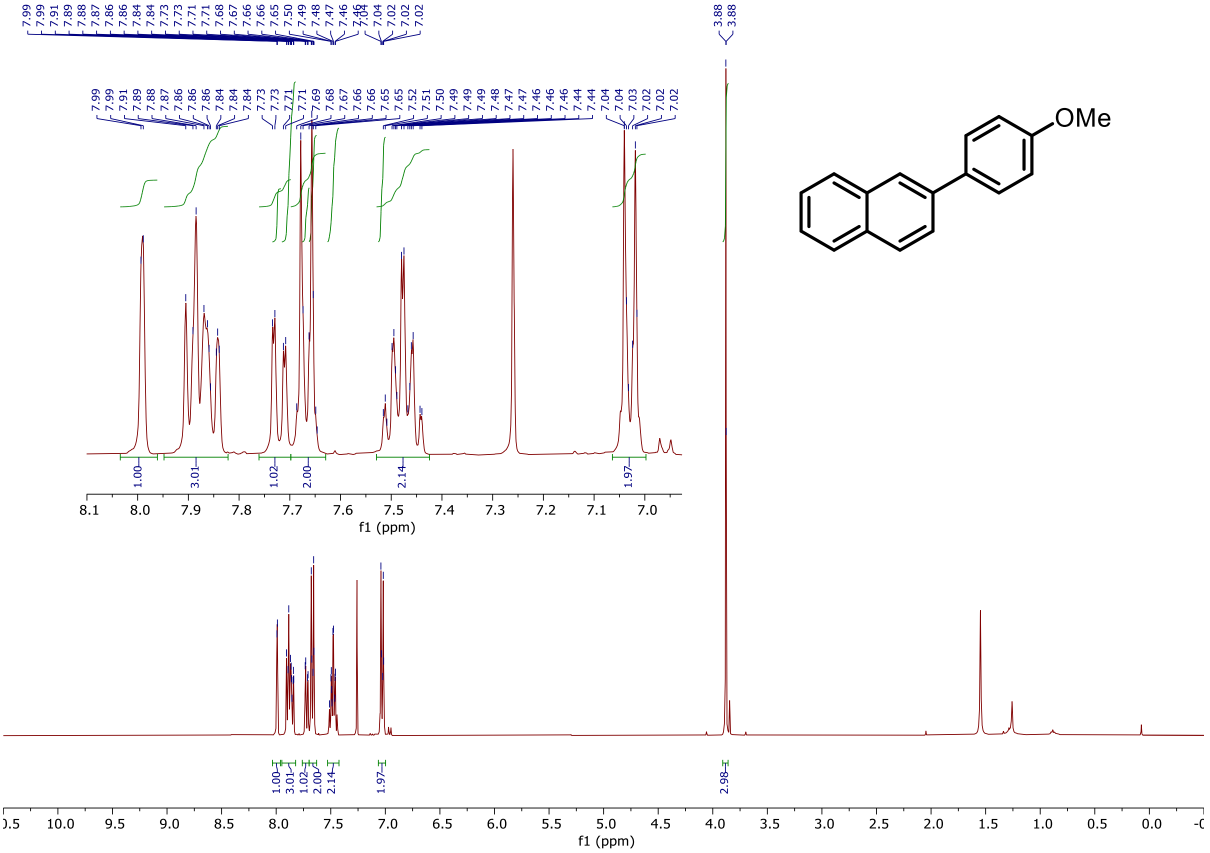


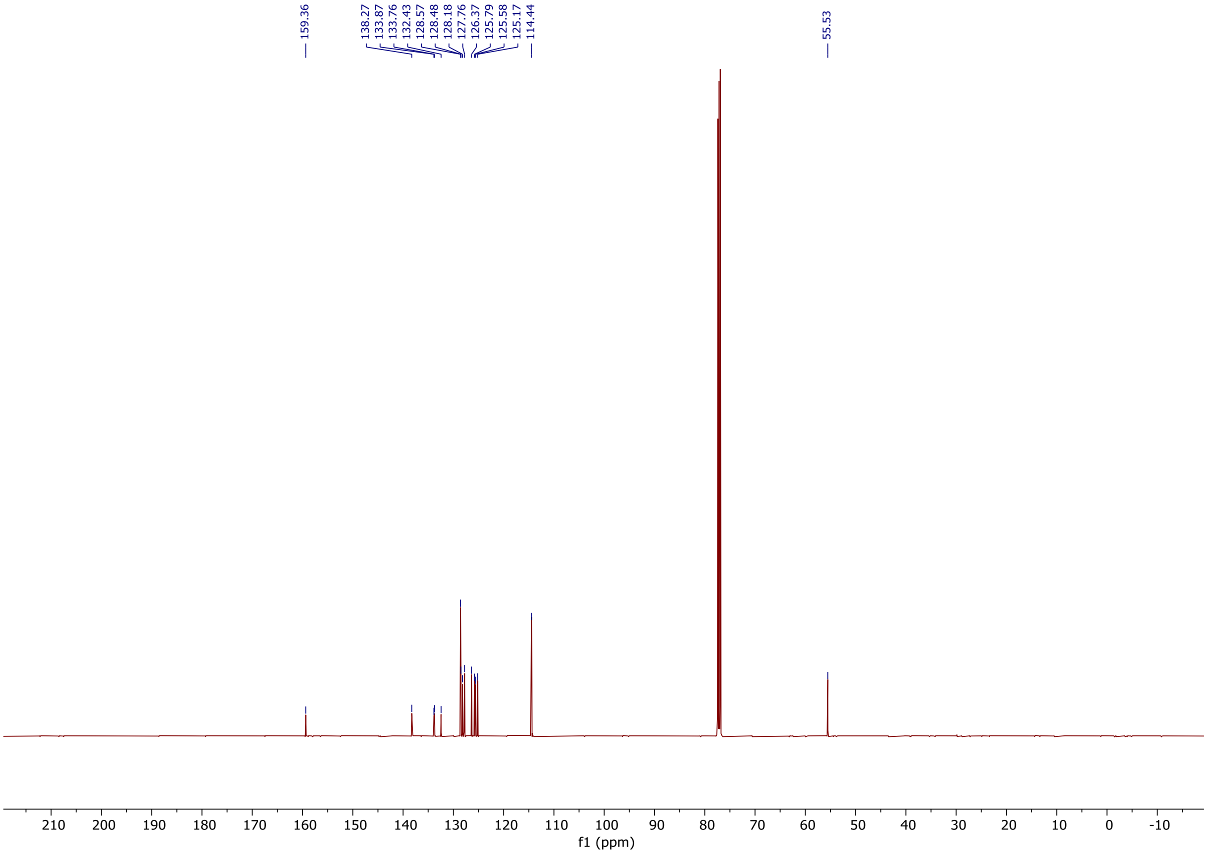


**2-(4-isopropoxyphenyl)naphthalene (3f)**

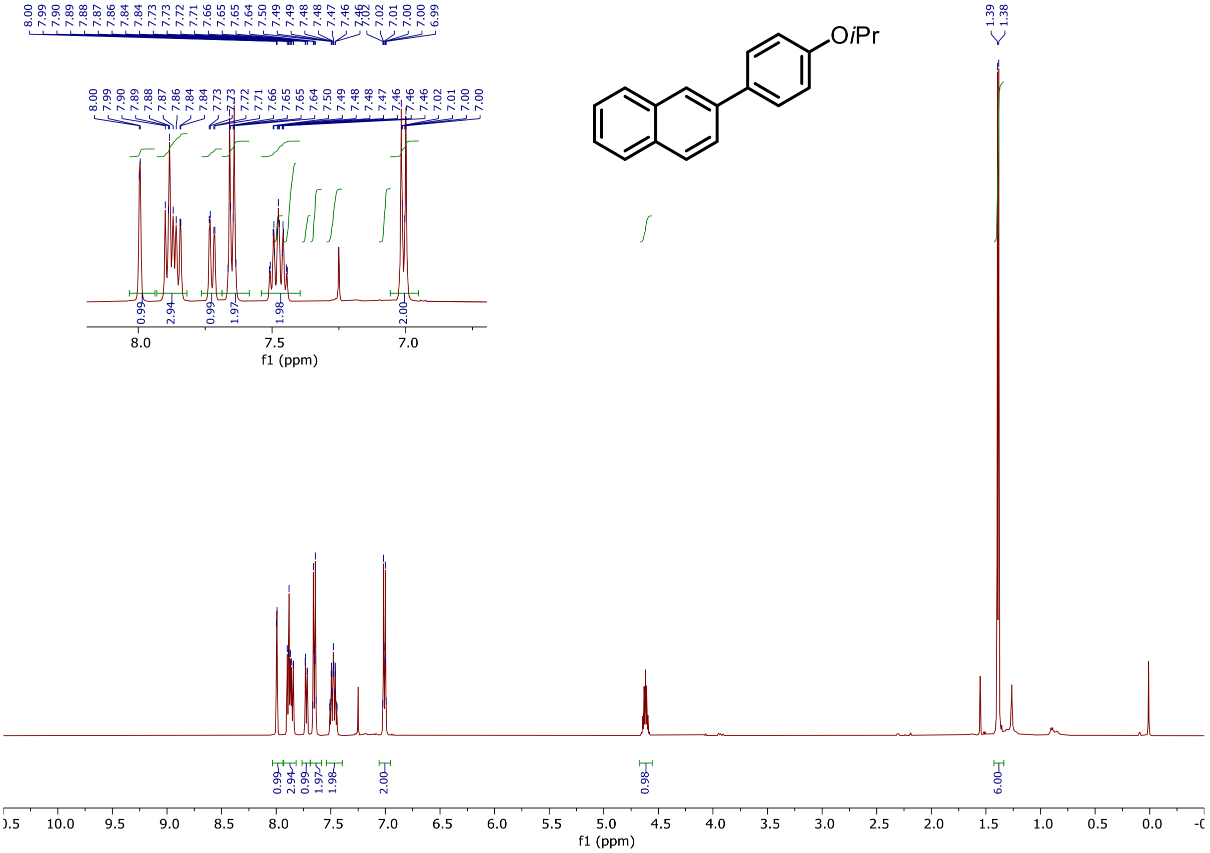

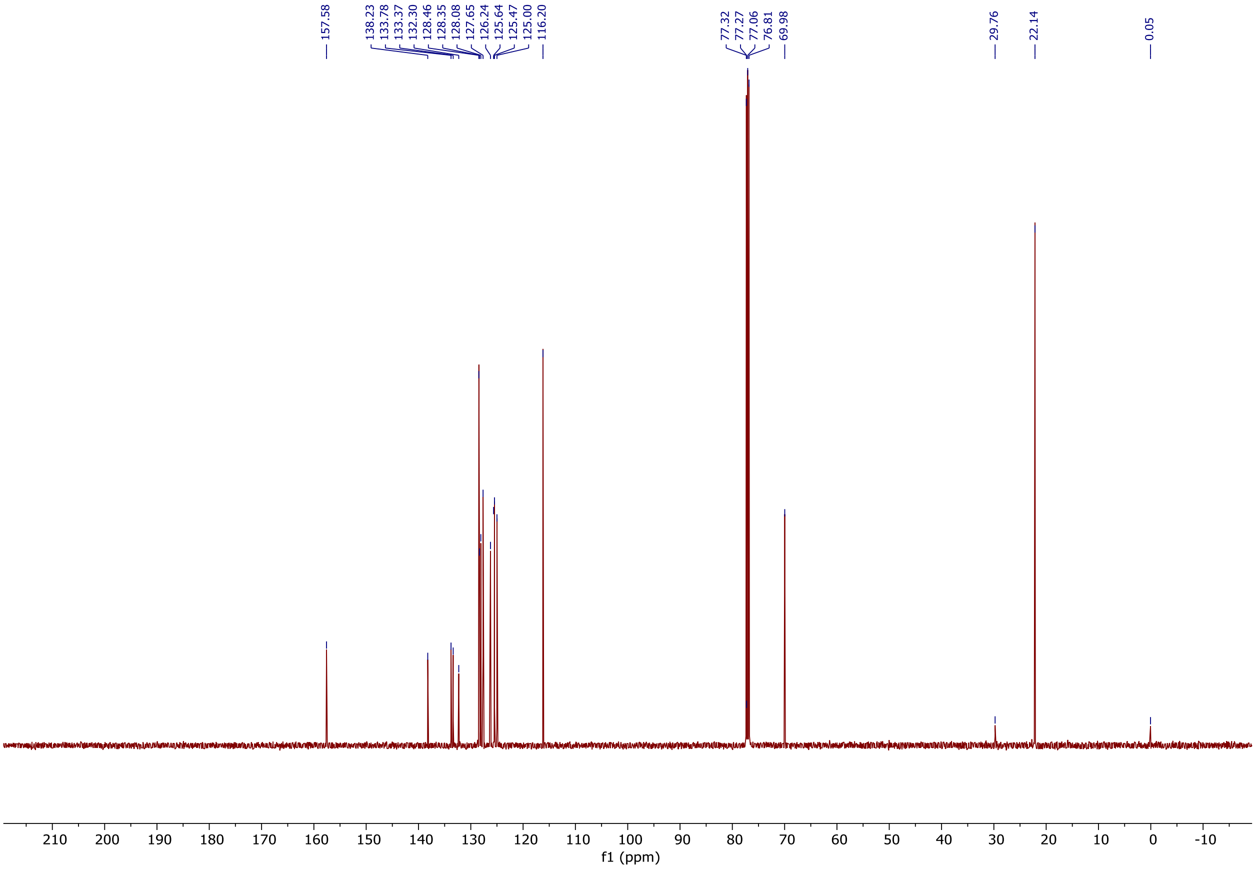

## 2-(4-Trifluoromethylphenyl) naphthalene (3g)

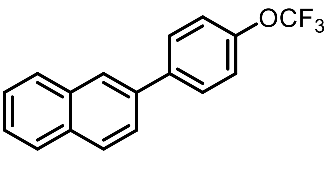

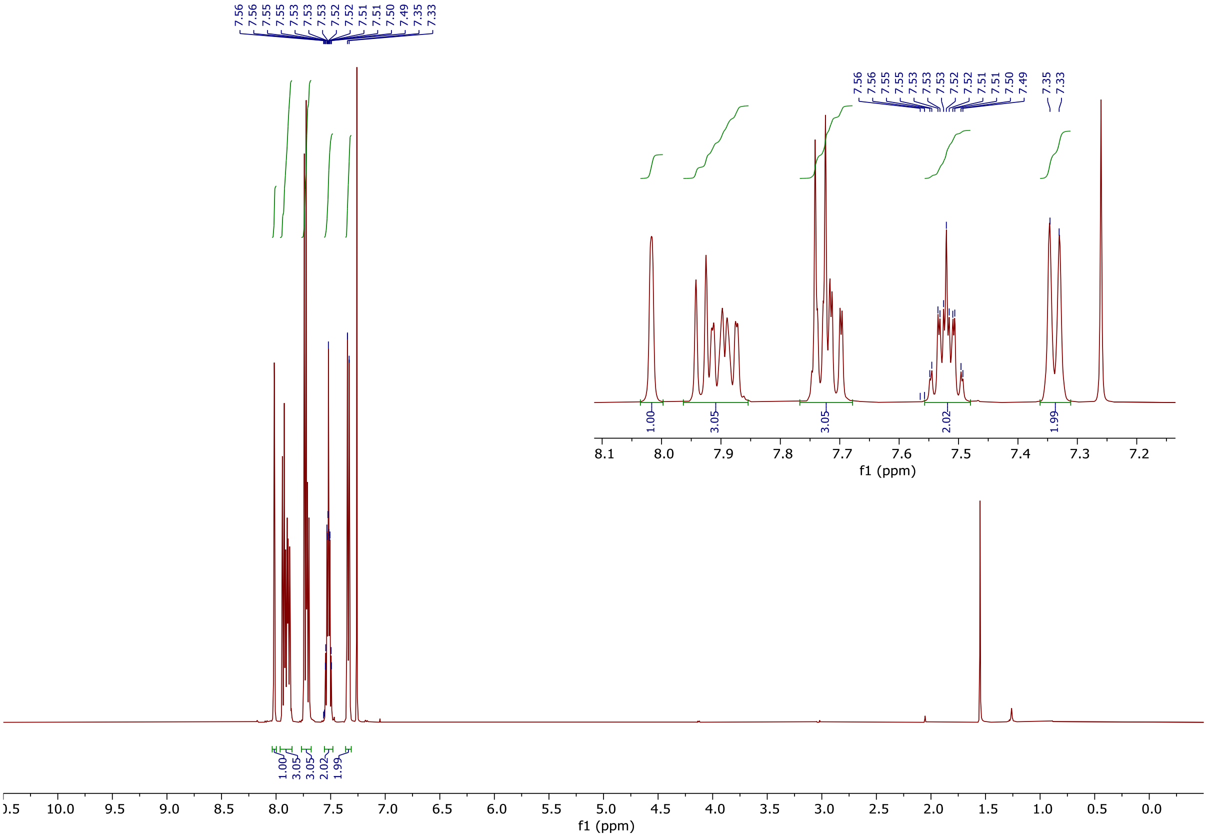
7.56 7.56 7.55 7.55 7.53 7.53 7.53 7.52 7.52 7.51 7.51 7.50 7.49 7.35 7.33
1.00 3.05 3.05 2.02 1.99
f1 (ppm)

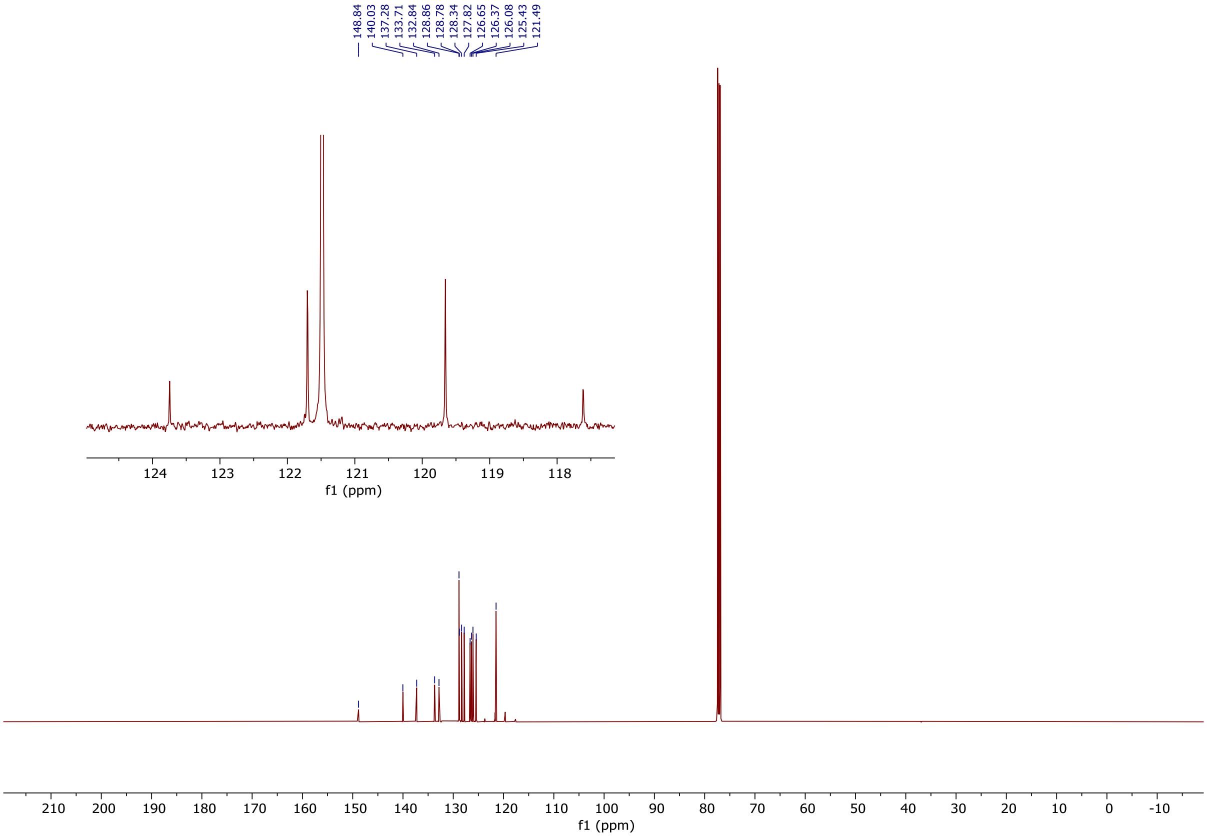
148.84 140.03 137.28 133.71 132.84 128.86 128.78 128.34 127.82 126.65 126.37 126.08 125.43 121.49
f1 (ppm)

$^{19}$F NMR (659 MHz, $CDCl_3$) δ -57.78.

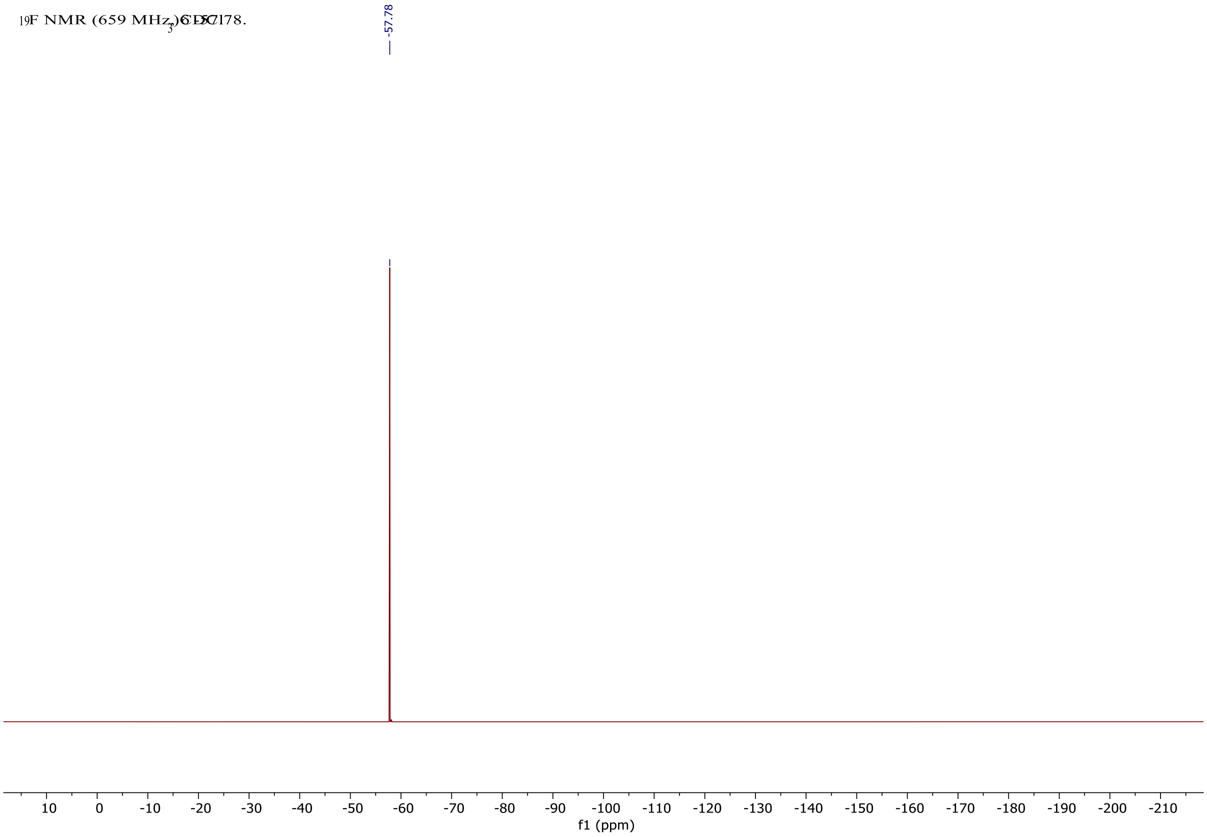

## 2-(4-methylphenyl) naphthalene (3h)

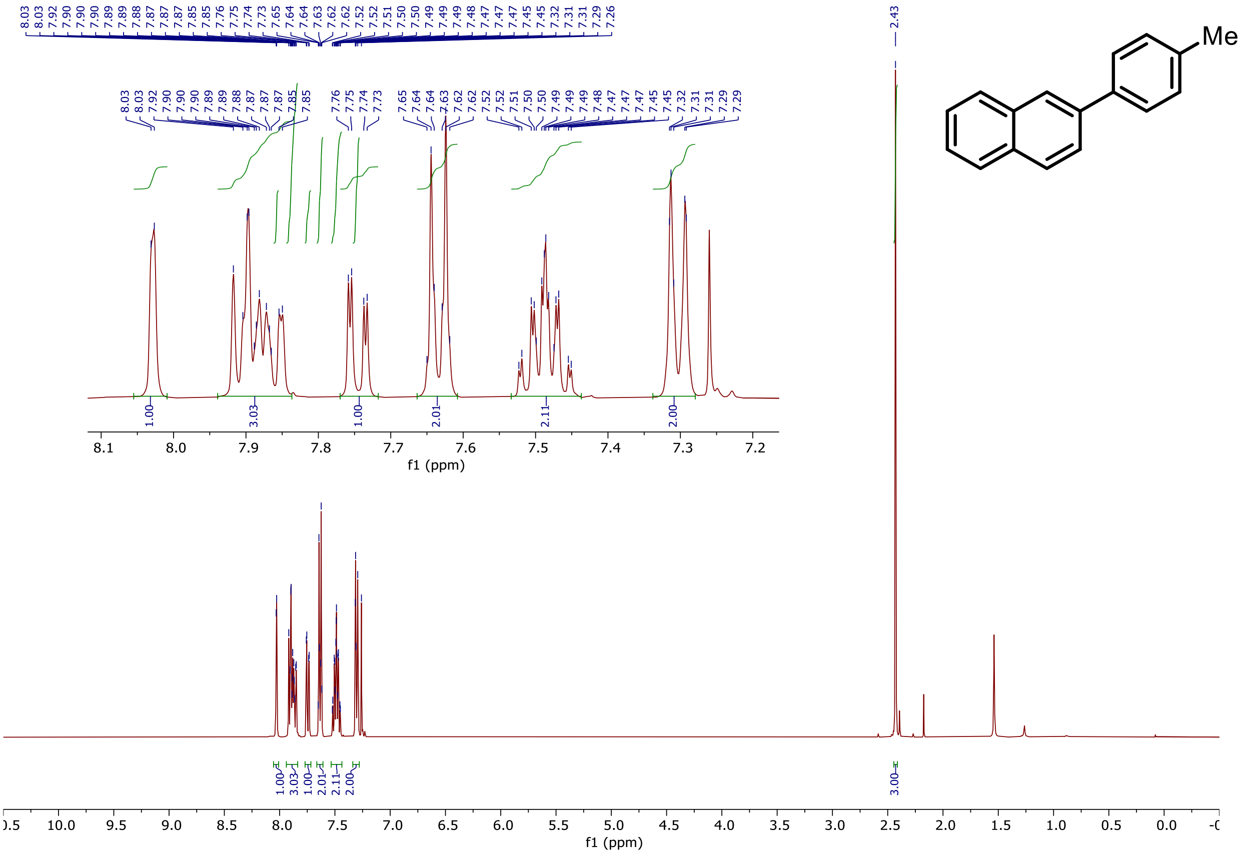


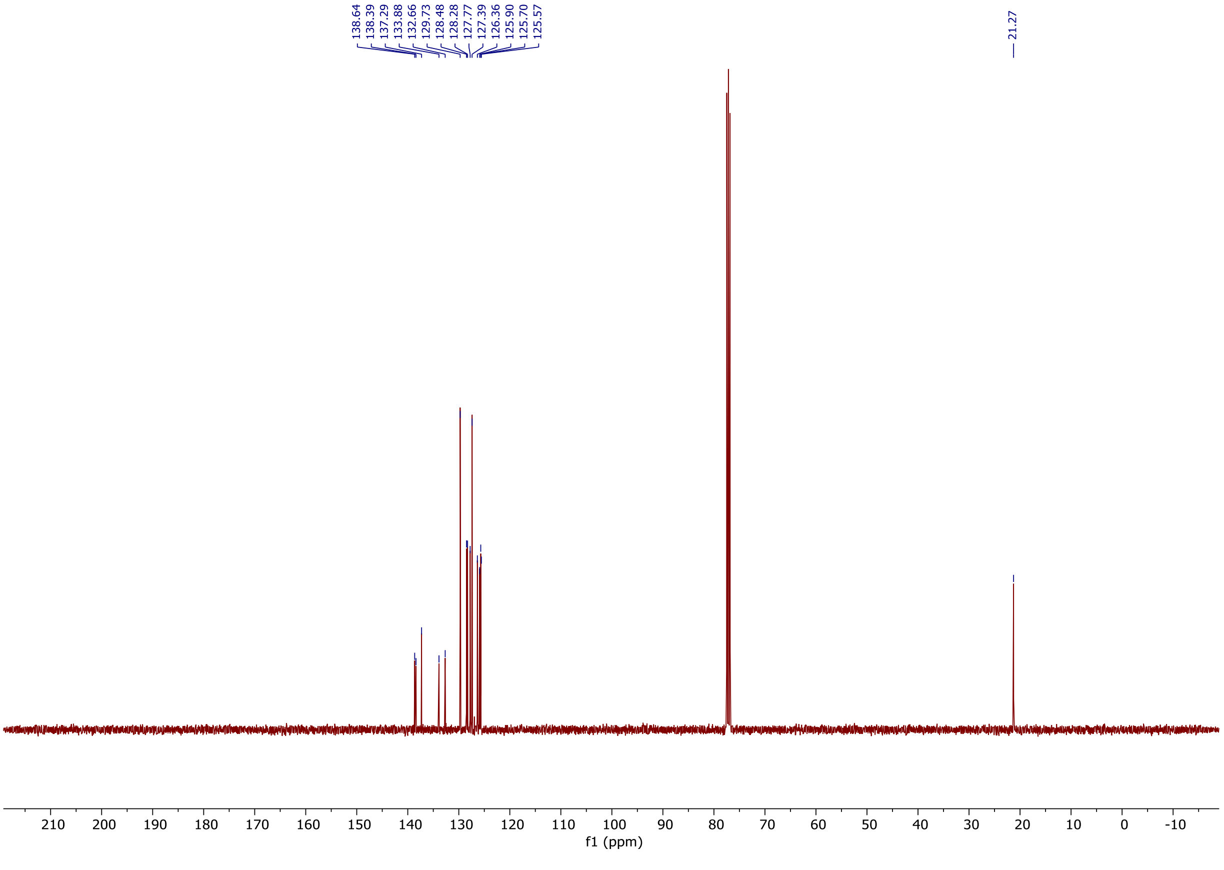

**2-(4-n-Butyl phenyl) naphthalene (3i)**

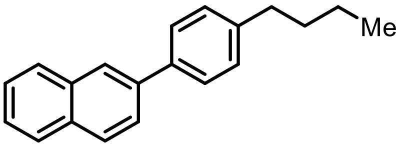


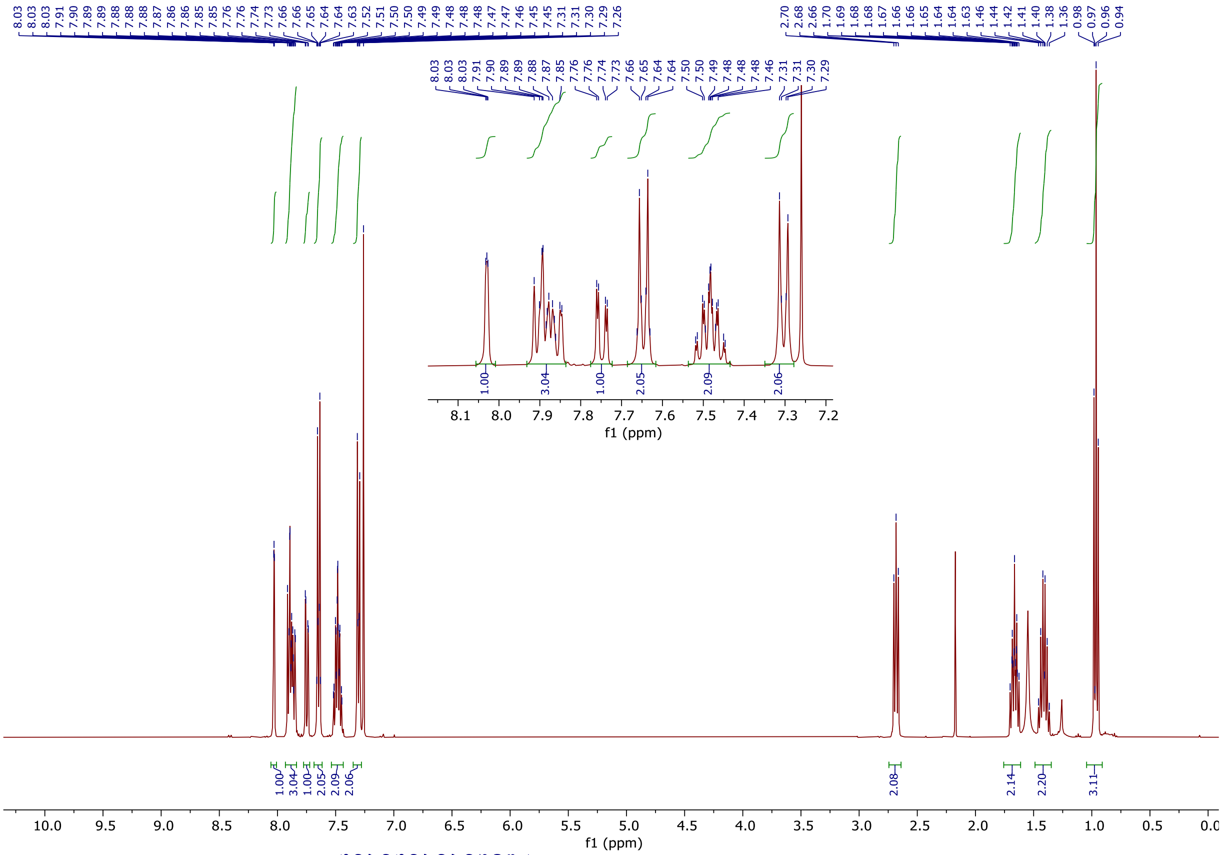

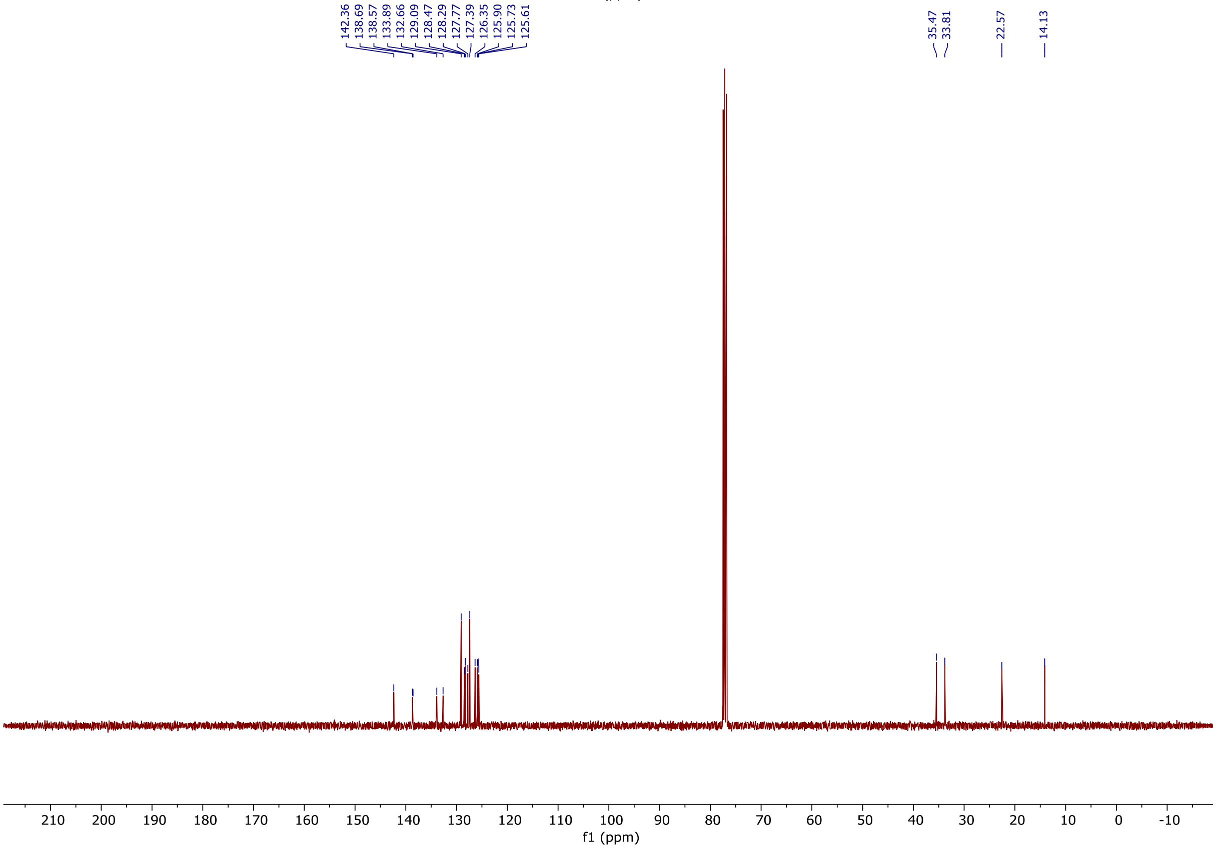

## 2-(4-tert-butylphenyl) naphthalene (3j)

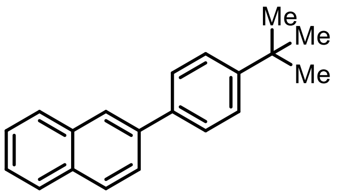

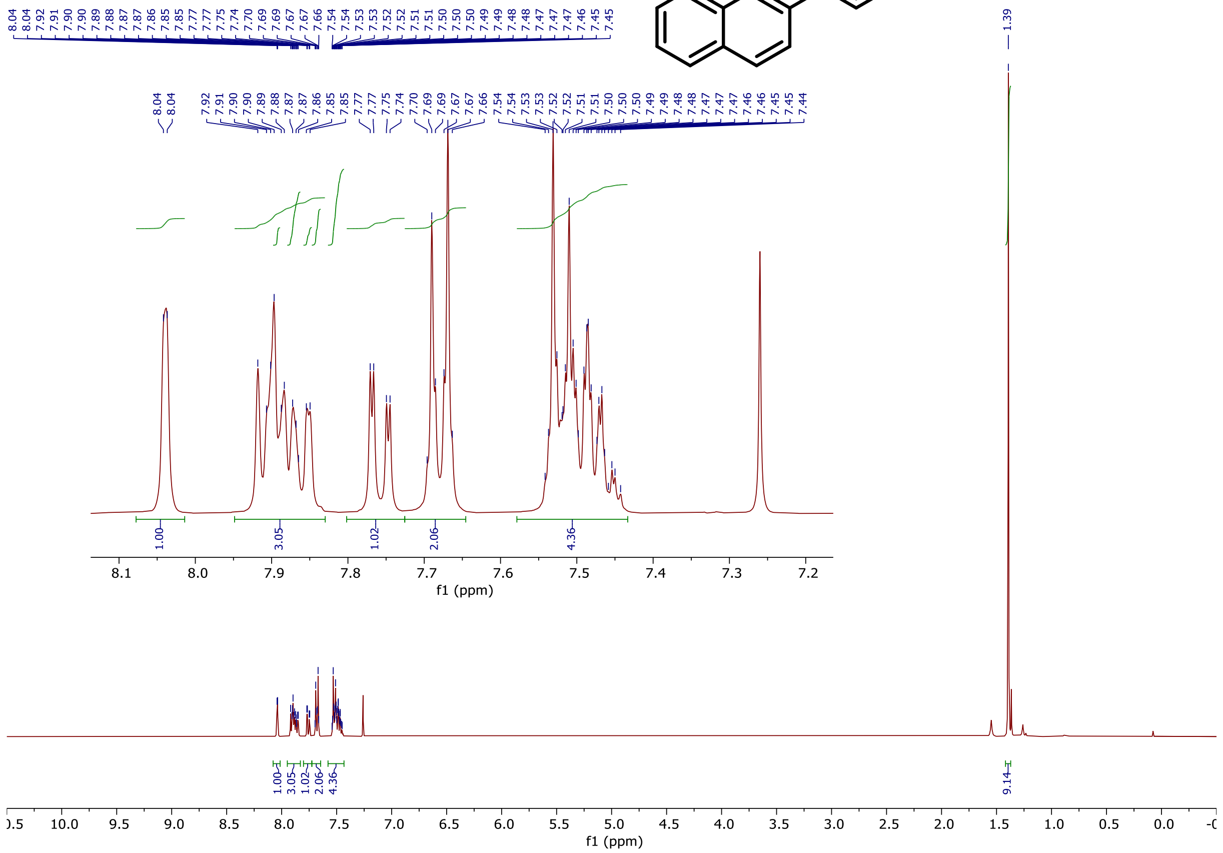

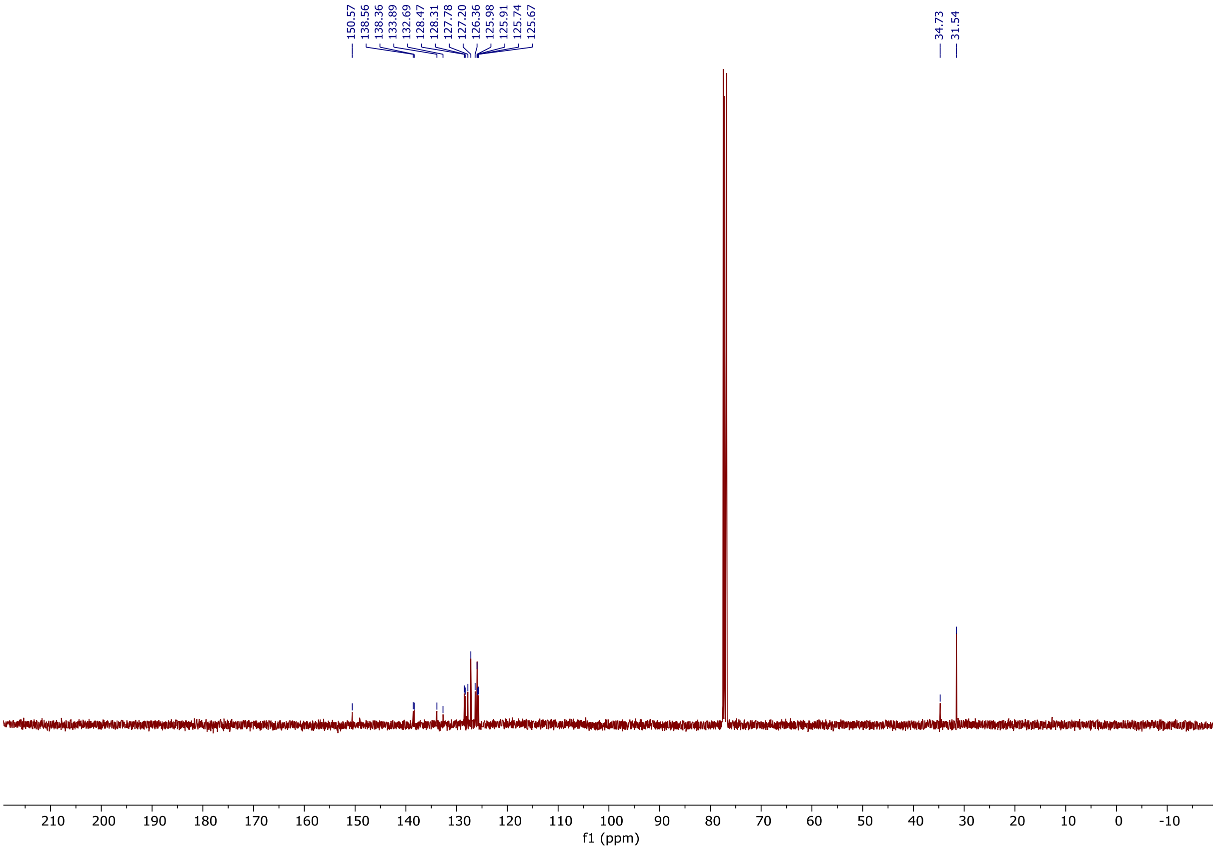


## 2-(4-Acetylphenyl) naphthalene (3k)

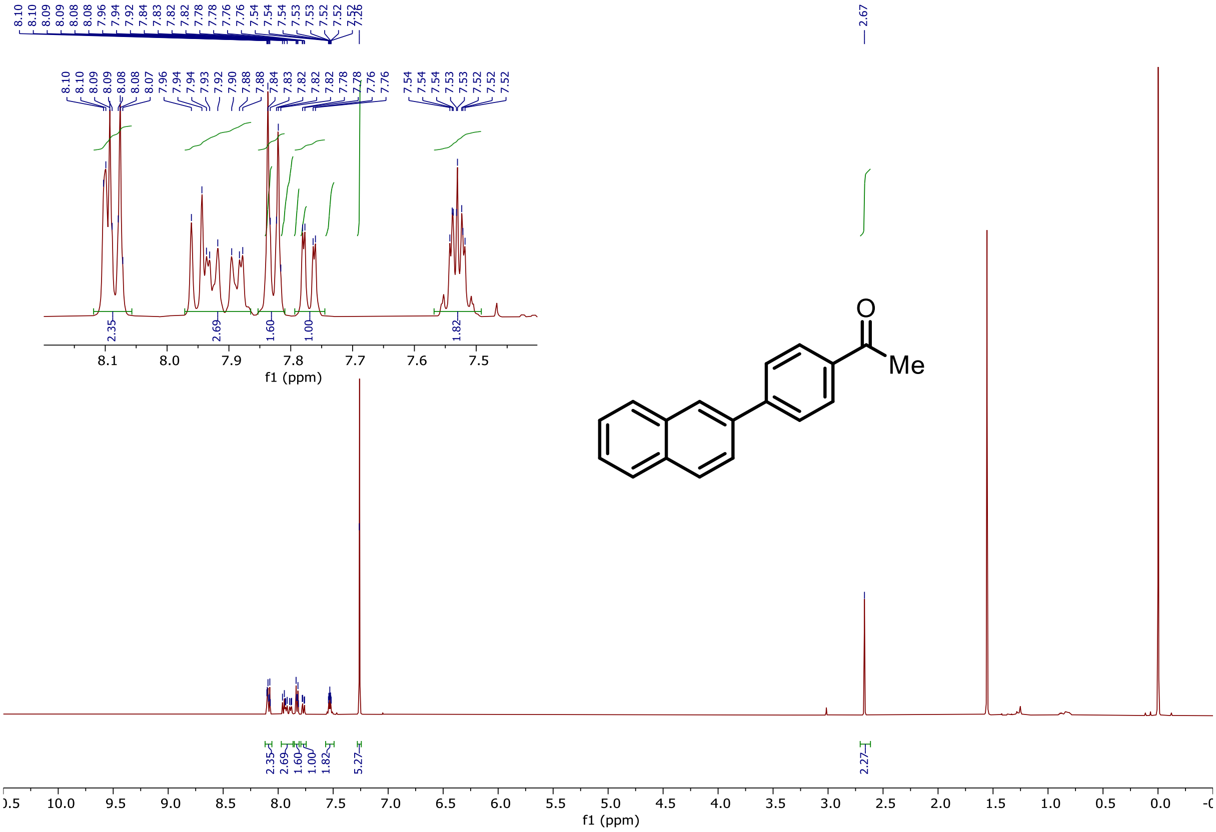

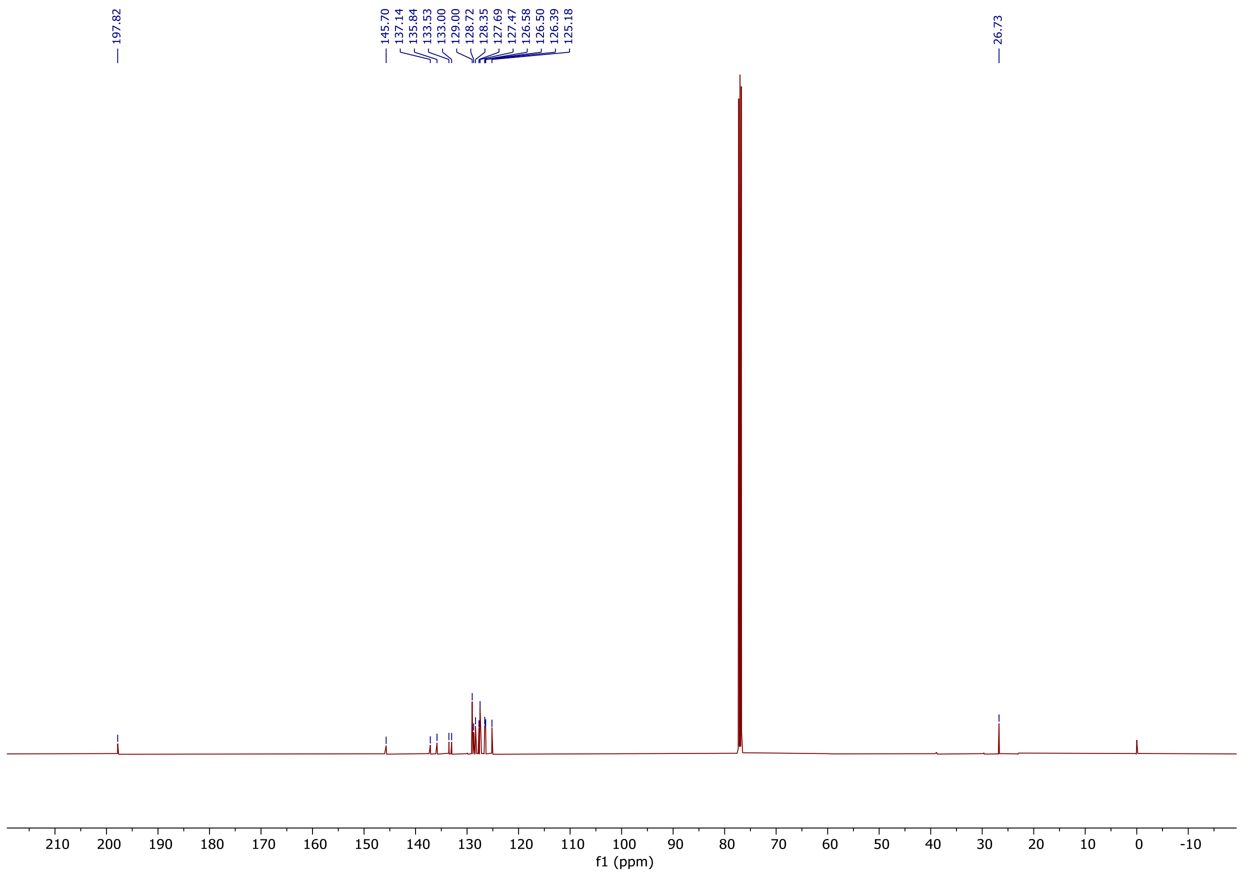


**2-(4-ethoxycarbonylphenyl) naphthalene (3l)**

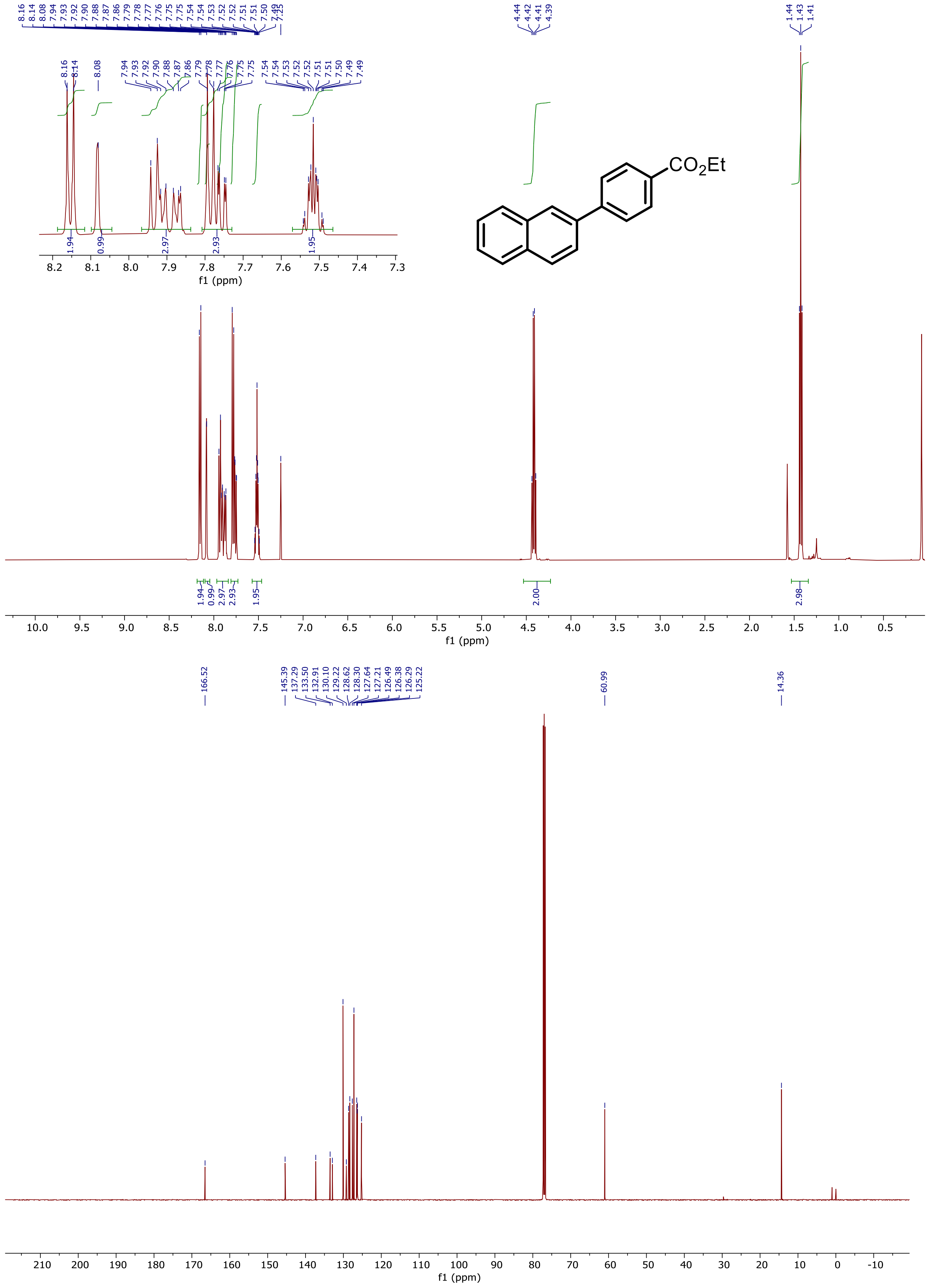

## 2-(4-trifluorophenyl) naphthalene (3m)

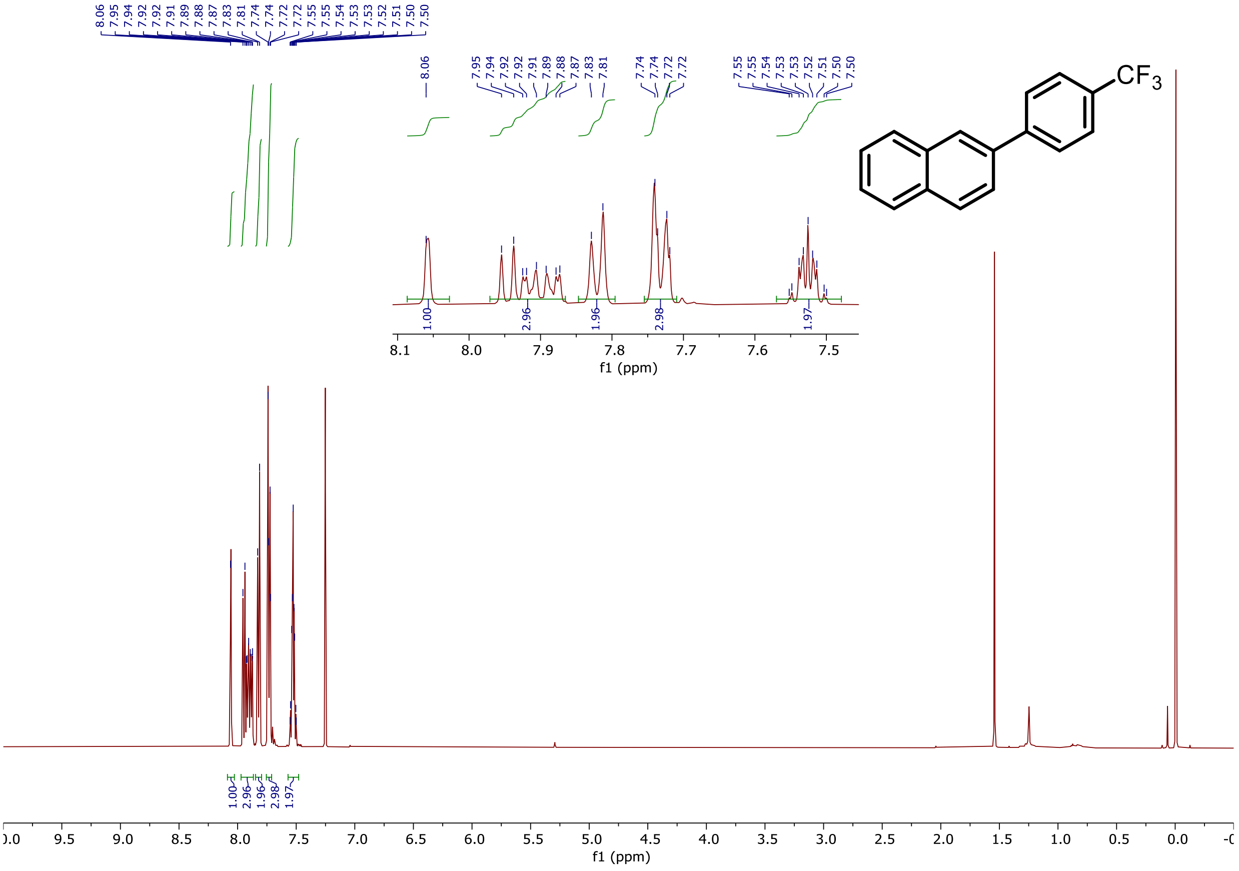


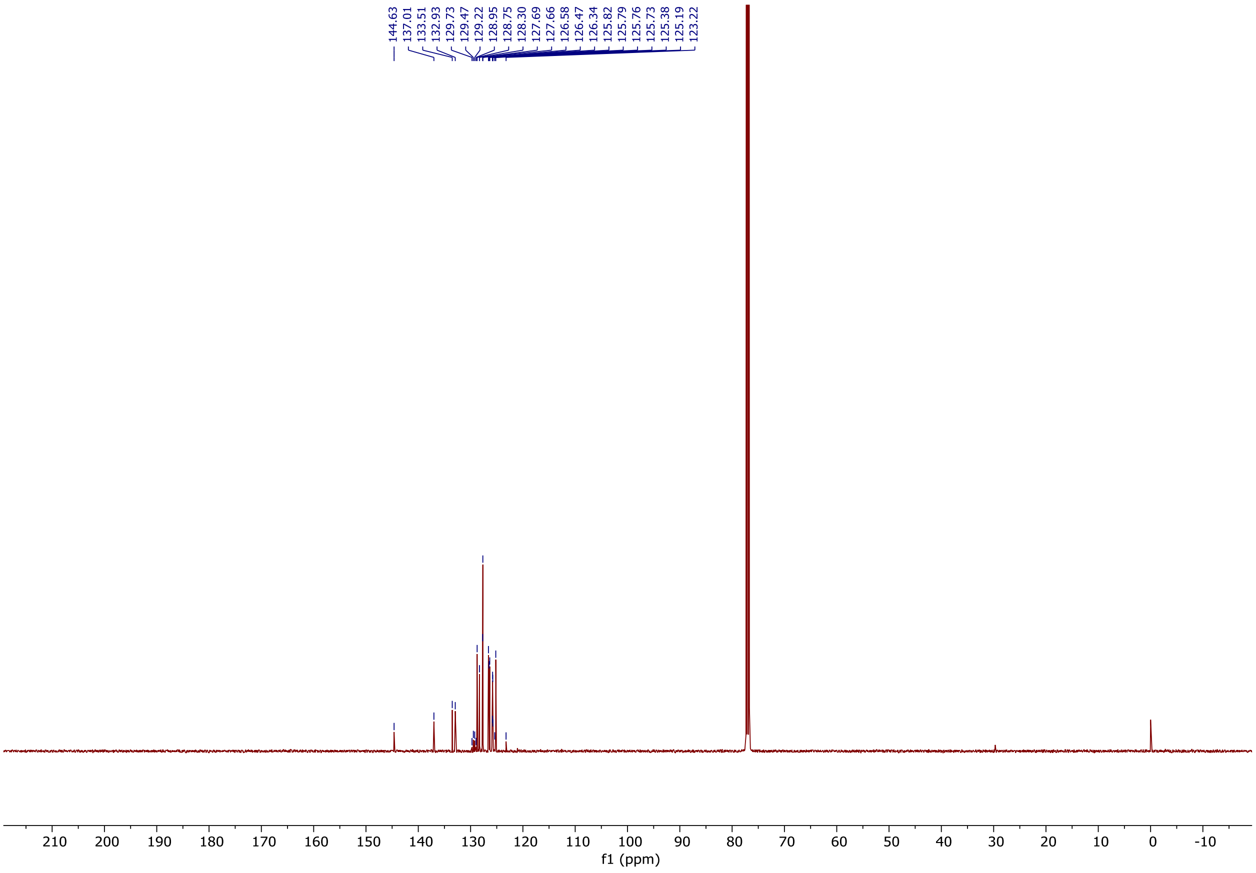


## 2-(4-cyanophenyl) naphthalene (3n)

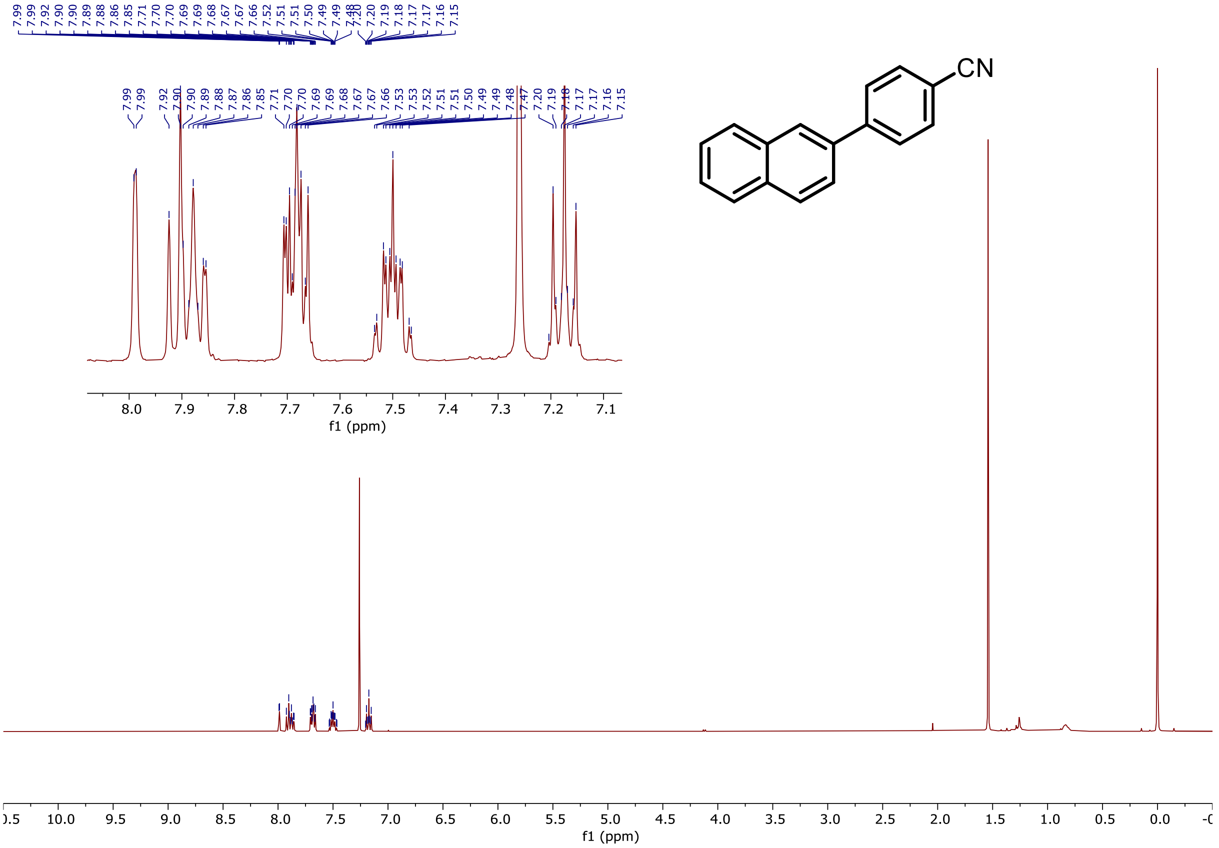


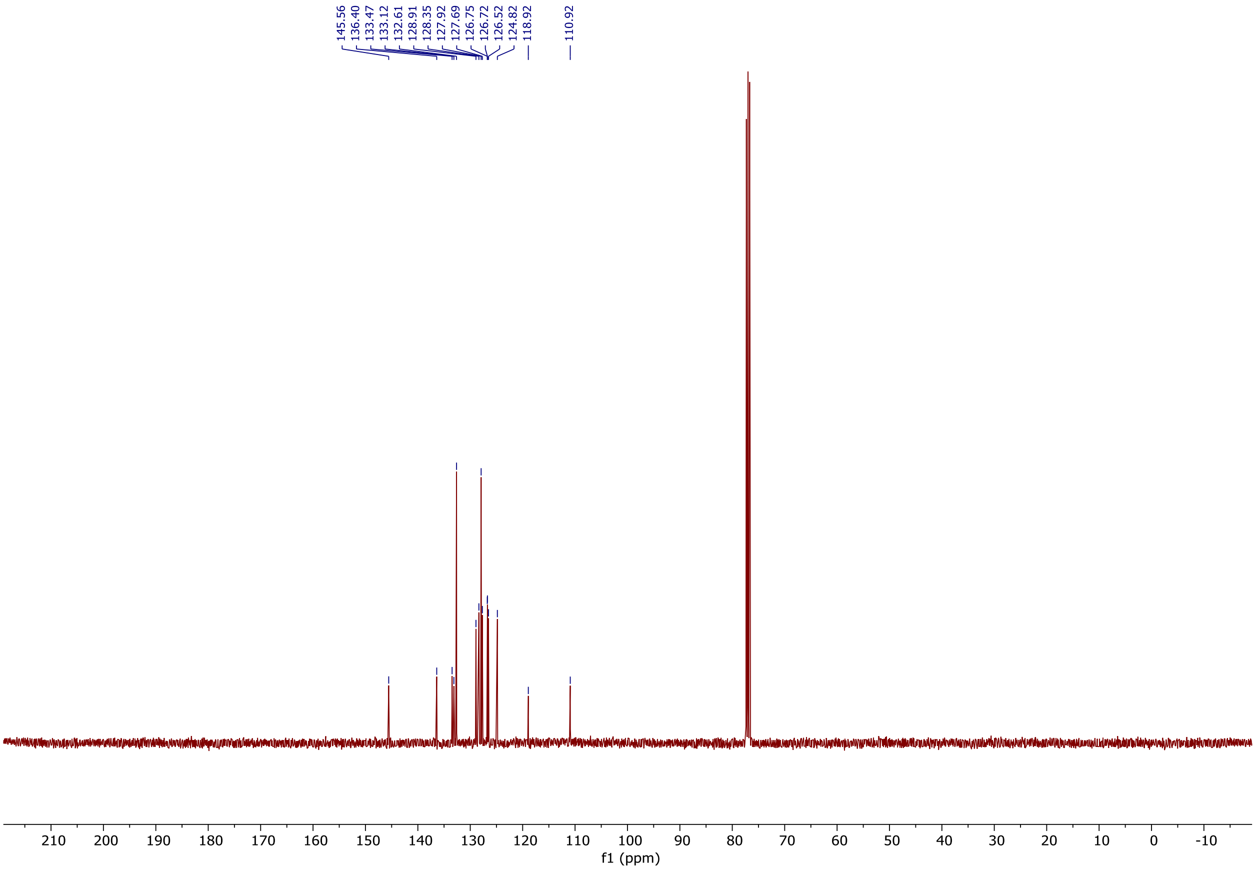


**3-(dibenzo[b,d]thiophen-2-yl)pyridine (3q)**

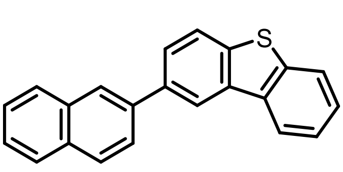

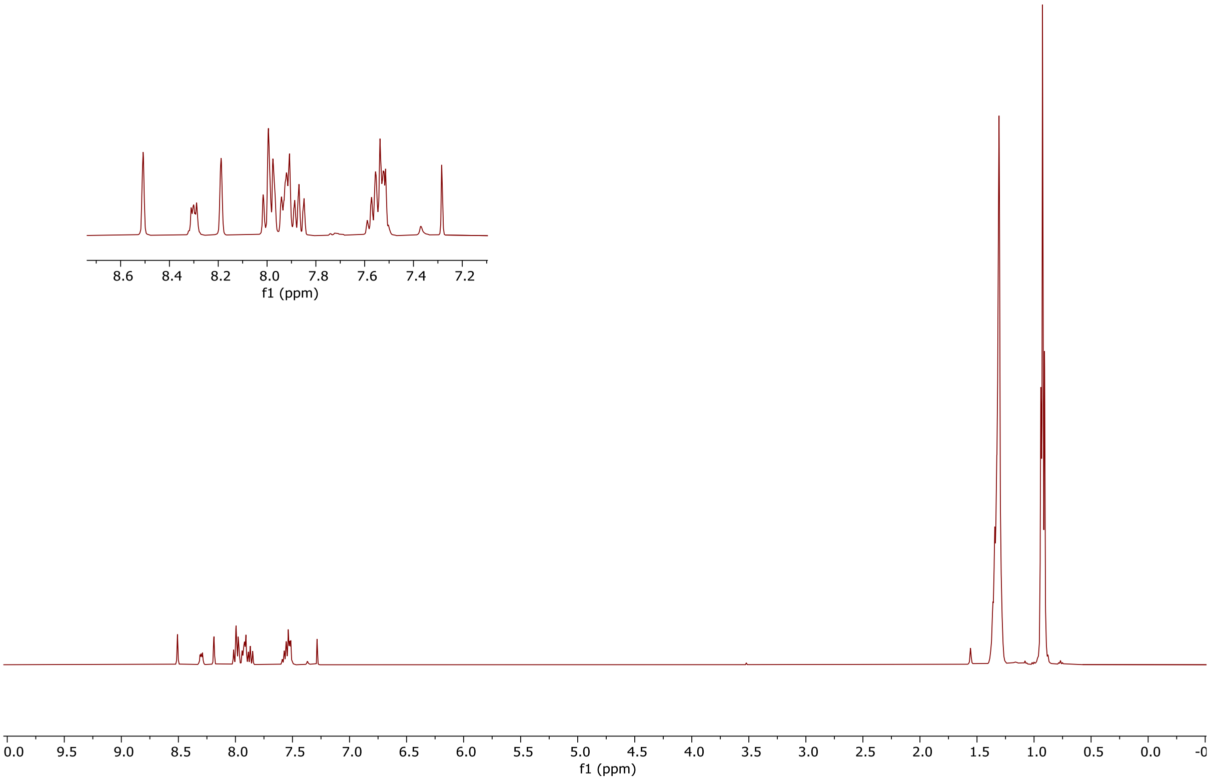


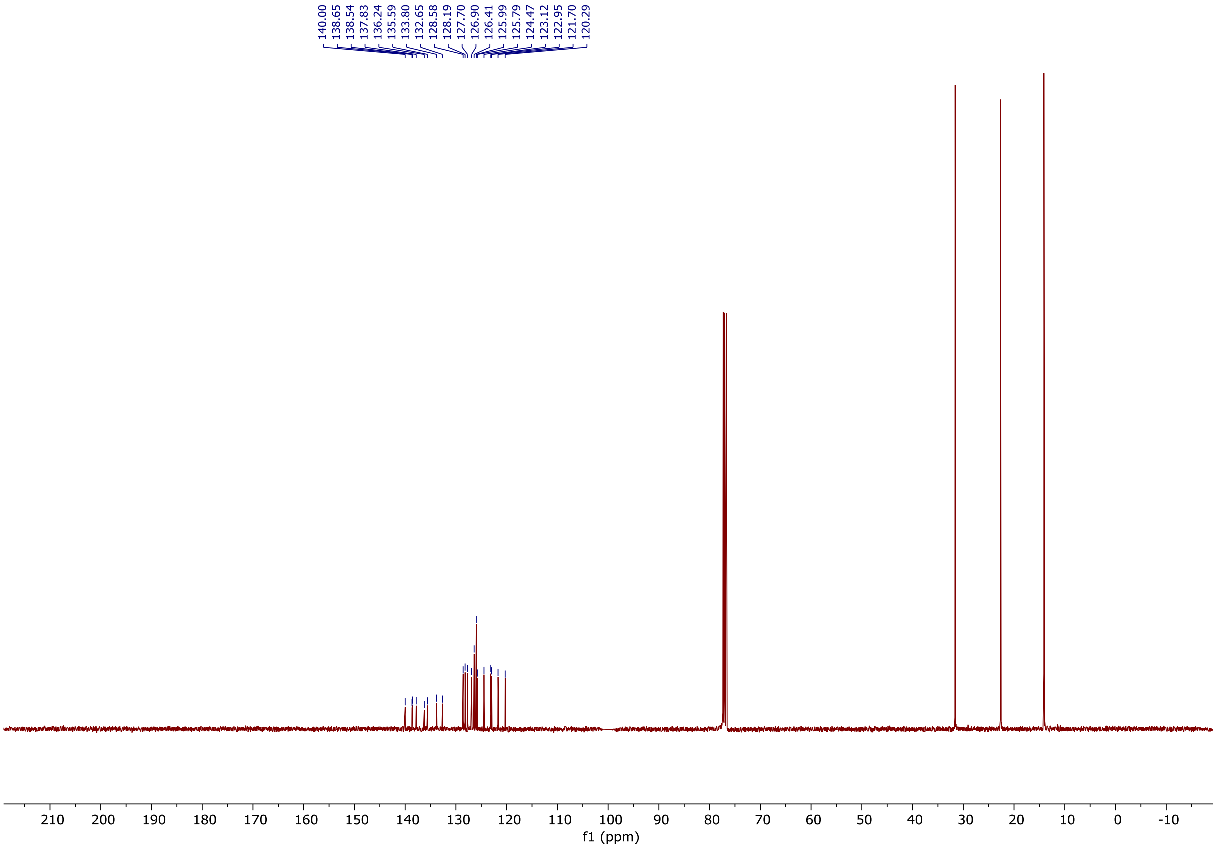


**residual hexane in NMR. Protodeboronated dibenzothiophene co-elutes with C-C coupled product. Cleanest fractions shown. Values given in main text are NMR yields.

# 1-(4-Fluorophenyl)naphthalene (3d)

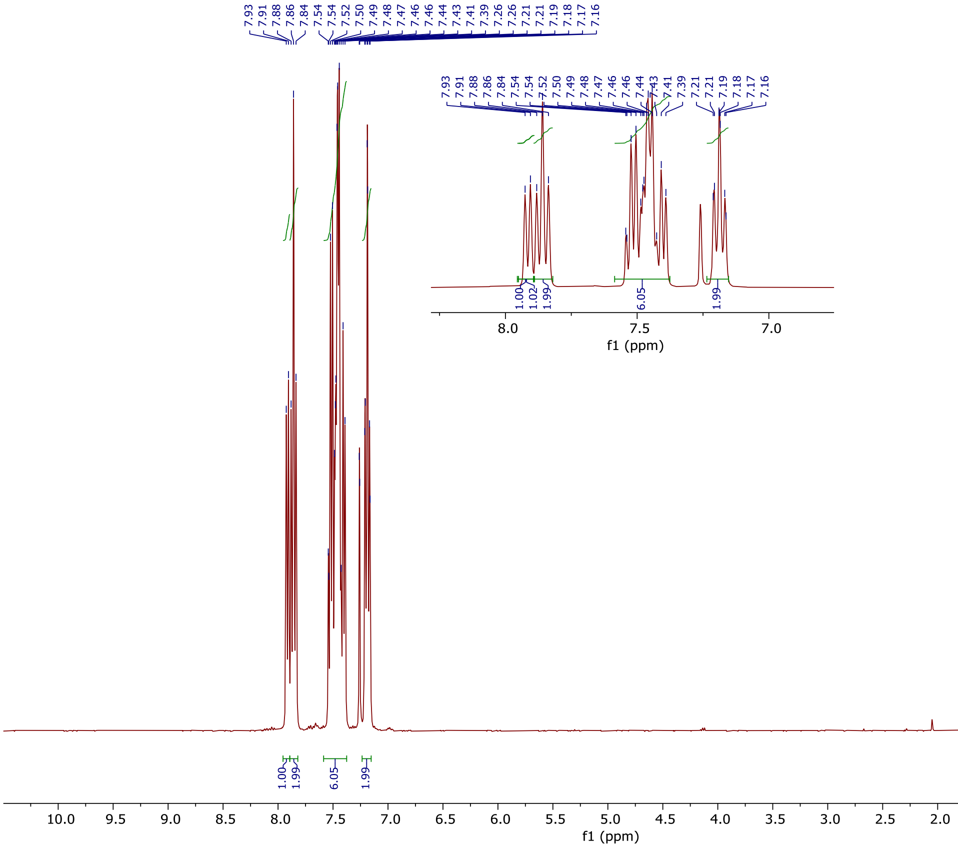


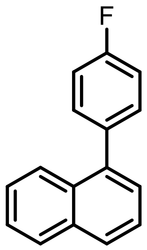


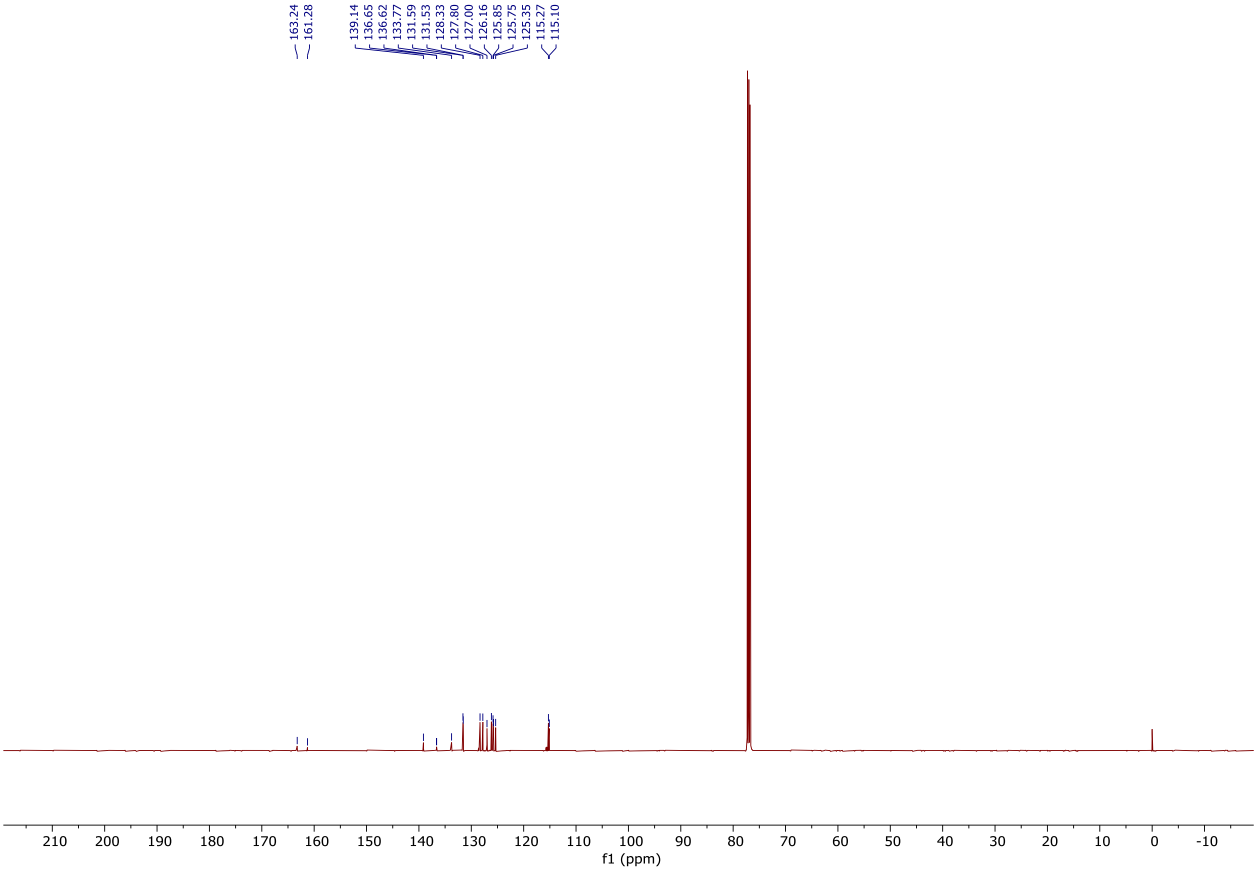

**4-Fluoro-1,1':4',1''-terphenyl (3e)**

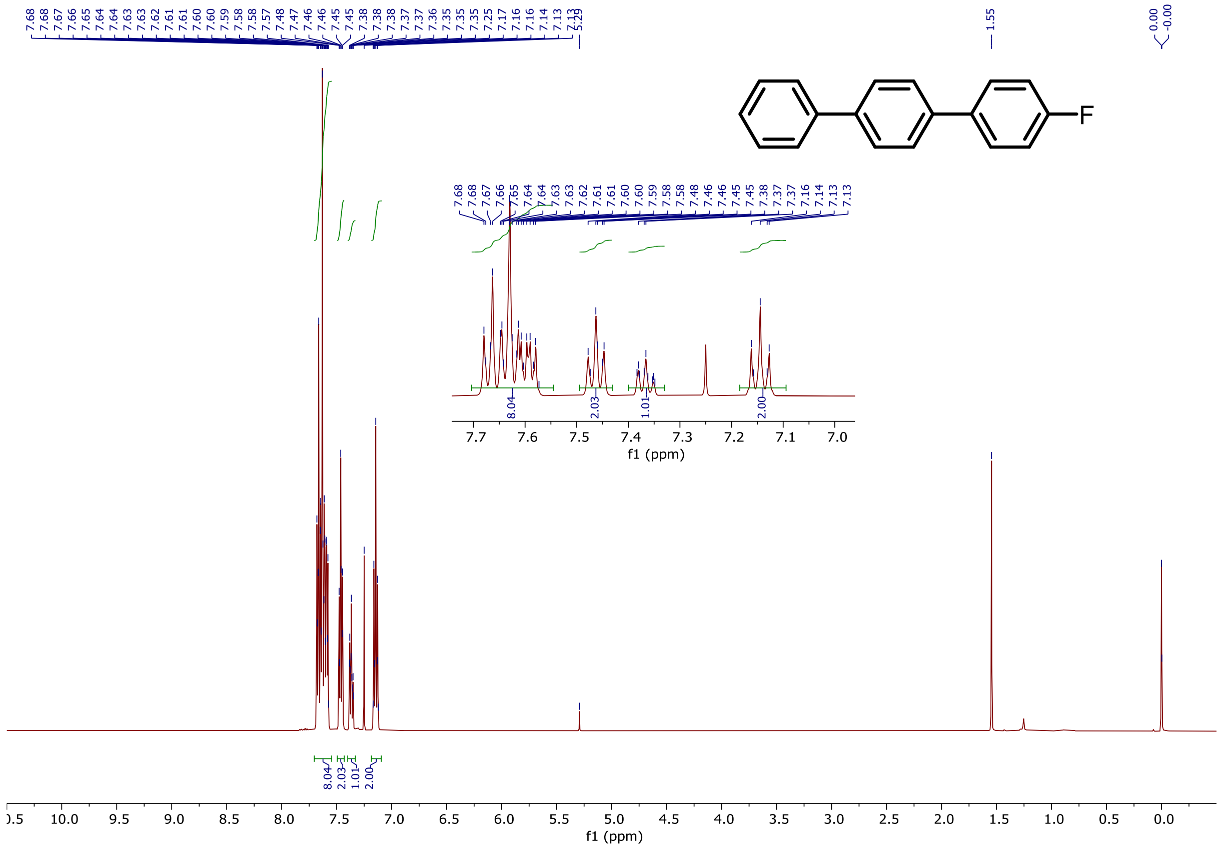

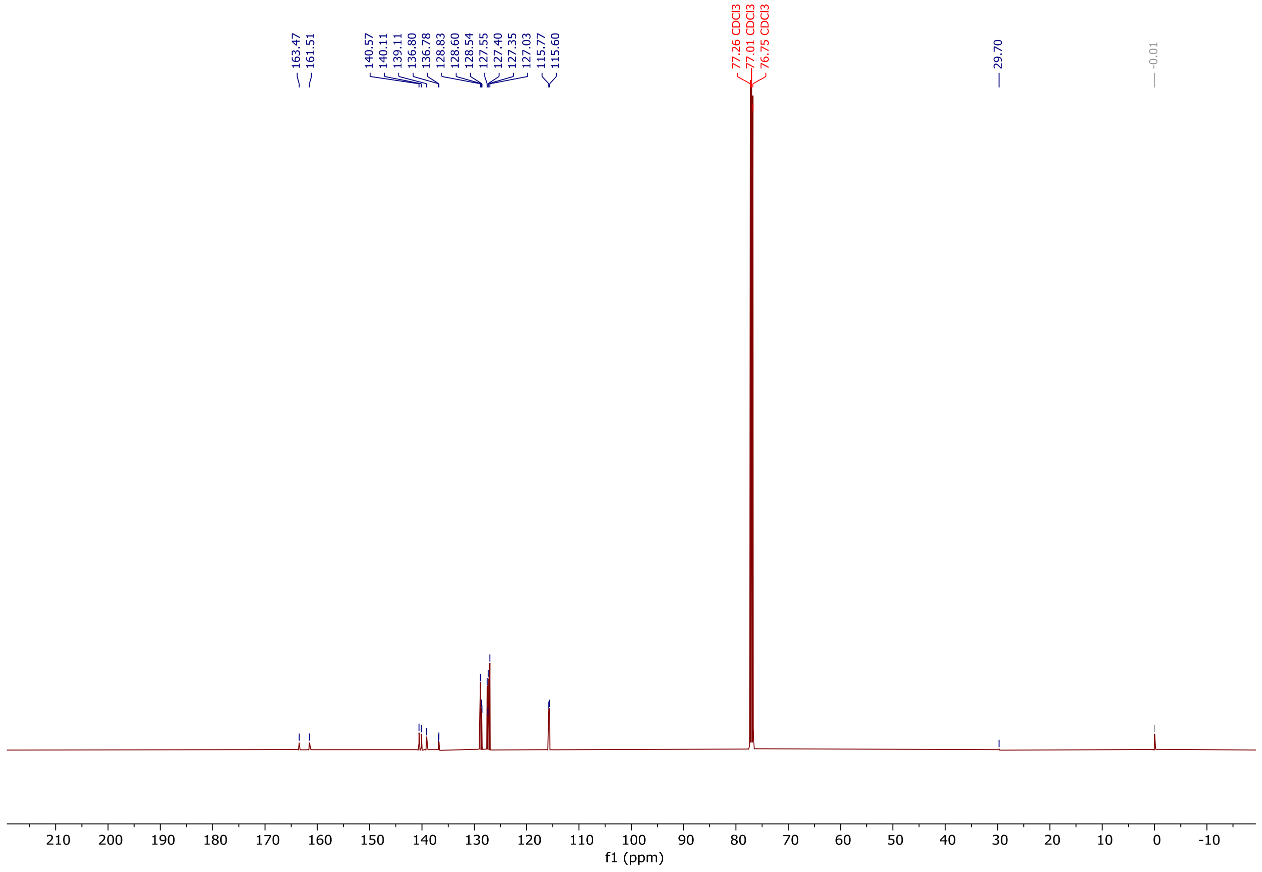

## 1-(4-fluorophenyl)-4-methoxynaphthalene (3r)

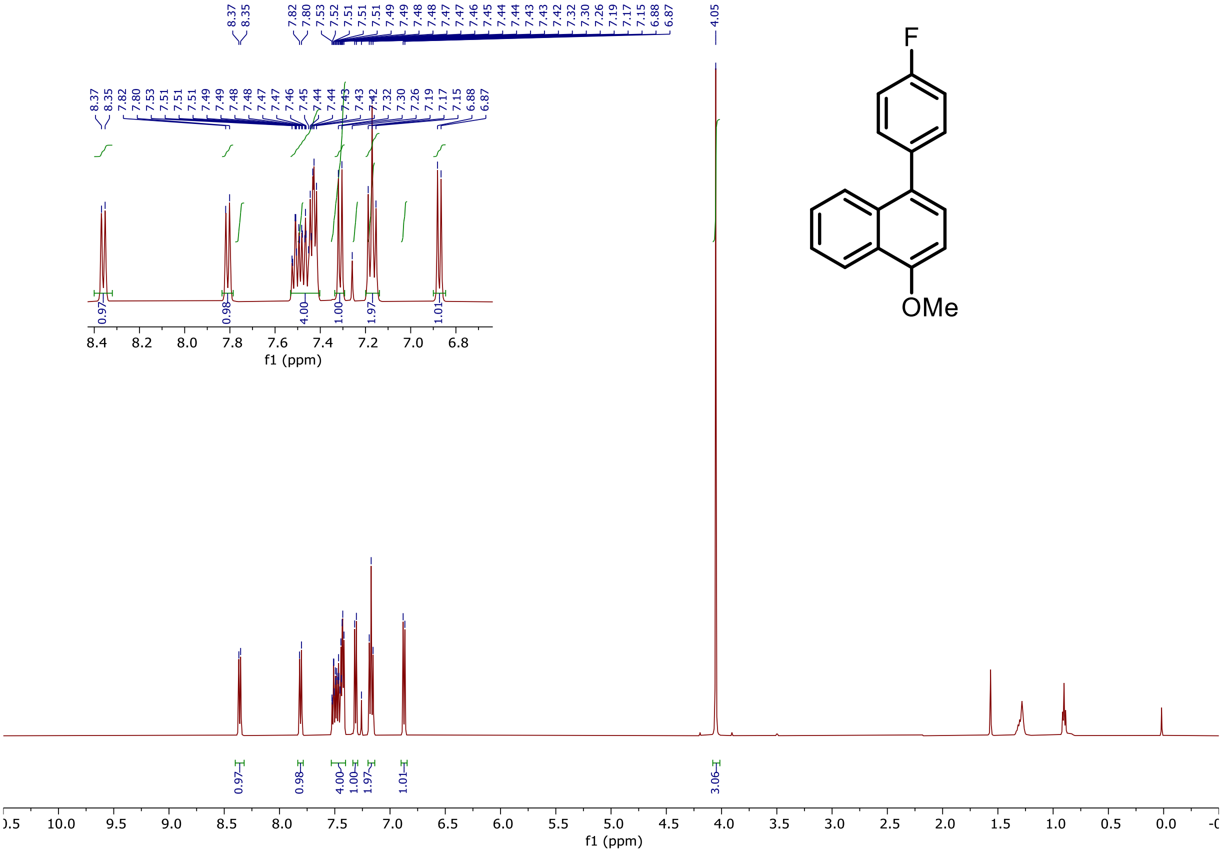


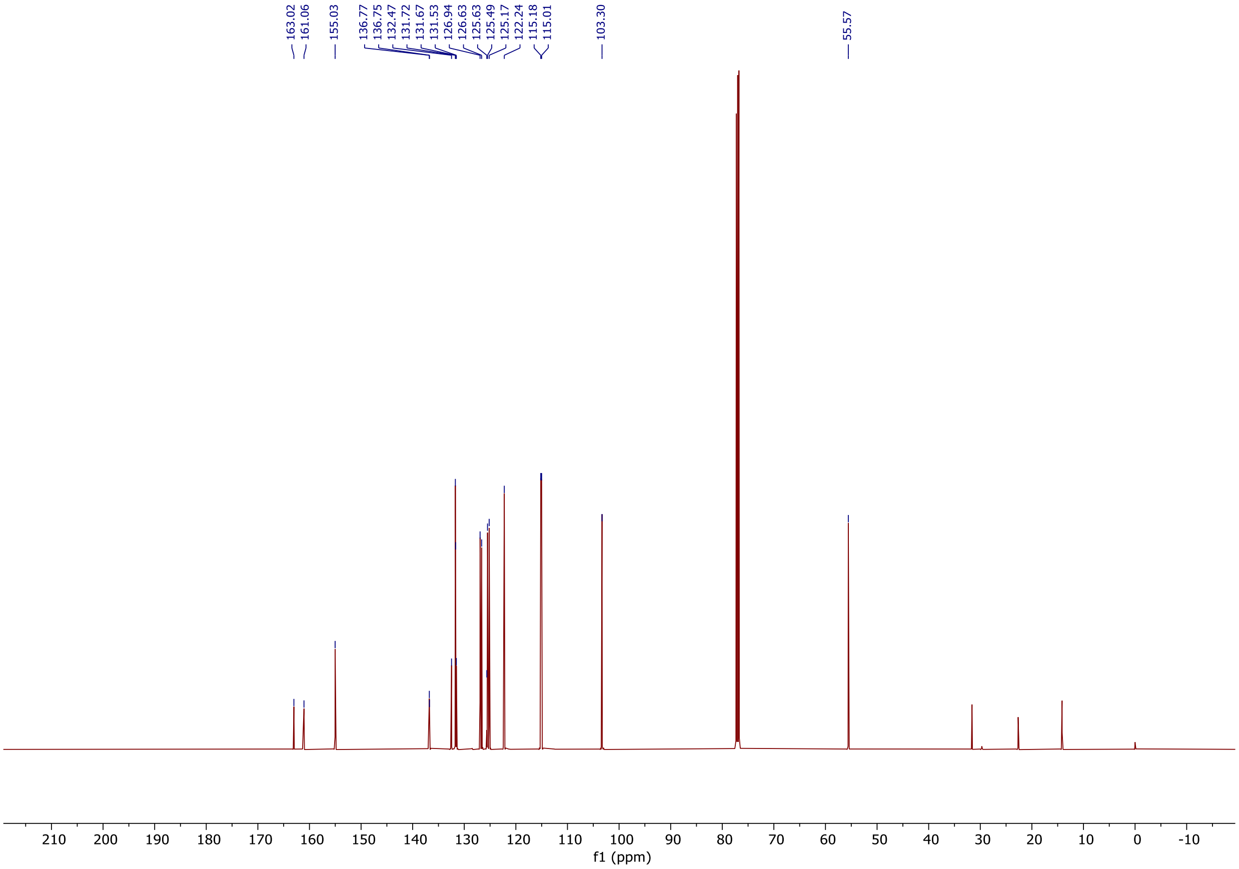

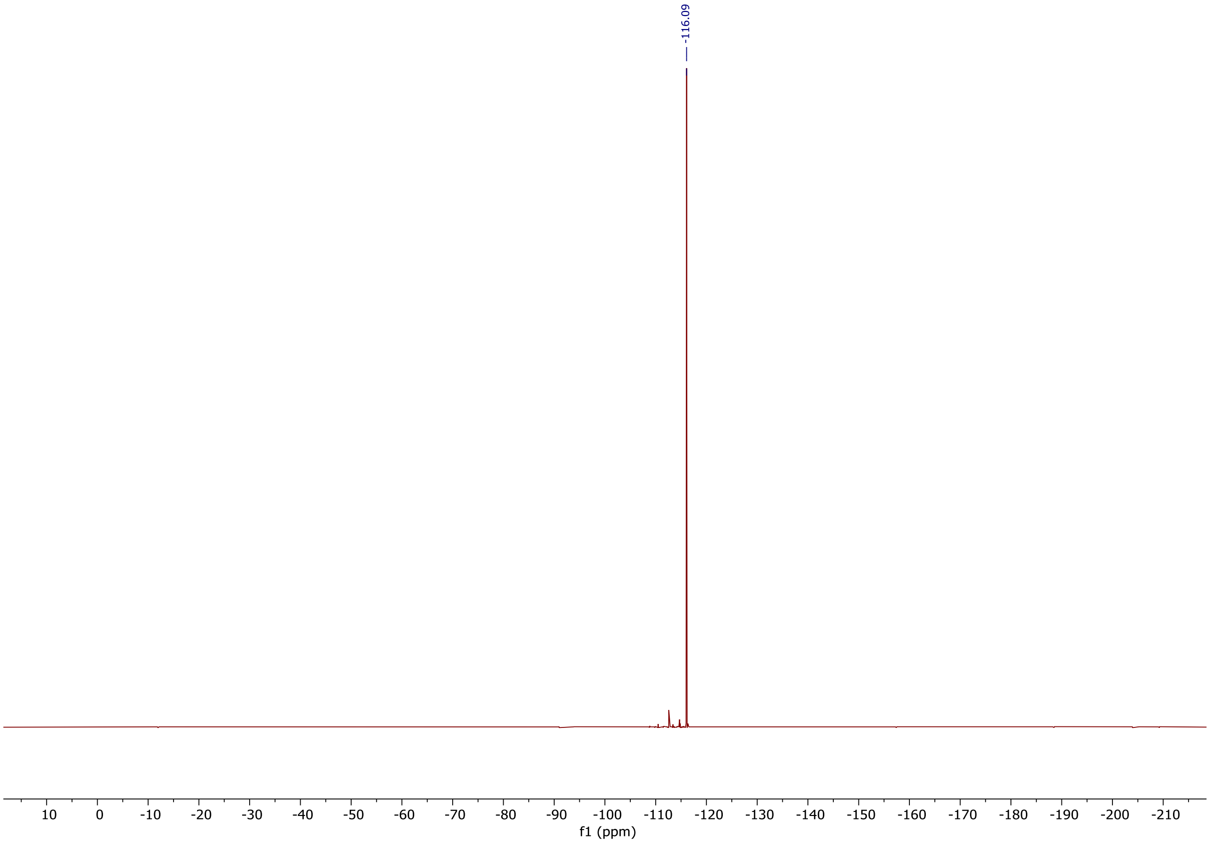
-116.09
10
0
-10
-20
-30
-40
-50
-60
-70
-80
-90
-100
-110
-120
-130
-140
-150
-160
-170
-180
-190
-200
-210
f1 (ppm)

## Preparation of 4-fluoro-4'-methoxy-1,1'-biphenyl (3s)

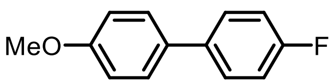

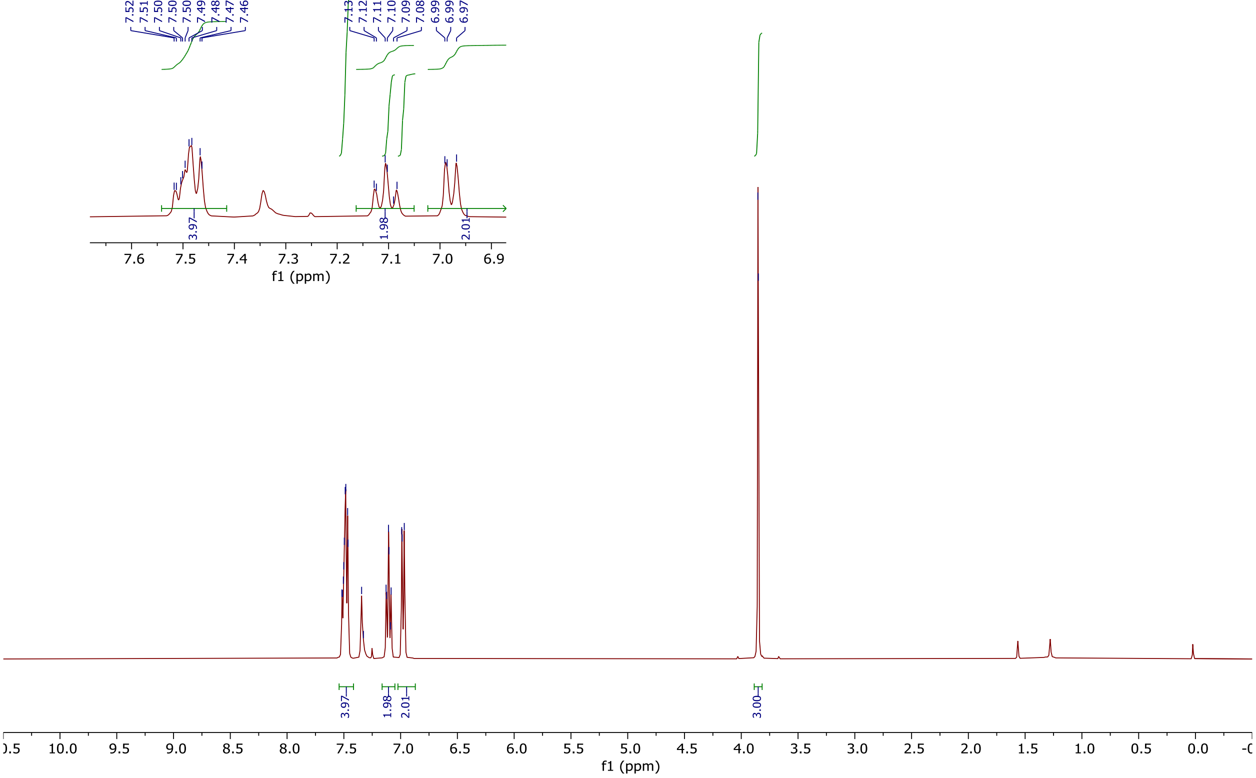

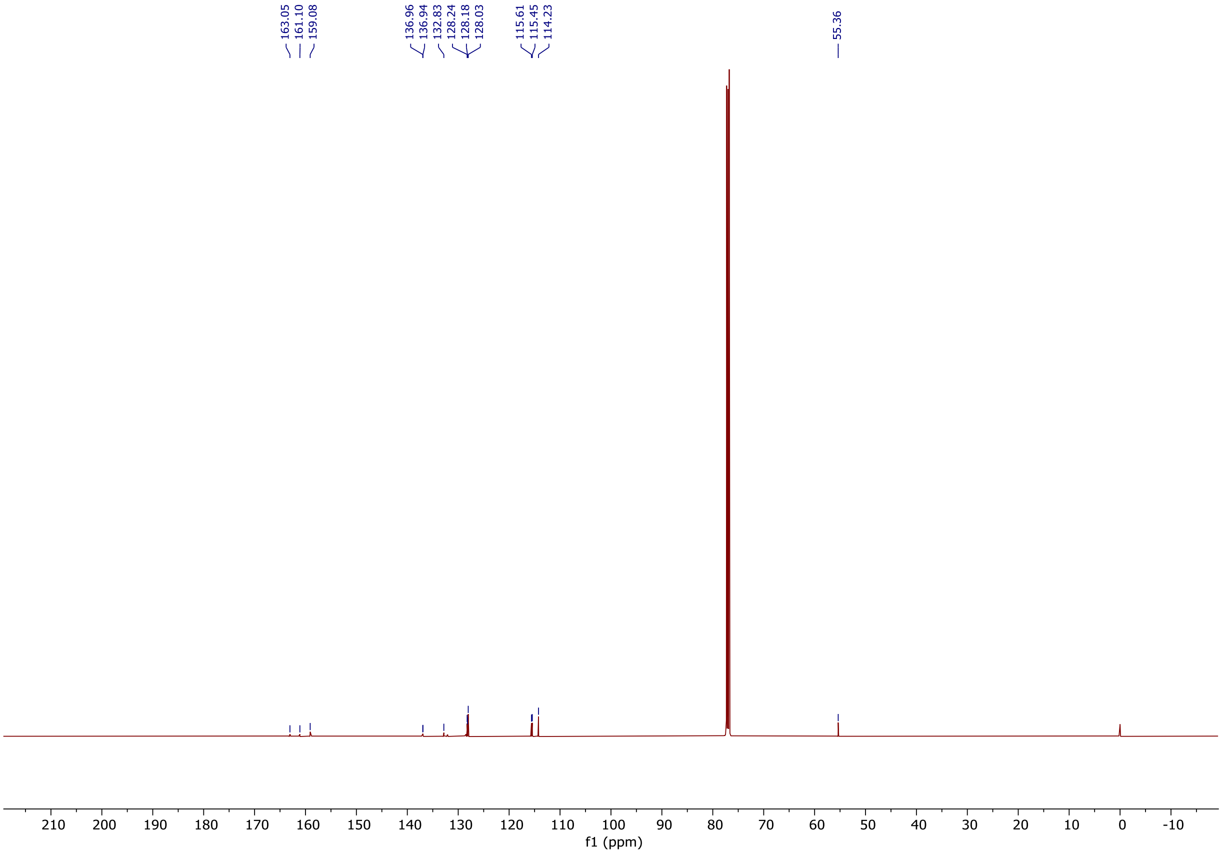

**4'-fluoro-[1,1'-biphenyl]-4-carbonitrile (3t)**

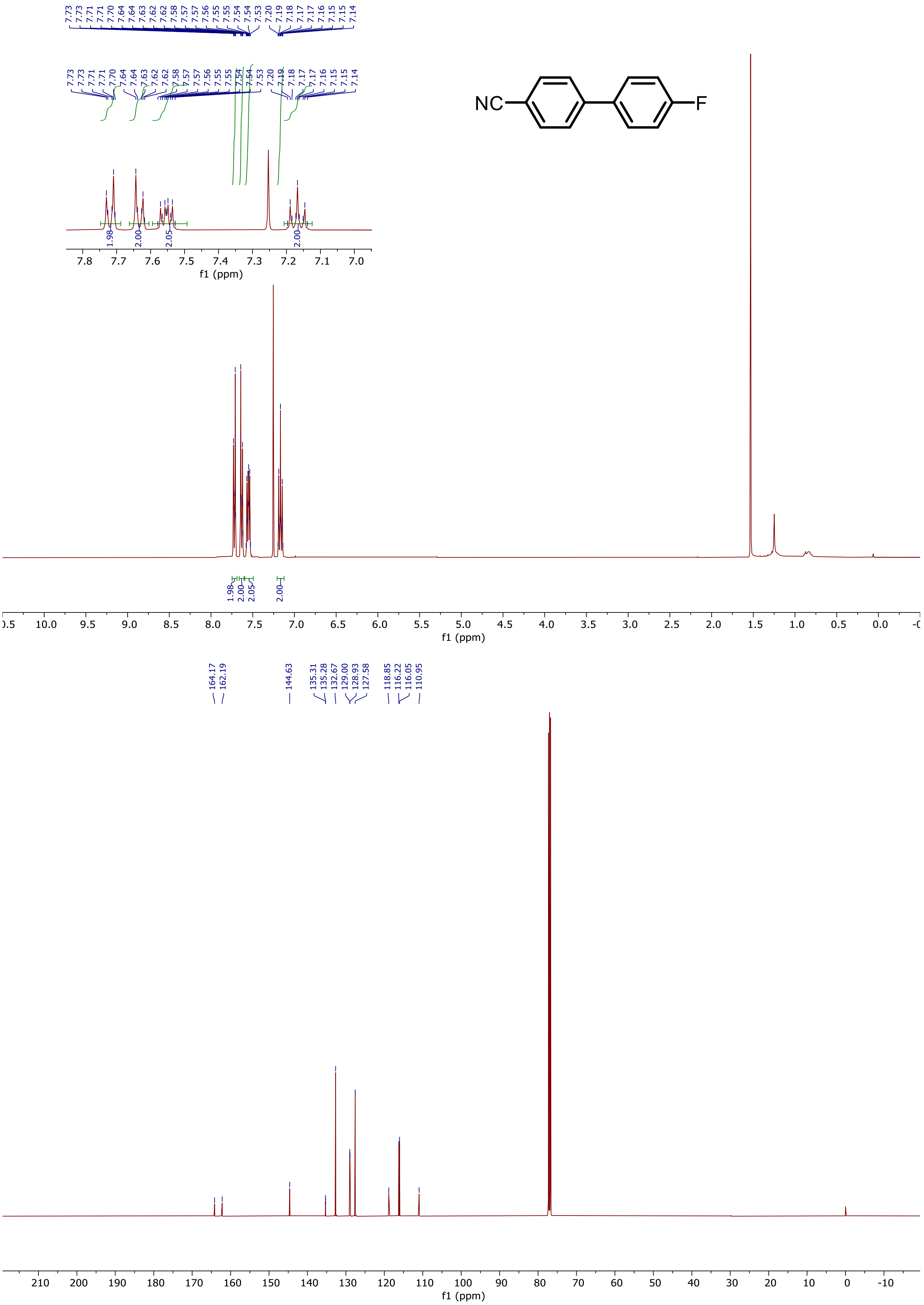

## Methyl 4'-fluoro-[1,1'-biphenyl]-4-carboxylate (3u)

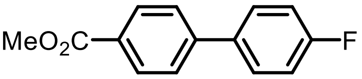


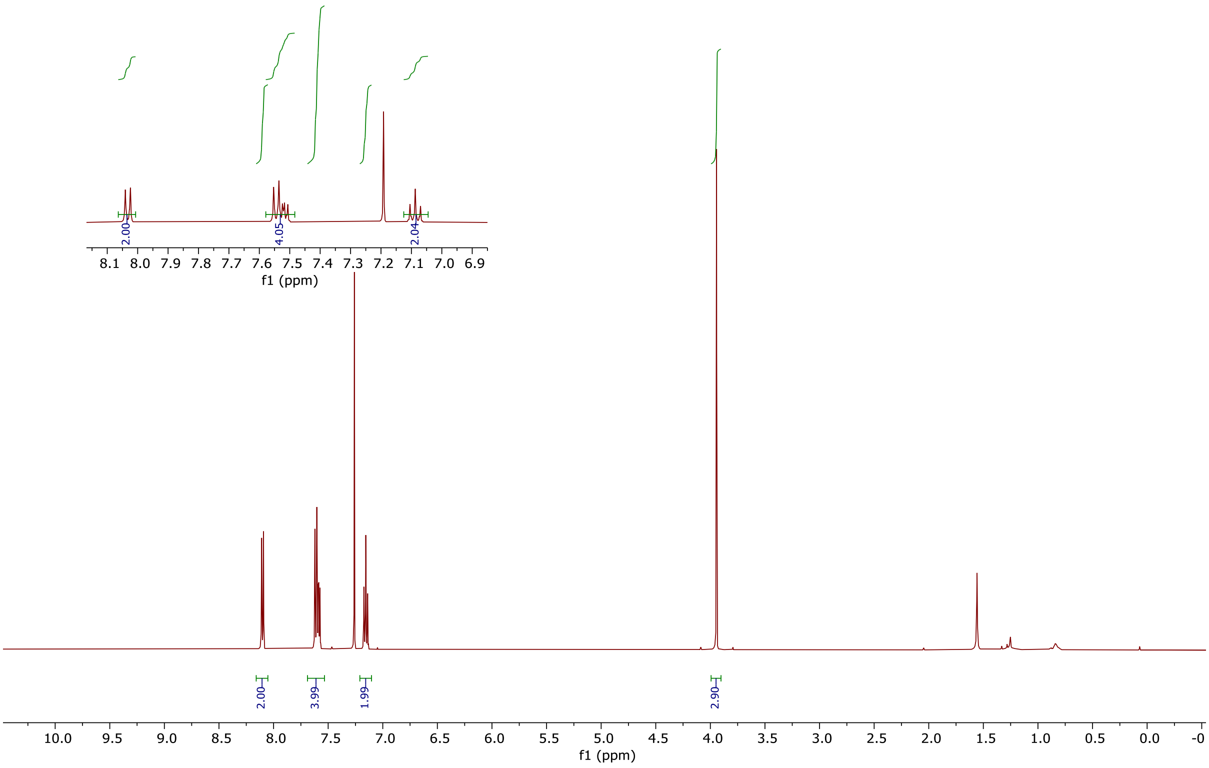


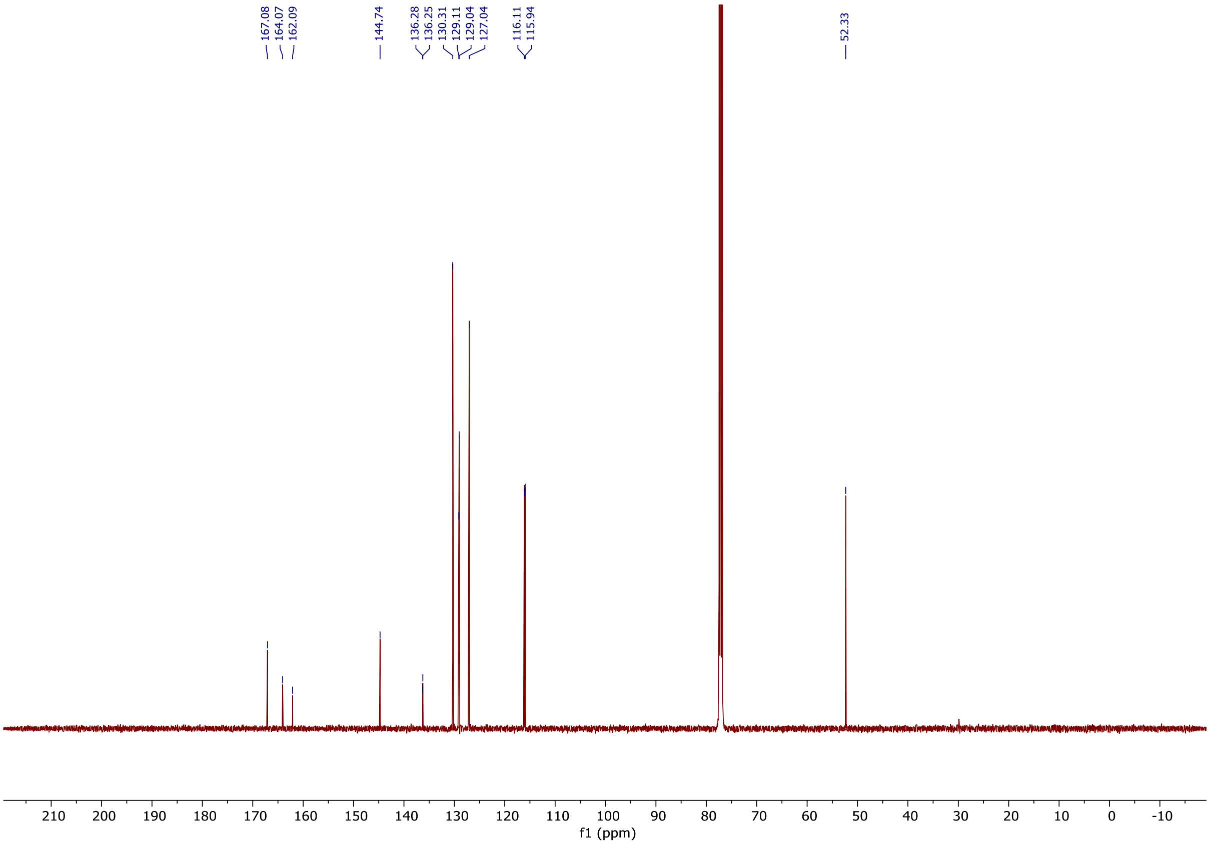

## 2-(4-Fluorophenyl)-pyridine (3v)

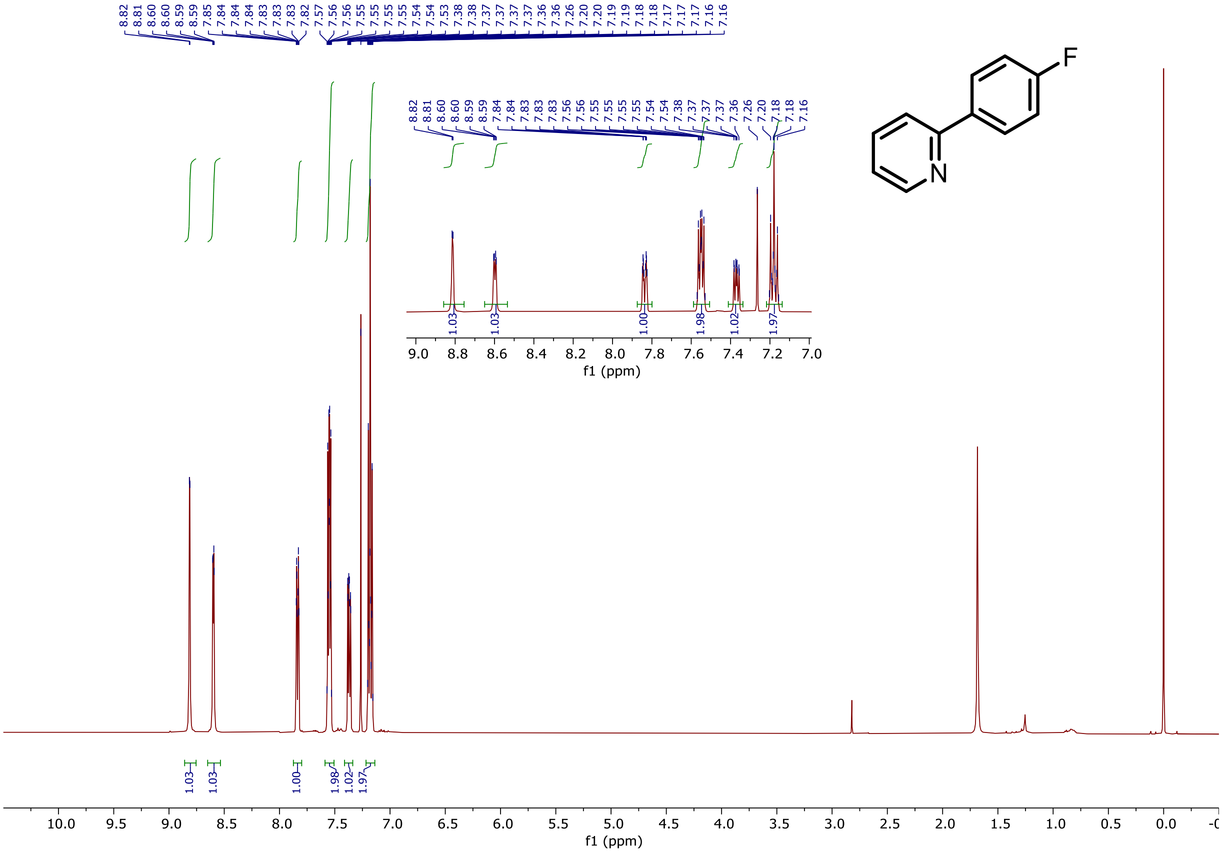

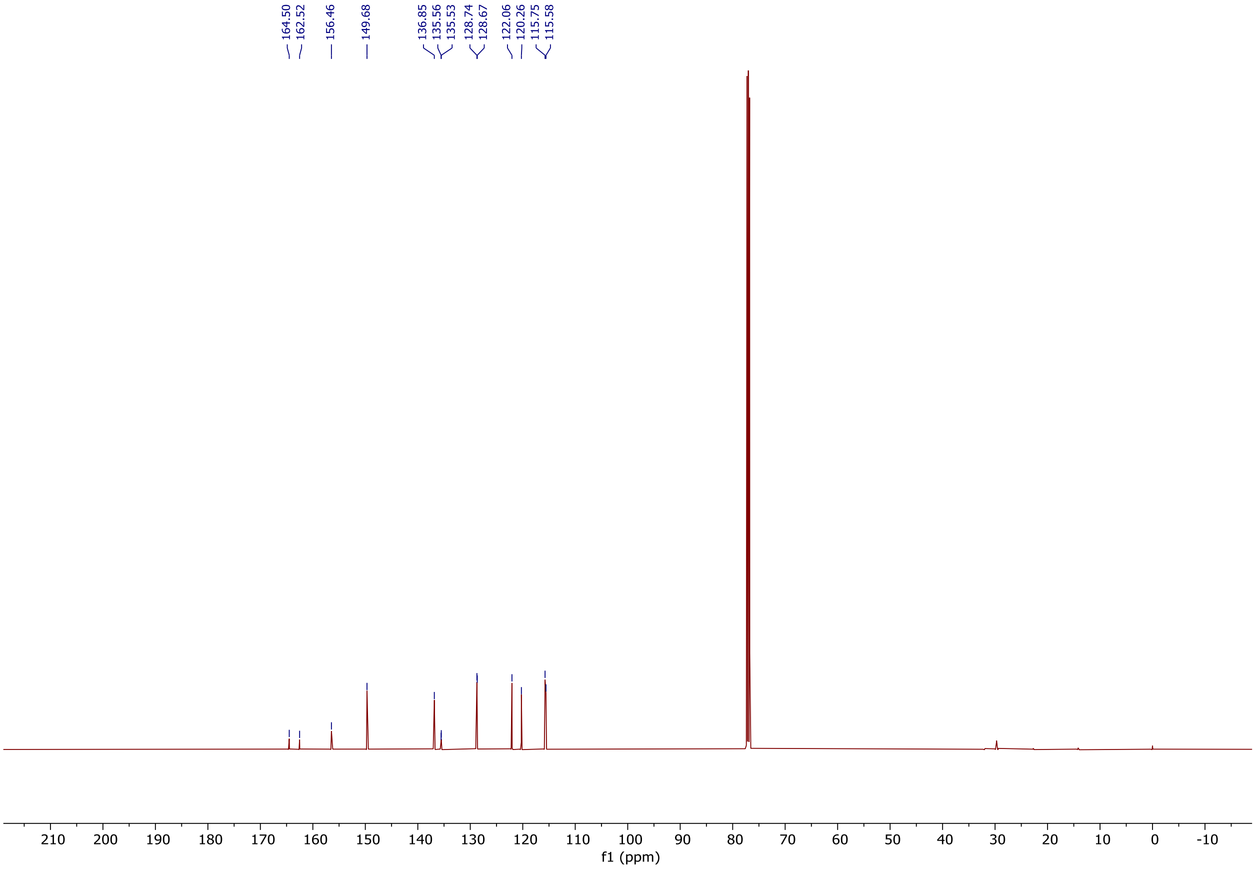

# 3-(4-Fluorophenyl)-pyridine (3w)

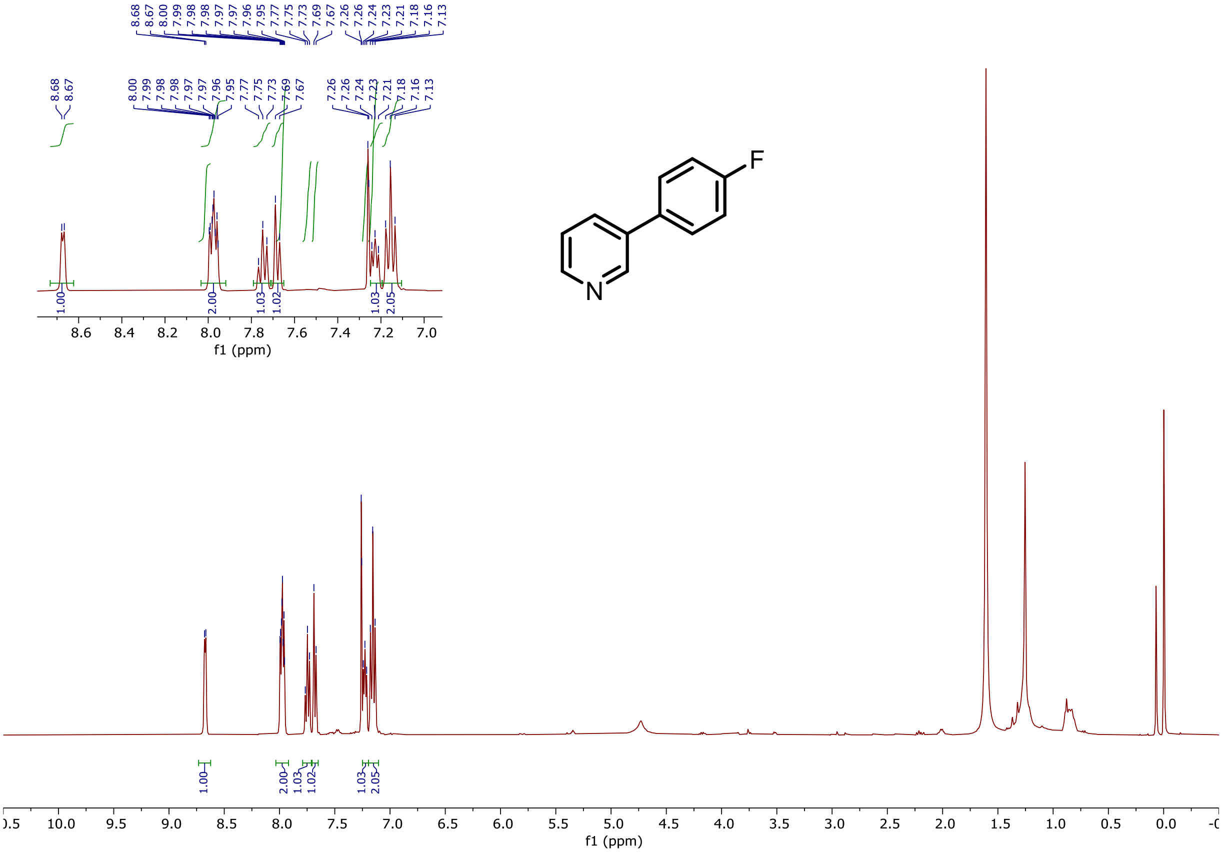


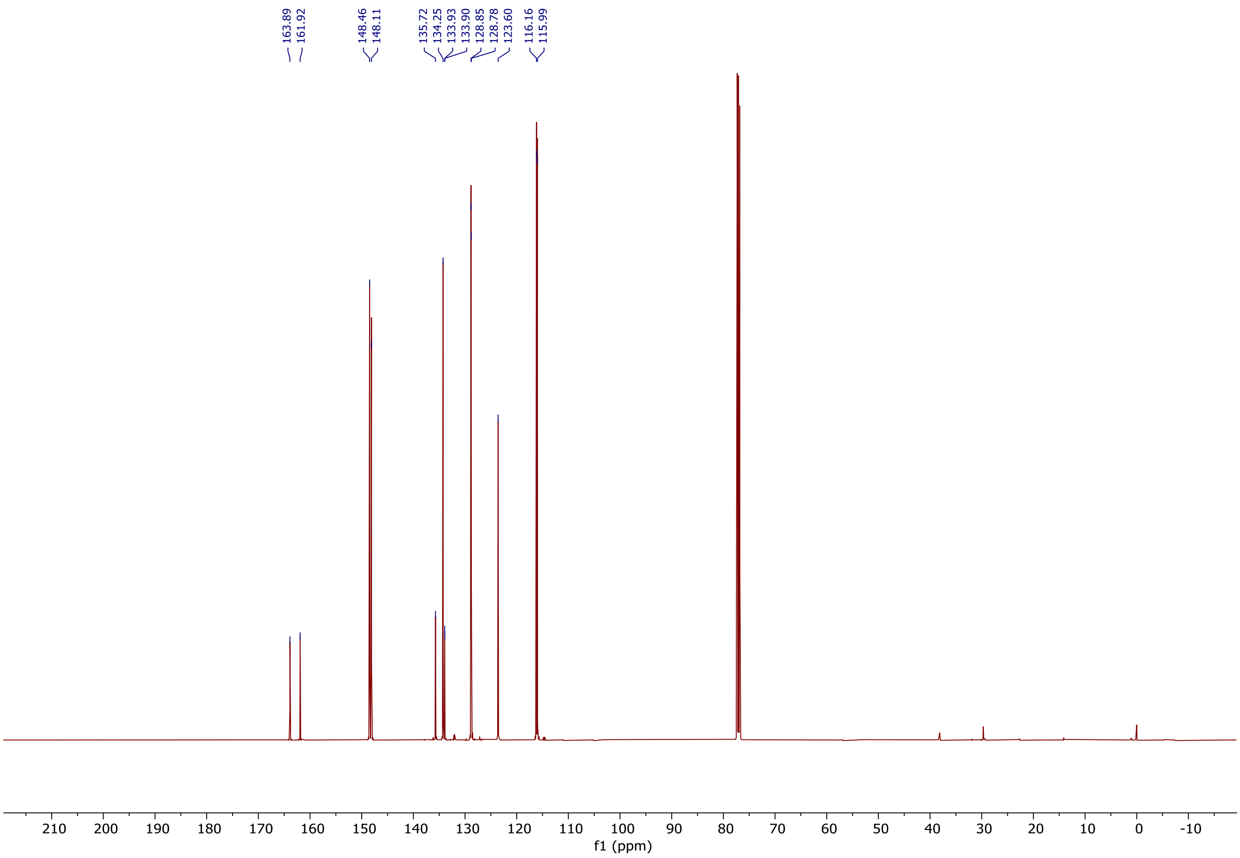

**1-(4-fluorophenyl)pyrene (3x)**

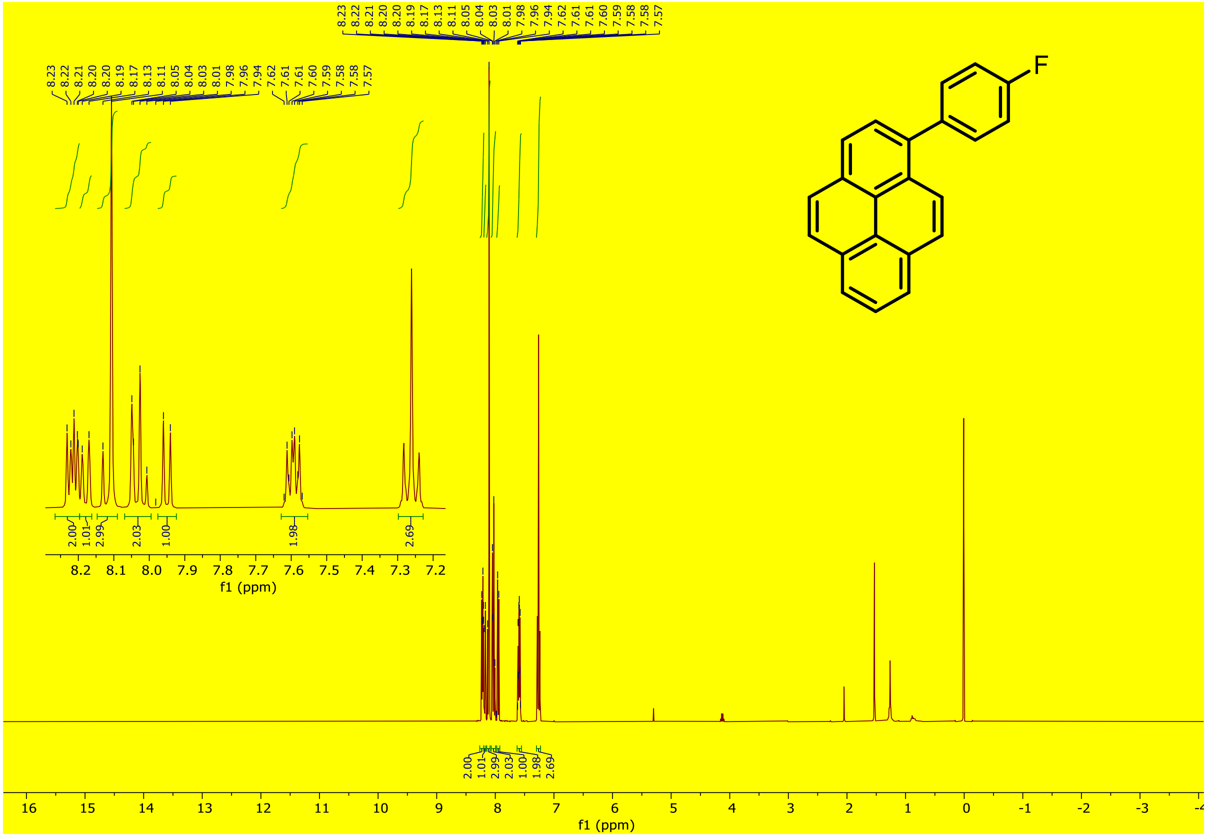

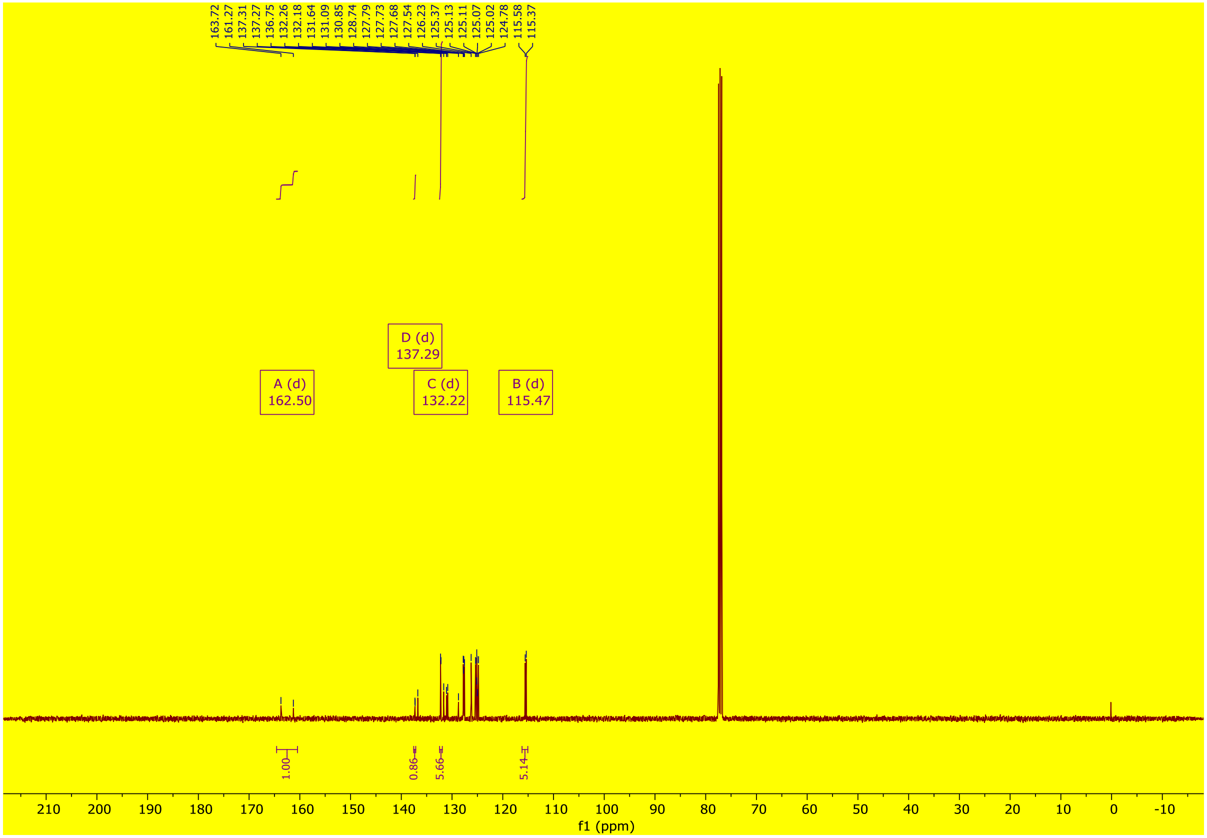

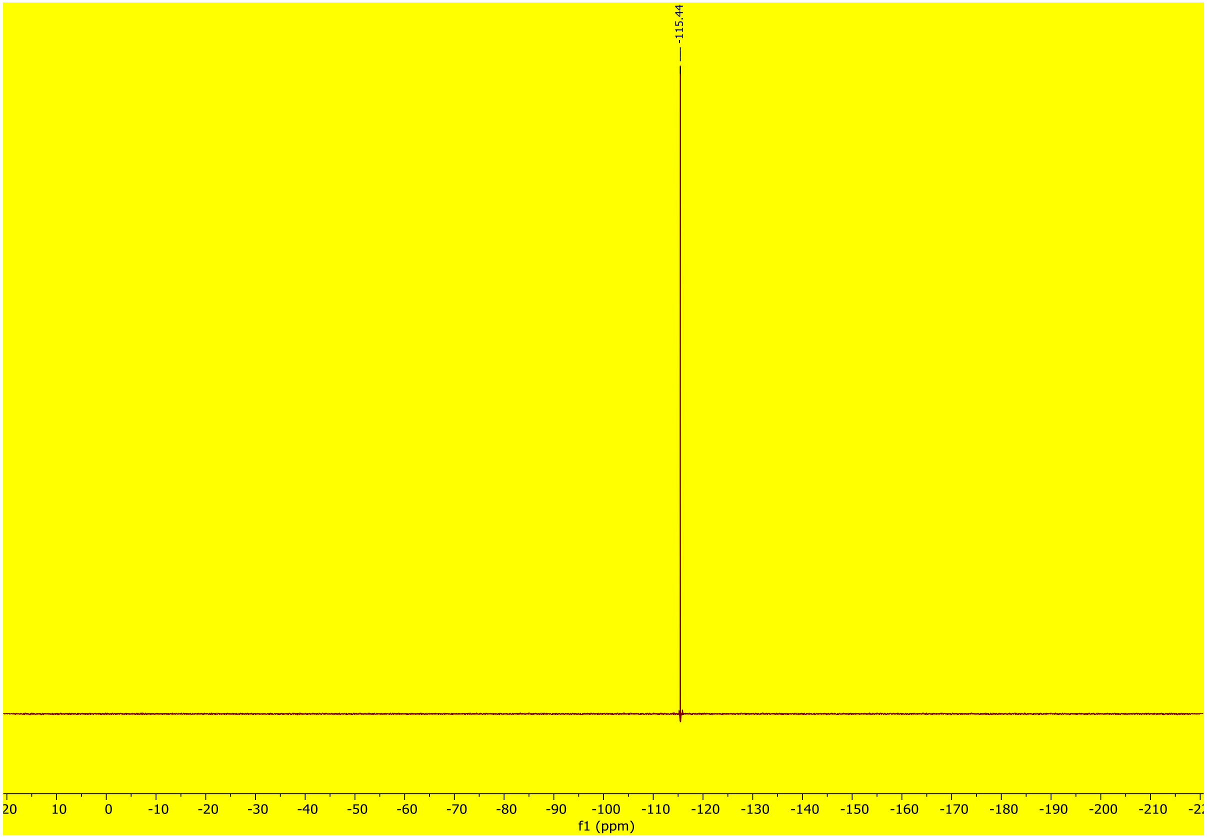


**4,4’-Difluoro-1,1’-biphenyl (4a)**

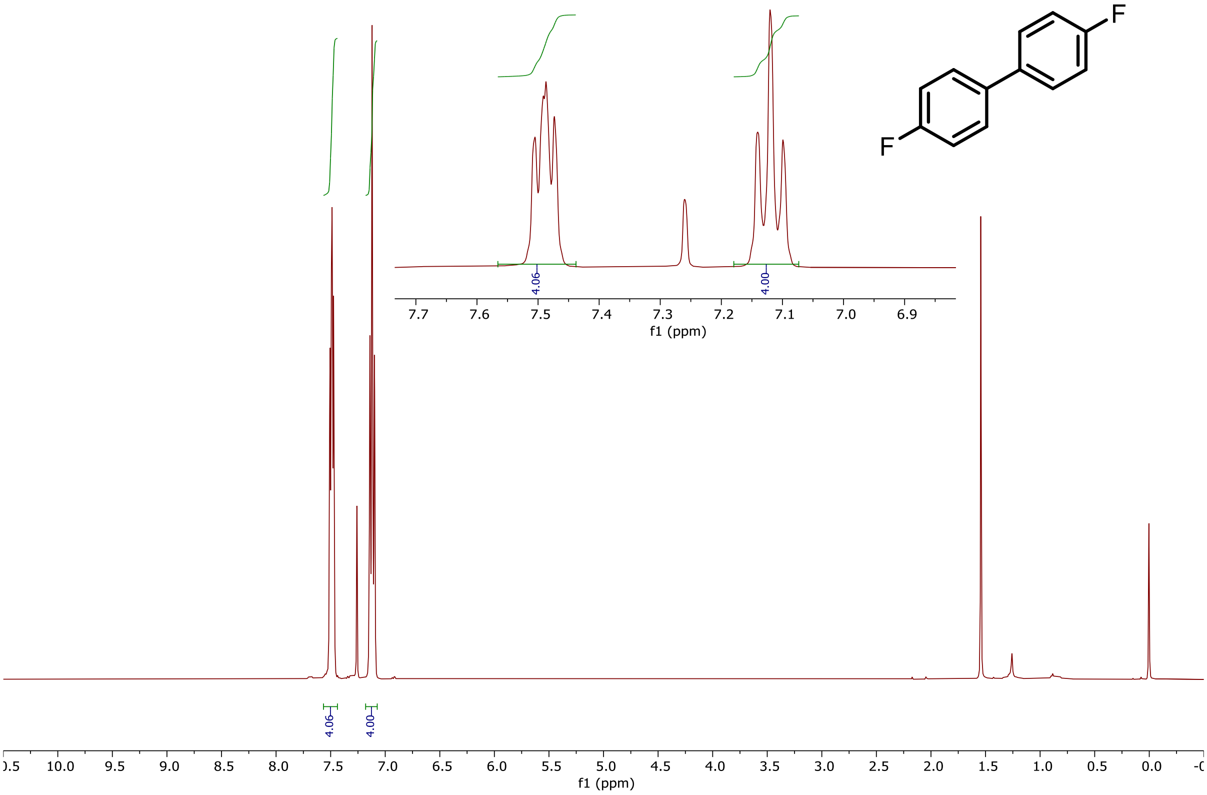

## 13. References


[1] K. W. Quasdorf, X. Tian, N. K. Garg, *J. Am. Chem. Soc.* **2008**, *130*, 14422–14423.
[2] K. W. Quasdorf, M. Riener, K. V. Petrova, N. K. Garg, *J. Am. Chem. Soc.* **2009**, *131*, 17748–17749.
[3] P. Boehm, S. Roediger, A. Bismuto, B. Morandi, *Angew. Chem. Int. Ed.* **2020**, *59*, 17887–17896.
[4] S. Zhu, C. Wang, L. Chen, R. Liang, Y. Yu, H. Jiang, *Org. Lett.* **2011**, *13*, 1146–1149.
[5] J. Wang, J. Zhao, H. Gong, *Chem. Commun.* **2017**, *53*, 10180–10183.
[6] D. A. Wilson, C. J. Wilson, C. Moldoveanu, A.-M. Resmerita, P. Corcoran, L. M. Hoang, B. M. Rosen, V. Percec, *J. Am. Chem. Soc.* **2010**, *132*, 1800–1801.
[7] B. Song, T. Knauber, L. J. Gooßen, *Angew. Chem. Int. Ed.* **2013**, *52*, 2954–2958.
[8] P. Leowanawat, N. Zhang, A.-M. Resmerita, B. M. Rosen, V. Percec, *J. Org. Chem.* **2011**, *76*, 9946–9955.
[9] K. W. Quasdorf, A. Antoft-Finch, P. Liu, A. L. Silberstein, A. Komaromi, T. Blackburn, S. D. Ramgren, K. N. Houk, V. Snieckus, N. K. Garg, *J. Am. Chem. Soc.* **2011**, *133*, 6352–6363.
[10] T. Agrawal, S. P. Cook, *Org. Lett.* **2013**, *15*, 96–99.
[11] S. D. Ramgren, A. L. Silberstein, Y. Yang, N. K. Garg, *Angew. Chem. Int. Ed.* **2011**, *50*, 2171–2173.
[12] B.-T. Guan, Y. Wang, B.-J. Li, D.-G. Yu, Z.-J. Shi, *J. Am. Chem. Soc.* **2008**, *130*, 14468–14470.
[13] H.-C. Shen, S. Pal, J.-J. Lian, R.-S. Liu, *J. Am. Chem. Soc.* **2003**, *125*, 15762–15763.
[14] J.-Y. Zhou, R.-Q. Liu, C.-Y. Wang, Y.-M. Zhu, *J. Org. Chem.* **2020**, *85*, 14149–14157.
[15] C. Qin, W. Lu, *J. Org. Chem.* **2008**, *73*, 7424–7427.
[16] M. I. Dawson, P. D. Hobbs, K. A. Derdzinski, W. R. Chao, G. Frenking, G. H. Loew, A. M. Jetten, J. L. Napoli, J. B. Williams, *J. Med. Chem.* **1989**, *32*, 1504–1517.
[17] G. A. Molander, D. E. Petrillo, N. R. Landzberg, J. C. Rohanna, B. Biolatto, *Synlett,* **2005**, *11*, 1763–1766
[18] J. L. Bolliger, C. M. Frech, *Adv. Synth. Catal.* **2010**, *352*, 1075–1080.
[19] J. M. Antelo Miguez, L. A. Adrio, A. Sousa-Pedrares, J. M. Vila, K. K. (Mimi) Hii, *J. Org. Chem.* **2007**, *72*, 7771–7774.
[20] S. E. Denmark, R. C. Smith, W.-T. T. Chang, J. M. Muhuhi, *J. Am. Chem. Soc.* **2009**, *131*, 3104–3118.
[21] S. Bernhardt, G. Manolikakes, T. Kunz, P. Knochel, *Angew. Chem. Int. Ed.* **2011**, *50*, 9205–9209.
[22] L. Wu, B.-L. Li, Y.-Y. Huang, H.-F. Zhou, Y.-M. He, Q.-H. Fan, *Org. Lett.* **2006**, *8*, 3605–3608.
[23] L. Zhi, H. Zhang, Z. Yang, W. Liu, B. Wang, *Chem. Commun.* **2016**, *52*, 6431–6434.
[24] N. Louaisil, P. D. Pham, F. Boeda, D. Faye, A.-S. Castanet, S. Legoupy, *Eur. J. Org. Chem.* **2011**, *2011*, 143–149.
[25] L. Yang, T. Zeng, Q. Shuai, X. Guo, C.-J. Li, *Chem. Commun.* **2011**, *47*, 2161–2163.
